# MaRe: Processing Big Data with application containers on Apache Spark

**DOI:** 10.1093/gigascience/giaa042

**Published:** 2020-05-05

**Authors:** Marco Capuccini, Martin Dahlö, Salman Toor, Ola Spjuth

**Affiliations:** 1 Department of Information Technology, Uppsala University, Box 337, 75105, Uppsala, Sweden; 2 Department of Pharmaceutical Biosciences, Uppsala University, Box 591, 751 24, Uppsala, Sweden; 3 Science for Life Laboratory, Uppsala University, Box 591, 751 24, Uppsala, Sweden; 4 Uppsala Multidisciplinary Center for Advanced Computational Science, Uppsala University, Box 337, 75105, Uppsala, Sweden

**Keywords:** MapReduce, application containers, Big Data, Apache Spark, workflows

## Abstract

**Background:**

Life science is increasingly driven by Big Data analytics, and the MapReduce programming model has been proven successful for data-intensive analyses. However, current MapReduce frameworks offer poor support for reusing existing processing tools in bioinformatics pipelines. Furthermore, these frameworks do not have native support for application containers, which are becoming popular in scientific data processing.

**Results:**

Here we present MaRe, an open source programming library that introduces support for Docker containers in Apache Spark. Apache Spark and Docker are the MapReduce framework and container engine that have collected the largest open source community; thus, MaRe provides interoperability with the cutting-edge software ecosystem. We demonstrate MaRe on 2 data-intensive applications in life science, showing ease of use and scalability.

**Conclusions:**

MaRe enables scalable data-intensive processing in life science with Apache Spark and application containers. When compared with current best practices, which involve the use of workflow systems, MaRe has the advantage of providing data locality, ingestion from heterogeneous storage systems, and interactive processing. MaRe is generally applicable and available as open source software.

## Findings

### Background and purpose

Life science is increasingly driven by Big Data analytics. From genomics, proteomics, and metabolomics to bioimaging and drug discovery, scientists need to analyze larger and larger amounts of data [1–5]. This means that datasets can no longer be stored and processed in a researcher’s workstation but instead need to be handled on distributed systems, at the organization level. For instance, the European Bioinformatics Institute, in Hinxton (United Kingdom), offers a total storage capacity of >160 petabytes for biologically significant data [6]. Such amounts of data pose major challenges for scientific analyses. First, there is a need to efficiently scale existing processing tools over massive datasets. In fact, bioinformatics software that was originally developed with the simplistic view of small-scale data will not scale on distributed computing platforms out of the box. The process of adapting such tools may introduce disruptive changes to the existing codebase, and it is generally unsustainable for most organizations. Second, the complexity in programming distributed systems may be hard to cope with for most researchers, who instead need to focus on the biological problem at hand. In addition, because life science is exploratory, scientists increasingly demand the ability to run interactive analyses rather than submitting jobs to batch systems. Third, when handling Big Data in distributed systems, data locality is a major concern. Indeed, if once data could be shuffled with little regard, with massive datasets it is not only inefficient [7] but also prohibitively expensive in terms of power consumption—estimated to be on the order of several hundred thousand dollars per year for a single next-generation high-performance computing (HPC) cluster [8]. For geographically dispersed datasets, locality awareness becomes even more challenging because computing resources need to be dynamically acquired close to the data [9]. Cloud computing solves this problem by enabling the allocation of virtual infrastructure on demand [10]. However, heterogeneity in storage systems for cloud providers [11] makes it hard to abstract data ingestion from many different sources. Finally, because bioinformatics software is characterized by complex software dependencies, deploying and managing a vast collection of tools in a large distributed system also represents a major challenge [12].

Current bionformatics best practices make use of workflow systems to orchestrate analyses over distributed computing platforms [13]. Workflow systems provide high-level APIs that allow for defining an execution graph of existing processing tools. At run time, the execution graph is used to pipeline the analysis on distributed cloud or HPC resources. Hence, the parallelization of the analysis is transparently carried out, by executing non-dependent tasks at the same time. Cutting-edge workflow systems, such as Luigi [14], NextFlow [15], Galaxy [16], and Pachyderm [17], allow for running processing tools as application containers. This lightweight packaging technology allows for encapsulating complete software environments, so that distributed systems can run the processing tools with no need of additional dependencies, in an isolated manner [18]. Hence, container-enabled workflow systems provide a fairly easy way to define distributed analyses comprising existing bioinformatics tools, and eliminating the need for managing complex software delivery process and dependency management. Nevertheless, workflow-oriented processing falls short when it comes to Big Data analyses. To the best of our knowledge, all of these systems use a decoupled shared storage system for synchronization and intermediate results storage. When dealing with large datasets, this translates to a massive and unnecessary communication in the underlying infrastructure. In addition, workflow systems usually support a limited amount of storage back ends, not seldom only POSIX file systems, making it hard to ingest data from heterogeneous cloud resources. Finally, owing to their batch-oriented nature, it is also intrinsically hard to enable interactive, exploratory analyses using workflow-oriented frameworks.

Google’s MapReduce programming model and its associated implementation pioneered uncomplicated Big Data analytics on distributed computing platforms [19]. When MapReduce is used, the analysis is defined in a high-level programming language that hides challenging parallel programming details including fault tolerance, data distribution, and locality-aware scheduling. Open source implementations of MapReduce are well established in industrial and scientific applications [20, 21], and numerous success stories in life science have been reported [22–24].

Apache Spark has emerged as the project that collected the largest community in the open source MapReduce ecosystems [25]. In addition to the MapReduce implementation, Apache Spark also provides increasingly important features, such as in-memory, interactive, and stream processing. Furthermore, owing to broad collaborations in the open source community, Apache Spark supports all of the major storage systems, enabling data ingestion from heterogeneous cloud resources. These characteristics are particularly appealing for the case of Big Data in life science. Nevertheless, Apache Spark and other similar frameworks offer poor support for composing analyses out of existing processing tools. This is usually limited to calling external programs, which can only access data sequentially, without support for application containers [26]. In fact, the main way of implementing analytics in MapReduce-oriented environments is to code each transformation using 1 of the available APIs. This way of implementing analyses contrasts with current best practices in bioinformatics, which promote the use of existing tools as application containers with the goal of improving the delivery, interoperability, and reproducibility of scientific pipelines [15].

Here we introduce MaRe—an open source programming library that extends Apache Spark, introducing comprehensive support for external tools and application containers in MapReduce. Similarly to container-enabled workflow systems, MaRe allows analyses to be defined in a high-level language, in which data transformations are performed by application containers. In addition, MaRe provides seamless management of data locality, as well as full interoperability, with the Apache Spark ecosystem. This last point allows MaRe analyses to ingest data from heterogeneous cloud storage systems and also provides support for interactive processing. Finally, by supporting Docker, the de facto standard container engine [27], MaRe is compatible with numerous existing container images.

In summary, the key contributions of the presented work are as follows:

- We introduce MaRe, an open source MapReduce-oriented programming library for container-based data processing on top of Apache Spark.
- We benchmark MaRe on 2 data-intensive applications in life science, showing ease of use and scalability.

### MaRe

#### Programming model

We introduce the MaRe programming model using a simple, yet interesting, example in genomics. A DNA sequence can be represented as a text file written in a language of 4 characters: A, T, G, C. The guanine-cytosine (GC) content in a DNA sequence has interesting biological implications; for instance, there is evidence that GC-rich genes are expressed more efficiently than GC-poor genes [28]. Hence, within a large DNA sequence it can be interesting to count G and C occurrences. Given an Ubuntu Docker image [29], the task can easily be implemented in MaRe using POSIX tools. Listing 1 shows such an implementation.

**Listing 1.** GC count in MaRe

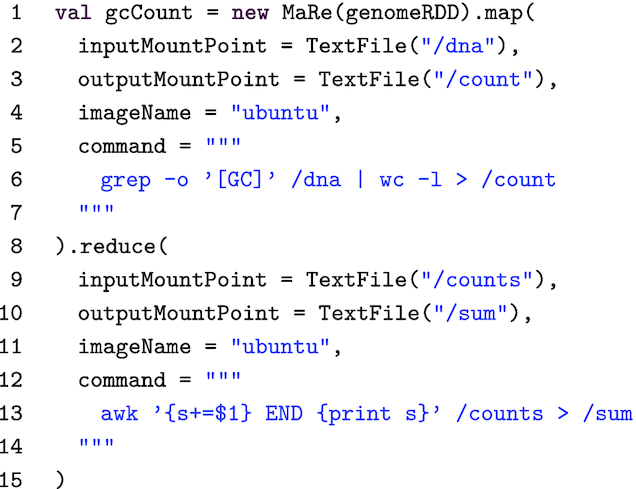

Being based on Apache Spark, MaRe has a similar programming model. The control flow of the analysis is coded in Scala [30], by the program in Listing 1. Such a program is called a "driver" in the Apache Spark terminology. The driver program can be packaged and submitted to a cluster (in batch mode), or executed interactively using a notebook environment such as Jupyter [31] or Apache Zeppelin [32]. Listing 1 starts by instantiating a MaRe object, which takes a resilient distributed dataset (RDD) [33], containing the input genome file in text format. Such an RDD can be easily loaded using the Apache Spark API from any of the supported storage backends. The map primitive (line 1–8) applies a command from the Docker image to each partition of the RDD. In our example we specify the Ubuntu image on line 4, and we use a command that combines grep and wc to filter and count GC occurrences (on line 6). The partitions are mounted in the Docker containers at the configured input mount point (”/dna” at line 2), and the command results are loaded back to MaRe from the configured output mount point (”/count” on line 3). In the example we use "TextFile" mount points because the input data are in text format. By default, MaRe considers each line in a text file as a separate record, but custom record separators can also be configured using the TextFile constructor.

At this point it is important to mention that MaRe can also handle binary files. For such data formats, the driver program should specify mount points of type "BinaryFiles." In this case, each RDD record is considered as a distinct binary file; thus, the specified mount point results in a directory containing multiple files (as opposed to TextFile, which mounts the records in a single file). We provide an example of the BinaryFiles mount point in the Evaluation section.

Coming back to Listing 1, after applying the "map" primitive, each RDD partition is transformed into a distinct GC count. The "reduce" primitive (lines 8–15) aggregates the counts in each partition to a cumulative sum. Again, we use mount points of type TextFile to mount the intermediate counts in the containers (”/counts” on line 9) and to read back the cumulative sum (”/sum” on line 10). The sum is computed using the awk command from the Ubuntu image (lines 11–14). Finally, the result is returned to the gcCount variable at line 1.

From the GC example, the reader may have noticed that our programming model is strongly inspired by MapReduce. In addition, Apache Spark users may have noticed that the GC count problem can easily be solved in pure Spark code. Indeed, the aim of the example is just to provide an easy introduction to MaRe, and 2 real-world applications are available in the Evaluation section.

Apart from map and reduce, MaRe provides an additional primitive. For real-world applications, we noticed that it is often necessary to group dataset records according to a specific logic before applying map or reduce. For this reason, MaRe also provides a "repartitionBy" primitive, which repartitions the RDD records according to a configurable grouping rule. More specifically, the repartitionBy primitive takes into account a user-provided keyBy function, which is used to compute a key for each record in the dataset. Then, the repartitioning is performed accordingly so that records with the same key end up in the same partition. An example of repartitionBy is available in the Evaluation section.

#### Implementation

MaRe comes as a thin layer on top of the RDD API [33], and it relies on Apache Spark to provide important features such as data locality, data ingestion, interactive processing, and fault tolerance. The implementation effort consists of (i) leveraging the RDD API to implement the MaRe primitives and (ii) handling data between containers and RDD structures.

##### Primitives

Each instance of a MaRe object retains an underlying RDD, which represents an abstraction of a dataset that is partitioned across Apache Spark workers. The map, reduce, and repartitionBy primitives utilize the underlying RDD API to operate such a dataset.

**Figure 1:**
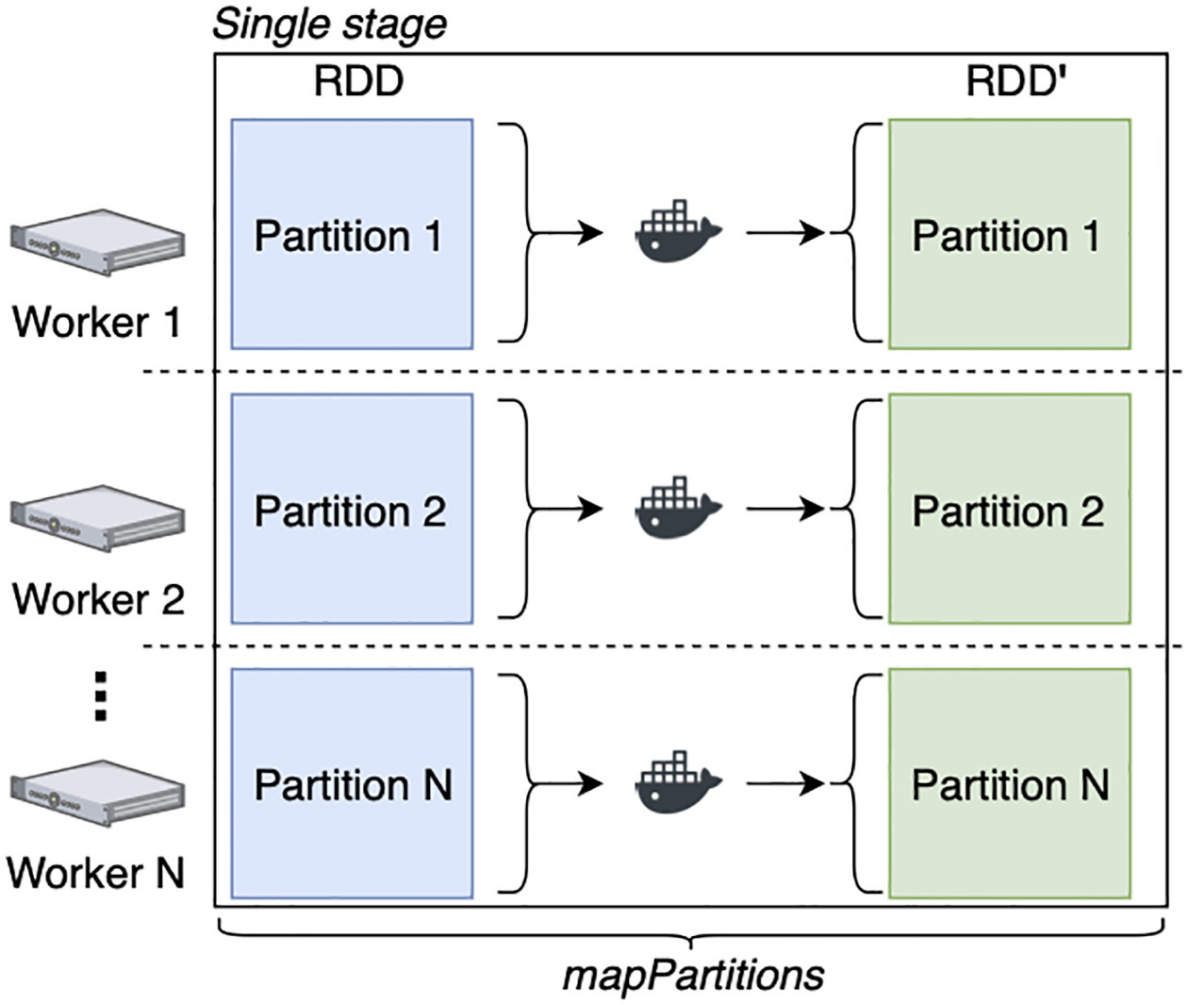
Execution diagram for the map primitive. The primitive takes an RDD that is partitioned over *N* nodes, it transforms each partition using a Docker container, and it returns a new RDD′. The logic is implemented using mapPartitions from the RDD API. Because mapPartitions generates a single stage, data are not shuffled between nodes.

Fig. 1 shows the execution diagram for the map primitive. For simplicity, in Fig. 1 we show a single partition per worker, but in reality workers may retain multiple partitions. This primitive takes an input RDD that is partitioned over *N* nodes, and it transforms each partition using a Docker container command—thus returning a new RDD′. This logic is implemented using "mapPartitions" from the RDD API. When calling mapPartitions, MaRe specifies a lambda expression that (i) makes the data available in the input mount point, (ii) runs the Docker container, and (iii) retrieves the results from the output mount point. When using mapPartitions, Apache Spark generates a single stage; thus, no data shuffle is performed.

**Figure 2:**
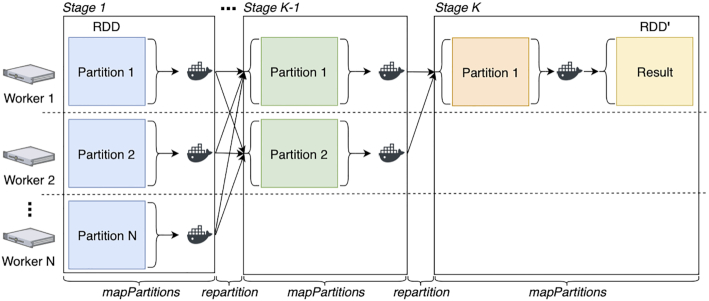
Execution diagram for the reduce primitive. The primitive takes an input RDD, partitioned over *N* nodes, and it iteratively aggregates records using a Docker container, reducing the number of partitions until an RDD′, containing a single result partition, is returned. The logic is implemented using mapPartitions and repartition from the RDD API, to aggregate records in partitions and to decrease the number of partitions, respectively. Because repartition is called in each of the *K* iterations, *K* stages are generated, giving place to *K* data shuffles.

Fig. 2 shows the execution diagram for the reduce primitive. This primitive takes an input RDD, partitioned over *N* nodes, and it iteratively aggregates records, reducing the number of partitions until an RDD′, containing a single result partition, is returned. Again, the input RDD may retain multiple partitions per node. However, as opposed to the map primitive, RDD′ always contains a single partition when it is returned. Given a user-configured depth *K*, the records in the RDD are aggregated using a tree-like algorithm. In each of the *K* levels in the tree, the records within each partition are first aggregated using a Docker container command. Like the map primitive, this first transformation is implemented using "mapPartitions" from the RDD API. Then, the number of partitions is decreased using "repartition" from the RDD API. This process is repeated *K* times until 1 single partition is left. At this point the records within the remaining partition are aggregated again using mapPartitions (from the RDD API), and RDD′ is returned. A new stage is generated each time repartition is used. Hence, reduce leads to *K* data shuffles. For this reason, when aggregating records, the user-provided command should always reduce the size of the partition. In addition, for consistent results, the command should perform an associative and commutative operation. By default MaRe sets *K* to 2; however, the user may chose a higher tree depth when it is not possible to sufficiently reduce the dataset size in 1 go.

Finally, the repartitionBy primitive is implemented by using keyBy and then repartition from the RDD API. MaRe uses the user-provided grouping rule with keyBy to compute a key for each RDD record, and then it applies repartition in conjunction with HashPartitioner [34], which makes sure that records with the same key end up in the same partition.

##### Data handling

One of the advantages of Apache Spark over other MapReduce-like systems is its ability to retain data in memory. To achieve this when passing the data to the application containers, there are a few options available: (i) Unix pipes [35], (ii) memory-mapped files [36], and (iii) tmpfs [37]. Solutions (i) and (ii) are the most memory-efficient because they do not need to materialize the data when passing it to the containers. However, (i) allows records to be seen only once in a stream-like manner, while (ii) requires the container-wrapped tools to be able to read from a memory-mapped file. Apache Spark loads data in memory sequentially and partition-wise. Partition size is configurable and often equals the block size in the underlying storage system. For the Hadoop distributed file system (HDFS) this value defaults to 128 MB, meaning that on an 8-core machine materializing again partitions on an in-memory file system would require 2 GB of memory in total—which is usually not a problem for modern data centers. Therefore, to support any wrapped tool, we decided to start by implementing solution (iii). This means that MaRe uses an in-memory tmpfs file system as temporary file space for the input and output mount points. The solution allows a standard POSIX mount point to be provided to the containers, while still retaining reasonable performance [37]. However, MaRe also provides users with the option of selecting any other disk-based file system for the temporary mount points. Even if this could in principle edge performance, this can be useful when a dockerized tool does not allow for splitting large partitions in smaller chunks of records; we show an example of this in the Evaluation section.

### Evaluation

We evaluate MaRe on 2 data-intensive applications in life science. The first application can be decomposed to somewhat independent jobs, where the data assigned to each job can be relatively small. This is where MapReduce-oriented programming libraries such as MaRe excel. Conversely, the second application requires larger chunks of data to be computed all at once, thus allowing us to show the performance penalty that is introduced in such a case. We evaluate in more detail (i) how the analyses can be implemented in MaRe and (ii) how the analyses scale over multiple nodes. To the best of our knowledge, no stable Spark-native implementation of the tools presented in the analyses is publicly available, making a fair performance comparison with a system that does not delegate data processing to an external application container unfeasible. To this extent, we would like to add that if such implementation were available there would be no advantage in rewriting the analyses using our programming library.

The scalability experiments were carried out on cPouta, an OpenStack-based cloud service operated by the Information Technology Center for Science (CSC) in Finland [38]. The driver programs were run interactively using an Apache Zeppelin environment [32], and the notebooks were made available to sustain reproducibility [39]. In addition, we also made available a deployment automation that enables our set-up to be replicated on cPouta, as well as any other OpenStack-based cloud provider [40].

#### Virtual screening

Virtual screening (VS) is a computer-based method to identify potential drug candidates by evaluating the binding affinity of virtual compounds against a biological target protein [41]. Given a 3D target structure, a molecular docking software is run against a large library of known molecular representations. For each compound in the virtual molecular library the docking software produces a pose, representing the orientation of the molecule in the target structure, and a binding affinity score. The poses with the highest affinity scores can be considered as potential drug leads for the target protein.

VS is data intensive because molecular libraries usually contain millions of compounds. A simple, yet effective, approach to scale VS consists of (i) distributing the molecular library over several nodes, (ii) running the docking software in parallel, and (iii) aggregating the top-scoring poses. Listing 2 shows how this logic can be implemented in MaRe, using FRED [42] as molecular docking software and sdsorter [43] to filter the top-scoring poses.

**Listing 2.** Virtual screening in MaRe

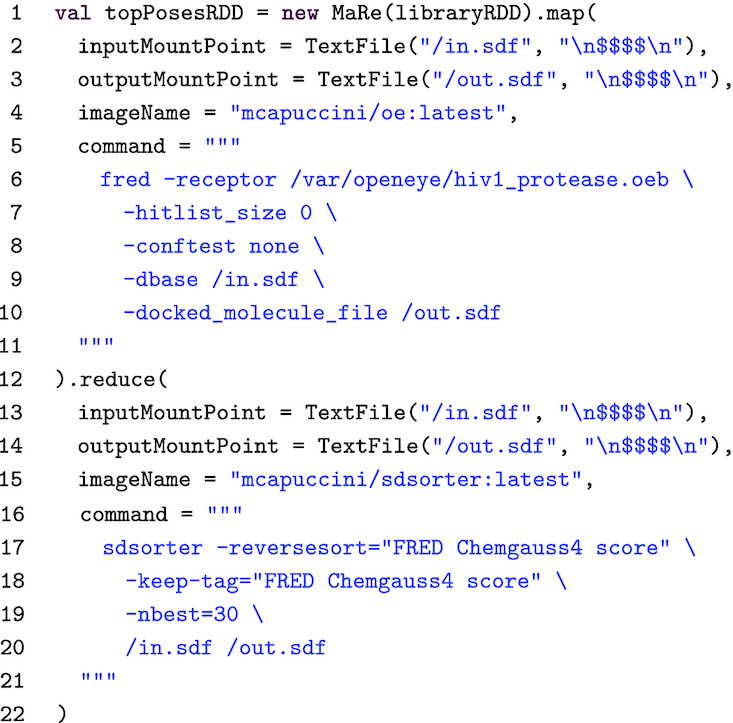

In Listing 2, we initialize MaRe by passing it a molecular library that was previously loaded as an RDD (libraryRDD on line 1). We implement the parallel molecular docking using the map primitive. On lines 2 and 3, we set input and output mount points as text files, and assuming the library to be in Structure-Data File (SDF) format [44] we use the custom record separator: "\n${\$\$\$\$}$\n”. On line 4, we specify a Docker image containing FRED. The image is not publicly available because it also contains our FRED license, but the license can be obtained free of charge for research purposes and we provide a Dockerfile [39] to build the image. On line 5, we specify the FRED command. We use an HIV-1 protease receptor [45] as target (which is wrapped in the Docker image), and we set (i) -hitlist_size 0 to not filter the poses in this stage, (ii) -conftest none to consider the input molecules as single conformations, (iii) -dbase /in.sdf to read the input molecules from the input mount point, and (iv) -docked_molecule_file /out.sdf to write the poses to the output mount point.

The map phase produces a pose for each molecule in libraryRDD. On line 12, we use the reduce primitive to filter the top 30 poses. On lines 13 and 14, we set the input and output mount points as we do for the map primitive. On line 15, we specify a publicly available Docker image containing sdsorter. On line 16, we specify the sdsorter command, and we set (i) -reversesort=”FRED Chemgauss4 score” to sort the poses from highest to lowest FRED score, (ii) -keep-tag=”FRED Chemgauss4 score to keep the score in the results, (iii) -nbest=30 to output the top 30 poses, and (iv) /in.sdf /out.sdf to read and write from the input mount point and to the output mount point, respectively. Note that this command performs an associative and commutative operation, thus ensuring correctness in the reduce phase. Finally, the results are returned to topPosesRDD, on line 1.

We benchmarked the analysis coded in Listing 2 against the SureChEMBL library [46] retrieved from the ZINC database [47], containing ∼2.2M molecules. The benchmark ran on top of a stand-alone Apache Spark cluster composed of 1 master and 12 worker nodes. Each node provided 10 cores and 43 GB of memory, thus resulting in a total of 120 cores and 516 GB of memory. The data were made available to the workers using a co-located HDFS storage. Under these settings, we evaluated the scalability in terms of weak scaling efficiency (WSE). This performance metric shows how the system scales when the amount of data and parallelism increase. To compute the WSEs we first ran the benchmark on 1/12 of the dataset using the dockerized tools on a worker node using their built-in, single-node parallelization. Then, we reran the pipeline using MaRe on 2/12, 4/12, 6/12, ... and 12/12 of the datasets, using 2, 4, 6, ...and 12 worker nodes, respectively. The WSE is then computed as the time for processing 1/12 of the data using the built-in, single-node parallelization, divided by the time for processing *N*/12 of the data using *N* nodes (for *N* = 2, 4, 6, ...,12). The ideal case, when the number of nodes is doubled, is to be able to process twice as much data in the same amount of time. Hence, a higher WSE indicates better performance.

**Figure 3:**
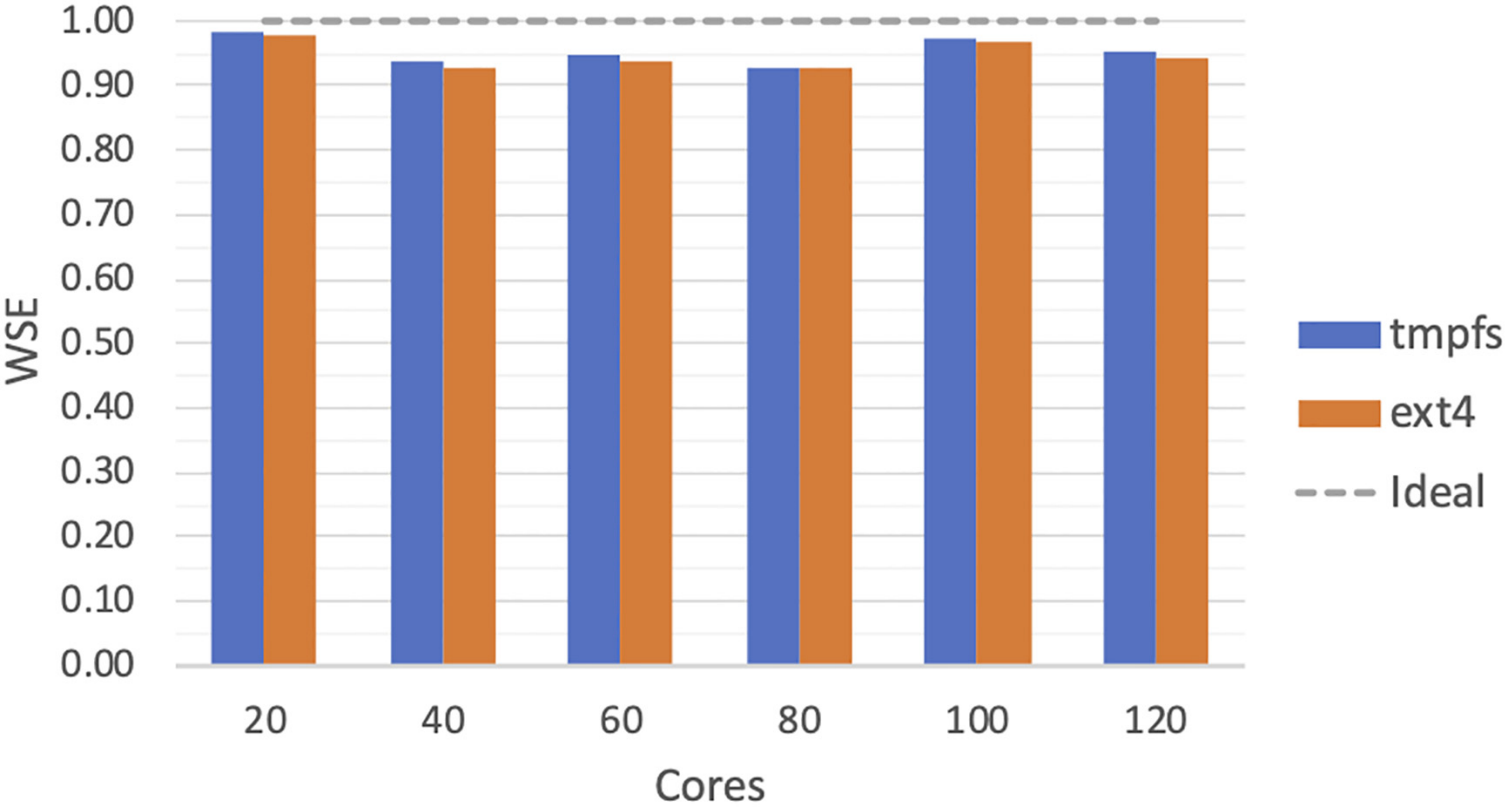
WSE for the VS application implemented in MaRe (Listing 2). The results are produced by using SureChEMBL as input, and we show the WSE when using tmpfs and ext4 as temporary mount point for passing the data to the containers.

Fig. 3 shows the WSE for the full analysis, when using tmpfs and a disk-based, ext4 file system [48] as temporary mount points. From the experiments it emerges that there is little difference between the 2 methods in terms of scaling efficiency—tmpfs improved the WSE by 0.02 at most. Indeed, the results in Fig. 3 indicate very good scalability, with a WSE close to ideal for both tmpfs and ext4. For 120 cores, the full benchmark ran in 2 hours and 21 minutes while 1/12 of the input data were processed by the built-in, single-node parallelization in 2 hours and 14 minutes—resulting in 0.94 WSE. This means that the overhead introduced by MaRe accounts for only 7 minutes in total.

Finally, to ensure the correctness of the parallelization, we ran sdsorter and FRED on a single core against 1,000 molecules that we randomly sampled from SureChEMBL, and we compared the results with those produced by the code in Listing 2.

#### Single-nucleotide polymorphism calling

A single-nucleotide polymorphism (SNP) is a position in a DNA sequence where a single nucleotide (or base pair) is different from another DNA sequence to which it is being compared [49]. When considering multiple samples, DNA sequences are usually compared individually to a reference genome: an agreed-upon sequence that is considered to represent an organism's genome. Once each DNA sequence has had its SNPs detected, or "called," the differences between the samples can be compared.

SNPs occur frequently. In fact, in humans roughly every 850th base pair is an SNP [50]. Calling SNPs has several use cases. For instance, SNPs can be used as high-resolution markers when comparing genomic regions between samples [51], as well as indicators of diseases in an individual [52]. Modern high-throughput sequencing methods for reading DNA often make use of a technique called "massively parallel sequencing" to read sequences longer than ∼200 bp, with a sufficiently small error rate. This is done by cleaving multiple copies of the source DNA into random fragments (called "reads") that are small enough to be accurately read, and then by aligning them to a reference genome. The overlapping fragments together form the sequence of the source DNA.

In order to accurately sequence 3 billion bases from a single human individual, 30-fold more read data need to be sequenced [1]. This makes SNP calling data-intensive, thus requiring parallelization. A simple MapReduce-oriented approach consists of (i) distributing the reads across several nodes, (ii) aligning the reads to a reference genome in parallel, and (iii) calling the SNPs with respect to the reference genome. The last step requires all the reads from a chromosome to be included in the SNP calling; thus, the maximum allowed parallelism is equal to the total number of chromosomes. Listing 3 shows how the described parallelization can be implemented in MaRe, using BWA for the alignment [53] and GATK [54] for the SNP calling. In contrast to the VS example, BWA and GATK provide a multithreaded implementation of the algorithms. Therefore, in Listing 3, we leverage this implementation for single-node parallelization.

**Listing 3.** SNP calling in MaRe

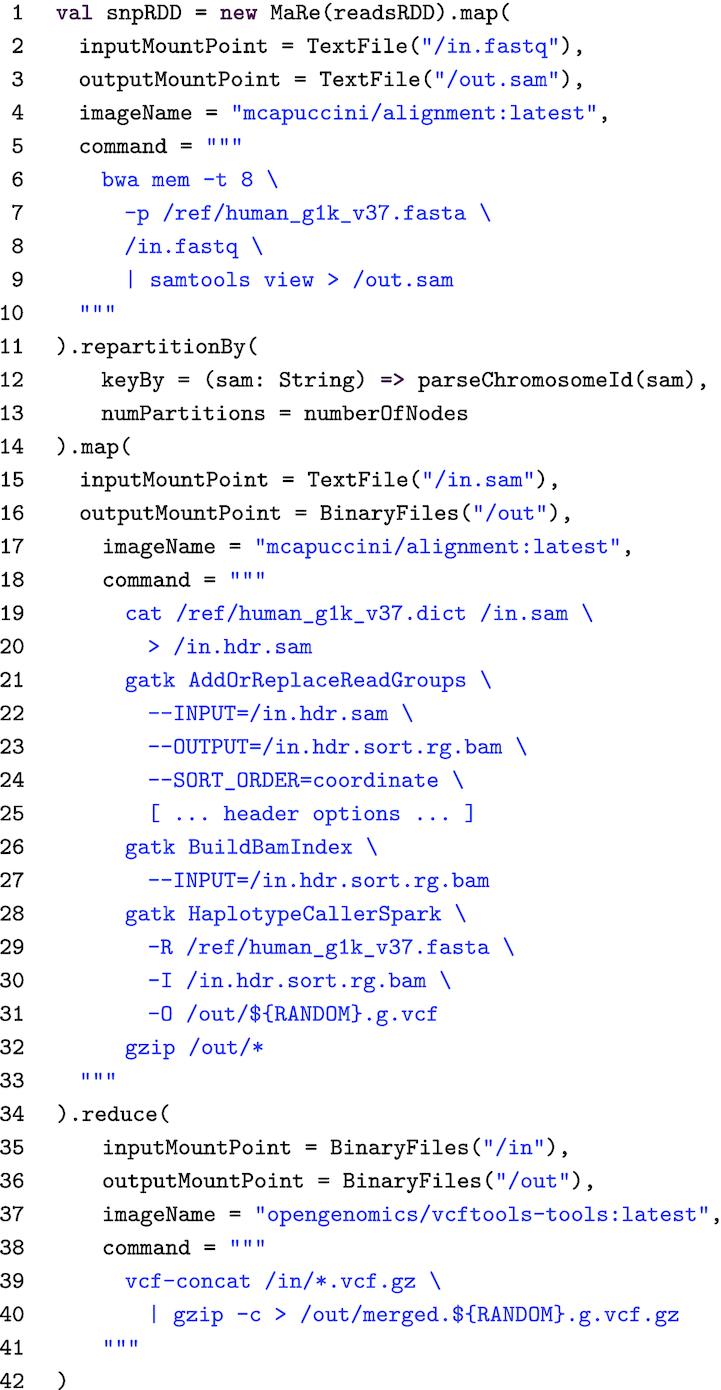

In Listing 3, MaRe is initialized by passing an RDD containing the reads for a human individual in interleaved FASTQ format [55] (readsRDD on line 1). We implement the parallel reads alignment using the map primitive. From lines 2 to 4, we set the mount points as text files, and we specify a publicly available Docker image containing the necessary software tools. On line 5 we specify the BWA command and we set (i) -t 8 to utilize 8 threads, (ii) -p /ref/human_g1k_v37.fasta to specify the reference genome location (in the container), and (iii) the input mount point /in.fastq. In addition, on line 9 we pipe the results to another piece of software, called samtools [56], to convert them from the binary BAM format [56] to the text SAM format [56]. Converting the results to text format makes it easier to parse the chromosome location in the next step.

When calling SNPs, GATK needs to read all of the aligned reads for a certain DNA region. Using chromosomes to define the regions makes sure that no reads will span a region break point—a problem that would need to be handled if chromosomes were to be split in smaller regions. To achieve this we need to (i) perform a chromosome-wise repartition of the dataset and (ii) allow MaRe to write temporary mount point data to disk. Point (ii) is enabled by setting the TMPDIR environment variable to a disk mount, in the Apache Zeppelin configuration. Even if this could potentially edge performance, this is necessary because the full partition size exceeds the tmpfs capacity in our worker nodes. Point (i) is implemented by using the repartitionBy primitive, on line 11. In particular, we specify a keyBy function that parses and returns the chromosome identifier (on line 12), and a number of partitions that is equal to the number of worker nodes (on line 13).

The map primitive (on line 14) uses the chromosome-wise partitioning to perform the SNP calling, with GATK. Because the data are in SAM format, we set the input mount point as text file (line 15). However, because we are going to zip the results before aggregating the SNPs (line 32), we set the output mount point as a binary files directory (”/out”, on line 16). On line 17, we set the same Docker image that we used for the initial mapping step, and, on line 18, we specify a command that (i) prepends the necessary SAM header to the input data (which is available inside the container under /ref/human_g1k_v37.dict, on line 19), (ii) coverts the SAM input to BAM format (line 23), (iii) builds an index for the BAM format (line 26), and (iv) runs the multithreaded SNP calling using GATK, producing a Variant Call Format (VCF) file [57] (line 28). A detailed description of the options used for each command can be found in the GATK documentation [58].

Finally, to aggregate the SNPs to a single zipped file, we use the reduce primitive. In this case we use binary file mount points (lines 35 and 36) and a publicly available image containing the VCFtools software [57] (line 37). On line 39, the specified command uses vcf-concat to merge all of the VCF files in the input mount point, and then it zips and writes them to the output mount point (line 40). Because MaRe applies the reduce command iteratively, intermediate partitions will contain multiple files. Therefore, to avoid file-name clashes, we include a random identifier in the command output (${RANDOM} at line 40).

We benchmarked the analysis in Listing 3 against the full individual reads dataset HG02666 (∼30 GB compressed FASTQ files), from the 1000 Genomes Project (1KGP) [50]. The benchmark ran on top of a stand-alone Apache Spark cluster composed of 1 master and 14 worker nodes. Each node provided 8 cores and 40 GB of memory, thus resulting in a total of 112 cores and 480 GB of memory. In addition, because after the chromosome-wise repartitioning, the partition size exceeded the tmpfs space in our workers, we used instance favors with a local solid state drive (SSD). This allowed the temporary mount point data to be written and read faster when compared to the previous benchmark. The data were made available to the workers using a co-located HDFS storage. Under these settings, we evaluated the scalability in terms of strong scaling efficiency (SSE). This performance metric shows how the system scales when the parallelism is increased while keeping the input size static. We evaluated this benchmark using SSE instead of WSE because there is no trivial way for downsampling the reference genome while keeping the behavior of the tools unaltered; the algorithms end up taking longer as they perform an exhaustive search when the reference genome is downsampled. To compute the SSEs we first ran the benchmark using the dockerized tools on a worker node with their built-in, single-node parallelization. Then, we reran the pipeline using MaRe on 6, 8, 10, 12, and 14 worker nodes. Then, letting *T*_1_ be the time for running the benchmark using the built-in, single-node parallelization and *T_N_* be the time for running the benchmark using *N* nodes (for *N* = 6, 8, 10, 12), we computed the SSE as *T*_1_/(*N* × *T_N_*) (we did not run on 2 and 4 nodes because the dataset size exceeded the total memory available to the Spark workers in these settings). The ideal case, when doubling the number of nodes, is to be able to run the benchmark twice as fast. Hence, a higher SSE indicates better performance.

**Figure 4:**
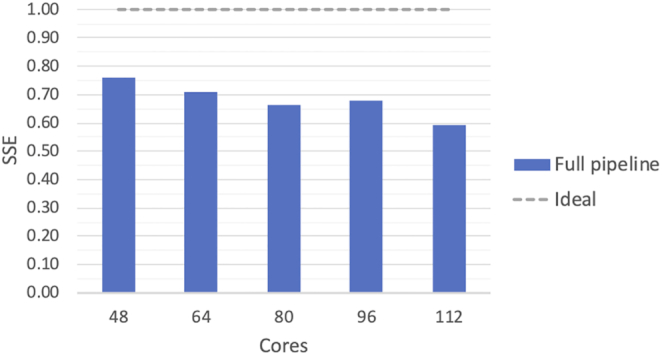
SSE for the SNP calling implemented in MaRe (Listing 3). The results are produced by using a full individual dataset from the 1KGP as input.

Fig. 4 shows the SSE for the full analysis. The SSE starts at 0.76 for 48 cores and decreases to 0.59 when running on 112 cores. Even if this does not show optimal performance, as in the VS use case, it still indicates good scalability. Indeed, the full benchmark ran in 3 hours and 24 minutes using MaRe on 112 cores, while it took 28 hours and 14 minutes using the built-in, single-node parallelization—leading to a speedup of 8.3.

**Figure 5:**
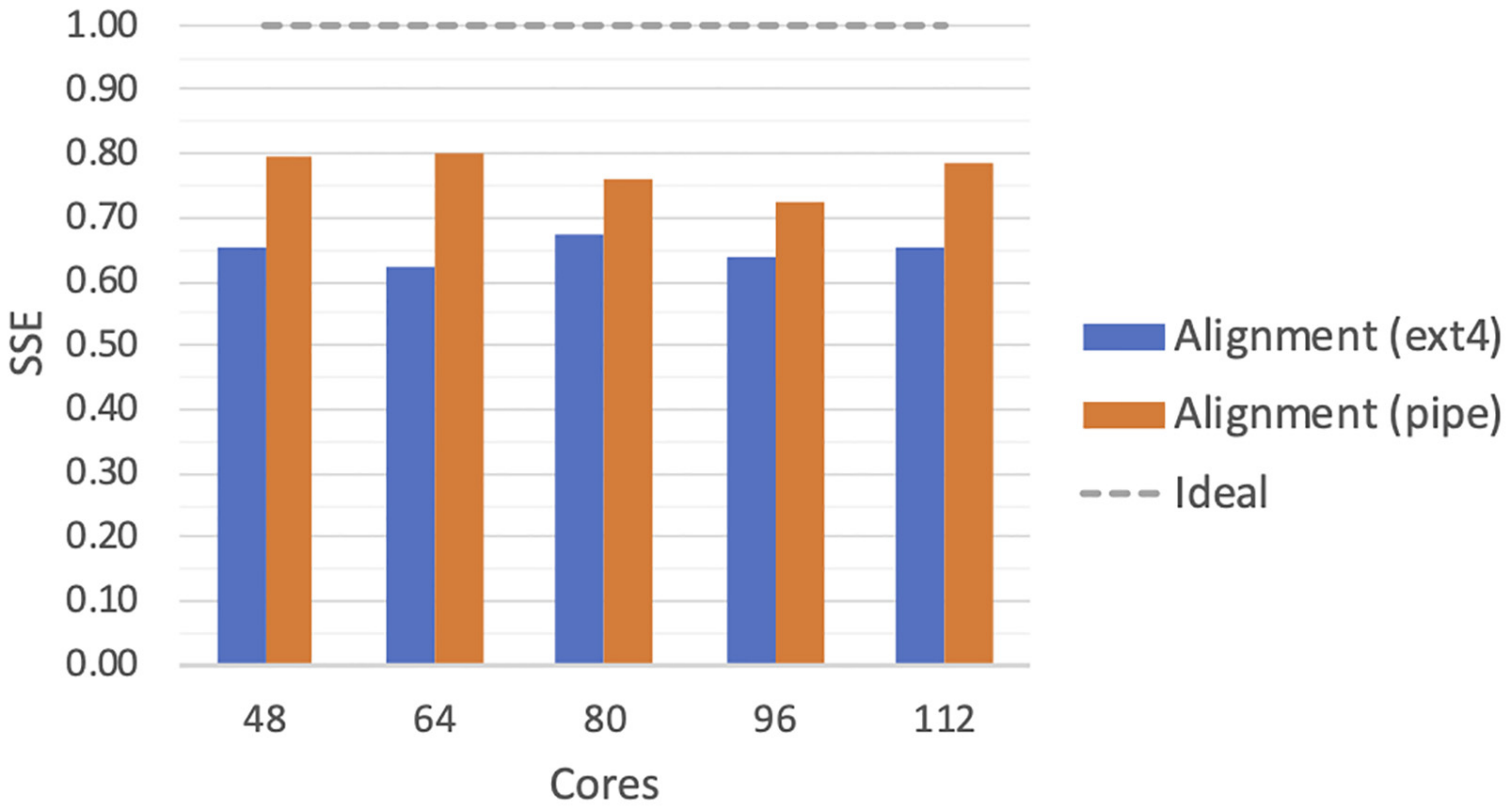
SSE for the SNP calling alignment stage implemented in MaRe (Listing 3, lines 1–13). The results are produced by using a full individual dataset from the 1KGP as input, and we show the SSE when using an SSD-based, ext4 temporary mount point as well as Unix pipes for passing the data to the containers.

The alignment portion of the benchmark uses BWA, which allows the reads to be input using pipes. It is interesting to compare how the SSE differs when using this input method as opposed to materializing the data on a temporary ext4 file space. Even though the standard RDD API provides a pipe method to do so, as we mentioned previously, this built-in implementation runs the external tool for each RDD record—which would result in considerable overhead. Instead, we compare the SSE achieved by MaRe with a pipePartition method, available in our benchmark repository [39], which pipes entire RDD partitions though a single dockerized tool instance. Fig. 5 shows the results of this comparison. Using pipes improved the SSE by ∼0.15 when running on 48 and 64 cores, by ∼0.08 when running on 80 and 96 cores, and by ∼0.12 when running on 112 cores. However, this improvement accounted for saving 6 minutes when running on 112 cores, which is negligible because the full analysis (including variant calling) took >3 hours to complete in such a setting.

### Discussion and conclusions

Big Data applications are getting increasing momentum in life science. Nowadays data are stored and processed in distributed systems, often in a geographically dispersed manner. This introduces a layer of complexity that MapReduce frameworks, such as Apache Spark, excel at handling [59]. Container engines, and in particular Docker, are also becoming an essential part of bioinformatics pipelines because they improve delivery, interoperability, and reproducibility of scientific analyses. By enabling application containers in MapReduce, MaRe constitutes an important advance in the scientific data-processing software ecosystem. When compared to current best practices in bioinformatics, relying solely on using workflow systems to orchestrate data pipelines, MaRe has the advantage of providing locality-aware scheduling, transparent ingestion from heterogeneous storage systems, and interactivity. As data become larger and more globally distributed, we envision scientists instantiating MaRe close to the data, and performing interactive analyses via cloud-oriented resources. In addition to the interactive mode, MaRe also support batch-oriented processing. This is important because it enables integration with existing bioinformatics pipelines. In practical terms, a packaged MaRe application can be launched by a workflow engine to enable data-intensive phases in a pipeline, and submitted to any of the resource managers supported by the Apache Spark community (including HPC systems [60]).

In the Evaluation section we show how researchers can easily implement 2 widely used applications in life science using MaRe. Both analyses can be coded in <50 lines of code, and they are seamlessly parallelized. The results show near optimal scalability for the VS application, with tmpfs improving performance over ext4 only by a negligible factor. The reason why there is no relevant performance improvement in using the former is that the container's running time dominates the time for materializing data on the temporary file space. Even though this may vary in other applications, in our experience this will often be the case for bioinformatics analyses, not justifying the additional effort in setting up a tmpfs space.

Scalability in the SNP-calling analysis is reasonably good but far from optimal. The reason for this is that before running the haplotype caller, a reasonable amount of data need to be shuffled across the nodes because GATK needs to see all of the data for a single chromosome at once in order to function properly, thus causing a large amount of data to be materialized on disk. Such overhead can be partly mitigated by enabling data streams via standard input and output between MaRe and containers, as the results in Fig. 5 show. This constitutes an area for future improvement; however, because GATK is unable to read data from the standard input, such improvement would not be directly applicable to the presented use case.

ADAM [61], a genomics data-processing framework built on top of Apache Spark, shows ideal scalability for a few, commonly used preprocessing steps in genomics pipelines—such as the SNP pipeline that we show in this article. Nevertheless, in real-world scenarios external software would still need to be used to compose end-to-end workflows. Indeed, ADAM itself provides a utility to integrate external tools into its pipelines [62]. Because this utility is based on pipes and it does not support application containers natively, it provides less flexibility in comparison with MaRe. Indeed, because MaRe is fully interoperable with Apache Spark, our recommendation for running genomics pipelines would be to use ADAM for the supported preprocessing steps and then MaRe to integrate external tools in the workflow.

The benchmarks that we show in this article are representative of 2 classes of problems where the application of MaRe could lead to different results in terms of performance. Materializing data is necessary to support any containerized tool, but our results show that this edges performance when records in large partitions need to be processed all together. In this case, reimplementing the analyses natively in Spark using the language of choice could lead to better performance; ADAM is a good example of this approach. It is however important to point out that the effort of reimplementing existing bioinformatics tools is seldom sustainable by research organizations. To give the reader an idea of this, ADAM is the product of a large collaboration maintaining thousands of lines of code. Owing to the current proliferation and heterogeneity of bioinformatics tools [63, 64], it is hard to imagine that such effort would generally be sustainable for many other applications. To this extent, MaRe stands out because it enables bioinformaticians to develop interoperable, distributed pipelines that scale reasonably well without the need to rewrite the existing codebase.

In conclusion, MaRe provides a MapReduce-oriented model to enable container-based bioinformatics analyses at scale. The project is available on GitHub [65] under an open source license, along with all of the code to reproduce the analyses in the Evaluation section [39].

## Methods

### Apache Spark

Apache Spark is an open source cluster-computing framework for the analysis of large-scale datasets [66]. The project originally started with the aim of overcoming the lack of in-memory processing in traditional MapReduce frameworks. Today, Apache Spark has evolved into a unified analytics engine, encompassing high-level APIs for machine learning, streaming, graph processing, and SQL, and it has become the largest open source project in Big Data analytics, with >1,000 contributors and >1,000 adopting organizations [25].

#### Clustering model

The Apache Spark clustering model includes a driver program, 1 or more worker nodes, and a cluster manager. The driver program is written by the user and controls the flow of the programmed analysis. For interactive analysis the driver program can run in notebooks environments such as Jupyter [31] and Apache Zeppelin [32]. Worker nodes communicate with the driver program, thus executing the distributed analysis as defined by the user. Finally, a cluster manager handles resources in the cluster, allowing for the executing processes to acquire them in the worker nodes. Apache Spark is cluster-manager agnostic and it can run in stand-alone settings, as well as on some popular platforms (e.g., Kubernetes [67], Mesos [68], and Hadoop YARN [69]).

#### Resilient distributed datasets

RDDs [33] are central to the Apache Spark programming model. RDDs are an abstraction of a dataset that is partitioned across the worker nodes. Hence, partitions can be operated in parallel in a scalable and fault-tolerant manner, and possibly cached in memory for recurrent access. As a unified processing engine, Apache Spark offers support for ingesting RDDs from numerous Big-Data–oriented storage systems. RDDs can be operated through Scala [30], Python [70], Java [71], and R [72] APIs. Such APIs expose RDDs as object collections, and they offer high-level methods to transform the datasets.

The mapPartition and repartition methods, from the RDD API, are useful to understand the MaRe implementation. The mapPartition method is inspired by functional programming languages. It takes as an argument a lambda expression that codes a data transformation, and it applies it to each partition, returning a new RDD. The repartition method, as the name suggests, changes the way the dataset records are partitioned across the worker nodes. It can be used to increase and decrease the number of partitions, thus affecting the level of parallelism, and it can also sort records in partitions, according to custom logics. In this case, an additional RDD method, namely, keyBy, needs to be used to compute a key for each RDD record. Similarly to mapPartition, keyBy applies a user-provided lambda expression to compute the record keys. Such keys are then used by repartition in conjunction with an extension of the Partitioner class [34] to assign records to partitions. For instance, when using HashPartitioner [73] records with same key always end up in the same RDD partition.

#### Stages and data locality

RDD methods are lazily applied to the underlying dataset. This means that until something needs to be written to a storage system or returned to the driver program, nothing is computed. In this way, Apache Spark can build a direct acyclic graph and thus optimize the physical execution plan. A physical execution plan is composed of processing tasks that are organized in stages. Typically, inside each stage the physical execution plan preserves data locality, while between stages a data shuffle occurs. In particular, a sequence of mapPartition methods generate a single stage, giving place to almost no communication in the physical execution plan. In contrast, each time repartition is applied to an RDD, a new stage is generated (and data shuffling occurs).

### Docker

Docker has emerged as the de facto standard application container engine [27]. Like virtual machines (VMs), application containers enable the encapsulation of software components so that any compliant computer system can execute them with no additional dependencies [18]. The advantage of Docker and similar container engines over virtualization consists of eliminating the need to run an operating system (OS) for each isolated environment. In contrast to hypervisors, container engines leverage kernel namespaces to isolate software environments, and thus run containers straight on the host OS. This makes application containers considerably lighter than VMs, enabling a more granular compartmentalization of software components.

#### Software Delivery

By enabling the encapsulation of entire software stacks, container engines have the potential to considerably simplify application delivery. Engines such as LXC [74] and Jails [75] have been available for almost 2 decades. Nevertheless, when compared to Docker these systems are poor in terms of software delivery functionalities. This is the reason why software containers' popularity exploded only when Docker emerged.

Docker containers can be defined using a text specification language. Using such language, users compose a Dockerfile that is parsed by Docker and then compiled into a Docker image. Docker images can then be released to public or private registries, becoming immediately available over the Internet. Therefore, by running the Docker engine, the end users can conveniently start the released containers locally.

#### Volumes

When using Docker containers for data processing, volumes play an important role. Indeed, there is a need for a mechanism to pass the input data to the containers and to retrieve the processed output from the isolated environment. Docker volumes allow for defining shared file spaces between containers and the host OS. Such volumes can be easily created when starting containers, by specifying a mapping between host OS file, or directories, and container mount points. Inside the containers these shared objects simply appear as regular files, or directories, under the specified mount point.

## Availability of Supporting Source Code and Requirements

Project name: MaRe

Project home page: https://github.com/mcapuccini/MaRe

Operating system(s): Platform independent

Programming language: Scala

Other requirements: Apache Spark and Docker

License: Apache License 2.0

RRID:SCR_018069

## Availability of Supporting Data and Materials

The dataset supporting the VS evaluation in this article is available in the ZINC database [47]. The specific subset that we used is available at http://zinc12.docking.org/catalogs/surechembl.

The 1KPG [50] dataset supporting the SNP evaluation is available on Amazon S3 (s3://1000genomes/phase3/data/HG02666). The relative BioProject accession number is PRJNA28889.

Images, results in tabular format, and an archival copy of the code are also available via GigaDB [76].

## Abbreviations

1KGP: 1000 Genome Project; API: application programming interface; bp: base pairs; BWA: Burrows-Wheeler Aligner; CSC: Information Technology Center for Science; GATK: Genome Analysis Toolkit; GC: guanine-cytosine; HDFS: Hadoop distributed file system; HIV: human immunodeficiency virus; HPC: high-performance computing; OS: operating system; POSIX: Portable Operating System Interface; RDD: resilient distributed dataset; SDF: structure-data file; SNP: single-nucleotide polymorphism; SSD: solid state drive; SSE: strong scaling efficiency; VCF: variant call format; VM: virtual machine; VS: virtual screening; WSE: weak scaling efficiency.

## Ethics Approval and Consent to Participate

All of the 1KGP data are consented for analysis, publication, and distribution. Ethics and consents are extensively explained in the 1KGP publications [50].

## Competing Interests

The authors declare that they have no competing interests.

## Funding

This research was supported by The European Commission’s Horizon 2020 program under grant agreement No. 654241 (PhenoMeNal).

## Authors' Contributions

M.C. and O.S. conceived the project. M.C. designed and implemented MaRe. M.C. and M.D. carried out the evaluation experiments. M.D. provided expertise in genomics. S.T. provided expertise in cloud computing. All authors read and approved the final manuscript.

## Supplementary Material

giaa042_GIGA-D-19-00170_Original_Submission

giaa042_GIGA-D-19-00170_Revision_1

giaa042_GIGA-D-19-00170_Revision_2

giaa042_GIGA-D-19-00170_Revision_3

giaa042_Response_to_Reviewer_Comments_Original_Submission

giaa042_Response_to_Reviewer_Comments_Revision_1

giaa042_Response_to_Reviewer_Comments_Revision_2

giaa042_Reviewer_1_Report_Original_Submission Umberto Ferraro Petrillo -- 6/14/2019 Reviewed

giaa042_Reviewer_1_Report_Revision_1 Umberto Ferraro Petrillo -- 2/19/2020 Reviewed

giaa042_Reviewer_2_Report_Original_Submission Andrew Lonie -- 6/24/2019 Reviewed
