# Supplementary material for: MaRe: Processing Big Data with application containers on Apache Spark
Source: Gigascience. 2020 May 5;9(5):giaa042. doi: 10.1093/gigascience/giaa042 (PMC7199472; doi:10.1093/gigascience/giaa042)
Supplement: giaa042_GIGA-D-19-00170_Revision_1 [file giaa042_giga-d-19-00170_revision_1.pdf]

GigaScience

MaRe: Processing Big Data with Application Containers on Apache Spark

--Manuscript Draft--

**Manuscript Number:**	GIGA-D-19-00170R1
**Full Title:**	MaRe: Processing Big Data with Application Containers on Apache Spark
**Article Type:**	Technical Note
**Funding Information:**	The European Commission’s Horizon 2020 programme (654241) — Dr. Ola Spjuth
**Abstract:**	Background. Life science is increasingly driven by Big Data analytics, and the MapReduce programming model has been proven successful for data-intensive analyses. However, current MapReduce frameworks offer poor support for reusing existing processing tools in bioinformatics pipelines. Further, these frameworks do not have native support for application containers, which are becoming popular in scientific data processing. Results. Here we present MaRe, an open-source programming library which introduces support for Docker containers in Apache Spark. Apache Spark and Docker are the MapReduce framework and container engine that have collected the largest open source community, thus MaRe provides interoperability with the cutting-edge software ecosystem. We demonstrate MaRe on two data-intensive applications in life science, showing ease of use and scalability. Conclusions. MaRe enables scalable data-intensive processing in life science with Apache Spark and application containers. When compared with current best practices, that involve the use of workflow systems, MaRe has the advantage of providing data locality, ingestion from heterogeneous storage systems and interactive processing. MaRe is generally-applicable and available as open source software.
**Corresponding Author:**	Marco Capuccini<br>Uppsala Universitet<br>Uppsala, SWEDEN
**Corresponding Author Secondary Information:**	
**Corresponding Author's Institution:**	Uppsala Universitet
**Corresponding Author's Secondary Institution:**	
**First Author:**	Marco Capuccini
**First Author Secondary Information:**	
**Order of Authors:**	Marco Capuccini
	Martin Dahlö
	Salman Toor
	Ola Spjuth
**Order of Authors Secondary Information:**	
**Response to Reviewers:**	Dear Editor,<br><br>Please find attached our revised manuscript "MaRe: Processing Big Data with Application Containers on Apache Spark" (GIGA-D-19-00170) submitted for publication in GigaScience.<br><br>We thank the reviewers for constructive criticism and in particular for suggesting to improve the evaluation section of our manuscript. We have now run the requested comparative experimentation where possible and included the results in the revised version. We believe that the scope and usefulness of the framework are now properly identified and discussed. In addition, we improved the sections that the reviewers found unclear.

Powered by Editorial Manager® and ProduXion Manager® from Aries Systems Corporation

We also have registered MaRe on SciCrunch.org and added its resource ID to the manuscript. Further, we have now specified the full URL to the 1000 genome project subset that we have utilized in the analysis.

Below we provide responses and comments to the reviewer's remarks and describe the updates we have made in the revised version of our manuscript. We hope that you now find it suitable for publication.

Sincerely,

Marco Capuccini and co-authors

Additional changes triggered by both reviewers comments
Even if not asked by the reviewers we did some minor changes to the evaluation section, which were triggered by the comparative experimentation.

First, the scaling efficiency measures in the first version of the manuscript were relative, meaning that we were computing the measures using the MaRe parallelization on a single node. When doing a comparative experimentation we instead need an absolute baseline to compute the measures, hence the scaling efficiency for both of the two use cases is now computed using the dockerized tools, built-in parallelization on a single node. We updated the evaluation section accordingly.

Second, when running the comparative experiments we were initially getting inconsistent results as the worker node flavors that we were previously using allowed overcommitted CPUs. This means that when increasing the number of nodes we did not always get the real parallelism that we were expecting. Changing the worker node flavor to one that does not allow overcommitment solved the problem. We updated the evaluation section describing the specifications of such flavor. Please note that we use slightly different node flavors in the two use cases now. In particular for the genomics use case we use a flavor with local SSD drive, which allows for materializing larger partitions faster on disk. As this kind of machines are scarce in our cloud provider, we scaled the analysis only up to 112 cores in the new version of the manuscript.

Finally, we switched the scaling efficiency metric for the genomics use case to Strong Scaling Efficiency (SSE). In the previous version of the manuscript the Weak Scaling Efficiency (WSE) was calculated without downsampling the input reference genome, thus giving a poor estimate of the metric. As there is no straightforward way of downsampling the reference genome without altering the behaviour of the tools, we kept the input data size fixed when increasing the parallelism in the new version of the study; thus computing SSE instead of WSE. We updated the evaluation section of the paper accordingly.

Reviewer #1
> I found the thesis of the paper to be interesting but a bit confusing. The title of the paper says that MaRe is a MapReduce oriented framework for processing Big Data. Then, it is said during the abstract that MaRe is a (new?) programming model. However, further discussion reveals that the programming model proposed by MaRe is essentially (a subset of) the same of Spark, with the only major difference being the ability to interact with external programs in a more seamless way than using the primitives coming with Spark.

> To this end, I think the authors should better clarify their contribution and, probably, put it in the right perspective. Namely, MaRe would be better presented as a software library acting as a wrapper for a Spark RDD and aiming to simplify the integration with external programs.

Thanks for pointing this out. Our initial reasoning was to see MaRe as a new programming model on top of Spark, but we agree that this can be confusing. To make our contribution more clear we updated the title of our manuscript, the abstract and the summary points in the last paragraph of the introduction.

> I also think the authors missed one important point while developing their work. Ok, I

Powered by Editorial Manager® and ProduXion Manager® from Aries Systems Corporation

am aware of the best practices encouraging the reuse of existing tools, but I would like to know if, using MaRe, I have to suffer from, let's say, a 10x slowdown with respect to a native implementation. Or, I would like to know what is the speedup achievable with respect to the usage of the standard facilities coming with Spark for running external processes. Instead, there is no evaluation of these cases. I think there are at least two solutions alternative to MaRe that should be considered in a comparative experimentation:

> - the transformation to apply to a certain dataset is not delegated to an external tool, but natively implemented in Spark using the language of choice

We understand your point. However, if some native Spark implementations of the tools used in the presented benchmarks were available there would be no need to reimplement them using MaRe. We could consider such implementations as existing tools and definitely encourage using them instead of our programming library. To the best of our knowledge, the only available Spark-based implementation of virtual screening (use case 1) was presented in our previous papers [1,2] and the only Spark-based implementation of genomics pipelines (use case 2) that has reached production readiness is ADAM [3]. Made an exception for a few preprocessing steps in ADAM, both of these existing implementations delegate data processing to external tools using pipes; the data is not solely processed using the language of choice.

Reusing existing tools is often the case in bioinformatics data processing as the effort of reimplementing single-node tools is seldom sustainable. Convincing arguments are the bioinformatics data pipelines available in repositories such as nf-core [4]. Also, another interesting supporting fact is that the development of the Spark-native tools in the GATK suite started in 2016 (this can be checked on GitHub https://github.com/broadinstitute/gatk) and even if backed by the Broad Institute still failed to produce a stable release; besides we, despite quite a lot of effort, couldn’t get the current beta to run on our cluster without errors. This clearly shows how much effort needs to be put in reimplementing such tools natively in Spark.

We expanded the first paragraph of the evaluation section to make our argument clear in the manuscript. Also, we now state clearly in the second last paragraph of the “discussion and conclusions” section that ADAM still relies on external tools to run real-world use cases.

> - the transformation is run through an external program by using the 'pipe' facility available with Spark.

Thanks for suggesting this comparison. Testing directly against RDD pipe would not make a fair comparison because such a method starts an instance of the external tool for each RDD record, thus introducing a considerable overhead. Please notice that MaRe feeds entire RDD partitions to the containers, hence generating way less tool startup overhead. However, similarly to what it was done in ADAM [3] and in our previous virtual screening implementation [1,2], for the revised manuscript we implemented a pipePartition method that pipes entire RDD partitions to the external tools and ran the comparison against it, where allowed by the external tools. In the added benchmarks, the only tool that allows for inputting the data via standard input is BWA, so this comparison was possible only for the alignment portion of the second benchmark. Please refer to figure 5, and its referencing paragraph in the updated evaluation section to see the results of such comparison.

> As an alternative, if the target application does not support the possibility of taking its input from the stdin, the input data is preliminarely saved in a file (e.g., using /tmpfs as MaRe does) and, then, it is used to run the external program.

As the reviewer acknowledges, copying/saving data on a preliminary file is exactly what MaRe does, so there would be no difference in performance when doing it manually in Spark. However, this would take many lines of codes, especially when implementing the reduce method, while MaRe makes it seamless. We believe this to be already clear in the implementation section of the manuscript.

> Along this line, another point that would have required a better investigation is the

Powered by Editorial Manager® and ProduXion Manager® from Aries Systems Corporation

choice of the solution to be used for storing temporary data to be processed by an external program. To this end, the solution chosen by the authors is to temporarily store data in memory using /tmpfs. I may be wrong, but this should mean that, at some point during the execution of an external process, the overall amount of available memory is decreased because input and/or output data is represented twice. This may have important consequences in processes where there is a high degree of parallelism and the amount of memory for executor is limited.

Thanks for pointing this out. It is true that by materializing the data on tmpfs we need twice as much memory for representing the partitions. However, please notice that Spark does not load partitions all at once, but it does it sequentially as resources become available. Since the partition size is configurable in Spark, one can tune it so that the total required memory will not exceed the available resources. Also notice that the partition size in Spark is equal to the block size in HDFS (128MB). This means that for a 8-cores machine a user would need 2GB of memory for representing the partitions twice. This is in most cases acceptable in modern data centers.

Representing data twice becomes a problem only when the user needs to aggregate large amounts of data on a single partition. This is necessary in our second benchmark, as GATK requires to see entire chromosomes at once. In such case a disk mount can be used instead of tmpfs; in our updated benchmark we used a local SSD drive.

We expanded the “data handling” section of the paper to make these points more clear.

> Conversely, the choice of storing this data on a persistent storage rather than in memory would be able to overcome this problem but would severely affect the performance of a process. These issues are briefly mentioned in the 'Discussion and conclusions section', while they would have required a much deeper investigation.

Thanks for pointing this out. An experimental comparison between tmpfs and persistent storage is possible for the virtual screening use case; please recall that for the other use case the intermediate partitions are too large to fit tmpfs. Please refer to figure 1 and its referencing text for the results of such comparison.

Surprisingly, there is very little overhead introduced by writing temporary data on the persistent disk; we used a regular block storage served over the network instead of SSD to evaluate this in the most penalizing settings. The reason why little overhead is introduced is that the partitions can be copied to the persistent disk relatively fast before the docker containers are started (recall that MaRe runs the tools for entire partitions and not record-wise). Then, since the container running time dominates over the data copying time, the total cost in terms of total running time is roughly the same. We update the second paragraph of the “discussion and conclusion” section accordingly.

> There are also some typos spread across the paper, such as:
> - Section 'Findings'.'Background and Purposes', page 2 : 'Finally by supporting Docker,' should be 'Finally, by supporting Docker'

Fixed.

> - Section 'Mare'.'Implementation', page 3: 'within each partitions' should be 'within each partition'

Fixed.

> - Section 'Evaluation', page 4 : 'Amanzon' should be 'Amazon'

Fixed.

> Finally, I think that the authors should put less emphasis on the possibility to ingest data from heterogeneous cloud resources as it is essentially inherited for free from Spark.

Powered by Editorial Manager® and ProduXion Manager® from Aries Systems Corporation

This is a good point. We removed this from the last paragraph of the introduction and we left out the benchmarks against multiple cloud storages.

Reviewer #2

> This first half of this work describes MaRe, a useful addition to the toolbox for scaling genomics analysis: a relatively simple approach to distributing container-based data-intensive analysis, based on MapReduce. The authors implement the framework in a sensible fashion, taking advantage of the various benefits of Apache Spark. The framework seems reasonable and useful.

> I am not convinced of the second part of the paper, which looks to evaluate MaRe using two real world applications. Admittedly it is not trivial to implement a distributive framework for generic applications that scales well, but that is sort of the point of the paper. Some specific concerns are around showing the the approach works for what is essentially a trivial distribution problem - where the data per job is small and jobs are relatively transactional and independent - but the major point of a general framework is that it is useful for more complex tasks, which the second variant calling example is.

Thanks for raising this point. Implementing a distributed framework for scaling any kind of application is out of the scope of this paper. Here, we aim at providing an alternative to workflow systems, which are broadly used in bioinformatics, that builds on top of the Apache Spark ecosystem. While scaling independent tasks is admittedly trivial, integrating application containers in Spark, such that containerized bioinformatics pipelines can easily be expressed in a few lines of code and yet scale reasonably good is not a simple problem. This is the main achievement of the presented work.

We believe this to be already clear in the current status of the paper.

> I have the nagging feeling that the specifics of the evaluation task here were set to the advantage of the framework, and still the outcome was just OK. The problem, as always, is that the individual tasks are dependent on I/O, and as the authors identify, data distribution is the factor in this example that dominates the scalability.

Thanks for pointing this out. Our intention with the evaluation of our work was to show two use cases that are somewhat representative of two classes problems that one may encounter when distributing bioinformatics pipelines. The first use case matches perfectly the MapReduce approach implemented by MaRe, thus we are able to show a scaling efficiency that is close to ideal; not “just OK”. In the second use case we deliberately expose where MaRe falls short by setting up a scenario in which the MapReduce model is disadvantaged. In our perspective the fact that even for this kind of problem the analysis still scales “just OK” is a strength of our framework rather than a weakness.

We updated the first paragraph of the evaluation section to make this more clear.

> This excerpt from the discussions and conclusions is telling:
> "Scalability in the SNP calling analysis is reasonably good but far from optimal. The reason for this is that before running the haplotype caller, a reasonable amount of data needs to be shuffled across the nodes as GATK needs to see all of
the data for a single chromosome at once in order to function properly, thus causing a large amount of data to be materialized on disk. Such overhead can be partly mitigated by enabling data streams via standard input and output between MaRe and containers, which constitutes an area for future improvement."

> In summary I think this paper needs additional work on the evaluation to identify the scope of the usefulness of the framework.; and the evaluation section itself needs to be clearer. The authors state :"It is however important to point out that while ADAM is application specific, MaRe applies to a variety of use cases in bioinformatics and it stands out by enabling distributed SNP calling in less than 50 lines of code."
If that's the case, I think the paper needs to identify and discuss the performance that can be expected across different types of use cases, and why.

Please refer to the previous point. Our intention with the two use cases is to show two

classes of problems for which one can expect ideal or suboptimal performance. We updated the first paragraph of the evaluation section to make this more clear.

> As a suggestion, a comparison to ADAM leading to a discussion of what the fundamental challenges of scaling I/O intensive tasks are and how that might map to different common tasks in bioinformatics, would be useful.

Thanks for suggesting a comparison with ADAM. We realized that we did not state explicitly that ADAM implements only a few preprocessing steps of the SNP pipeline [3]. Near-ideal scalability is shown in [3] only for these preprocessing steps, however in real-world settings some external tools would be needed to run a complete analysis. Indeed, ADAM provides a modified version of RDD pipes for running external tools [5], but no study has yet quantified what kind of performance one can expect when running external tools in ADAM. One major problem with pipes is that not every external tool is capable of accepting data via standard input. GATK, which provides a state of the art variant caller, is an example of such a tool. For this reason we were not able to reproduce the same pipeline that we ran for our genomics benchmark using ADAM. However, the first part of the pipeline uses a tool that can read data via standard input (BWA). Hence, we could compare the scaling efficiency that we obtained using MaRe, for this first portion of the pipeline, with a similar implementation of the modified RDD pipe routine included in ADAM. We prefered to simply reimplement this routine as the ADAM project requires many dependencies that would be hard to bring in our environment. Figure 5, and its referencing text, present the results of this new comparison. We also added the discussed details about ADAM in the fourth paragraph of the “discussion and conclusion” section.

> Or at least a discussion of the characteristics of problems that MaRe would suit.

We expanded the second last paragraph of the “discussion and conclusion” section to point out where MaRe falls short. In summary, when records in large partitions need to be processed all together it is not reasonable to expect ideal scalability, however given the effort that sometimes need to be put in reimplementing bioinformatics tools in Spark, scaling the analyses in MaRe could prove to be more sustainable.

> There are also a handful of expression and grammatical errors:

>> "Such amounts of data poses major challenges for scientific analyses"

> such amounts of data _pose_ major challenges…

Fixed.

> "but also prohibitively expensive in terms of power consumption, estimated to be in the order of several hundred thousand dollars per year"

>> is this for all life science data transfer over the entire planet? The sentence needs some qualification.

Referring to the cited work, this is for a single next-generation HPC cluster. We added this detail to the sentence to make it more clear.

> "In summary, the key contribution of the presented work are:"

> In summary, the key contributions of the presented work are:

Fixed.

> "We demonstrate data ingestion from three large-scale storage systems: Hadoop Distributed File System (HDFS) [41], Swift [42] and Amanzon S3 [43]. In our settings HDFS was co-located with the Apache Spark"

>> Amazon S3

Fixed.

Powered by Editorial Manager® and ProduXion Manager® from Aries Systems Corporation

	> "One of the advantages of Apache Spark over other MapReduce-like systems is the ability of retaining data in memory. Hence, for better performance it is preferable to keep RDD records in memory when mounting them in the containers."<br><br>> the word 'Hence' doesn't make logical sense in this sentence.<br><br>We removed the sentence starting with "Hence".<br><br>> "The full benchmark runs in ~1.8 hours when using 128 vCPUs, including data ingestion from S3."<br><br>>> It's impossible to know what this means as it is given in isolation - no context and no comparison to performance after. I think this is an example of a significant flaw in the paper, in that the theoretical and implementation parts seem fine, but the evaluation is not really convincing.<br><br>This is a good point. We added some details about the running time on a single node to add some context and comparison of the statement. Please refer to the second last paragraph of the "Virtual Screening" section and to the second last paragraph of the "Single Nucleotide Polymorphism" section.<br><br>*References*<br>1. Capuccini M, Ahmed L, Schaal W, Laure E, Spjuth O. Large-scale virtual screening on public cloud resources with Apache Spark. J Cheminform. 2017;9: 15.<br>2. Ahmed L, Georgiev V, Capuccini M, Toor S, Schaal W, Laure E, et al. Efficient iterative virtual screening with Apache Spark and conformal prediction. J Cheminform. 2018;10: 8.<br>3. Nothaft FA, Linderman M, Franklin MJ, Joseph AD, Patterson DA, Massie M, et al. Rethinking Data-Intensive Science Using Scalable Analytics Systems. Proceedings of the 2015 ACM SIGMOD International Conference on Management of Data - SIGMOD '15. 2015. doi:10.1145/2723372.2742787<br>4. Ewels P. nf-core. [cited 28 Jan 2020]. Available: https://nf-co.re/pipelines<br>5. Using ADAM's Pipe API — bdgenomics.adam 0.23.0-SNAPSHOT documentation. [cited 29 Jan 2020]. Available: https://adam.readthedocs.io/en/latest/api/pipes/
**Additional Information:**	
**Question**	**Response**
Are you submitting this manuscript to a special series or article collection?	No
**Experimental design and statistics**<br><br>Full details of the experimental design and statistical methods used should be given in the Methods section, as detailed in our Minimum Standards Reporting Checklist. Information essential to interpreting the data presented should be made available in the figure legends.<br><br>Have you included all the information requested in your manuscript?	Yes
**Resources**	No

Powered by Editorial Manager® and ProduXion Manager® from Aries Systems Corporation

A description of all resources used, including antibodies, cell lines, animals and software tools, with enough information to allow them to be uniquely identified, should be included in the Methods section. Authors are strongly encouraged to cite Research Resource Identifiers (RRIDs) for antibodies, model organisms and tools, where possible.<br><br>Have you included the information requested as detailed in our Minimum Standards Reporting Checklist?	
If not, please give reasons for any omissions below.<br><br>as follow-up to "**Resources**<br><br>A description of all resources used, including antibodies, cell lines, animals and software tools, with enough information to allow them to be uniquely identified, should be included in the Methods section. Authors are strongly encouraged to cite Research Resource Identifiers (RRIDs) for antibodies, model organisms and tools, where possible.<br><br>Have you included the information requested as detailed in our Minimum Standards Reporting Checklist?<br><br>"	In this work we use public data from the 1000 genome project and from the ZINC molecular database. Relevant details are explained in the referenced publications.
**Availability of data and materials**<br><br>All datasets and code on which the conclusions of the paper rely must be either included in your submission or deposited in publicly available repositories (where available and ethically appropriate), referencing such data using a unique identifier in the references and in the "Availability of Data and Materials" section of your manuscript.	Yes

Powered by Editorial Manager® and ProduXion Manager® from Aries Systems Corporation

Have you have met the above requirement as detailed in our Minimum Standards Reporting Checklist?	

Powered by Editorial Manager® and ProduXion Manager® from Aries Systems Corporation

Manuscript (changes highlighted) Click here to access/download;Manuscript;manuscript-diff.pdf

OXFORD (GIGA)n SCIENCE

GigaScience, 2019, 1–13

doi: xx.xxxx/xxxx
Manuscript in Preparation
Technical Note

TECHNICAL NOTE

MaRe: Processing Big Data with Application Containers on Apache Spark

Marco Capuccini[1,2,*], Martin Dahlö[2,3,4], Salman Toor[1] and Ola Spjuth[2]

[1]Department of Information Technology, Uppsala University, Sweden and [2]Department of Pharmaceutical Biosciences, Uppsala University, Sweden and [3]Science for Life Laboratory, Uppsala University, Sweden and [4]Uppsala Multidisciplinary Center for Advanced Computational Science, Uppsala University, Sweden

[*] marco.capuccini@it.uu.se

Abstract

Background. Life science is increasingly driven by Big Data analytics, and the MapReduce programming model has been proven successful for data-intensive analyses. However, current MapReduce frameworks offer poor support for reusing existing processing tools in bioinformatics pipelines. Further, these frameworks do not have native support for application containers, which are becoming popular in scientific data processing.
Results. Here we present MaRe, ~~a programming model with an associated~~ an open-source ~~implementation,~~ programming library which introduces support for ~~application containers in MapReduce. MaRe is based on~~ Docker containers in Apache Spark. Apache Spark and Docker ~~,~~ are the MapReduce framework and container engine that have collected the largest open source community, thus ~~providing~~ MaRe provides interoperability with the cutting-edge software ecosystem. We demonstrate MaRe on two data-intensive applications in life science, showing ease of use and scalability.
Conclusions. MaRe enables scalable data-intensive processing in life science with ~~MapReduce~~ Apache Spark and application containers. When compared with current best practices, that involve the use of workflow systems, MaRe has the advantage of providing data locality, ingestion from heterogeneous storage systems and interactive processing. MaRe is generally-applicable and available as open source software.

Key words: MapReduce; application containers; Big Data; Apache Spark; workflows.

Findings

Background and purpose

Life science is increasingly driven by Big Data analytics. From genomics, proteomics and metabolomics to bioimaging and drug discovery, scientists need to analyze larger and larger amounts of data [1, 2, 3, 4, 5]. This means that datasets can no longer be stored and processed in a researcher's workstation, but they instead need to be handled on distributed systems, at organization level. For instance, the European Bioinformatics Institute, in Hinxton (United Kingdom), offers a total storage capacity of over 160 petabytes for biologically-significant data [6]. Such amounts of data pose major challenges for scientific analyses. First, there is a need to efficiently scale existing processing tools over massive datasets. In fact, bioinformatics software that was originally developed with the simplistic view of small-scale data, will not scale on distributed computing platforms out of the box. The process of adapting such tools may introduce disruptive changes to the existing codebase, and it is generally unsustainable for most organizations. Secondly, the complexity in programming distributed systems may be hard to cope with for most researchers, who instead need to focus on the biological problem at hand. In addition, as life science is exploratory, scientists increasingly demand being able to run interactive analyses rather than submitting jobs to batch systems. Thirdly, when handling Big Data in distributed systems, data locality is a major concern. Indeed, if once data could be shuffled with little regard, with massive datasets it is not only inefficient [7], but also prohibitively expensive in

Compiled on: February 10, 2020.
Draft manuscript prepared by the author.

terms of power consumption ~~,~~ – estimated to be in the order of several hundred thousand dollars per year for a single next-generation High-Performance Computing (HPC) cluster [8]. For geographically dispersed datasets, locality-awareness becomes even more challenging, as computing resources need to be dynamically acquired close to the data [9]. Cloud computing solves this problem by enabling the allocation of virtual infrastructure on demand [10]. However, heterogeneity in storage systems for cloud providers [11], makes it hard to abstract data ingestion from many different sources. Finally, as bioinformatics software is characterized by complex software dependencies, deploying and managing a vast collection of tools in a large distributed system also represents a major challenge [12].

Current bionformatics best practices make use of workflow systems, to orchestrate analyses over distributed computing platforms [13]. Workflow systems provide high-level Application Programming Interfaces (APIs) that allow for defining an execution graph of existing processing tools. At run time, the execution graph is used to pipeline the analysis on distributed cloud or ~~High-Performance Computing (HPC)~~ HPC resources. Hence, the parallelization of the analysis is transparently carried out, by executing non-dependent tasks at the same time. Cutting-edge workflow systems, such as Luigi [14], NextFlow [15], Galaxy [16] and Pachyderm [17] allow for running processing tools as application containers. This light-weight packaging technology allows for encapsulating complete software environments, so that distributed systems can run the processing tools with no need of additional dependencies, in an isolated manner [18]. Hence, container-enabled workflow systems provide a fairly easy way to define distributed analyses comprising existing bioinformatics tools, and eliminating the need for managing complex software delivery process and dependency management. Nevertheless, workflow-oriented processing falls short when it comes to Big Data analyses. To the best of the authors knowledge, all of these systems utilize a decoupled shared storage system, for synchronization and intermediate results storage. When dealing with large datasets, this translates to a massive and unnecessary communication in the underlying infrastructure. In addition, workflow systems usually support a limited amount of storage backends, not seldom only POSIX file systems, making it hard to ingest data from heterogeneous cloud resources. Finally, due to their batch-oriented nature, it is also intrinsically hard to enable interactive, exploratory analyses using workflow-oriented frameworks.

Google's MapReduce programming model and its associated implementation pioneered uncomplicated Big Data analytics on distributed computing platforms [19]. When using MapReduce, the analysis is defined in a high-level programming language that hides challenging parallel programming details including fault tolerance, data distribution and locality-aware scheduling. Open-source implementations of MapReduce are well established in industrial and scientific applications [20, 21], and numerous success stories in life science have been reported [22, 23, 24].

Apache Spark has emerged as the project that collected the largest community, in the open-source MapReduce ecosystems [25]. In addition to the MapReduce implementation, Apache Spark also provides increasingly important features, such as in-memory, interactive and stream processing. Furthermore, due to broad collaborations in the open-source community, Apache Spark supports all of the major storage systems, enabling data ingestion from heterogeneous cloud resources. These characteristics are particularly appealing for the case of Big Data in life science. Nevertheless, Apache Spark, and other similar frameworks, offer poor support for composing analyses out of existing processing tools. This is usually limited to calling external programs, which can only access data sequentially, without support for application containers [26]. In fact, the main way of implementing analytics in MapReduce-oriented environments is to code each transformation using one of the available APIs. This way of implementing analyses contrasts with current best practices in bioinformatics, that promote the usage of existing tools as application containers with the goal of improving delivery, interoperability and reproducibility of scientific pipelines [15].

Here we introduce MaRe: an open-source programming library that extends Apache Spark, introducing comprehensive support for external tools and application containers in MapReduce. Similarly to container-enabled workflow systems, MaRe allows to define analyses in a high-level language, in which data transformations are performed by application containers. In addition, MaRe provides seamless management of data locality as well as full interoperability, with the Apache Spark ecosystem. This last point allows MaRe analyses to ingest data from heterogeneous cloud storage systems, and also provides support interactive processing. Finally, by supporting Docker, the de facto standard container engine [27], MaRe is compatible with numerous existing container images.

In summary, the key contributions of the presented work are:

- We introduce ~~a~~ MaRe: an open-source MapReduce-oriented programming ~~model~~ library for container-based data processing on top of Apache Spark.
- ~~We provide MaRe: an open-source implementation of the introduced programming model.~~
- We benchmark MaRe on two data-intensive applications in life science, showing ease of use and scalability~~, also in relation to three large-scale storage systems~~.

MaRe

Programming Model

We introduce the MaRe programming model using a simple, yet interesting, example in genomics. A DNA sequence can be represented as a text file written in a language of 4 characters: A,T,G,C. The GC content in a DNA sequence has interesting biological implications; for instance there is evidence that GC-rich genes are expressed more efficiently than GC-poor genes [28]. Hence, within a large DNA sequence it can be interesting to count G and C occurrences. Given an Ubuntu Docker image [29], the task can easily be implemented in MaRe using POSIX tools. Listing 1 shows such implementation.

Listing 1. GC count in MaRe

```
val gcCount = new MaRe(genomeRDD).map(
  inputMountPoint = TextFile("/dna"),
  outputMountPoint = TextFile("/count"),
  imageName = "ubuntu",
  command = """
    grep -o '[GC]' /dna | wc -l > /count
  """
).reduce(
  inputMountPoint = TextFile("/counts"),
  outputMountPoint = TextFile("/sum"),
  imageName = "ubuntu",
  command = """
    awk '{s+=$1} END {print s}' /counts > /sum
  """
)
```

Being based on Apache Spark, MaRe has a similar programming model. The control flow of the analysis is coded in Scala [30], by the program in listing 1. Such program is called *driver*

in the Apache Spark terminology. The driver program can be packaged and submitted to a cluster (in batch mode), or executed interactively using a notebook environment such as Jupyter [31] or Apache Zeppelin [32]. Listing 1 starts by instantiating a `MaRe` object, which takes a Resilient Distributed Dataset (RDD) [33], containing the input genome file in text format. Such RDD can be easily loaded using the Apache Spark API from any of the supported storage backends. The `map` primitive (line 1 to 8) applies a command from the Docker image to each partition of the RDD. In our example we specify the Ubuntu image on line 4, and we use a command that combines `grep` and `wc` to filter and count GC occurrences (on line 6). The partition are mounted in the Docker containers in the configured input mount point (`"/dna"` at line 2), and the command results are loaded back to MaRe from the configured output mount point (`"/count"` on line 3). In the example we use *TextFile* mount points as the input data is in text format. By default, MaRe considers each line in a text file as a separate record, but custom record separators can also be configured using the *TextFile* constructor.

At this point it is important to mention that MaRe can also handle binary files. For such data formats, the driver program should specify mount points of type `BinaryFiles`. In this case, each RDD record is considered as a distinct binary file, thus the specified mount point results in a directory containing multiple files (as opposed to `TextFile` that mounts the records in a single file). We provide an example of the `BinaryFiles` mount point in the evaluation section.

Coming back to listing 1, after applying the `map` primitive, each RDD partition is transformed into a distinct GC count. The `reduce` primitive (line 8 to 15), aggregates the counts in each partition to a cumulative sum. Again, we use mount points of type `TextFile`, to mount the intermediate counts in the containers (`"/counts"` on line 9) and to read back the cumulative sum (`"/sum"` on line 10). The sum is computed using the `awk` command from the Ubuntu image (lines 11 to 14). Finally, the result is returned to the `gcCount` variable at line 1.

From the GC example, the reader may have noticed that our programming model is strongly inspired by MapReduce. In addition, Apache Spark users may have noticed that the GC count problem can easily be solved in pure Spark code. Indeed, the aim of the example is just to provide an easy introduction to MaRe, and two real-world applications are available in the evaluation section.

Apart from `map` and `reduce`, MaRe provides an additional primitive. For real-world applications, we noticed that it is often needed to group dataset records according to a specific logic before applying `map` or `reduce`. For this reason, MaRe also provides a `repartitonBy` primitive, which repartitions the RDD records according to a configurable grouping rule. More specifically, the `repartitonBy` primitive takes into account a user-provided `keyBy` function, which is used to compute a key for each record in the dataset. Then, the repartitioning is performed accordingly so that records with same key end up in the same partition. An example of `repartitonBy` is available in the evaluation section.

Implementation

MaRe comes as a thin layer on top of the RDD API [33], and it relies on Apache Spark to provide important features such as data locality, data ingestion, interactive processing, and fault tolerance. The implementation effort consists of: (i) leveraging the RDD API to implement the MaRe primitives and (ii) handling data between containers and RDD structures.

Primitives. Each instance of a `MaRe` object retains an underlying RDD, which represents an abstraction of a dataset that is partitioned across Apache Spark workers. The `map`, `reduce` and `repartitionBy` primitives utilize the underlying RDD API to operate such dataset.

Figure 1 shows the execution diagram for the `map` primitive. For simplicity, in figure 1 we show a single partition per worker, but in reality workers may retain multiple partitions. This primitive takes an input RDD that is partitioned over N nodes, and it transforms each partition using a Docker container command - thus returning a new RDD'. This logic is implemented using *mapPartitions* from the RDD API. When calling *mapPartitions*, MaRe specifies a lambda expression that: (i) makes the data available in the input mount point, (ii) runs the Docker container and (iii) retrieves the results from the output mount point. When using *mapPartitions*, Apache Spark generates a single stage, thus no data shuffle is performed.

Figure 2 shows the execution diagram for the `reduce` primitive. This primitive takes an input RDD, partitioned over N nodes, and it iteratively aggregates records, reducing the number of partition until an RDD', containing a single result partition, is returned. Again, the input RDD may retain multiple partitions per node. However, as opposed to the `map` primitive, RDD' always contains a single partition when it is returned. Given a user-configured depth K, the records in the RDD are aggregated using a tree-like algorithm. In each of the K levels in the tree, the records within each partitions are first aggregated using a Docker container command. Like the `map` primitive, this first transformation is implemented using *mapPartitions*, from the RDD API. Then, the number of partitions is decreased using *repartition* from the RDD API. This process is repeated K times until one single partition is left. At this point the records within the remaining partition are aggregated again using *mapPartitions* (from the RDD API), and RDD' is returned. A new stage is generated each time *repartition* is used. Hence, `reduce` leads to K data shuffles. For this reason, when aggregating records, the user-provided command should always reduce the size of the partition. In addition, for results consistency, the command should perform an associative and commutative operation. By default MaRe sets K to 2, however the user may chose a higher tree depth when it is not possible to sufficiently reduce the dataset size in one go.

Finally, the `repartitionBy` primitive is implemented by using *keyBy*, and then *repartition* from the RDD API. MaRe uses the user-provided grouping rule with *keyBy*, to compute a key for each RDD record, and then it applies `repartition` in counjuction with *HashPartitioner* [34], which makes sure that records with same key end up in the same partition.

Data Handling. One of the advantages of Apache Spark over other MapReduce-like systems is the ability of retaining data in memory. ~~Hence, for better performance it is preferable to keep RDD records in memory when mounting them in the containers.~~ To achieve this when passing the data to the application containers, there are a few options available: (i) Unix pipes [35], (ii) memory-mapped files [36] and (iii) *tmpfs* [37]. Solution (i) and (ii) are the most ~~performant~~ memory-efficient as they do not need to materialize the data when passing it to the containers. However, (i) allows to see records only once in a stream-like manner, while (ii) requires the container-wrapped tools to be able to read from a memory-mapped file. Apache Spark loads data in memory sequentially and partiton-wise. Partition size is configurable and often equals to the block size in the underlying storage system. For the Hadoop Distributed File System (HDFS) this value defaults to 128MB, meaning that on a 8-core machine materializing again partitions on an in-memory file system would require 2GB of memory in total – which is usually not a problem on modern data centers. Therefore, to support any wrapped-tool, we decided to start by implementing solution (iii). This means that MaRe uses an in-memory

tmpfs file system as temporary file space for the input and output mount points. The solution allows to provide a standard POSIX mount point to the containers, while still retaining reasonable performance [37]. However, MaRe also provides users with the option of selecting any other disk-based file system for the temporary mount points. Even if this ~~is not the best solution from a performanceperspective, it can still be useful for particularly large partitions that need to be processed at once~~could in principle edge performance, this can be useful when a dockerized tool does not allow for splitting large partitions in smaller chunks of records – we show an example of this in the evaluation section.

Evaluation

We evaluate MaRe on two data-intensive applications in life science. ~~More specifically~~ The first application can be decomposed to somewhat independent jobs, where the data assigned to each job can be relatively small. This is where MapReduce-oriented programming libraries such as MaRe excel. Conversely, the second application requires to compute larger chunks of data all at once, thus allowing us to show the performance penalty that is introduced in such case. More in detail we evaluate: (i) how the analyses can be implemented in MaRe and (ii) how the analyses scale over multiple nodes. To the best of our knowledge, no stable Spark-native implementation of the tools presented in the analyses is publicly available, making a fair performance comparison with a system that does not delegate data processing to an external application container unfeasible. To this extent, we would like to add that if such implementation were available there would be no advantage in rewriting the analyses using our programming library.

The scalability experiments were carried out on cPouta: an OpenStack-based cloud service operated by the Information Technology Center for Science (CSC) in Finland [38]. ~~On top of cPouta, we ran a stand-alone Apache Spark cluster composed of 1 master and 16 worker nodes. Each node provided 8 virtual Central Processing Units (vCPUs) and 32GB of memory, thus resulting in a total of 128 vCPUs and 512GB of memory.~~ The driver programs were run interactively using an Apache Zeppelin environment [32], and the notebooks were made available to sustain reproducibility [39]. In addition, we also made available a deployment automation that enables to replicate our setup on cPouta, as well as any other OpenStack-based cloud provider [40].

~~Since MaRe is conceived for data-intensive applications, the scalability is primarily evaluated in terms of Weak Scaling Efficiency (WSE). This performance metric shows how the system scale when increasing data and parallelism. To compute the WSEs we first ran the analyses on the full evaluation datasets using 16 worker nodes. Then, we ran again on 1/2, 1/4, 1/8 and 1/16 of the datasets, using 8, 4, 2 and 1 nodes respectively. The WSE is then computed as the time for processing the 1/16 of the data on 16 nodes, divided by the time for processing 1/N of the data using 16/N nodes (for N=1,2,4,8,16). The ideal case, when doubling the number of nodes, is to be able to process twice as much data in the same amount of time. Hence, a higher WSE indicates better performance.~~

~~We demonstrate data ingestion from three large-scale storage systems: Hadoop Distributed File System (HDFS)~~ [41] ~~, Swift [42] and Amazon S3~~ [43]. ~~In our settings HDFS was co-located with the Apache Spark cluster. This means that the HDFS daemons ran in the worker nodes, allowing for near-zero network communication. Swift is provided as a service by cPouta, thus being decoupled from our worker nodes. However, by setting up the cluster on cPouta, we ran the analyses close to Swift (thus enabling fast ingestion). Finally, S3 is provided by Amazon, hence in this case the analysis accessed data from a remote location. Even if this is not optimal from a performance perspective, it can sometimes be unfeasible to store large datasets locally and here we aim to show the tradeoff for this setting.~~

Virtual Screening

Virtual Screening (VS) is a computer-based method to identify potential drug candidates, by evaluating the binding affinity of virtual compounds against a biological target protein [44]. Given a 3D target structure, a molecular docking software is run against a large library of known molecular representations. For each compound in the virtual molecular library the docking software produces a pose, representing the orientation of the molecule in the target structure, and a binding affinity score. The poses with the highest affinity scores can be considered as potential drug leads for the target protein.

VS is data-intensive as molecular libraries usually contain millions of compounds. A simple, yet effective, approach to scale VS consists of: (i) distributing the molecular library over several nodes, (ii) running the docking software in parallel and (iii) aggregating the top-scoring poses. Listing 2 shows how this logic can be implemented in MaRe, using FRED [45] as molecular docking software, and sdsorter [46] to filter the top-scoring poses.

Listing 2. Virtual Screening in MaRe

```
1  val topPosesRDD = new MaRe(libraryRDD).map(
2    inputMountPoint = TextFile("/in.sdf", "\n$$$$\n"),
3    outputMountPoint = TextFile("/out.sdf", "\n$$$$\n"),
4    imageName = "mcapuccini/oe:latest",
5    command = """
6      fred -receptor /var/openeye/hiv1_protease.oeb \
7        -hitlist_size 0 \
8        -conftest none \
9        -dbase /in.sdf \
10       -docked_molecule_file /out.sdf
11   """
12 ).reduce(
13   inputMountPoint = TextFile("/in.sdf", "\n$$$$\n"),
14   outputMountPoint = TextFile("/out.sdf", "\n$$$$\n"),
15   imageName = "mcapuccini/sdsorter:latest",
16   command = """
17     sdsorter -reversesort="FRED Chemgauss4 score" \
18       -keep-tag="FRED Chemgauss4 score" \
19       -nbest=30 \
20       /in.sdf /out.sdf
21   """
22 )
```

In listing 2, we initialize MaRe by passing it a molecular library that was previously loaded as an RDD (`libraryRDD` on line 1). We implement the parallel molecular docking using the `map` primitive. On line 2 and 3, we set input and output mount points as text files, and assuming the library to be in Structure-Data File (SDF) format [47] we use the custom record separator: "`\n$$$$\n`". On line 4, we specify a Docker image containing FRED. The image is not publicly available as it also contains our FRED license, but the license can be obtained free of charge for research purposes and we provide a *Dockerfile* [39] to build the image. On line 5, we specify the FRED command. We use a HIV-1 protease receptor [48] as target (which is wrapped in the Docker image), and we set: (i) `-hitlist_size 0` to not filter the poses in this stage, (ii) `-conftest none` to consider the input molecules as single conformations, (iii) `-dbase /in.sdf` to read the input molecules from the input mount point and (iv)

-docked_molecule_file /out.sdf to write the poses to the output mount point.

The map phase produces a pose for each molecule in libraryRDD. On line 12, we use the reduce primitive to filter the top 30 poses. On line 13 and 14, we set the input and output mount points as we do for the map primitive. On line 15, we specify a publicly available Docker image containing sdsorter. On line 16, we specify the sdsorter command, and we set: (i) -reversesort="FRED Chemgauss4 score" to sort the poses from highest to lowest FRED score, (ii) -keep-tag="FRED Chemgauss4 score to keep the score in the results, (iii) -nbest=30 to output the top 30 poses and (iv) /in.sdf /out.sdf to read and write from the input mount point and to the output mount point respectively. Please notice that this command performs an associative and commutative operation, thus ensuring correctness in the reduce phase. Finally, the results are returned to topPosesRDD, on line 1.

We benchmarked the analysis coded in listing 2 against the SureChEMBL library [49] retrieved from the ZINC database [50], containing ~2.2M molecules. The ~~full benchmark runs in ~3 hours when using 128 vCPUs.~~ benchmark ran on top of a stand-alone Apache Spark cluster composed of 1 master and 12 worker nodes. Each node provided 10 cores and 43GB of memory, thus resulting in a total of 120 cores and 516GB of memory. The data was made available to the workers using a co-located HDFS storage. Under these settings, we evaluated the scalability in terms of Weak Scaling Efficiency (WSE). This performance metric shows how the system scale when increasing data and parallelism. To compute the WSEs we first ran the benchmark on 1/12 of the dataset using the dockerized tools on a worker node using their built-in, single-node parallelization. Then, we ran again the pipeline using MaRe on 2/12, 4/12, 6/12, . . . and 12/12 of the datasets, using 2, 4, 6, . . . and 12 worker nodes respectively. The WSE is then computed as the time for processing 1/12 of the data using the built-in, single-node parallelization, divided by the time for processing N/12 of the data using N nodes (for N=2,4,6,. . . ,12). The ideal case, when doubling the number of nodes, is to be able to process twice as much data in the same amount of time. Hence, a higher WSE indicates better performance.

Figure 3 shows the WSE for the full ~~analyisis~~analysis, when using ~~HDFS and Swift. The~~ *tmpfs* and a disk-based, *ext4* file system [51] as temporary mount points. From the experiments it emerges that there is little difference between the two methods in terms of scaling efficiency – *tmpfs* improved the WSE by 0.02 at most. Indeed, the results in figure 3 indicate very good scalability ~~, with WSE close and even exceeding~~ with a WSE close to ideal for both *tmpfs* and *ext4*. For 120 cores, the full benchmark ran in 2 hours and 21 minutes while 1~~, up to 64 vCPUs. For 128 vCPUs the WSE levels off slightly at ~0.9, still indicating good scalability.~~ /12 of the input data was processed by the the built-in, single-node parallelization in 2 hours and 14 minutes – resulting in 0.94 WSE. This means that the overhead introduced by MaRe accounts for only 7 minutes in total.

Finally, to ensure the correctness of the parallelization, we ran sdsorter and FRED on a single core against 1K molecules that we randomly sampled from SureChEMBL, and we compared the results with those produced by the code in listing 2.

Single Nucleotide Polymorphism Calling

A Single Nucleotide Polymorphism (SNP) is a position in a DNA sequence where a single nucleotide (or base pair) is different when compared to another DNA sequence [52]. When considering multiple samples, DNA sequences are usually compared individually to a reference genome: an agreed-upon sequence that is considered to represent an organisms genome. Once each DNA sequence has had its SNPs detected, or *called*, the differences between the samples can be compared.

SNPs are frequently occurring. In fact, in humans roughly every 850th base pair is a SNP [53]. Calling SNPs has several use cases. For instance, SNPs can be used as high-resolution markers when comparing genomic regions between samples [54], as well as indicators of diseases in an individual [55]. Modern high-throughput sequencing methods for reading DNA often make use of a technique called *massively parallel sequencing*, to read sequences longer than ~200 base pairs, with a sufficiently small error rate. This is done by cleaving multiple copies of the source DNA into random fragments (called *reads*) that are small enough to be accurately read, and then by aligning them to a reference genome. The overlapping fragments together form the sequence of the source DNA.

In order to accurately sequence 3 billion bases from a single human individual, 30-fold more reads data needs to be sequenced [1]. This makes SNP calling data-intensive, thus requiring parallelization. A simple MapReduce-oriented approach consists of: (i) distributing the reads across several nodes, (ii) aligning the reads to a reference genome in parallel and (iii) calling the SNPs with respect to the reference genome. The last step requires all the reads from a chromosome to be included in the SNP calling, thus the maximum allowed parallelism is equal to the total number of chromosomes. Listing 3 shows how the described parallelization can be implemented in MaRe, using BWA for the alignment [56] and GATK [57] for the SNP calling. As opposite the VS example, BWA and GATK provide a multithreaded implementation of the algorithms. Therefore, in listing 3, we leverage such implementation for single-node parallelization.

Listing 3. SNP Calling in MaRe

```
val snpRDD = new MaRe(readsRDD).map(
  inputMountPoint = TextFile("/in.fastq"),
  outputMountPoint = TextFile("/out.sam"),
  imageName = "mcapuccini/alignment:latest",
  command = """
    bwa mem -t 8 \
      -p /ref/human_g1k_v37.fasta \
      /in.fastq \
      | samtools view > /out.sam
  """
).repartitionBy(
    keyBy = (sam: String) => parseChromosomeId(sam),
    numPartitions = numberOfNodes
).map(
  inputMountPoint = TextFile("/in.sam"),
  outputMountPoint = BinaryFiles("/out"),
    imageName = "mcapuccini/alignment:latest",
    command = """
      cat /ref/human_g1k_v37.dict /in.sam \
        > /in.hdr.sam
      gatk AddOrReplaceReadGroups \
        --INPUT=/in.hdr.sam \
        --OUTPUT=/in.hdr.sort.rg.bam \
        --SORT_ORDER=coordinate \
        [ ... header options ... ]
      gatk BuildBamIndex \
        --INPUT=/in.hdr.sort.rg.bam
      gatk HaplotypeCallerSpark \
        -R /ref/human_g1k_v37.fasta \
        -I /in.hdr.sort.rg.bam \
        -O /out/${RANDOM}.g.vcf
      gzip /out/*
  """
).reduce(
```

```
35    inputMountPoint = BinaryFiles("/in"),
36    outputMountPoint = BinaryFiles("/out"),
37    imageName = "opengenomics/vcftools-tools:latest",
38    command = """
39      vcf-concat /in/*.vcf.gz \
40        | gzip -c > /out/merged.${RANDOM}.g.vcf.gz
41    """
42  )
```

In listing 3, MaRe is initialized by passing an RDD containing the reads for a human individual in interleaved FASTQ format[58] (`readsRDD` on line 1). We implement the parallel reads alignment using the `map` primitive. From line 2 to 4, we set the mount points as text files, and we specify a publicly available Docker image containing the necessary software tools. On line 5 we specify the BWA command and we set: (i) `-t 8` to utilize 8 threads, (ii) `-p /ref/human_g1k_v37.fasta` to specify the reference genome location (in the container) and (iii) the input mount point `/in.fastq`. In addition, on line 9 we pipe the results to another software, called `samtools` [59], to convert them from the binary BAM format [59] to the text SAM format [59]. Converting the results to text format makes it easier to parse the chromosome location in the next step.

When calling SNPs, GATK needs to read all of the aligned reads for a certain DNA region. Using chromosomes to define the regions makes sure that no reads will span a region break point - a problem that would need to be handled if chromosomes were to be split in smaller regions. To achieve this we need to: (i) perform a chromosome-wise repartition of the dataset and (ii) allow MaRe to write temporary mount point data to disk. Point (ii) is enabled by setting the `TMPDIR` environment variable to a disk mount, in the Apache Zeppelin configuration. Even if this ~~is not optimal in terms of performance, it~~ could potentially edge performance, this is necessary as the full partition size exceeds the ~~tmpfs~~ *tmpfs* capacity in our worker nodes. Point (i) is implemented by using the `repartitionBy` primitive, on line 11. In particular, we specify a `keyBy` function that parses and returns a the chromosome identifier (on line 12), and a number of partitions that is equal to the number of worker nodes (on line 13).

The `map` primitive (on line 14) uses the chromosome-wise partitioning to perform the SNP calling, with GATK. Since the data is in SAM format, we set the input mount point as text file (line 15). However, since we are going to zip the results before aggregating the SNPs (line 32), we set the output mount point as a binary files directory (`"/out"`, on line 16). On line 17, we set the same Docker image that we used for the initial mapping step and, on line 18, we specify a command that: (i) prepends the necessary SAM header to the input data (which is available inside the container under `/ref/human_g1k_v37.dic`, on line 19), (ii) coverts the SAM input in BAM format (line 23), (iii) builds an index for the BAM format (line 26) and (iv) runs the multithreaded SNP calling using GATK, producing a Variant Call Format (VCF) file [60] (line 28). A detailed description of the options, used for each command, can be found in the GATK documentation [61].

Finally, to aggregate the SNPs to a single zipped file, we use the `reduce` primitive. In this case we use binary file mount points (lines 35 and 36) and a publicly available image containing the VCFtools software [60] (line 37). On line 39, the specified command uses `vcf-concat` to merge all of the VCF files in the input mount point, and then it zips and writes them to the output mount point (line 40). Since MaRe applies the reduce command iteratively, intermediate partitions will contain multiple files. Therefore, to avoid file-name clashes, we include a random identifier in the command output (`$RANDOM` at line 40).

We benchmarked the analysis in listing 3 against the full individual reads dataset `HG02666` (~30GB compressed FASTQ files), from the 1000 Genomes Project (1KGP) [53]. ~~Since the tools run multiple threads, we configured the "spark.task.cpus" property to~~ The benchmark ran on top of a stand-alone Apache Spark cluster composed of 1 master and 14 worker nodes. Each node provided 8 ~~in order to ensure proper resource allocation in our cluster setup. Amazon S3 hosts the full 1KGP dataset () and represents a common ingestion source for this use case. The full benchmark runs in ~1.8 hours when using 128 vCPUs, including data ingestion from S3. When studying the WSE for this application we do not consider the ingestion time, as obviously S3 does not host random samples of the dataset. In fact, when performing the runs to compute the WSE, we downsampled the data at run time. Under this assumption the input size of the ingestion phase is static. Therefore, instead of evaluating the ingestion in terms of WSE, we show how the speed increased when adding~~ cores and 40GB of memory, thus resulting in a total of 112 cores and 480GB of memory. In addition, since after the chromosome-wise repartitioning, the partition size exceeded the *tmpfs* space in our workers, we used cloud favors with a local Solid State Drive (SSD). This allowed to write and read the temporary mount point data faster when compared to the previous benchmark. The data was made available to the workers using a co-located HDFS storage. Under these settings, we evaluated the scalability in terms of Strong Scaling Efficiency (SSE). This performance metric shows how the system scale when increasing the parallelism while keeping the input size static. We evaluated this benchmark using SSE instead of WSE as there is no trivial way for downsampling the reference genome while keeping the behaviour of the tools unaltered; the algorithms end up taking longer as they perform an exhaustive search when the reference genome is downsampled. To compute the SSEs we first ran the benchmark using the dockerized tools on a worker node with their built-in, single-node parallelization. Then, we ran again the pipeline using MaRe on 6, 8, 10, 12 and 14 worker nodes. ~~Figure ?? shows the WSE of the analysis (excluding ingestion)and figure ?? shows the speedup of the ingestion phase. The speedup is computed as~~ Then, let T_1 be the time for running the benchmark using the built-in, single-node parallelization and T_N be the time for running the benchmark using N nodes (for N=6,8,10,12), we computed the SSE as $T_1/(N \times T_N)$ - we did not run on 2 and 4 nodes as the dataset size exceeded the total memory available to the Spark workers in these settings. The ideal case, when doubling the number of nodes, is to be able to run the benchmark twice as fast. Hence, a higher SSE indicates better performance.

Figure 4 shows the SSE for the ~~ingestion time for N workers divided by the ingestion time for 1 worker. The WSE oscillates between 0.70 and 0.80 up to 64 vCPUs, and it decreases to ~0.6 at 128 vCPUs~~full analysis. The SSE starts at 0.76 for 48 cores and decreases to 0.59 when running on 112 cores. Even if this does not show optimal performance, as in the VS use case, it still indicates good scalability. ~~The ingestion speedup is close to ideal for up to 4 workers,~~ Indeed, the full benchmark ran in 3 hours and 24 minutes using MaRe on 112 cores, while it took 28 hours and 14 minutes using the built-in, single-node parallelization - leading to a speedup of 8.3.

The alignment portion of the benchmark uses BWA which allows to input the reads using pipes. It is interesting to compare how the SSE differs when using this input method as opposed to materializing the data on a temporary *ext4* file space. Even though the standard RDD API provides a *pipe* method to do so, as we mentioned previously, this built-in implementation runs the external tool for each RDD record - which would result in a considerable overhead. Instead, we compare the SSE achieved by MaRe with a *pipePartion* method, available in our benchmark repository [39], which pipes entire RDD partitions

though a single dockerized tool instance. Figure 5 shows the results of this comparison. Using pipes improved the SSE by ~0.15 when running on 48 and 64 cores, by ~0.08 when running on 80 and ~~it levels off slightly from 8 to 16 workers~~96 cores and by ~0.12 when running on 112 cores. However, this improvement accounted for saving 6 minutes when running on 112 cores, which is negligible as the full analysis (including variant calling) took more than 3 hours to complete in such setting.

Discussion and conclusions

Big Data applications are getting increasing momentum in life science. Data is nowadays stored and processed in distributed systems, often in a geographically dispersed manner. This introduces a layer of complexity that MapReduce frameworks, such as Apache Spark, excel at handling [62]. Container engines, and in particular Docker, are also becoming an essential part of bioinformatics pipelines as they improve delivery, interoperability and reproducibility of scientific analyses. By enabling application containers in MapReduce, MaRe constitutes an important advancement in the scientific data-processing software ecosystem. When compared to current best practices in bioinformatics, relying solely on using workflow systems to orchestrate data pipelines, MaRe has the advantage of providing locality-aware scheduling, transparent ingestion from heterogeneous storage systems and interactivity. As data becomes larger and more globally distributed, we envision scientists to instantiate MaRe close to the data, and perform interactive analyses via cloud-oriented resources. In addition to the interactive mode, MaRe also support batch-oriented processing. This is important as it enables integration with existing bioinformatics pipelines. In practical terms, a packaged MaRe application can be launched by a workflow engine to enable data-intensive phases in a pipeline, and submitted to any of the resource managers supported by the Apache Spark community (including HPC systems [63]).

In the evaluation section we have shown how researchers can easily implement two widely-used applications in life science, using MaRe. Both analyses can be coded in less than 50 lines of code, and they are seamlessly parallelized. The results show near optimal scalability for the VS application ~~, with HDFS performing slightly better than Swift. The slight performance improvement is because of HDFS co-location with the worker nodes, allowing for less network communication~~with *tmpfs* improving performance over *ext4* only by a negligible factor. The reason why there is no relevant performance improvement in using the former is that the containers running time dominate over the time for materializing data on the temporary file space. Even though this may vary in other applications, in our experience this will often be the case for bioinformatics analyses, not justifying the additional effort in setting up a *tmpfs* space.

Scalability in the SNP calling analysis is reasonably good but far from optimal. The reason for this is that before running the haplotype caller, a reasonable amount of data needs to be shuffled across the nodes as GATK needs to see all of the data for a single chromosome at once in order to function properly, thus causing a large amount of data to be materialized on disk. Such overhead can be partly mitigated by enabling data streams via standard input and output between MaRe and containers ~~, which~~ – as the results in figure 5 show. This constitutes an area for future improvement~~. Pure Apache Sparkimplementations of SNP calling such as ADAM~~ [64] ~~show better scalability than MaRe .~~ , however since GATK is unable to read data from the standard input such improvement would not be directly applicable to the presented use case.

ADAM [65], a genomics data-processing framework built on top of Apache Spark, shows ideal scalability for a few, commonly-used preprocessing steps in genomics pipelines – such as the SNP pipeline that we show in this paper. Nevertheless, in real-world scenarios external software would still need to be employed to compose end-to-end workflows. Indeed, ADAM itself provides a utility to integrate external tools into its pipelines [66]. Since such utility is based on pipes and it does not support application containers natively, it provides less flexibility if compared to MaRe. Indeed, as MaRe is fully interoperable with Apache Spark, our recommendation for running genomics pipelines would be to use ADAM for the supported preprocessing steps and then MaRe to integrate external tools in the workflow.

The benchmarks that we show in this paper are representative of two classes of problems where the application of MaRe could lead to different results in terms of performance. Materializing data is a necessary to support any containerized tool, but our results show that this edges performance when records in large partitions need to be processed all together. In this case, reimplementing the analyses natively in Spark using the language of choice could lead to better performance – ADAM is a good example of this approach. It is however important to point out that ~~while ADAM is application specific, MaRe applies to a variety of use cases in bioinformatics and it stands out by enabling distributed SNP calling in less than 50 lines of code. To this extent~~the the effort of reimplementing existing bioinformatics tools is seldom sustainable by research organization. To give the reader an idea of this, ADAM is the product of a large collaboration ~~, counting~~ maintaining thousands of lines of code. ~~As such effort is not always sustainable, we point out that MaRe provides a reasonably good way of implementing general-purpose data-intensive pipelines with considerably smaller effort~~ Due to the current proliferation and heterogeneity of bioinformatics tools [67, 68], it is hard to imagine that such effort would generally be sustainable for many other applications. To this extent, MaRe stands out as it enables bioinformaticians to develop interoperable, distributed pipelines that scale reasonably well without the need to rewrite the existing codebase. ~~Finally we highlight that, as opposite to ADAM, MaRe provides interoperability with any bioinformatics tool through the adoption of application containers (including ADAM itself).~~

In conclusion, MaRe provides a MapReduce-oriented model to enable container-based bioinformatics analyses at scale. The project is available on GitHub [69] under an open source license, along with all of the code to reproduce the analyses in the evaluation section [39].

Methods

Apache Spark

Apache Spark is an open source cluster-computing framework, for the analysis of large-scale dataset [70]. The project originally started with the aim of overcoming lack of in-memory processing in traditional MapReduce frameworks. Today, Apache Spark has evolved in a unified analytics engine, encompassing high-level APIs for machine learning, streaming, graph processing and SQL, and it has become the largest open source project in Big Data analytics with over 1000 contributors and over 1000 adopting organizations [25].

Clustering model

The Apache Spark clustering model includes: a driver program, one or more worker nodes and a cluster manager. The driver program is written by the user and controls the flow of the

programmed analysis. For interactive analysis the driver program can run in notebooks environments such as Jupyter [31] and Apache Zeppelin [32]. Worker nodes communicate with the driver program, thus executing the distributed analysis as defined by the user. Finally, a cluster manager handles resources in the cluster, allowing for the executing processes to acquire them in the worker nodes. Apache Spark is cluster-manager agnostic and it can run in stand alone settings, as well as on some popular platforms (e.g., Kubernetes [71], Mesos [72] and Hadoop YARN [73]).

Resilient Distributed Datasets

Resilient Distributed Datasets (RDDs) [33] are central in the Apache Spark programming model. RDDs are an abstraction of a dataset that is partitioned across the worker nodes. Hence, partitions can be operated in parallel in a scalable and fault-tolerant manner, and possibly cached in memory for recurrent access. As a unified processing engine, Apache Spark offers support for ingesting RDDs from numerous big-data-oriented storage systems. RDDs can be operated through: Scala [30], Python [74], Java [75] and R [76] APIs. Such APIs expose RDDs as object collections, and they offer high-level methods to transform the datasets.

The *mapPartition* and the *repartition* methods, from the RDD API, are useful to understand the MaRe implementation. The *mapPartition* method is inspired by functional programming languages. It takes as an argument a lambda expression that codes a data transformation, and it applies it to each partition, returning a new RDD. The *repartition* method, as the name suggests, changes the way the dataset records are partitioned across the worker nodes. It can be used to increase and decrease the number of partitions, thus affecting the level of parallelism, and it can also sort records in partitions, according to custom logics. In this case, an additional RDD method, namely *keyBy*, needs to be used to compute a key for each RDD record. Similarly to *mapPartition*, *keyBy* applies a user-provided lambda expression to compute the record keys. Such keys are then used by *repartition* in conjunction with an extension of the *Partitioner* class [34] to assign records to partitions. For instance, when using *HashPartitioner* [77] records with same key always end up in the same RDD partition.

Stages and data locality

RDD methods are lazily applied to the underlying dataset. This means that until something needs to be written to a storage system, or returned to the driver program, nothing is computed. In this way, Apache Spark can build a direct acyclic graph and thus optimize the physical execution plan. A physical execution plan is composed of processing tasks that are organized in stages. Typically, inside each stage the physical execution plan preserves data locality, while between stages a data shuffle occurs. In particular, a sequence of mapPartition methods generate a single stage, giving place to almost no communication in the physical execution plan. In contrast, each time *repartition* is applied to an RDD, a new stage is generated (and data shuffling occurs).

Docker

Docker has emerged as the de-facto standard application container engine [27]. Like Virtual Machines (VMs), application containers enable the encapsulation of software components so that any compliant computer system can execute them with no additional dependencies [18]. The advantage of Docker and similar container engines over virtualization consists of eliminating the need of running an ~~Operative~~ Operating System (OS) for each isolated environment. As opposite to hypervisors, container engines leverage on kernel namespaces to isolate software environments, and thus run containers straight on the host OS. This makes application containers considerably lighter than VMs, enabling a more granular compartmentalization of software components.

Software Delivery

By enabling the encapsulation of entire software stacks, container engines have the potential of considerably simplify application delivery. Engines such as LXC [78] and Jails [79] have been available for almost two decades. Nevertheless, when compared to Docker these systems are poor in terms of software delivery functionalities. This is the reason why software containers popularity exploded only when Docker emerged.

Docker containers can be defined using a text specification language. Using such language, users compose a *Dockerfile* which is parsed by Docker, and then complied into a Docker image. Docker images can then be released to public or private registries, becoming immediately available over the Internet. Therefore, by running the Docker engine, the end users can conveniently start the released containers locally.

Volumes

When using Docker containers for data processing, volumes play an important role. Indeed, there is a need for a mechanism to pass the input data to the containers, and to retrieve the processed output from the isolated environment. Docker volumes allow for defining shared file spaces between containers and the host OS. Such volumes can be easily created when starting containers, by specifying a mapping between host OS file, or directories, and container mount points. Inside the containers these shared objects simply appear as regular files, or directories, under the specified mount point.

Availability of supporting source code and requirements

Project name: MaRe
Project home page: https://github.com/mcapuccini/MaRe
Operating system(s): Platform independent
Programming language: Scala
Other requirements: Apache Spark and Docker
License: Apache License 2.0
Research Resource Identification Initiative ID: SCR_018069

Availability of supporting data

The data set supporting the VS evaluation in this article is available in the ZINC database [50]. The data set supporting the SNP evaluation is available on Amazon S3 (s3://1000genomes/phase3/data/HG02666).

Declarations

List of abbreviations

1KGP: one thousand genome project; API: application programming interface; CSC: information technology center for science; HDFS: hadoop distributed file system; HPC: high-performance computing; OS: operative system; RDD: resilient distributed dataset; SDF: structure-data file; SNP: single nucleotide polymorphism; VCF: variant call format; VM: virtual machine; VS: virtual screening; WSE: weak scaling efficiency~~; vCPU: virtual central processing unit~~.

Ethics approval and consent to participate

All of the 1KGP data is consented for analysis, publication and distribution. Ethics and consents are extensively explained in the 1KGP publications [53].

Competing interests

The authors declare that they have no competing interests.

Funding

This research was supported by The European Commission's Horizon 2020 programme under grant agreement number 654241 (PhenoMeNal).

Author's contributions

MC and OS conceived the project. MC designed and implemented MaRe. MC and MD carried out the evaluation experiments. MD provided expertise in genomics. ST provided expertise in cloud computing. All authors read and approved the final manuscript.

Acknowledgment

We kindly acknowledge contributions to cloud resources by CSC (https://www.csc.fi), the Nordic e-Infrastructure Collaboration (https://neic.no) and the SNIC Science Cloud [80]. Academic license for docking software was provided by OpenEye Scientific.

References

1. Stephens ZD, Lee SY, Faghri F, Campbell RH, Zhai C, Efron MJ, et al. Big data: astronomical or genomical? PLoS biology 2015;13(7):e1002195.
2. Foster LJ, DeMarco ML, At the Intersection of Proteomics and Big Data Science. Clinical Chemistry; 2017.
3. Peters K, Bradbury J, Bergmann S, Capuccini M, Cascante M, de Atauri P, et al. PhenoMeNal: Processing and analysis of Metabolomics data in the Cloud. GigaScience 2018;8(2):giy149.
4. Peng H. Bioimage informatics: a new area of engineering biology. Bioinformatics 2008;24(17):1827–1836.
5. Brown N, Cambruzzi J, Cox PJ, Davies M, Dunbar J, Plumbley D, et al. Big Data in Drug Discovery. In: Progress in medicinal chemistry, vol. 57 Elsevier; 2018.p. 277–356.
6. Cook CE, Lopez R, Stroe O, Cochrane G, Brooksbank C, Birney E, et al. The European Bioinformatics Institute in 2018: tools, infrastructure and training. Nucleic acids research 2018;47(D1):D15–D22.
7. Tan J, Meng X, Zhang L. Delay tails in MapReduce scheduling. ACM SIGMETRICS Performance Evaluation Review 2012;40(1):5–16.
8. Gearing Up for the Next Challenge in High-Performance Computing;. Accessed: 2019-04-25. https://str.llnl.gov/march-2015/still.
9. Convolbo MW, Chou J, Hsu CH, Chung YC. GEODIS: towards the optimization of data locality-aware job scheduling in geo-distributed data centers. Computing 2018;100(1):21–46.
10. Fox A, Griffith R, Joseph A, Katz R, Konwinski A, Lee G, et al. Above the clouds: A berkeley view of cloud computing. Dept Electrical Eng and Comput Sciences, University of California, Berkeley, Rep UCB/EECS 2009;28(13):2009.
11. Mansouri Y, Toosi AN, Buyya R. Data storage management in cloud environments: Taxonomy, survey, and future directions. ACM Computing Surveys (CSUR) 2018;50(6):91.
12. Williams CL, Sica JC, Killen RT, Balis UG. The growing need for microservices in bioinformatics. Journal of Pathology Informatics 2016;7.
13. Leipzig J. A review of bioinformatic pipeline frameworks. Briefings in bioinformatics 2017;18(3):530–536.
14. Lampa S, Alvarsson J, Spjuth O. Towards agile large-scale predictive modelling in drug discovery with flow-based programming design principles. Journal of cheminformatics 2016;8(1):67.
15. Di Tommaso P, Chatzou M, Floden EW, Barja PP, Palumbo E, Notredame C. Nextflow enables reproducible computational workflows. Nature biotechnology 2017;35(4):316.
16. Moreno P, Pireddu L, Roger P, Goonasekera N, Afgan E, Van Den Beek M, et al. Galaxy-Kubernetes integration: scaling bioinformatics workflows in the cloud. BioRxiv 2018;p. 488643.
17. Novella JA, Emami Khoonsari P, Herman S, Whitenack D, Capuccini M, Burman J, et al. Container-based bioinformatics with Pachyderm. Bioinformatics 2018;35(5):839–846.
18. Open Container Initiative, The 5 principles of Standard Containers; 2016. Accessed: 2019-04-25. https://github.com/opencontainers/runtime-spec/blob/master/principles.md.
19. Dean J, Ghemawat S. MapReduce: simplified data processing on large clusters. Communications of the ACM 2008;51(1):107–113.
20. Bhandarkar M. MapReduce programming with apache Hadoop. In: 2010 IEEE International Symposium on Parallel & Distributed Processing (IPDPS) IEEE; 2010. p. 1–1.
21. Gunarathne T, Wu TL, Qiu J, Fox G. MapReduce in the Clouds for Science. In: 2010 IEEE second international conference on cloud computing technology and science IEEE; 2010. p. 565–572.
22. Mohammed EA, Far BH, Naugler C. Applications of the MapReduce programming framework to clinical big data analysis: current landscape and future trends. BioData mining 2014;7(1):22.
23. Guo R, Zhao Y, Zou Q, Fang X, Peng S. Bioinformatics applications on apache spark. GigaScience 2018;7(8):giy098.
24. Schönherr S, Forer L, Weißensteiner H, Kronenberg F, Specht G, Kloss-Brandstätter A. Cloudgene: A graphical execution platform for MapReduce programs on private and public clouds. BMC bioinformatics 2012;13(1):200.
25. Zaharia M, Xin RS, Wendell P, Das T, Armbrust M, Dave A, et al. Apache spark: a unified engine for big data processing. Communications of the ACM 2016;59(11):56–65.
26. Ding M, Zheng L, Lu Y, Li L, Guo S, Guo M. More convenient more overhead: the performance evaluation of hadoop streaming. In: Proceedings of the 2011 ACM Symposium on Research in Applied Computation ACM; 2011. p. 307–313.
27. Shimel A, Docker becomes de facto Linux standard; 2016. Accessed: 2019-04-25. http://www.networkworld.com/article/2226751/opensource-subnet/docker-becomes-de-facto-linux-standard.html.
28. Kudla G, Lipinski L, Caffin F, Helwak A, Zylicz M. High guanine and cytosine content increases mRNA levels in mammalian cells. PLoS biology 2006;4(6):e180.
29. Ubuntu Docker Image;. Accessed: 2019-04-25. https://hub.docker.com/_/ubuntu.
30. Odersky M, Altherr P, Cremet V, Emir B, Maneth S, Micheloud S, et al. An overview of the Scala programming lan-

guage; 2004.
31. Kluyver T, Ragan-Kelley B, Pérez F, Granger BE, Bussonnier M, Frederic J, et al. Jupyter Notebooks-a publishing format for reproducible computational workflows. In: ELPUB; 2016. p. 87–90.
32. Cheng Y, Liu FC, Jing S, Xu W, Chau DH. Building big data processing and visualization pipeline through apache zeppelin. In: Proceedings of the Practice and Experience on Advanced Research Computing ACM; 2018. p. 57.
33. Zaharia M, Chowdhury M, Das T, Dave A, Ma J, McCauley M, et al. Resilient distributed datasets: A fault-tolerant abstraction for in-memory cluster computing. In: Proceedings of the 9th USENIX conference on Networked Systems Design and Implementation USENIX Association; 2012. p. 2–2.
34. Laskowski J, HashPartitioner;. Accessed: 2019-04-25. https://jaceklaskowski.gitbooks.io/mastering-apache-spark/spark-rdd-HashPartitioner.html.
35. Peek J, O'Reilly T, Loukides M. UNIX power tools 1998;.
36. Tevanian A, Rashid RF, Young M, Golub DB, Thompson MR, Bolosky WJ, et al. A UNIX Interface for Shared Memory and Memory Mapped Files Under Mach. In: USENIX Summer Citeseer; 1987. p. 53–68.
37. Snyder P. tmpfs: A virtual memory file system. In: Proceedings of the autumn 1990 EUUG Conference; 1990. p. 241–248.
38. cPouta IaaS Cloud;. Accessed: 2019-04-25. https://research.csc.fi/cpouta.
39. MaRe Benchmarks;. Accessed: 2019-04-25. https://github.com/mcapuccini/mare-benchmarks.
40. OpenStack Apache Spark Terraform Module;. Accessed: 2019-04-25. https://github.com/mcapuccini/terraform-openstack-spark.
41. Shvachko K, Kuang H, Radia S, Chansler R, et al. The hadoop distributed file system. In: MSST, vol. 10; 2010. p. 1–10.
42. Swift;. Accessed: 2019-04-25. https://www.swiftstack.com.
43. Amazon S3;. Accessed: 2019-04-25. https://aws.amazon.com/s3.
44. Cheng T, Li Q, Zhou Z, Wang Y, Bryant SH. Structure-based virtual screening for drug discovery: a problem-centric review. The AAPS journal 2012;14(1):133–141.
45. McGann M. FRED pose prediction and virtual screening accuracy. Journal of chemical information and modeling 2011;51(3):578–596.
46. sdsorter;. Accessed: 2019-04-25. https://sourceforge.net/projects/sdsorter.
47. Dalby A, Nourse JG, Hounshell WD, Gushurst AK, Grier DL, Leland BA, et al. Description of several chemical structure file formats used by computer programs developed at Molecular Design Limited. Journal of chemical information and computer sciences 1992;32(3):244–255.
48. Bäckbro K, Löwgren S, Österlund K, Atepo J, Unge T, Hultén J, et al. Unexpected binding mode of a cyclic sulfamide HIV-1 protease inhibitor. Journal of medicinal chemistry 1997;40(6):898–902.
49. Papadatos G, Davies M, Dedman N, Chambers J, Gaulton A, Siddle J, et al. SureChEMBL: a large-scale, chemically annotated patent document database. Nucleic acids research 2015;44(D1):D1220–D1228.
50. Irwin JJ, Sterling T, Mysinger MM, Bolstad ES, Coleman RG. ZINC: a free tool to discover chemistry for biology. Journal of chemical information and modeling 2012;52(7):1757–1768.
51. Mathur A, Cao M, Bhattacharya S, Dilger A, Tomas A, Vivier L. The new ext4 filesystem: current status and future plans. In: Proceedings of the Linux symposium, vol. 2 Citeseer; 2007. p. 21–33.
52. Karki R, Pandya D, Elston RC, Ferlini C. Defining "mutation" and "polymorphism" in the era of personal genomics. BMC medical genomics 2015;8(1):37.
53. Consortium GP, et al. A global reference for human genetic variation. Nature 2015;526(7571):68.
54. Collins FS. Medical and Societal Consequences of the Human Genome Project. New England Journal of Medicine 1999;341(1):28–37. https://doi.org/10.1056/NEJM199907013410106, pMID: 10387940.
55. Kruglyak L. Prospects for whole-genome linkage disequilibrium mapping of common disease genes. Nature Genetics 1999;22(2):139–144. https://doi.org/10.1038/9642.
56. Li H, Durbin R. Fast and accurate short read alignment with Burrows–Wheeler transform. bioinformatics 2009;25(14):1754–1760.
57. McKenna A, Hanna M, Banks E, Sivachenko A, Cibulskis K, Kernytsky A, et al. The Genome Analysis Toolkit: a MapReduce framework for analyzing next-generation DNA sequencing data. Genome research 2010;20(9):1297–1303.
58. Cock PJA, Fields CJ, Goto N, Heuer ML, Rice PM. The Sanger FASTQ file format for sequences with quality scores, and the Solexa/Illumina FASTQ variants. Nucleic Acids Res 2010 Apr;38(6):1767–1771. https://www.ncbi.nlm.nih.gov/pubmed/20015970, 20015970[pmid].
59. Li H, Handsaker B, Wysoker A, Fennell T, Ruan J, Homer N, et al. The Sequence Alignment/Map format and SAMtools. Bioinformatics 2009 06;25(16):2078–2079. https://doi.org/10.1093/bioinformatics/btp352.
60. Danecek P, Auton A, Abecasis G, Albers CA, Banks E, DePristo MA, et al. The variant call format and VCFtools. Bioinformatics 2011;27(15):2156–2158.
61. GATK Documentation;. Accessed: 2019-04-25. https://software.broadinstitute.org/gatk/documentation/tooldocs/current.
62. Khanam Z, Agarwal S. Map-reduce implementations: survey and performance comparison. Int J Comput Sci Inf Technol(IJCSIT) 2015;7(4).
63. Chaimov N, Malony A, Canon S, Iancu C, Ibrahim KZ, Srinivasan J. Scaling spark on hpc systems. In: Proceedings of the 25th ACM International Symposium on High-Performance Parallel and Distributed Computing ACM; 2016. p. 97–110.
64. Massie M, Nothaft F, Hartl C, Kozanitis C, Schumacher A, Joseph AD, et al. Adam: Genomics formats and processing patterns for cloud scale computing. University of California, Berkeley Technical Report, No UCB/EECS-2013 2013;207:2013.
65. Nothaft FA, Massie M, Danford T, Zhang Z, Laserson U, Yeksigian C, et al. Rethinking data-intensive science using scalable analytics systems. In: Proceedings of the 2015 ACM SIGMOD International Conference on Management of Data; 2015. p. 631–646.
66. ADAM Pipe API;. Accessed: 2019-04-25. https://adam.readthedocs.io/en/latest/api/pipes/.
67. Duck G, Nenadic G, Filannino M, Brass A, Robertson DL, Stevens R. A survey of bioinformatics database and software usage through mining the literature. PloS one 2016;11(6):e0157989.
68. Dahlö M, Scofield DG, Schaal W, Spjuth O. Tracking the NGS revolution: managing life science research on shared high-performance computing clusters. GigaScience 2018 04;7(5). https://doi.org/10.1093/gigascience/giy028.
69. MaRe;. Accessed: 2019-04-25. https://github.com/mcapuccini/MaRe.
70. Zaharia M, Chowdhury M, Franklin MJ, Shenker S, Stoica I. Spark: Cluster computing with working sets. HotCloud 2010;10(10-10):95.

71. Kubernetes;. Accessed: 2019-04-25. https://kubernetes.io.
72. Hindman B, Konwinski A, Zaharia M, Ghodsi A, Joseph AD, Katz RH, et al. Mesos: A platform for fine-grained resource sharing in the data center. In: NSDI, vol. 11; 2011. p. 22–22.
73. Hadoop YARN;. Accessed: 2019-04-25. https://hadoop.apache.org/docs/current/hadoop-yarn/hadoop-yarn-site/YARN.html.
74. The Python Programming Language;. Accessed: 2019-04-25. http://www.python.org.
75. The Java Programming Language;. Accessed: 2019-04-25. https://docs.oracle.com/javase/8/docs/technotes/guides/language/index.html.
76. Ihaka R, Gentleman R. R: a language for data analysis and graphics. Journal of computational and graphical statistics 1996;5(3):299–314.
77. Laskowski J, Partitioner;. Accessed: 2019-04-25. https://jaceklaskowski.gitbooks.io/mastering-apache-spark/spark-rdd-partitions.html.
78. LXC;. Accessed: 2019-04-25. https://linuxcontainers.org/.
79. Kamp PH, Watson RN. Jails: Confining the omnipotent root. In: Proceedings of the 2nd International SANE Conference, vol. 43; 2000. p. 116.
80. Toor S, Lindberg M, Falman I, Vallin A, Mohill O, Freyhult P, et al. SNIC science cloud (SSC): A national-scale cloud infrastructure for swedish academia. In: 2017 IEEE 13th International Conference on e-Science (e-Science) IEEE; 2017. p. 219–227.

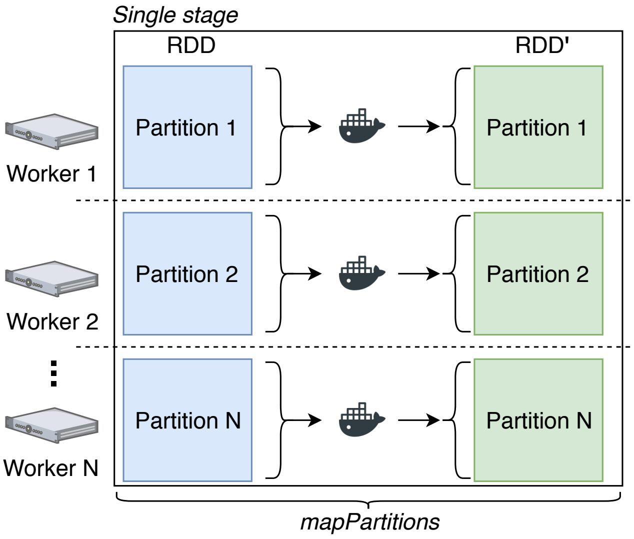

Figure 1. Execution diagram for the `map` primitive. The primitive takes an RDD that is partitioned over N nodes, it transforms each partition using a Docker container and it returns a new RDD'. The logic is implemented using *mapPartitions* from the RDD API. Since *mapPartitions* generates a single stage, data is not shuffled between nodes.

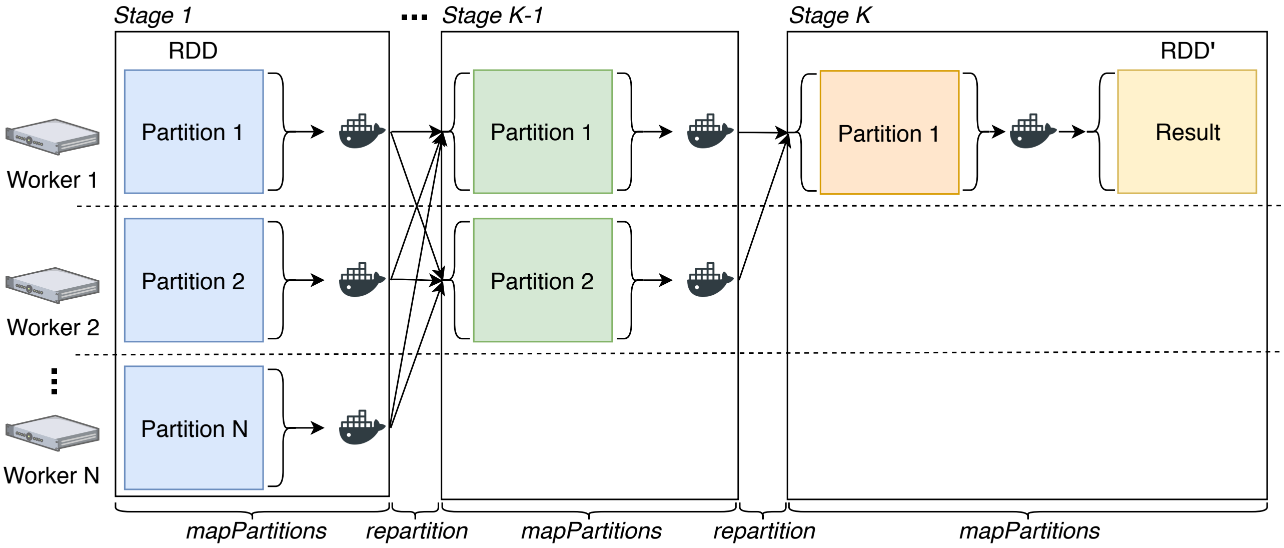

Figure 2. Execution diagram for the `reduce` primitive. The primitive takes an input RDD, partitioned over N nodes, and it iteratively aggregates records using a Docker container, reducing the number of partition until an RDD', containing a single result partition, is returned. The logic is implemented using *mapPartitions* and *repartition* from the RDD API, to aggregate records in partitions and to decrease the number of partitions respectively. Since `repartition` is called in each of the K iterations, K stages are generated, giving place to K data shuffles.

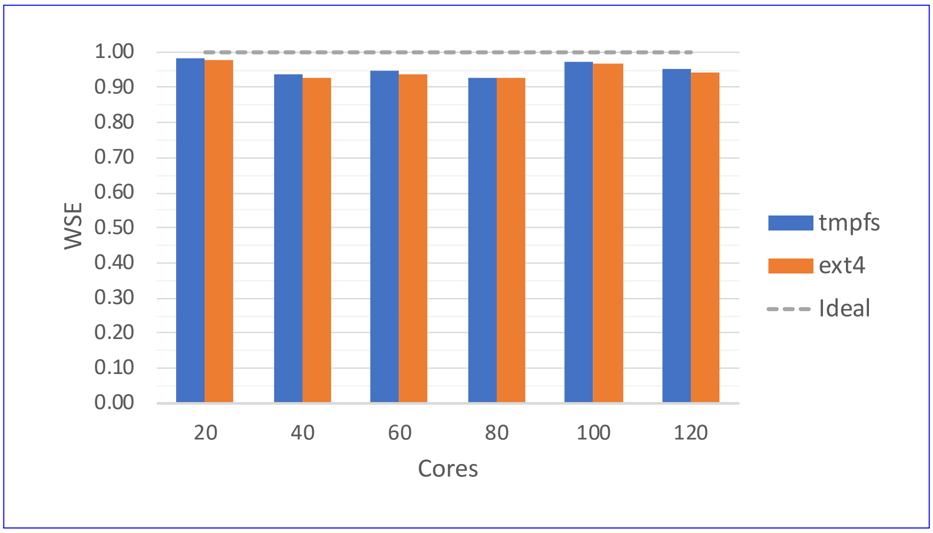

Figure 3. WSE for the VS application implemented in MaRe (listing 2). The results are produced by using SureChEMBL as input ~~,~~ and we show the WSE ~~for two storage backends: HDFS~~ when using *tmpfs* and ~~Swift. Please notice that~~ *ext4* as temporary mount point for passing the ~~*vCPUs* axis is in base two logarithmic scale~~data to the containers.

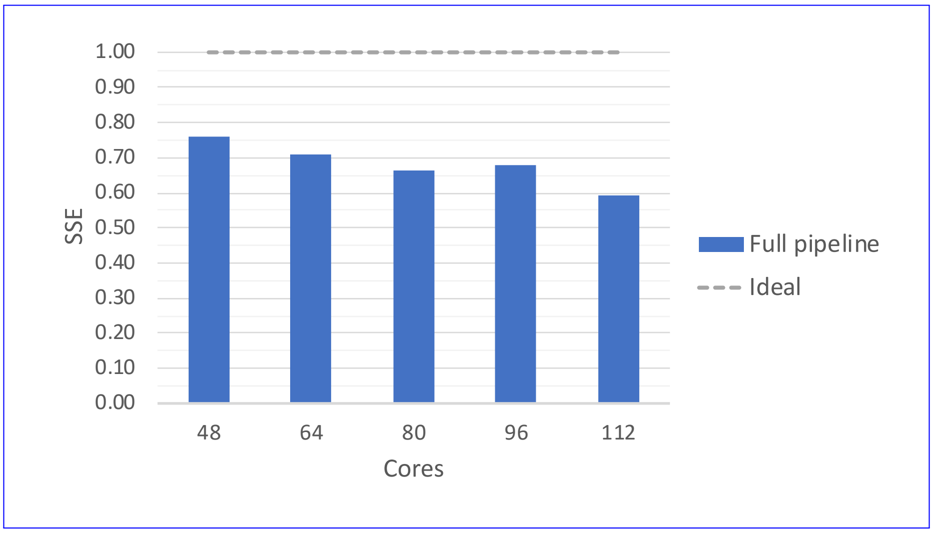

Figure 4. ~~WSE~~ SSE for the SNP calling implemented in MaRe (listing 3). The results are produced by using a full individual dataset from the ~~1000 genomes project~~ 1KGP as input.~~Please notice that the *vCPUs* axis is in base two logarithmic scale.~~

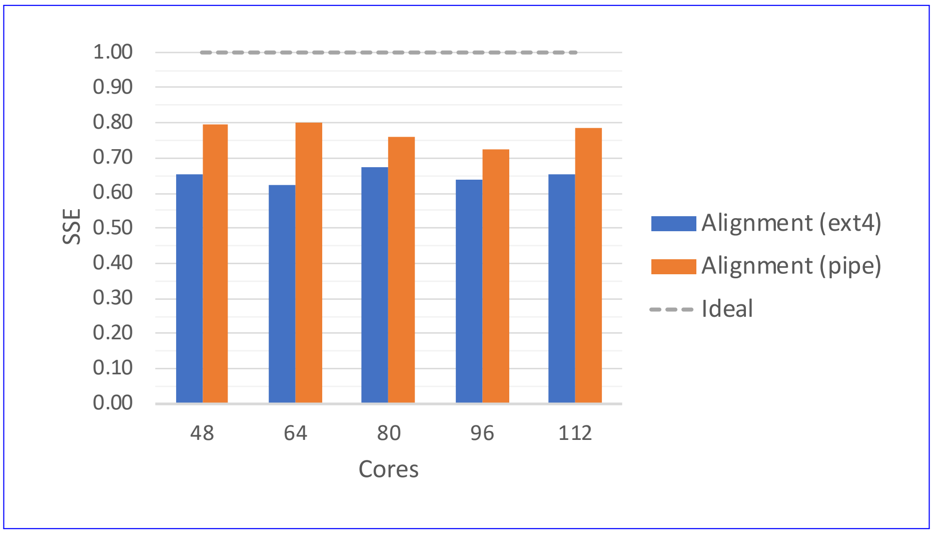

Figure 5. ~~Ingestion speedup~~ SSE for ~~one~~ the SNP calling alignment stage implemented in MaRe (listing 3, lines 1 to 13). The results are produced by using a full individual dataset from the ~~1000 genomes project~~1KGP as input and we show the SSE when using an SSD-based, *ext4* temporary mount point as well as Unix pipes for passing the data to the containers.~~Please notice that both axis are in base two logarithmic scale.~~

Manuscript (clean) Click here to access/download;Manuscript;manuscript.pdf

GigaScience, 2019, 1–12

doi: xx.xxxx/xxxx
Manuscript in Preparation
Technical Note

TECHNICAL NOTE

MaRe: Processing Big Data with Application Containers on Apache Spark

Marco Capuccini[1,2,*], Martin Dahlö[2,3,4], Salman Toor[1] and Ola Spjuth[2]

[1]Department of Information Technology, Uppsala University, Sweden and [2]Department of Pharmaceutical Biosciences, Uppsala University, Sweden and [3]Science for Life Laboratory, Uppsala University, Sweden and [4]Uppsala Multidisciplinary Center for Advanced Computational Science, Uppsala University, Sweden

* marco.capuccini@it.uu.se

Abstract

Background. Life science is increasingly driven by Big Data analytics, and the MapReduce programming model has been proven successful for data-intensive analyses. However, current MapReduce frameworks offer poor support for reusing existing processing tools in bioinformatics pipelines. Further, these frameworks do not have native support for application containers, which are becoming popular in scientific data processing.
Results. Here we present MaRe, an open-source programming library which introduces support for Docker containers in Apache Spark. Apache Spark and Docker are the MapReduce framework and container engine that have collected the largest open source community, thus MaRe provides interoperability with the cutting-edge software ecosystem. We demonstrate MaRe on two data-intensive applications in life science, showing ease of use and scalability.
Conclusions. MaRe enables scalable data-intensive processing in life science with Apache Spark and application containers. When compared with current best practices, that involve the use of workflow systems, MaRe has the advantage of providing data locality, ingestion from heterogeneous storage systems and interactive processing. MaRe is generally-applicable and available as open source software.

Key words: MapReduce; application containers; Big Data; Apache Spark; workflows.

Findings

Background and purpose

Life science is increasingly driven by Big Data analytics. From genomics, proteomics and metabolomics to bioimaging and drug discovery, scientists need to analyze larger and larger amounts of data [1, 2, 3, 4, 5]. This means that datasets can no longer be stored and processed in a researcher's workstation, but they instead need to be handled on distributed systems, at organization level. For instance, the European Bioinformatics Institute, in Hinxton (United Kingdom), offers a total storage capacity of over 160 petabytes for biologically-significant data [6]. Such amounts of data pose major challenges for scientific analyses. First, there is a need to efficiently scale existing processing tools over massive datasets. In fact, bioinformatics software that was originally developed with the simplistic view of small-scale data, will not scale on distributed computing platforms out of the box. The process of adapting such tools may introduce disruptive changes to the existing codebase, and it is generally unsustainable for most organizations. Secondly, the complexity in programming distributed systems may be hard to cope with for most researchers, who instead need to focus on the biological problem at hand. In addition, as life science is exploratory, scientists increasingly demand being able to run interactive analyses rather than submitting jobs to batch systems. Thirdly, when handling Big Data in distributed systems, data locality is a major concern. Indeed, if once data could be shuffled with little regard, with massive datasets it is not only inefficient [7], but also prohibitively expensive in terms of power consumption – estimated to be in the order of several hundred thousand dollars per year for a single

Compiled on: February 10, 2020.
Draft manuscript prepared by the author.

next-generation High-Performance Computing (HPC) cluster [8]. For geographically dispersed datasets, locality-awareness becomes even more challenging, as computing resources need to be dynamically acquired close to the data [9]. Cloud computing solves this problem by enabling the allocation of virtual infrastructure on demand [10]. However, heterogeneity in storage systems for cloud providers [11], makes it hard to abstract data ingestion from many different sources. Finally, as bioinformatics software is characterized by complex software dependencies, deploying and managing a vast collection of tools in a large distributed system also represents a major challenge [12].

Current bionformatics best practices make use of workflow systems, to orchestrate analyses over distributed computing platforms [13]. Workflow systems provide high-level Application Programming Interfaces (APIs) that allow for defining an execution graph of existing processing tools. At run time, the execution graph is used to pipeline the analysis on distributed cloud or HPC resources. Hence, the parallelization of the analysis is transparently carried out, by executing non-dependent tasks at the same time. Cutting-edge workflow systems, such as Luigi [14], NextFlow [15], Galaxy [16] and Pachyderm [17] allow for running processing tools as application containers. This light-weight packaging technology allows for encapsulating complete software environments, so that distributed systems can run the processing tools with no need of additional dependencies, in an isolated manner [18]. Hence, container-enabled workflow systems provide a fairly easy way to define distributed analyses comprising existing bioinformatics tools, and eliminating the need for managing complex software delivery process and dependency management. Nevertheless, workflow-oriented processing falls short when it comes to Big Data analyses. To the best of the authors knowledge, all of these systems utilize a decoupled shared storage system, for synchronization and intermediate results storage. When dealing with large datasets, this translates to a massive and unnecessary communication in the underlying infrastructure. In addition, workflow systems usually support a limited amount of storage backends, not seldom only POSIX file systems, making it hard to ingest data from heterogeneous cloud resources. Finally, due to their batch-oriented nature, it is also intrinsically hard to enable interactive, exploratory analyses using workflow-oriented frameworks.

Google's MapReduce programming model and its associated implementation pioneered uncomplicated Big Data analytics on distributed computing platforms [19]. When using MapReduce, the analysis is defined in a high-level programming language that hides challenging parallel programming details including fault tolerance, data distribution and locality-aware scheduling. Open-source implementations of MapReduce are well established in industrial and scientific applications [20, 21], and numerous success stories in life science have been reported [22, 23, 24].

Apache Spark has emerged as the project that collected the largest community, in the open-source MapReduce ecosystems [25]. In addition to the MapReduce implementation, Apache Spark also provides increasingly important features, such as in-memory, interactive and stream processing. Furthermore, due to broad collaborations in the open-source community, Apache Spark supports all of the major storage systems, enabling data ingestion from heterogeneous cloud resources. These characteristics are particularly appealing for the case of Big Data in life science. Nevertheless, Apache Spark, and other similar frameworks, offer poor support for composing analyses out of existing processing tools. This is usually limited to calling external programs, which can only access data sequentially, without support for application containers [26]. In fact, the main way of implementing analytics in MapReduce-oriented environments is to code each transformation using one of the available APIs. This way of implementing analyses contrasts with current best practices in bioinformatics, that promote the usage of existing tools as application containers with the goal of improving delivery, interoperability and reproducibility of scientific pipelines [15].

Here we introduce MaRe: an open-source programming library that extends Apache Spark, introducing comprehensive support for external tools and application containers in MapReduce. Similarly to container-enabled workflow systems, MaRe allows to define analyses in a high-level language, in which data transformations are performed by application containers. In addition, MaRe provides seamless management of data locality as well as full interoperability, with the Apache Spark ecosystem. This last point allows MaRe analyses to ingest data from heterogeneous cloud storage systems, and also provides support interactive processing. Finally, by supporting Docker, the de facto standard container engine [27], MaRe is compatible with numerous existing container images.

In summary, the key contributions of the presented work are:

- We introduce MaRe: an open-source MapReduce-oriented programming library for container-based data processing on top of Apache Spark.
- We benchmark MaRe on two data-intensive applications in life science, showing ease of use and scalability.

MaRe

Programming Model

We introduce the MaRe programming model using a simple, yet interesting, example in genomics. A DNA sequence can be represented as a text file written in a language of 4 characters: A,T,G,C. The GC content in a DNA sequence has interesting biological implications; for instance there is evidence that GC-rich genes are expressed more efficiently than GC-poor genes [28]. Hence, within a large DNA sequence it can be interesting to count G and C occurrences. Given an Ubuntu Docker image [29], the task can easily be implemented in MaRe using POSIX tools. Listing 1 shows such implementation.

Listing 1. GC count in MaRe

```
val gcCount = new MaRe(genomeRDD).map(
  inputMountPoint = TextFile("/dna"),
  outputMountPoint = TextFile("/count"),
  imageName = "ubuntu",
  command = """
    grep -o '[GC]' /dna | wc -l > /count
  """
).reduce(
  inputMountPoint = TextFile("/counts"),
  outputMountPoint = TextFile("/sum"),
  imageName = "ubuntu",
  command = """
    awk '{s+=$1} END {print s}' /counts > /sum
  """
)
```

Being based on Apache Spark, MaRe has a similar programming model. The control flow of the analysis is coded in Scala [30], by the program in listing 1. Such program is called *driver* in the Apache Spark terminology. The driver program can be packaged and submitted to a cluster (in batch mode), or executed interactively using a notebook environment such as Jupyter [31] or Apache Zeppelin [32]. Listing 1 starts by instantiating a `MaRe` object, which takes a Resilient Distributed Dataset (RDD) [33], containing the input genome file in text

format. Such RDD can be easily loaded using the Apache Spark API from any of the supported storage backends. The `map` primitive (line 1 to 8) applies a command from the Docker image to each partition of the RDD. In our example we specify the Ubuntu image on line 4, and we use a command that combines `grep` and `wc` to filter and count GC occurrences (on line 6). The partition are mounted in the Docker containers in the configured input mount point (`"/dna"` at line 2), and the command results are loaded back to MaRe from the configured output mount point (`"/count"` on line 3). In the example we use *TextFile* mount points as the input data is in text format. By default, MaRe considers each line in a text file as a separate record, but custom record separators can also be configured using the *TextFile* constructor.

At this point it is important to mention that MaRe can also handle binary files. For such data formats, the driver program should specify mount points of type `BinaryFiles`. In this case, each RDD record is considered as a distinct binary file, thus the specified mount point results in a directory containing multiple files (as opposed to `TextFile` that mounts the records in a single file). We provide an example of the `BinaryFiles` mount point in the evaluation section.

Coming back to listing 1, after applying the `map` primitive, each RDD partition is transformed into a distinct GC count. The `reduce` primitive (line 8 to 15), aggregates the counts in each partition to a cumulative sum. Again, we use mount points of type `TextFile`, to mount the intermediate counts in the containers (`"/counts"` on line 9) and to read back the cumulative sum (`"/sum"` on line 10). The sum is computed using the `awk` command from the Ubuntu image (lines 11 to 14). Finally, the result is returned to the `gcCount` variable at line 1.

From the GC example, the reader may have noticed that our programming model is strongly inspired by MapReduce. In addition, Apache Spark users may have noticed that the GC count problem can easily be solved in pure Spark code. Indeed, the aim of the example is just to provide an easy introduction to MaRe, and two real-world applications are available in the evaluation section.

Apart from `map` and `reduce`, MaRe provides an additional primitive. For real-world applications, we noticed that it is often needed to group dataset records according to a specific logic before applying `map` or `reduce`. For this reason, MaRe also provides a `repartitonBy` primitive, which repartitions the RDD records according to a configurable grouping rule. More specifically, the `repartitonBy` primitive takes into account a user-provided `keyBy` function, which is used to compute a key for each record in the dataset. Then, the repartitioning is performed accordingly so that records with same key end up in the same partition. An example of `repartitonBy` is available in the evaluation section.

Implementation

MaRe comes as a thin layer on top of the RDD API [33], and it relies on Apache Spark to provide important features such as data locality, data ingestion, interactive processing, and fault tolerance. The implementation effort consists of: (i) leveraging the RDD API to implement the MaRe primitives and (ii) handling data between containers and RDD structures.

Primitives. Each instance of a `MaRe` object retains an underlying RDD, which represents an abstraction of a dataset that is partitioned across Apache Spark workers. The `map`, `reduce` and `repartitionBy` primitives utilize the underlying RDD API to operate such dataset.

Figure 1 shows the execution diagram for the `map` primitive. For simplicity, in figure 1 we show a single partition per worker, but in reality workers may retain multiple partitions. This primitive takes an input RDD that is partitioned over N nodes, and it transforms each partition using a Docker container command - thus returning a new RDD'. This logic is implemented using *mapPartitions* from the RDD API. When calling *mapPartitions*, MaRe specifies a lambda expression that: (i) makes the data available in the input mount point, (ii) runs the Docker container and (iii) retrieves the results from the output mount point. When using *mapPartitions*, Apache Spark generates a single stage, thus no data shuffle is performed.

Figure 2 shows the execution diagram for the `reduce` primitive. This primitive takes an input RDD, partitioned over N nodes, and it iteratively aggregates records, reducing the number of partition until an RDD', containing a single result partition, is returned. Again, the input RDD may retain multiple partitions per node. However, as opposed to the `map` primitive, RDD' always contains a single partition when it is returned. Given a user-configured depth K, the records in the RDD are aggregated using a tree-like algorithm. In each of the K levels in the tree, the records within each partitions are first aggregated using a Docker container command. Like the `map` primitive, this first transformation is implemented using *mapPartitions*, from the RDD API. Then, the number of partitions is decreased using *repartition* from the RDD API. This process is repeated K times until one single partition is left. At this point the records within the remaining partition are aggregated again using *mapPartitions* (from the RDD API), and RDD' is returned. A new stage is generated each time *repartition* is used. Hence, `reduce` leads to K data shuffles. For this reason, when aggregating records, the user-provided command should always reduce the size of the partition. In addition, for results consistency, the command should perform an associative and commutative operation. By default MaRe sets K to 2, however the user may chose a higher tree depth when it is not possible to sufficiently reduce the dataset size in one go.

Finally, the `repartitionBy` primitive is implemented by using *keyBy*, and then *repartition* from the RDD API. MaRe uses the user-provided grouping rule with *keyBy*, to compute a key for each RDD record, and then it applies `repartition` in counjuction with *HashPartitioner* [34], which makes sure that records with same key end up in the same partition.

Data Handling. One of the advantages of Apache Spark over other MapReduce-like systems is the ability of retaining data in memory. To achieve this when passing the data to the application containers, there are a few options available: (i) Unix pipes [35], (ii) memory-mapped files [36] and (iii) *tmpfs* [37]. Solution (i) and (ii) are the most memory-efficient as they do not need to materialize the data when passing it to the containers. However, (i) allows to see records only once in a stream-like manner, while (ii) requires the container-wrapped tools to be able to read from a memory-mapped file. Apache Spark loads data in memory sequentially and partiton-wise. Partition size is configurable and often equals to the block size in the underlying storage system. For the Hadoop Distributed File System (HDFS) this value defaults to 128MB, meaning that on a 8-core machine materializing again partitions on an in-memory file system would require 2GB of memory in total – which is usually not a problem on modern data centers. Therefore, to support any wrapped-tool, we decided to start by implementing solution (iii). This means that MaRe uses an in-memory *tmpfs* file system as temporary file space for the input and output mount points. The solution allows to provide a standard POSIX mount point to the containers, while still retaining reasonable performance [37]. However, MaRe also provides users with the option of selecting any other disk-based file system for the temporary mount points. Even if this could in principle edge performance, this can be useful when a dockerized tool does not allow for splitting large partitions in smaller chunks of records – we show an example of this in the evaluation sec-

tion.

Evaluation

We evaluate MaRe on two data-intensive applications in life science. The first application can be decomposed to somewhat independent jobs, where the data assigned to each job can be relatively small. This is where MapReduce-oriented programming libraries such as MaRe excel. Conversely, the second application requires to compute larger chunks of data all at once, thus allowing us to show the performance penalty that is introduced in such case. More in detail we evaluate: (i) how the analyses can be implemented in MaRe and (ii) how the analyses scale over multiple nodes. To the best of our knowledge, no stable Spark-native implementation of the tools presented in the analyses is publicly available, making a fair performance comparison with a system that does not delegate data processing to an external application container unfeasible. To this extent, we would like to add that if such implementation were available there would be no advantage in rewriting the analyses using our programming library.

The scalability experiments were carried out on cPouta: an OpenStack-based cloud service operated by the Information Technology Center for Science (CSC) in Finland [38]. The driver programs were run interactively using an Apache Zeppelin environment [32], and the notebooks were made available to sustain reproducibility [39]. In addition, we also made available a deployment automation that enables to replicate our setup on cPouta, as well as any other OpenStack-based cloud provider [40].

Virtual Screening

Virtual Screening (VS) is a computer-based method to identify potential drug candidates, by evaluating the binding affinity of virtual compounds against a biological target protein [41]. Given a 3D target structure, a molecular docking software is run against a large library of known molecular representations. For each compound in the virtual molecular library the docking software produces a pose, representing the orientation of the molecule in the target structure, and a binding affinity score. The poses with the highest affinity scores can be considered as potential drug leads for the target protein.

VS is data-intensive as molecular libraries usually contain millions of compounds. A simple, yet effective, approach to scale VS consists of: (i) distributing the molecular library over several nodes, (ii) running the docking software in parallel and (iii) aggregating the top-scoring poses. Listing 2 shows how this logic can be implemented in MaRe, using FRED [42] as molecular docking software, and sdsorter [43] to filter the top-scoring poses.

Listing 2. Virtual Screening in MaRe

```
1  val topPosesRDD = new MaRe(libraryRDD).map(
2    inputMountPoint = TextFile("/in.sdf", "\n$$$$\n"),
3    outputMountPoint = TextFile("/out.sdf", "\n$$$$\n"),
4    imageName = "mcapuccini/oe:latest",
5    command = """
6      fred -receptor /var/openeye/hiv1_protease.oeb \
7        -hitlist_size 0 \
8        -conftest none \
9        -dbase /in.sdf \
10       -docked_molecule_file /out.sdf
11   """
12 ).reduce(
13   inputMountPoint = TextFile("/in.sdf", "\n$$$$\n"),
14   outputMountPoint = TextFile("/out.sdf", "\n$$$$\n"),
15   imageName = "mcapuccini/sdsorter:latest",
16   command = """
17     sdsorter -reversesort="FRED Chemgauss4 score" \
18       -keep-tag="FRED Chemgauss4 score" \
19       -nbest=30 \
20       /in.sdf /out.sdf
21   """
22 )
```

In listing 2, we initialize MaRe by passing it a molecular library that was previously loaded as an RDD (`libraryRDD` on line 1). We implement the parallel molecular docking using the `map` primitive. On line 2 and 3, we set input and output mount points as text files, and assuming the library to be in Structure-Data File (SDF) format [44] we use the custom record separator: `"\n$$$$\n"`. On line 4, we specify a Docker image containing FRED. The image is not publicly available as it also contains our FRED license, but the license can be obtained free of charge for research purposes and we provide a *Dockerfile* [39] to build the image. On line 5, we specify the FRED command. We use a HIV-1 protease receptor [45] as target (which is wrapped in the Docker image), and we set: (i) `-hitlist_size 0` to not filter the poses in this stage, (ii) `-conftest none` to consider the input molecules as single conformations, (iii) `-dbase /in.sdf` to read the input molecules from the input mount point and (iv) `-docked_molecule_file /out.sdf` to write the poses to the output mount point.

The map phase produces a pose for each molecule in `libraryRDD`. On line 12, we use the `reduce` primitive to filter the top 30 poses. On line 13 and 14, we set the input and output mount points as we do for the `map` primitive. On line 15, we specify a publicly available Docker image containing sdsorter. On line 16, we specify the sdsorter command, and we set: (i) `-reversesort="FRED Chemgauss4 score"` to sort the poses from highest to lowest FRED score, (ii) `-keep-tag="FRED Chemgauss4 score"` to keep the score in the results, (iii) `-nbest=30` to output the top 30 poses and (iv) `/in.sdf /out.sdf` to read and write from the input mount point and to the output mount point respectively. Please notice that this command performs an associative and commutative operation, thus ensuring correctness in the reduce phase. Finally, the results are returned to `topPosesRDD`, on line 1.

We benchmarked the analysis coded in listing 2 against the SureChEMBL library [46] retrieved from the ZINC database [47], containing ∼2.2M molecules. The benchmark ran on top of a stand-alone Apache Spark cluster composed of 1 master and 12 worker nodes. Each node provided 10 cores and 43GB of memory, thus resulting in a total of 120 cores and 516GB of memory. The data was made available to the workers using a co-located HDFS storage. Under these settings, we evaluated the scalability in terms of Weak Scaling Efficiency (WSE). This performance metric shows how the system scale when increasing data and parallelism. To compute the WSEs we first ran the benchmark on 1/12 of the dataset using the dockerized tools on a worker node using their built-in, single-node parallelization. Then, we ran again the pipeline using MaRe on 2/12, 4/12, 6/12, ...and 12/12 of the datasets, using 2, 4, 6, ...and 12 worker nodes respectively. The WSE is then computed as the time for processing 1/12 of the data using the built-in, single-node parallelization, divided by the time for processing N/12 of the data using N nodes (for N=2,4,6,...,12). The ideal case, when doubling the number of nodes, is to be able to process twice as much data in the same amount of time. Hence, a higher WSE indicates better performance.

Figure 3 shows the WSE for the full analysis, when using *tmpfs* and a disk-based, *ext4* file system [48] as temporary mount points. From the experiments it emerges that there is little difference between the two methods in terms of scaling

efficiency – *tmpfs* improved the WSE by 0.02 at most. Indeed, the results in figure 3 indicate very good scalability with a WSE close to ideal for both *tmpfs* and *ext4*. For 120 cores, the full benchmark ran in 2 hours and 21 minutes while 1/12 of the input data was processed by the the built-in, single-node parallelization in 2 hours and 14 minutes – resulting in 0.94 WSE. This means that the overhead introduced by MaRe accounts for only 7 minutes in total.

Finally, to ensure the correctness of the parallelization, we ran sdsorter and FRED on a single core against 1K molecules that we randomly sampled from SureChEMBL, and we compared the results with those produced by the code in listing 2.

Single Nucleotide Polymorphism Calling

A Single Nucleotide Polymorphism (SNP) is a position in a DNA sequence where a single nucleotide (or base pair) is different when compared to another DNA sequence [49]. When considering multiple samples, DNA sequences are usually compared individually to a reference genome: an agreed-upon sequence that is considered to represent an organisms genome. Once each DNA sequence has had its SNPs detected, or *called*, the differences between the samples can be compared.

SNPs are frequently occurring. In fact, in humans roughly every 850th base pair is a SNP [50]. Calling SNPs has several use cases. For instance, SNPs can be used as high-resolution markers when comparing genomic regions between samples [51], as well as indicators of diseases in an individual [52]. Modern high-throughput sequencing methods for reading DNA often make use of a technique called *massively parallel sequencing*, to read sequences longer than ~200 base pairs, with a sufficiently small error rate. This is done by cleaving multiple copies of the source DNA into random fragments (called *reads*) that are small enough to be accurately read, and then by aligning them to a reference genome. The overlapping fragments together form the sequence of the source DNA.

In order to accurately sequence 3 billion bases from a single human individual, 30-fold more reads data needs to be sequenced [1]. This makes SNP calling data-intensive, thus requiring parallelization. A simple MapReduce-oriented approach consists of: (i) distributing the reads across several nodes, (ii) aligning the reads to a reference genome in parallel and (iii) calling the SNPs with respect to the reference genome. The last step requires all the reads from a chromosome to be included in the SNP calling, thus the maximum allowed parallelism is equal to the total number of chromosomes. Listing 3 shows how the described parallelization can be implemented in MaRe, using BWA for the alignment [53] and GATK [54] for the SNP calling. As opposite the VS example, BWA and GATK provide a multithreaded implementation of the algorithms. Therefore, in listing 3, we leverage such implementation for single-node parallelization.

Listing 3. SNP Calling in MaRe

```
1  val snpRDD = new MaRe(readsRDD).map(
2    inputMountPoint = TextFile("/in.fastq"),
3    outputMountPoint = TextFile("/out.sam"),
4    imageName = "mcapuccini/alignment:latest",
5    command = """
6      bwa mem -t 8 \
7        -p /ref/human_g1k_v37.fasta \
8        /in.fastq \
9        | samtools view > /out.sam
10   """
11 ).repartitionBy(
12     keyBy = (sam: String) => parseChromosomeId(sam),
13     numPartitions = numberOfNodes
14 ).map(
15     inputMountPoint = TextFile("/in.sam"),
16     outputMountPoint = BinaryFiles("/out"),
17     imageName = "mcapuccini/alignment:latest",
18     command = """
19       cat /ref/human_g1k_v37.dict /in.sam \
20         > /in.hdr.sam
21       gatk AddOrReplaceReadGroups \
22         --INPUT=/in.hdr.sam \
23         --OUTPUT=/in.hdr.sort.rg.bam \
24         --SORT_ORDER=coordinate \
25         [ ... header options ... ]
26       gatk BuildBamIndex \
27         --INPUT=/in.hdr.sort.rg.bam
28       gatk HaplotypeCallerSpark \
29         -R /ref/human_g1k_v37.fasta \
30         -I /in.hdr.sort.rg.bam \
31         -O /out/${RANDOM}.g.vcf
32       gzip /out/*
33   """
34 ).reduce(
35     inputMountPoint = BinaryFiles("/in"),
36     outputMountPoint = BinaryFiles("/out"),
37     imageName = "opengenomics/vcftools-tools:latest",
38     command = """
39       vcf-concat /in/*.vcf.gz \
40         | gzip -c > /out/merged.${RANDOM}.g.vcf.gz
41     """
42 )
```

In listing 3, MaRe is initialized by passing an RDD containing the reads for a human individual in interleaved FASTQ format[55] (`readsRDD` on line 1). We implement the parallel reads alignment using the `map` primitive. From line 2 to 4, we set the mount points as text files, and we specify a publicly available Docker image containing the necessary software tools. On line 5 we specify the BWA command and we set: (i) `-t 8` to utilize 8 threads, (ii) `-p /ref/human_g1k_v37.fasta` to specify the reference genome location (in the container) and (iii) the input mount point `/in.fastq`. In addition, on line 9 we pipe the results to another software, called `samtools` [56], to convert them from the binary BAM format [56] to the text SAM format [56]. Converting the results to text format makes it easier to parse the chromosome location in the next step.

When calling SNPs, GATK needs to read all of the aligned reads for a certain DNA region. Using chromosomes to define the regions makes sure that no reads will span a region break point – a problem that would need to be handled if chromosomes were to be split in smaller regions. To achieve this we need to: (i) perform a chromosome-wise repartition of the dataset and (ii) allow MaRe to write temporary mount point data to disk. Point (ii) is enabled by setting the `TMPDIR` environment variable to a disk mount, in the Apache Zeppelin configuration. Even if this could potentially edge performance, this is necessary as the full partition size exceeds the *tmpfs* capacity in our worker nodes. Point (i) is implemented by using the `repartitionBy` primitive, on line 11. In particular, we specify a `keyBy` function that parses and returns a the chromosome identifier (on line 12), and a number of partitions that is equal to the number of worker nodes (on line 13).

The `map` primitive (on line 14) uses the chromosome-wise partitioning to perform the SNP calling, with GATK. Since the data is in SAM format, we set the input mount point as text file (line 15). However, since we are going to zip the results before aggregating the SNPs (line 32), we set the output mount point as a binary files directory (`"/out"`, on line 16). On line 17, we set the same Docker image that we used for the initial mapping step and, on line 18, we specify a command that: (i)

prepends the necessary SAM header to the input data (which is available inside the container under `/ref/human_g1k_v37.dic`, on line 19), (ii) coverts the SAM input in BAM format (line 23), (iii) builds an index for the BAM format (line 26) and (iv) runs the multithreaded SNP calling using GATK, producing a Variant Call Format (VCF) file [57] (line 28). A detailed description of the options, used for each command, can be found in the GATK documentation [58].

Finally, to aggregate the SNPs to a single zipped file, we use the `reduce` primitive. In this case we use binary file mount points (lines 35 and 36) and a publicly available image containing the VCFtools software [57] (line 37). On line 39, the specified command uses `vcf-concat` to merge all of the VCF files in the input mount point, and then it zips and writes them to the output mount point (line 40). Since MaRe applies the reduce command iteratively, intermediate partitions will contain multiple files. Therefore, to avoid file-name clashes, we include a random identifier in the command output (`$RANDOM` at line 40).

We benchmarked the analysis in listing 3 against the full individual reads dataset `HG02666` (∼30GB compressed FASTQ files), from the 1000 Genomes Project (1KGP) [50]. The benchmark ran on top of a stand-alone Apache Spark cluster composed of 1 master and 14 worker nodes. Each node provided 8 cores and 40GB of memory, thus resulting in a total of 112 cores and 480GB of memory. In addition, since after the chromosome-wise repartitioning, the partition size exceeded the *tmpfs* space in our workers, we used cloud favors with a local Solid State Drive (SSD). This allowed to write and read the temporary mount point data faster when compared to the previous benchmark. The data was made available to the workers using a co-located HDFS storage. Under these settings, we evaluated the scalability in terms of Strong Scaling Efficiency (SSE). This performance metric shows how the system scale when increasing the parallelism while keeping the input size static. We evaluated this benchmark using SSE instead of WSE as there is no trivial way for downsampling the reference genome while keeping the behaviour of the tools unaltered; the algorithms end up taking longer as they perform an exhaustive search when the reference genome is downsampled. To compute the SSEs we first ran the benchmark using the dockerized tools on a worker node with their built-in, single-node parallelization. Then, we ran again the pipeline using MaRe on 6, 8, 10, 12 and 14 worker nodes. Then, let T_1 be the time for running the benchmark using the built-in, single-node parallelization and T_N be the time for running the benchmark using N nodes (for N=6,8,10,12), we computed the SSE as $T_1/(N \times T_N)$ – we did not run on 2 and 4 nodes as the dataset size exceeded the total memory available to the Spark workers in these settings. The ideal case, when doubling the number of nodes, is to be able to run the benchmark twice as fast. Hence, a higher SSE indicates better performance.

Figure 4 shows the SSE for the full analysis. The SSE starts at 0.76 for 48 cores and decreases to 0.59 when running on 112 cores. Even if this does not show optimal performance, as in the VS use case, it still indicates good scalability. Indeed, the full benchmark ran in 3 hours and 24 minutes using MaRe on 112 cores, while it took 28 hours and 14 minutes using the built-in, single-node parallelization – leading to a speedup of 8.3.

The alignment portion of the benchmark uses BWA which allows to input the reads using pipes. It is interesting to compare how the SSE differs when using this input method as opposed to materializing the data on a temporary *ext4* file space. Even though the standard RDD API provides a *pipe* method to do so, as we mentioned previously, this built-in implementation runs the external tool for each RDD record – which would result in a considerable overhead. Instead, we compare the SSE achieved by MaRe with a *pipePartion* method, available in our benchmark repository [39], which pipes entire RDD partitions though a single dockerized tool instance. Figure 5 shows the results of this comparison. Using pipes improved the SSE by ∼0.15 when running on 48 and 64 cores, by ∼0.08 when running on 80 and 96 cores and by ∼0.12 when running on 112 cores. However, this improvement accounted for saving 6 minutes when running on 112 cores, which is negligible as the full analysis (including variant calling) took more than 3 hours to complete in such setting.

Discussion and conclusions

Big Data applications are getting increasing momentum in life science. Data is nowadays stored and processed in distributed systems, often in a geographically dispersed manner. This introduces a layer of complexity that MapReduce frameworks, such as Apache Spark, excel at handling [59]. Container engines, and in particular Docker, are also becoming an essential part of bioinformatics pipelines as they improve delivery, interoperability and reproducibility of scientific analyses. By enabling application containers in MapReduce, MaRe constitutes an important advancement in the scientific data-processing software ecosystem. When compared to current best practices in bioinformatics, relying solely on using workflow systems to orchestrate data pipelines, MaRe has the advantage of providing locality-aware scheduling, transparent ingestion from heterogeneous storage systems and interactivity. As data becomes larger and more globally distributed, we envision scientists to instantiate MaRe close to the data, and perform interactive analyses via cloud-oriented resources. In addition to the interactive mode, MaRe also support batch-oriented processing. This is important as it enables integration with existing bioinformatics pipelines. In practical terms, a packaged MaRe application can be launched by a workflow engine to enable data-intensive phases in a pipeline, and submitted to any of the resource managers supported by the Apache Spark community (including HPC systems [60]).

In the evaluation section we have shown how researchers can easily implement two widely-used applications in life science, using MaRe. Both analyses can be coded in less than 50 lines of code, and they are seamlessly parallelized. The results show near optimal scalability for the VS application with *tmpfs* improving performance over *ext4* only by a negligible factor. The reason why there is no relevant performance improvement in using the former is that the containers running time dominate over the time for materializing data on the temporary file space. Even though this may vary in other applications, in our experience this will often be the case for bioinformatics analyses, not justifying the additional effort in setting up a *tmpfs* space.

Scalability in the SNP calling analysis is reasonably good but far from optimal. The reason for this is that before running the haplotype caller, a reasonable amount of data needs to be shuffled across the nodes as GATK needs to see all of the data for a single chromosome at once in order to function properly, thus causing a large amount of data to be materialized on disk. Such overhead can be partly mitigated by enabling data streams via standard input and output between MaRe and containers – as the results in figure 5 show. This constitutes an area for future improvement, however since GATK is unable to read data from the standard input such improvement would not be directly applicable to the presented use case.

ADAM [61], a genomics data-processing framework built on top of Apache Spark, shows ideal scalability for a few, commonly-used preprocessing steps in genomics pipelines – such as the SNP pipeline that we show in this paper. Nevertheless, in real-world scenarios external software would still

need to be employed to compose end-to-end workflows. Indeed, ADAM itself provides a utility to integrate external tools into its pipelines [62]. Since such utility is based on pipes and it does not support application containers natively, it provides less flexibility if compared to MaRe. Indeed, as MaRe is fully interoperable with Apache Spark, our recommendation for running genomics pipelines would be to use ADAM for the supported preprocessing steps and then MaRe to integrate external tools in the workflow.

The benchmarks that we show in this paper are representative of two classes of problems where the application of MaRe could lead to different results in terms of performance. Materializing data is a necessary to support any containerized tool, but our results show that this edges performance when records in large partitions need to be processed all together. In this case, reimplementing the analyses natively in Spark using the language of choice could lead to better performance – ADAM is a good example of this approach. It is however important to point out that the the effort of reimplementing existing bioinformatics tools is seldom sustainable by research organization. To give the reader an idea of this, ADAM is the product of a large collaboration maintaining thousands of lines of code. Due to the current proliferation and heterogeneity of bioinformatics tools [63, 64], it is hard to imagine that such effort would generally be sustainable for many other applications. To this extent, MaRe stands out as it enables bioinformaticians to develop interoperable, distributed pipelines that scale reasonably well without the need to rewrite the existing codebase.

In conclusion, MaRe provides a MapReduce-oriented model to enable container-based bioinformatics analyses at scale. The project is available on GitHub [65] under an open source license, along with all of the code to reproduce the analyses in the evaluation section [39].

Methods

Apache Spark

Apache Spark is an open source cluster-computing framework, for the analysis of large-scale dataset [66]. The project originally started with the aim of overcoming lack of in-memory processing in traditional MapReduce frameworks. Today, Apache Spark has evolved in a unified analytics engine, encompassing high-level APIs for machine learning, streaming, graph processing and SQL, and it has become the largest open source project in Big Data analytics with over 1000 contributors and over 1000 adopting organizations [25].

Clustering model

The Apache Spark clustering model includes: a driver program, one or more worker nodes and a cluster manager. The driver program is written by the user and controls the flow of the programmed analysis. For interactive analysis the driver program can run in notebooks environments such as Jupyter [31] and Apache Zeppelin [32]. Worker nodes communicate with the driver program, thus executing the distributed analysis as defined by the user. Finally, a cluster manager handles resources in the cluster, allowing for the executing processes to acquire them in the worker nodes. Apache Spark is cluster-manager agnostic and it can run in stand alone settings, as well as on some popular platforms (e.g., Kubernetes [67], Mesos [68] and Hadoop YARN [69]).

Resilient Distributed Datasets

Resilient Distributed Datasets (RDDs) [33] are central in the Apache Spark programming model. RDDs are an abstraction of a dataset that is partitioned across the worker nodes. Hence, partitions can be operated in parallel in a scalable and fault-tolerant manner, and possibly cached in memory for recurrent access. As a unified processing engine, Apache Spark offers support for ingesting RDDs from numerous big-data-oriented storage systems. RDDs can be operated through: Scala [30], Python [70], Java [71] and R [72] APIs. Such APIs expose RDDs as object collections, and they offer high-level methods to transform the datasets.

The *mapPartition* and the *repartition* methods, from the RDD API, are useful to understand the MaRe implementation. The *mapPartition* method is inspired by functional programming languages. It takes as an argument a lambda expression that codes a data transformation, and it applies it to each partition, returning a new RDD. The *repartition* method, as the name suggests, changes the way the dataset records are partitioned across the worker nodes. It can be used to increase and decrease the number of partitions, thus affecting the level of parallelism, and it can also sort records in partitions, according to custom logics. In this case, an additional RDD method, namely *keyBy*, needs to be used to compute a key for each RDD record. Similarly to *mapPartition*, *keyBy* applies a user-provided lambda expression to compute the record keys. Such keys are then used by *repartition* in conjunction with an extension of the *Partitioner* class [34] to assign records to partitions. For instance, when using *HashPartitioner* [73] records with same key always end up in the same RDD partition.

Stages and data locality

RDD methods are lazily applied to the underlying dataset. This means that until something needs to be written to a storage system, or returned to the driver program, nothing is computed. In this way, Apache Spark can build a direct acyclic graph and thus optimize the physical execution plan. A physical execution plan is composed of processing tasks that are organized in stages. Typically, inside each stage the physical execution plan preserves data locality, while between stages a data shuffle occurs. In particular, a sequence of mapPartition methods generate a single stage, giving place to almost no communication in the physical execution plan. In contrast, each time *repartition* is applied to an RDD, a new stage is generated (and data shuffling occurs).

Docker

Docker has emerged as the de-facto standard application container engine [27]. Like Virtual Machines (VMs), application containers enable the encapsulation of software components so that any compliant computer system can execute them with no additional dependencies [18]. The advantage of Docker and similar container engines over virtualization consists of eliminating the need of running an Operating System (OS) for each isolated environment. As opposite to hypervisors, container engines leverage on kernel namespaces to isolate software environments, and thus run containers straight on the host OS. This makes application containers considerably lighter than VMs, enabling a more granular compartmentalization of software components.

Software Delivery

By enabling the encapsulation of entire software stacks, container engines have the potential of considerably simplify application delivery. Engines such as LXC [74] and Jails [75] have been available for almost two decades. Nevertheless, when compared to Docker these systems are poor in terms of software delivery functionalities. This is the reason why software containers popularity exploded only when Docker emerged.

Docker containers can be defined using a text specification

language. Using such language, users compose a *Dockerfile* which is parsed by Docker, and then complied into a Docker image. Docker images can then be released to public or private registries, becoming immediately available over the Internet. Therefore, by running the Docker engine, the end users can conveniently start the released containers locally.

Volumes
When using Docker containers for data processing, volumes play an important role. Indeed, there is a need for a mechanism to pass the input data to the containers, and to retrieve the processed output from the isolated environment. Docker volumes allow for defining shared file spaces between containers and the host OS. Such volumes can be easily created when starting containers, by specifying a mapping between host OS file, or directories, and container mount points. Inside the containers these shared objects simply appear as regular files, or directories, under the specified mount point.

Availability of supporting source code and requirements

Project name: MaRe
Project home page: https://github.com/mcapuccini/MaRe
Operating system(s): Platform independent
Programming language: Scala
Other requirements: Apache Spark and Docker
License: Apache License 2.0
Research Resource Identification Initiative ID: SCR_018069

Availability of supporting data

The data set supporting the VS evaluation in this article is available in the ZINC database [47]. The data set supporting the SNP evaluation is available on Amazon S3 (s3://1000genomes/phase3/data/HG02666).

Declarations

List of abbreviations

1KGP: one thousand genome project; API: application programming interface; CSC: information technology center for science; HDFS: hadoop distributed file system; HPC: high-performance computing; OS: operative system; RDD: resilient distributed dataset; SDF: structure-data file; SNP: single nucleotide polymorphism; VCF: variant call format; VM: virtual machine; VS: virtual screening; WSE: weak scaling efficiency.

Ethics approval and consent to participate

All of the 1KGP data is consented for analysis, publication and distribution. Ethics and consents are extensively explained in the 1KGP publications [50].

Competing interests

The authors declare that they have no competing interests.

Funding

This research was supported by The European Commission's Horizon 2020 programme under grant agreement number 654241 (PhenoMeNal).

Author's contributions

MC and OS conceived the project. MC designed and implemented MaRe. MC and MD carried out the evaluation experiments. MD provided expertise in genomics. ST provided expertise in cloud computing. All authors read and approved the final manuscript.

Acknowledgment

We kindly acknowledge contributions to cloud resources by CSC (https://www.csc.fi), the Nordic e-Infrastructure Collaboration (https://neic.no) and the SNIC Science Cloud [76]. Academic license for docking software was provided by OpenEye Scientific.

References

1. Stephens ZD, Lee SY, Faghri F, Campbell RH, Zhai C, Efron MJ, et al. Big data: astronomical or genomical? PLoS biology 2015;13(7):e1002195.
2. Foster LJ, DeMarco ML, At the Intersection of Proteomics and Big Data Science. Clinical Chemistry; 2017.
3. Peters K, Bradbury J, Bergmann S, Capuccini M, Cascante M, de Atauri P, et al. PhenoMeNal: Processing and analysis of Metabolomics data in the Cloud. GigaScience 2018;8(2):giy149.
4. Peng H. Bioimage informatics: a new area of engineering biology. Bioinformatics 2008;24(17):1827–1836.
5. Brown N, Cambruzzi J, Cox PJ, Davies M, Dunbar J, Plumbley D, et al. Big Data in Drug Discovery. In: Progress in medicinal chemistry, vol. 57 Elsevier; 2018.p. 277–356.
6. Cook CE, Lopez R, Stroe O, Cochrane G, Brooksbank C, Birney E, et al. The European Bioinformatics Institute in 2018: tools, infrastructure and training. Nucleic acids research 2018;47(D1):D15–D22.
7. Tan J, Meng X, Zhang L. Delay tails in MapReduce scheduling. ACM SIGMETRICS Performance Evaluation Review 2012;40(1):5–16.
8. Gearing Up for the Next Challenge in High-Performance Computing;. Accessed: 2019-04-25. https://str.llnl.gov/march-2015/still.
9. Convolbo MW, Chou J, Hsu CH, Chung YC. GEODIS: towards the optimization of data locality-aware job scheduling in geo-distributed data centers. Computing 2018;100(1):21–46.
10. Fox A, Griffith R, Joseph A, Katz R, Konwinski A, Lee G, et al. Above the clouds: A berkeley view of cloud computing. Dept Electrical Eng and Comput Sciences, University of California, Berkeley, Rep UCB/EECS 2009;28(13):2009.
11. Mansouri Y, Toosi AN, Buyya R. Data storage management in cloud environments: Taxonomy, survey, and future directions. ACM Computing Surveys (CSUR) 2018;50(6):91.
12. Williams CL, Sica JC, Killen RT, Balis UG. The growing need for microservices in bioinformatics. Journal of Pathology Informatics 2016;7.
13. Leipzig J. A review of bioinformatic pipeline frameworks. Briefings in bioinformatics 2017;18(3):530–536.
14. Lampa S, Alvarsson J, Spjuth O. Towards agile large-scale predictive modelling in drug discovery with flow-based programming design principles. Journal of cheminformatics 2016;8(1):67.
15. Di Tommaso P, Chatzou M, Floden EW, Barja PP, Palumbo E, Notredame C. Nextflow enables reproducible computational workflows. Nature biotechnology 2017;35(4):316.
16. Moreno P, Pireddu L, Roger P, Goonasekera N, Afgan E, Van

Den Beek M, et al. Galaxy-Kubernetes integration: scaling bioinformatics workflows in the cloud. BioRxiv 2018;p. 488643.
17. Novella JA, Emami Khoonsari P, Herman S, Whitenack D, Capuccini M, Burman J, et al. Container-based bioinformatics with Pachyderm. Bioinformatics 2018;35(5):839–846.
18. Open Container Initiative, The 5 principles of Standard Containers; 2016. Accessed: 2019-04-25. https://github.com/opencontainers/runtime-spec/blob/master/principles.md.
19. Dean J, Ghemawat S. MapReduce: simplified data processing on large clusters. Communications of the ACM 2008;51(1):107–113.
20. Bhandarkar M. MapReduce programming with apache Hadoop. In: 2010 IEEE International Symposium on Parallel & Distributed Processing (IPDPS) IEEE; 2010. p. 1–1.
21. Gunarathne T, Wu TL, Qiu J, Fox G. MapReduce in the Clouds for Science. In: 2010 IEEE second international conference on cloud computing technology and science IEEE; 2010. p. 565–572.
22. Mohammed EA, Far BH, Naugler C. Applications of the MapReduce programming framework to clinical big data analysis: current landscape and future trends. BioData mining 2014;7(1):22.
23. Guo R, Zhao Y, Zou Q, Fang X, Peng S. Bioinformatics applications on apache spark. GigaScience 2018;7(8):giy098.
24. Schönherr S, Forer L, Weißensteiner H, Kronenberg F, Specht G, Kloss-Brandstätter A. Cloudgene: A graphical execution platform for MapReduce programs on private and public clouds. BMC bioinformatics 2012;13(1):200.
25. Zaharia M, Xin RS, Wendell P, Das T, Armbrust M, Dave A, et al. Apache spark: a unified engine for big data processing. Communications of the ACM 2016;59(11):56–65.
26. Ding M, Zheng L, Lu Y, Li L, Guo S, Guo M. More convenient more overhead: the performance evaluation of hadoop streaming. In: Proceedings of the 2011 ACM Symposium on Research in Applied Computation ACM; 2011. p. 307–313.
27. Shimel A, Docker becomes de facto Linux standard; 2016. Accessed: 2019-04-25. http://www.networkworld.com/article/2226751/opensource-subnet/docker-becomes-de-facto-linux-standard.html.
28. Kudla G, Lipinski L, Caffin F, Helwak A, Zylicz M. High guanine and cytosine content increases mRNA levels in mammalian cells. PLoS biology 2006;4(6):e180.
29. Ubuntu Docker Image;. Accessed: 2019-04-25. https://hub.docker.com/_/ubuntu.
30. Odersky M, Altherr P, Cremet V, Emir B, Maneth S, Micheloud S, et al. An overview of the Scala programming language; 2004.
31. Kluyver T, Ragan-Kelley B, Pérez F, Granger BE, Bussonnier M, Frederic J, et al. Jupyter Notebooks-a publishing format for reproducible computational workflows. In: ELPUB; 2016. p. 87–90.
32. Cheng Y, Liu FC, Jing S, Xu W, Chau DH. Building big data processing and visualization pipeline through apache zeppelin. In: Proceedings of the Practice and Experience on Advanced Research Computing ACM; 2018. p. 57.
33. Zaharia M, Chowdhury M, Das T, Dave A, Ma J, McCauley M, et al. Resilient distributed datasets: A fault-tolerant abstraction for in-memory cluster computing. In: Proceedings of the 9th USENIX conference on Networked Systems Design and Implementation USENIX Association; 2012. p. 2–2.
34. Laskowski J, HashPartitioner;. Accessed: 2019-04-25. https://jaceklaskowski.gitbooks.io/mastering-apache-spark/spark-rdd-HashPartitioner.html.
35. Peek J, O'Reilly T, Loukides M. UNIX power tools 1998;.
36. Tevanian A, Rashid RF, Young M, Golub DB, Thompson MR, Bolosky WJ, et al. A UNIX Interface for Shared Memory and Memory Mapped Files Under Mach. In: USENIX Summer Citeseer; 1987. p. 53–68.
37. Snyder P. tmpfs: A virtual memory file system. In: Proceedings of the autumn 1990 EUUG Conference; 1990. p. 241–248.
38. cPouta IaaS Cloud;. Accessed: 2019-04-25. https://research.csc.fi/cpouta.
39. MaRe Benchmarks;. Accessed: 2019-04-25. https://github.com/mcapuccini/mare-benchmarks.
40. OpenStack Apache Spark Terraform Module;. Accessed: 2019-04-25. https://github.com/mcapuccini/terraform-openstack-spark.
41. Cheng T, Li Q, Zhou Z, Wang Y, Bryant SH. Structure-based virtual screening for drug discovery: a problem-centric review. The AAPS journal 2012;14(1):133–141.
42. McGann M. FRED pose prediction and virtual screening accuracy. Journal of chemical information and modeling 2011;51(3):578–596.
43. sdsorter;. Accessed: 2019-04-25. https://sourceforge.net/projects/sdsorter.
44. Dalby A, Nourse JG, Hounshell WD, Gushurst AK, Grier DL, Leland BA, et al. Description of several chemical structure file formats used by computer programs developed at Molecular Design Limited. Journal of chemical information and computer sciences 1992;32(3):244–255.
45. Bäckbro K, Löwgren S, Österlund K, Atepo J, Unge T, Hultén J, et al. Unexpected binding mode of a cyclic sulfamide HIV-1 protease inhibitor. Journal of medicinal chemistry 1997;40(6):898–902.
46. Papadatos G, Davies M, Dedman N, Chambers J, Gaulton A, Siddle J, et al. SureChEMBL: a large-scale, chemically annotated patent document database. Nucleic acids research 2015;44(D1):D1220–D1228.
47. Irwin JJ, Sterling T, Mysinger MM, Bolstad ES, Coleman RG. ZINC: a free tool to discover chemistry for biology. Journal of chemical information and modeling 2012;52(7):1757–1768.
48. Mathur A, Cao M, Bhattacharya S, Dilger A, Tomas A, Vivier L. The new ext4 filesystem: current status and future plans. In: Proceedings of the Linux symposium, vol. 2 Citeseer; 2007. p. 21–33.
49. Karki R, Pandya D, Elston RC, Ferlini C. Defining "mutation" and "polymorphism" in the era of personal genomics. BMC medical genomics 2015;8(1):37.
50. Consortium GP, et al. A global reference for human genetic variation. Nature 2015;526(7571):68.
51. Collins FS. Medical and Societal Consequences of the Human Genome Project. New England Journal of Medicine 1999;341(1):28–37. https://doi.org/10.1056/NEJM199907013410106, pMID: 10387940.
52. Kruglyak L. Prospects for whole-genome linkage disequilibrium mapping of common disease genes. Nature Genetics 1999;22(2):139–144. https://doi.org/10.1038/9642.
53. Li H, Durbin R. Fast and accurate short read alignment with Burrows-Wheeler transform. bioinformatics 2009;25(14):1754–1760.
54. McKenna A, Hanna M, Banks E, Sivachenko A, Cibulskis K, Kernytsky A, et al. The Genome Analysis Toolkit: a MapReduce framework for analyzing next-generation DNA sequencing data. Genome research 2010;20(9):1297–1303.
55. Cock PJA, Fields CJ, Goto N, Heuer ML, Rice PM. The Sanger FASTQ file format for sequences with quality scores, and the Solexa/Illumina FASTQ variants. Nucleic Acids Res 2010 Apr;38(6):1767–1771. https://www.ncbi.nlm.nih.gov/pubmed/20015970, 20015970[pmid].

56. Li H, Handsaker B, Wysoker A, Fennell T, Ruan J, Homer N, et al. The Sequence Alignment/Map format and SAMtools. Bioinformatics 2009 06;25(16):2078–2079. https://doi.org/10.1093/bioinformatics/btp352.
57. Danecek P, Auton A, Abecasis G, Albers CA, Banks E, DePristo MA, et al. The variant call format and VCFtools. Bioinformatics 2011;27(15):2156–2158.
58. GATK Documentation;. Accessed: 2019-04-25. https://software.broadinstitute.org/gatk/documentation/tooldocs/current.
59. Khanam Z, Agarwal S. Map-reduce implementations: survey and performance comparison. Int J Comput Sci Inf Technol(IJCSIT) 2015;7(4).
60. Chaimov N, Malony A, Canon S, Iancu C, Ibrahim KZ, Srinivasan J. Scaling spark on hpc systems. In: Proceedings of the 25th ACM International Symposium on High-Performance Parallel and Distributed Computing ACM; 2016. p. 97–110.
61. Nothaft FA, Massie M, Danford T, Zhang Z, Laserson U, Yeksigian C, et al. Rethinking data-intensive science using scalable analytics systems. In: Proceedings of the 2015 ACM SIGMOD International Conference on Management of Data; 2015. p. 631–646.
62. ADAM Pipe API;. Accessed: 2019-04-25. https://adam.readthedocs.io/en/latest/api/pipes/.
63. Duck G, Nenadic G, Filannino M, Brass A, Robertson DL, Stevens R. A survey of bioinformatics database and software usage through mining the literature. PloS one 2016;11(6):e0157989.
64. Dahlö M, Scofield DG, Schaal W, Spjuth O. Tracking the NGS revolution: managing life science research on shared high-performance computing clusters. GigaScience 2018 04;7(5). https://doi.org/10.1093/gigascience/giy028.
65. MaRe;. Accessed: 2019-04-25. https://github.com/mcapuccini/MaRe.
66. Zaharia M, Chowdhury M, Franklin MJ, Shenker S, Stoica I. Spark: Cluster computing with working sets. HotCloud 2010;10(10-10):95.
67. Kubernetes;. Accessed: 2019-04-25. https://kubernetes.io.
68. Hindman B, Konwinski A, Zaharia M, Ghodsi A, Joseph AD, Katz RH, et al. Mesos: A platform for fine-grained resource sharing in the data center. In: NSDI, vol. 11; 2011. p. 22–22.
69. Hadoop YARN;. Accessed: 2019-04-25. https://hadoop.apache.org/docs/current/hadoop-yarn/hadoop-yarn-site/YARN.html.
70. The Python Programming Language;. Accessed: 2019-04-25. http://www.python.org.
71. The Java Programming Language;. Accessed: 2019-04-25. https://docs.oracle.com/javase/8/docs/technotes/guides/language/index.html.
72. Ihaka R, Gentleman R. R: a language for data analysis and graphics. Journal of computational and graphical statistics 1996;5(3):299–314.
73. Laskowski J, Partitioner;. Accessed: 2019-04-25. https://jaceklaskowski.gitbooks.io/mastering-apache-spark/spark-rdd-partitions.html.
74. LXC;. Accessed: 2019-04-25. https://linuxcontainers.org/.
75. Kamp PH, Watson RN. Jails: Confining the omnipotent root. In: Proceedings of the 2nd International SANE Conference, vol. 43; 2000. p. 116.
76. Toor S, Lindberg M, Falman I, Vallin A, Mohill O, Freyhult P, et al. SNIC science cloud (SSC): A national-scale cloud infrastructure for swedish academia. In: 2017 IEEE 13th International Conference on e-Science (e-Science) IEEE; 2017. p. 219–227.

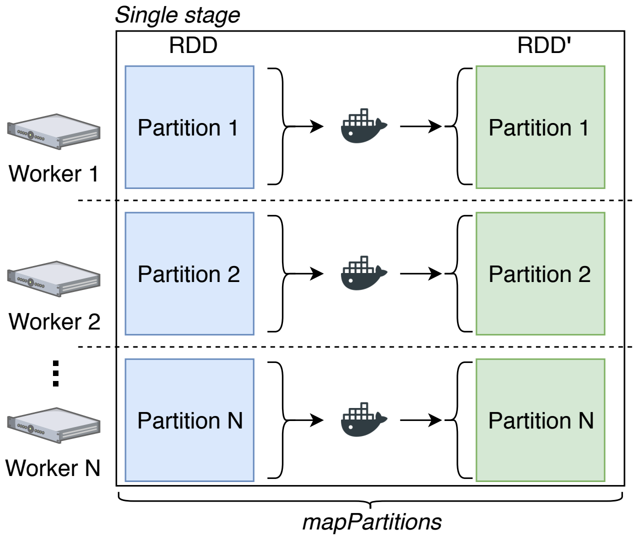

Figure 1. Execution diagram for the `map` primitive. The primitive takes an RDD that is partitioned over N nodes, it transforms each partition using a Docker container and it returns a new RDD'. The logic is implemented using *mapPartitions* from the RDD API. Since *mapPartitions* generates a single stage, data is not shuffled between nodes.

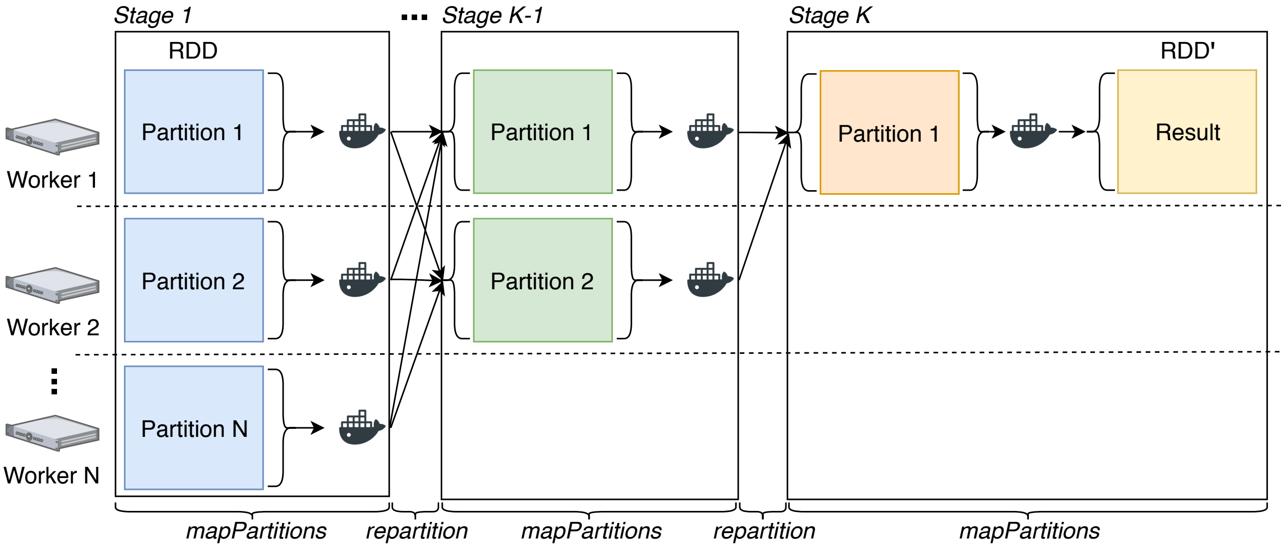

Figure 2. Execution diagram for the `reduce` primitive. The primitive takes an input RDD, partitioned over N nodes, and it iteratively aggregates records using a Docker container, reducing the number of partition until an RDD', containing a single result partition, is returned. The logic is implemented using *mapPartitions* and *repartition* from the RDD API, to aggregate records in partitions and to decrease the number of partitions respectively. Since `repartition` is called in each of the K iterations, K stages are generated, giving place to K data shuffles.

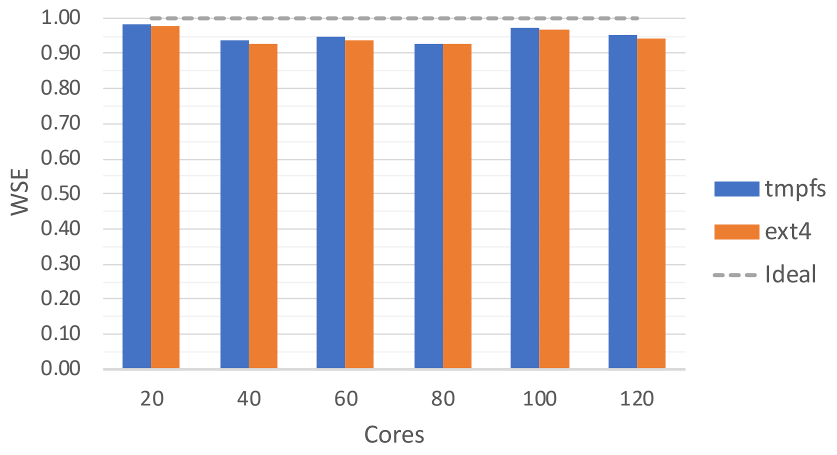

Figure 3. WSE for the VS application implemented in MaRe (listing 2). The results are produced by using SureChEMBL as input and we show the WSE when using *tmpfs* and *ext4* as temporary mount point for passing the data to the containers.

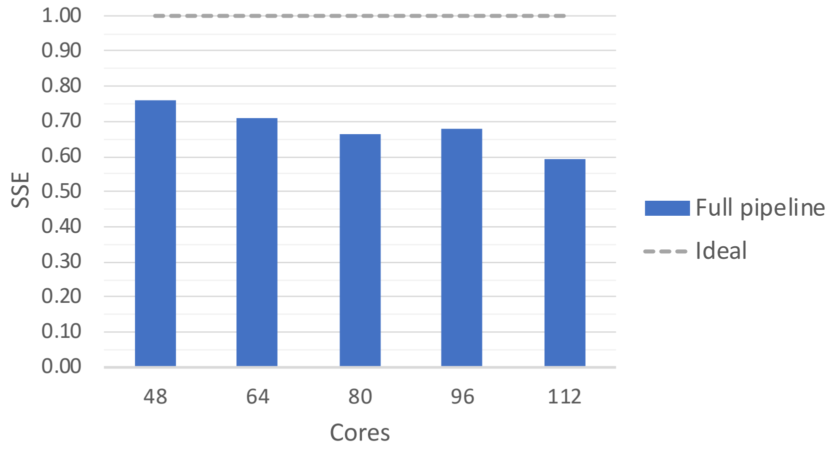

Figure 4. SSE for the SNP calling implemented in MaRe (listing 3). The results are produced by using a full individual dataset from the 1KGP as input.

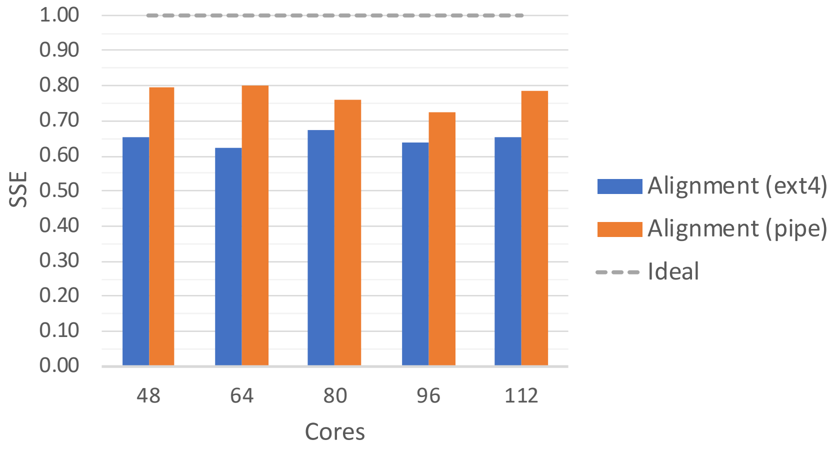

Figure 5. SSE for the SNP calling alignment stage implemented in MaRe (listing 3, lines 1 to 13). The results are produced by using a full individual dataset from the 1KGP as input and we show the SSE when using an SSD-based, *ext4* temporary mount point as well as Unix pipes for passing the data to the containers.

Figure

Click here to access/download;Figure;figur

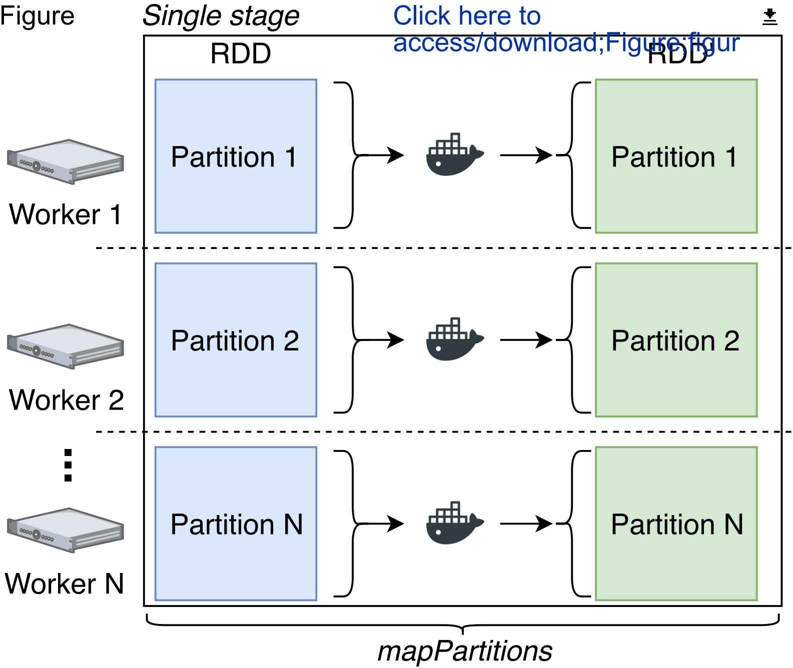

Click here to access/download;Figure;figure_2.pdf

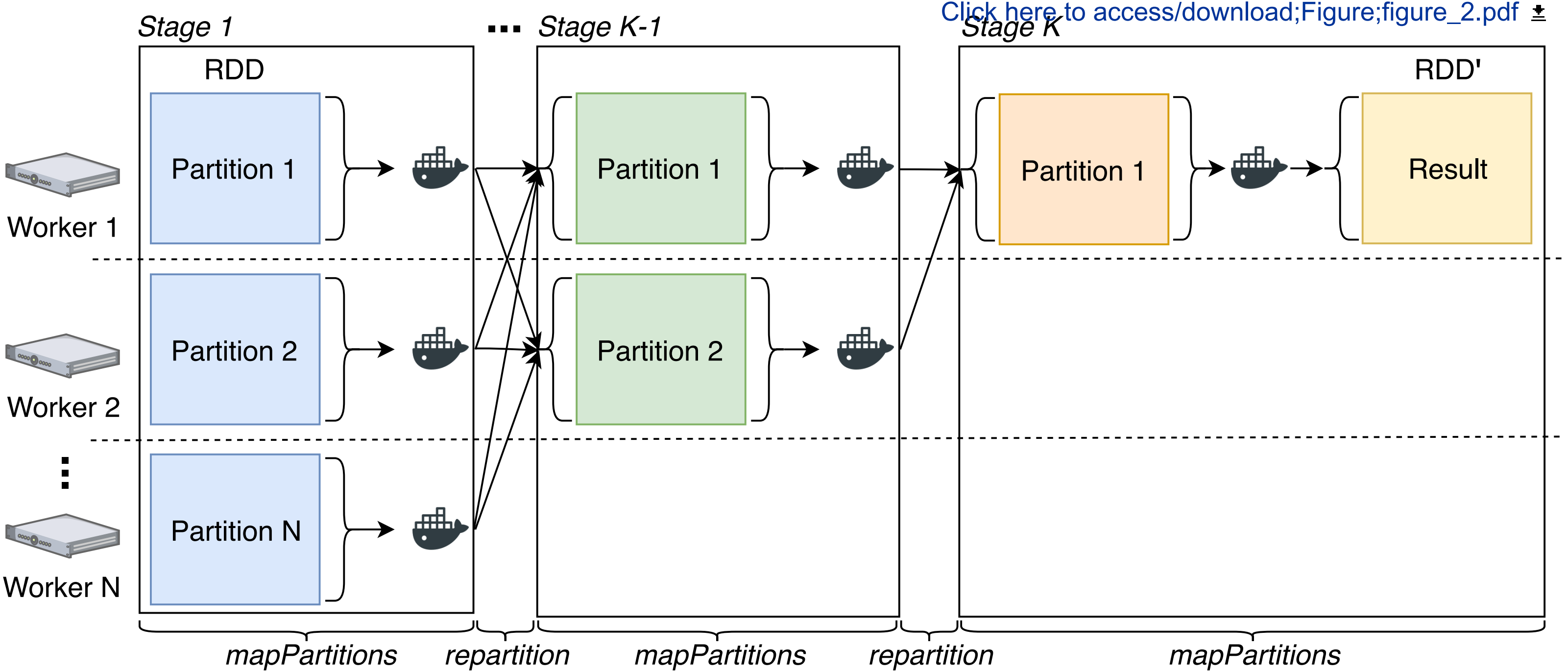

Figure

Click here to access/download;Figure;figure_3.pdf

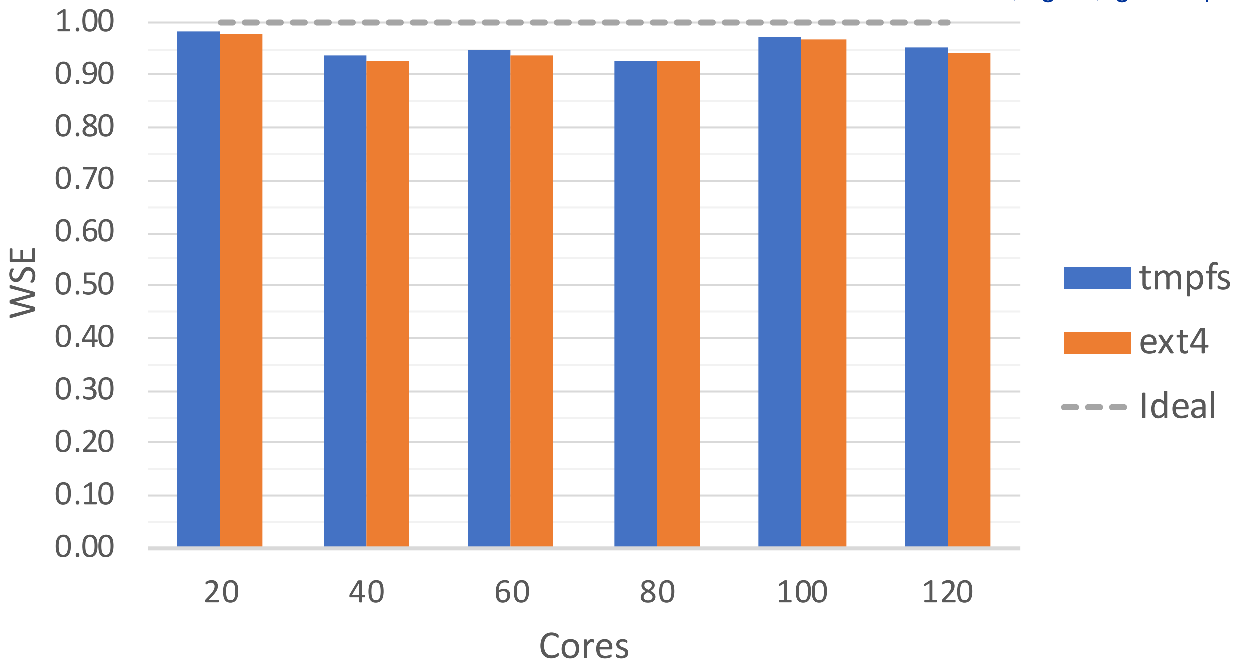

Figure

Click here to access/download;Figure;figure_4.pdf

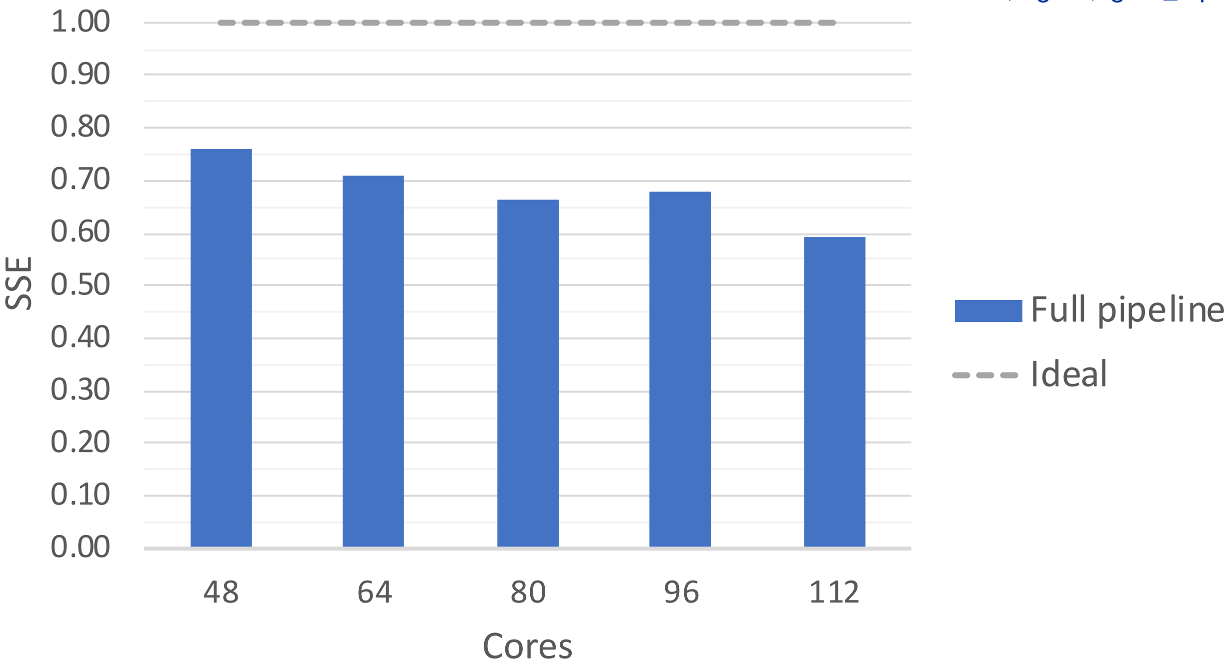

Figure

Click here to access/download;Figure;figure_5.pdf

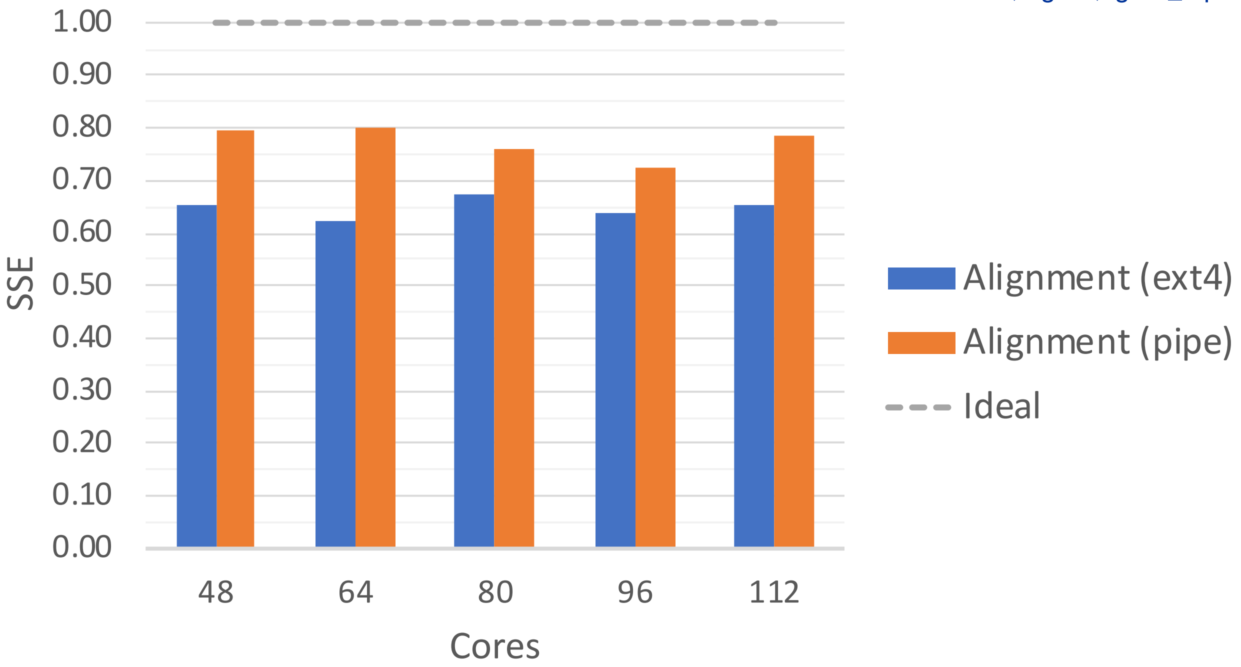

 Click here to access/download;Personal Cover;MaRe Rebuttal.pdf

Dear Editor,

Please find attached our revised manuscript "MaRe: Processing Big Data with Application Containers on Apache Spark" (GIGA-D-19-00170) submitted for publication in GigaScience.

We thank the reviewers for constructive criticism and in particular for suggesting to improve the evaluation section of our manuscript. We have now run the requested comparative experimentation where possible and included the results in the revised version. We believe that the scope and usefulness of the framework are now properly identified and discussed. In addition, we improved the sections that the reviewers found unclear.

We also have registered MaRe on SciCrunch.org and added its resource ID to the manuscript. Further, we have now specified the full URL to the 1000 genome project subset that we have utilized in the analysis.

Below we provide responses and comments to the reviewer's remarks and describe the updates we have made in the revised version of our manuscript. We hope that you now find it suitable for publication.

Sincerely,

Marco Capuccini and co-authors

Additional changes triggered by both reviewers comments

Even if not asked by the reviewers we did some minor changes to the evaluation section, which were triggered by the comparative experimentation.

First, the scaling efficiency measures in the first version of the manuscript were relative, meaning that we were computing the measures using the MaRe parallelization on a single node. When doing a comparative experimentation we instead need an absolute baseline to compute the measures, hence the scaling efficiency for both of the two use cases is now computed using the dockerized tools, built-in parallelization on a single node. We updated the evaluation section accordingly.

Second, when running the comparative experiments we were initially getting inconsistent results as the worker node flavors that we were previously using allowed overcommitted CPUs. This means that when increasing the number of nodes we did not always get the real parallelism that we were expecting. Changing the worker node flavor to one that does not allow overcommitment solved the problem. We updated the evaluation section describing the specifications of such flavor. Please note that we use slightly different node flavors in the two use cases now. In particular for the genomics use case we use a flavor with local SSD drive, which allows for materializing larger partitions faster on disk. As this kind of machines are scarce in our cloud provider, we scaled the analysis only up to 112 cores in the new version of the manuscript.

Finally, we switched the scaling efficiency metric for the genomics use case to Strong Scaling Efficiency (SSE). In the previous version of the manuscript the Weak Scaling Efficiency (WSE) was calculated without downsampling the input reference genome, thus giving a poor estimate of the metric. As there is no straightforward way of downsampling the reference genome without altering the behaviour of the tools, we kept the input data size fixed when increasing the parallelism in the new version of the study; thus computing SSE instead of WSE. We updated the evaluation section of the paper accordingly.

Reviewer #1

I found the thesis of the paper to be interesting but a bit confusing. The title of the paper says that MaRe is a MapReduce oriented framework for processing Big Data. Then, it is said during the abstract that MaRe is a (new?) programming model. However, further discussion reveals that the programming model proposed by MaRe is essentially (a subset of) the same of Spark, with the only major difference being the ability to interact with external programs in a more seamless way than using the primitives coming with Spark.

To this end, I think the authors should better clarify their contribution and, probably, put it in the right perspective. Namely, MaRe would be better presented as a software library acting as a wrapper for a Spark RDD and aiming to simplify the integration with external programs.

Thanks for pointing this out. Our initial reasoning was to see MaRe as a new programming model on top of Spark, but we agree that this can be confusing. To make our contribution more clear we updated the title of our manuscript, the abstract and the summary points in the last paragraph of the introduction.

I also think the authors missed one important point while developing their work. Ok, I am aware of the best practices encouraging the reuse of existing tools, but I would like to know if, using MaRe, I have to suffer from, let's say, a 10x slowdown with respect to a native implementation. Or, I would like to know what is the speedup achievable with respect to the usage of the standard facilities coming with Spark for running external processes. Instead, there is no evaluation of these cases. I think there are at least two solutions alternative to MaRe that should be considered in a comparative experimentation:

- the transformation to apply to a certain dataset is not delegated to an external tool, but natively implemented in Spark using the language of choice

We understand your point. However, if some native Spark implementations of the tools used in the presented benchmarks were available there would be no need to reimplement them using MaRe. We could consider such implementations as existing tools and definitely encourage using them instead of our programming library. To the best of our knowledge, the only available Spark-based implementation of virtual screening (use case 1) was presented in our previous papers [1,2] and the only Spark-based implementation of genomics pipelines (use case 2) that has reached production readiness is ADAM [3]. Made an exception for a few preprocessing steps in ADAM, both of these existing implementations delegate data processing to external tools using pipes; the data is **not** solely processed using the language of choice.

Reusing existing tools is often the case in bioinformatics data processing as the effort of reimplementing single-node tools is seldom sustainable. Convincing arguments are the bioinformatics data pipelines available in repositories such as nf-core [4]. Also, another interesting supporting fact is that the development of the Spark-native tools in the GATK suite started in 2016[1] and even if backed by the Broad Institute still failed to produce a stable release; besides we, despite quite a lot of effort, couldn't get the current beta to run on our cluster without errors. This clearly shows how much effort needs to be put in reimplementing such tools natively in Spark.

We expanded the first paragraph of the evaluation section to make our argument clear in the manuscript. Also, we now state clearly in the second last paragraph of the "discussion and conclusions" section that ADAM still relies on external tools to run real-world use cases.

- the transformation is run through an external program by using the 'pipe' facility available with Spark.

Thanks for suggesting this comparison. Testing directly against RDD pipe would not make a fair comparison because such a method starts an instance of the external tool for each RDD record, thus introducing a considerable overhead. Please notice that MaRe feeds entire RDD partitions to the containers, hence generating way less tool startup overhead. However, similarly to what it was done in ADAM [3] and in our previous virtual screening implementation [1,2], for the revised manuscript we implemented a *pipePartition* method that pipes entire RDD partitions to the external tools and ran the comparison against it, where allowed by the external tools. In the added benchmarks, the only tool that allows for inputting the data via standard input is BWA, so this comparison was possible only for the alignment portion of the second benchmark. Please refer to figure 5, and its referencing paragraph in the updated evaluation section to see the results of such comparison.

As an alternative, if the target application does not support the possibility of taking its input from the stdin, the input data is preliminarly saved in a file (e.g., using /tmpfs as MaRe does) and, then, it is used to run the external program.

As the reviewer acknowledges, copying/saving data on a preliminary file is exactly what MaRe does, so there would be no difference in performance when doing it manually in Spark. However, this would take many lines of codes, especially when implementing the *reduce* method, while MaRe makes it seamless. We believe this to be already clear in the implementation section of the manuscript.

Along this line, another point that would have required a better investigation is the choice of the solution to be used for storing temporary data to be processed by an external program. To this end, the solution chosen by the authors is to temporarily store data in memory using /tmpfs. I may be wrong, but this should mean that, at some point during the execution of an external process, the overall amount of available memory is decreased because input and/or

[1] This can be checked on GitHub https://github.com/broadinstitute/gatk.

output data is represented twice. This may have important consequences in processes where there is a high degree of parallelism and the amount of memory for executor is limited.

Thanks for pointing this out. It is true that by materializing the data on tmpfs we need twice as much memory for representing the partitions. However, please notice that Spark does not load partitions all at once, but it does it sequentially as resources become available. Since the partition size is configurable in Spark, one can tune it so that the total required memory will not exceed the available resources. Also notice that the partition size in Spark is equal to the block size in HDFS (128MB). This means that for a 8-cores machine a user would need 2GB of memory for representing the partitions twice. This is in most cases acceptable in modern data centers.

Representing data twice becomes a problem only when the user needs to aggregate large amounts of data on a single partition. This is necessary in our second benchmark, as GATK requires to see entire chromosomes at once. In such case a disk mount can be used instead of tmpfs; in our updated benchmark we used a local SSD drive.

We expanded the “data handling” section of the paper to make these points more clear.

Conversely, the choice of storing this data on a persistent storage rather than in memory would be able to overcome this problem but would severely affect the performance of a process. These issues are briefly mentioned in the 'Discussion and conclusions section', while they would have required a much deeper investigation.

Thanks for pointing this out. An experimental comparison between tmpfs and persistent storage is possible for the virtual screening use case; please recall that for the other use case the intermediate partitions are too large to fit tmpfs. Please refer to figure 1 and its referencing text for the results of such comparison.

Surprisingly, there is very little overhead introduced by writing temporary data on the persistent disk; we used a regular block storage served over the network instead of SSD to evaluate this in the most penalizing settings. The reason why little overhead is introduced is that the partitions can be copied to the persistent disk relatively fast before the docker containers are started (recall that MaRe runs the tools for entire partitions and not record-wise). Then, since the container running time dominates over the data copying time, the total cost in terms of total running time is roughly the same. We update the second paragraph of the “discussion and conclusion” section accordingly.

There are also some typos spread across the paper, such as:
- Section 'Findings'.'Background and Purposes', page 2 : 'Finally by supporting Docker,' should be 'Finally, by supporting Docker'

Fixed.

- Section 'Mare'.'Implementation', page 3: 'within each partitions' should be 'within each partition'

Fixed.

- Section 'Evaluation', page 4 : 'Amanzon' should be 'Amazon'

Fixed.

Finally, I think that the authors should put less emphasis on the possibility to ingest data from heterogeneous cloud resources as it is essentially inherited for free from Spark.

This is a good point. We removed this from the last paragraph of the introduction and we left out the benchmarks against multiple cloud storages.

Reviewer #2

This first half of this work describes MaRe, a useful addition to the toolbox for scaling genomics analysis: a relatively simple approach to distributing container-based data-intensive analysis, based on MapReduce. The authors implement the framework in a sensible fashion, taking advantage of the various benefits of Apache Spark. The framework seems reasonable and useful.

I am not convinced of the second part of the paper, which looks to evaluate MaRe using two real world applications. Admittedly it is not trivial to implement a distributive framework for generic applications that scales well, but that is sort of the point of the paper. Some specific concerns are around showing the the approach works for what is essentially a trivial distribution problem - where the data per job is small and jobs are relatively transactional and independent - but the major point of a general framework is that it is useful for more complex tasks, which the second variant calling example is.

Thanks for raising this point. Implementing a distributed framework for scaling any kind of application is out of the scope of this paper. Here, we aim at providing an alternative to workflow systems, which are broadly used in bioinformatics, that builds on top of the Apache Spark ecosystem. While scaling independent tasks is admittedly trivial, integrating application containers in Spark, such that containerized bioinformatics pipelines can easily be expressed in a few lines of code and yet scale reasonably good is not a simple problem. This is the main achievement of the presented work.

We believe this to be already clear in the current status of the paper.

I have the nagging feeling that the specifics of the evaluation task here were set to the advantage of the framework, and still the outcome was just OK. The problem, as always, is that the individual tasks are dependent on I/O, and as the authors identify, data distribution is the factor in this example that dominates the scalability.

Thanks for pointing this out. Our intention with the evaluation of our work was to show two use cases that are somewhat representative of two classes problems that one may encounter when distributing bioinformatics pipelines. The first use case matches perfectly the MapReduce approach implemented by MaRe, thus we are able to show a scaling efficiency that is close to ideal; not “just OK”. In the second use case we deliberately expose where MaRe falls short by setting up a scenario in which the MapReduce model is disadvantaged. In our perspective the fact that even for this kind of problem the analysis still scales “just OK” is a strength of our framework rather than a weakness.

We updated the first paragraph of the evaluation section to make this more clear.

This excerpt from the discussions and conclusions is telling:
"Scalability in the SNP calling analysis is reasonably good but far from optimal. The reason for this is that before running the haplotype caller, a reasonable amount of data needs to be shuffled across the nodes as GATK needs to see all of
the data for a single chromosome at once in order to function properly, thus causing a large amount of data to be materialized on disk. Such overhead can be partly mitigated by enabling data streams via standard input and output between MaRe and containers, which constitutes an area for future improvement."

In summary I think this paper needs additional work on the evaluation to identify the scope of the usefulness of the framework.; and the evaluation section itself needs to be clearer. The authors state :"It is however important to point out that while ADAM is application specific, MaRe applies to a variety of use cases in bioinformatics and it stands out by enabling distributed SNP calling in less than 50 lines of code."
If that's the case, I think the paper needs to identify and discuss the performance that can be expected across different types of use cases, and why.

Please refer to the previous point. Our intention with the two use cases is to show two classes of problems for which one can expect ideal or suboptimal performance. We updated the first paragraph of the evaluation section to make this more clear.

As a suggestion, a comparison to ADAM leading to a discussion of what the fundamental challenges of scaling I/O intensive tasks are and how that might map to different common tasks in bioinformatics, would be useful.

Thanks for suggesting a comparison with ADAM. We realized that we did not state explicitly that ADAM implements only a few preprocessing steps of the SNP pipeline [3]. Near-ideal scalability is shown in [3] only for these preprocessing steps, however in real-world settings some external tools would be needed to run a complete analysis. Indeed, ADAM provides a modified version of RDD pipes for running external tools [5], but no study has yet quantified what kind of performance one can expect when running external tools in ADAM. One major problem with pipes is that not every external tool is capable of accepting data via standard input. GATK, which provides a state of the art variant caller, is an example of such a tool. For this reason we were not able to reproduce the same pipeline that we ran for our genomics benchmark using ADAM. However, the first part of the pipeline uses a tool that

can read data via standard input (BWA). Hence, we could compare the scaling efficiency that we obtained using MaRe, for this first portion of the pipeline, with a similar implementation of the modified RDD pipe routine included in ADAM. We prefered to simply reimplement this routine as the ADAM project requires many dependencies that would be hard to bring in our environment. Figure 5, and its referencing text, present the results of this new comparison. We also added the discussed details about ADAM in the fourth paragraph of the “discussion and conclusion” section.

Or at least a discussion of the characteristics of problems that MaRe would suit.

We expanded the second last paragraph of the “discussion and conclusion” section to point out where MaRe falls short. In summary, when records in large partitions need to be processed all together it is not reasonable to expect ideal scalability, however given the effort that sometimes need to be put in reimplementing bioinformatics tools in Spark, scaling the analyses in MaRe could prove to be more sustainable.

There are also a handful of expression and grammatical errors:

"Such amounts of data poses major challenges for scientific analyses"

> such amounts of data _pose_ major challenges…

Fixed.

"but also prohibitively expensive in terms of power consumption, estimated to be in the order of several hundred thousand dollars per year"

> is this for all life science data transfer over the entire planet? The sentence needs some qualification.

Referring to the cited work, this is for a single next-generation HPC cluster. We added this detail to the sentence to make it more clear.

"In summary, the key contribution of the presented work are:"

> In summary, the key contributions of the presented work are:

Fixed.

"We demonstrate data ingestion from three large-scale storage systems: Hadoop Distributed File System (HDFS) [41], Swift [42] and Amanzon S3 [43]. In our settings HDFS was co-located with the Apache Spark"

> Amazon S3

Fixed.

"One of the advantages of Apache Spark over other MapReduce-like systems is the ability of retaining data in memory. Hence, for better performance it is preferable to keep RDD records in memory when mounting them in the containers."

> the word 'Hence' doesn't make logical sense in this sentence.

We removed the sentence starting with “Hence”.

"The full benchmark runs in ~1.8 hours when using 128 vCPUs, including data ingestion from S3."

> It's impossible to know what this means as it is given in isolation - no context and no comparison to performance after. I think this is an example of a significant flaw in the paper, in that the theoretical and implementation parts seem fine, but the evaluation is not really convincing.

This is a good point. We added some details about the running time on a single node to add some context and comparison of the statement. Please refer to the second last paragraph of the “Virtual Screening” section and to the second last paragraph of the “Single Nucleotide Polymorphism” section.

References

1. Capuccini M, Ahmed L, Schaal W, Laure E, Spjuth O. Large-scale virtual screening on public cloud resources with Apache Spark. J Cheminform. 2017;9: 15.

2. Ahmed L, Georgiev V, Capuccini M, Toor S, Schaal W, Laure E, et al. Efficient iterative virtual screening with Apache Spark and conformal prediction. J Cheminform. 2018;10: 8.

3. Nothaft FA, Linderman M, Franklin MJ, Joseph AD, Patterson DA, Massie M, et al. Rethinking Data-Intensive Science Using Scalable Analytics Systems. Proceedings of the 2015 ACM SIGMOD International Conference on Management of Data - SIGMOD ’15. 2015. doi:10.1145/2723372.2742787

4. Ewels P. nf-core. [cited 28 Jan 2020]. Available: https://nf-co.re/pipelines

5. Using ADAM’s Pipe API — bdgenomics.adam 0.23.0-SNAPSHOT documentation. [cited 29 Jan 2020]. Available: https://adam.readthedocs.io/en/latest/api/pipes/
