# Supplementary material for: MaRe: Processing Big Data with application containers on Apache Spark
Source: Gigascience. 2020 May 5;9(5):giaa042. doi: 10.1093/gigascience/giaa042 (PMC7199472; doi:10.1093/gigascience/giaa042)
Supplement: giaa042_GIGA-D-19-00170_Revision_2 [file giaa042_giga-d-19-00170_revision_2.pdf]

GigaScience

MaRe: Processing Big Data with Application Containers on Apache Spark

--Manuscript Draft--

**Manuscript Number:**	GIGA-D-19-00170R2
**Full Title:**	MaRe: Processing Big Data with Application Containers on Apache Spark
**Article Type:**	Technical Note
**Funding Information:**	The European Commission's Horizon 2020 programme (654241) — Dr. Ola Spjuth
**Abstract:**	Background. Life science is increasingly driven by Big Data analytics, and the MapReduce programming model has been proven successful for data-intensive analyses. However, current MapReduce frameworks offer poor support for reusing existing processing tools in bioinformatics pipelines. Further, these frameworks do not have native support for application containers, which are becoming popular in scientific data processing. Results. Here we present MaRe, an open-source programming library which introduces support for Docker containers in Apache Spark. Apache Spark and Docker are the MapReduce framework and container engine that have collected the largest open source community, thus MaRe provides interoperability with the cutting-edge software ecosystem. We demonstrate MaRe on two data-intensive applications in life science, showing ease of use and scalability. Conclusions. MaRe enables scalable data-intensive processing in life science with Apache Spark and application containers. When compared with current best practices, that involve the use of workflow systems, MaRe has the advantage of providing data locality, ingestion from heterogeneous storage systems and interactive processing. MaRe is generally-applicable and available as open source software.
**Corresponding Author:**	Marco Capuccini<br>Uppsala Universitet<br>Uppsala, SWEDEN
**Corresponding Author Secondary Information:**	
**Corresponding Author's Institution:**	Uppsala Universitet
**Corresponding Author's Secondary Institution:**	
**First Author:**	Marco Capuccini
**First Author Secondary Information:**	
**Order of Authors:**	Marco Capuccini
	Martin Dahlö
	Salman Toor
	Ola Spjuth
**Order of Authors Secondary Information:**	

Powered by Editorial Manager® and ProduXion Manager® from Aries Systems Corporation

**Response to Reviewers:**	Dear Editor,<br><br>Please find attached our revised manuscript "MaRe: Processing Big Data with Application Containers on Apache Spark" (GIGA-D-19-00170) submitted for publication in GigaScience.<br><br>We are pleased with the reviewer's positive outlook.<br><br>Below we provide responses to the remaining issues pointed out by the editor and describe the updates we have made in the revised version of our manuscript. We hope that you now find it suitable for publication.<br><br>Sincerely,<br><br>Marco Capuccini and co-authors<br><br>> Prior to publication, please include a citation to your upcoming GigaDB dataset (including the DOI link) to your reference list, and cite this by number in the data availability section<br><br>We added the citation to the GigaDB dataset in the data availability section.<br><br>> In the data availability section, please also include the relevant citation (and BioProject accession number(s)) for the 1000 Genomes data<br><br>We added the citation to the 1KGP publication and its BioProject accession number in the data availability section.<br><br>> please also consider to mention details on exactly which data from ZINC was used, to help reproducibility<br><br>We added the URL to the specific ZINC subset that we used in the data availability section.
**Additional Information:**	
**Question**	**Response**
Are you submitting this manuscript to a special series or article collection?	No
**Experimental design and statistics**<br><br>Full details of the experimental design and statistical methods used should be given in the Methods section, as detailed in our Minimum Standards Reporting Checklist. Information essential to interpreting the data presented should be made available in the figure legends.<br><br>Have you included all the information requested in your manuscript?	Yes

Powered by Editorial Manager® and ProduXion Manager® from Aries Systems Corporation

**Resources**<br><br>A description of all resources used, including antibodies, cell lines, animals and software tools, with enough information to allow them to be uniquely identified, should be included in the Methods section. Authors are strongly encouraged to cite Research Resource Identifiers (RRIDs) for antibodies, model organisms and tools, where possible.<br><br>Have you included the information requested as detailed in our Minimum Standards Reporting Checklist?	No
If not, please give reasons for any omissions below.<br><br>as follow-up to "**Resources**<br><br>A description of all resources used, including antibodies, cell lines, animals and software tools, with enough information to allow them to be uniquely identified, should be included in the Methods section. Authors are strongly encouraged to cite Research Resource Identifiers (RRIDs) for antibodies, model organisms and tools, where possible.<br><br>Have you included the information requested as detailed in our Minimum Standards Reporting Checklist?<br><br>"	In this work we use public data from the 1000 genome project and from the ZINC molecular database. Relevant details are explained in the referenced publications.
**Availability of data and materials**<br><br>All datasets and code on which the conclusions of the paper rely must be either included in your submission or deposited in publicly available repositories (where available and ethically appropriate), referencing such data using a unique identifier in the references and in the "Availability of Data and Materials" section of your manuscript.<br><br>Have you have met the above requirement as detailed in our Minimum Standards Reporting Checklist?	Yes

Powered by Editorial Manager® and ProduXion Manager® from Aries Systems Corporation

Click here to access/download;LaTeX - Main Document (TeX file);main.tex

```
This is pdfTeX, Version 3.14159265-2.6-1.40.19 (TeX Live 2018/W32TeX)
(preloaded format=pdflatex 2018.7.12)  1 APR 2020 10:15
entering extended mode
 restricted \write18 enabled.
 %&-line parsing enabled.
**main.tex
(./main.tex
LaTeX2e <2018-04-01> patch level 5
(./oup-contemporary.cls
Document Class: oup-contemporary 2017/06/28, v1.1
(c:/TeXLive/2018/texmf-dist/tex/latex/base/article.cls
Document Class: article 2014/09/29 v1.4h Standard LaTeX document class
(c:/TeXLive/2018/texmf-dist/tex/latex/base/size10.clo
File: size10.clo 2014/09/29 v1.4h Standard LaTeX file (size option)
)
\c@part=\count80
\c@section=\count81
\c@subsection=\count82
\c@subsubsection=\count83
\c@paragraph=\count84
\c@subparagraph=\count85
\c@figure=\count86
\c@table=\count87
\abovecaptionskip=\skip41
\belowcaptionskip=\skip42
\bibindent=\dimen102
) (c:/TeXLive/2018/texmf-dist/tex/latex/base/inputenc.sty
Package: inputenc 2018/04/06 v1.3b Input encoding file
\inpenc@prehook=\toks14
\inpenc@posthook=\toks15
) (c:/TeXLive/2018/texmf-dist/tex/latex/base/fontenc.sty
Package: fontenc 2017/04/05 v2.0i Standard LaTeX package
(c:/TeXLive/2018/texmf-dist/tex/latex/base/t1enc.def
File: t1enc.def 2017/04/05 v2.0i Standard LaTeX file
LaTeX Font Info:    Redeclaring font encoding T1 on input line 48.
)) (c:/TeXLive/2018/texmf-dist/tex/generic/oberdiek/ifpdf.sty
Package: ifpdf 2017/03/15 v3.2 Provides the ifpdf switch
) (c:/TeXLive/2018/texmf-dist/tex/latex/microtype/microtype.sty
Package: microtype 2018/01/14 v2.7a Micro-typographical refinements (RS)
(c:/TeXLive/2018/texmf-dist/tex/latex/graphics/keyval.sty
Package: keyval 2014/10/28 v1.15 key=value parser (DPC)
\KV@toks@=\toks16
)
\MT@toks=\toks17
\MT@count=\count88
LaTeX Info: Redefining \textls on input line 793.
\MT@outer@kern=\dimen103
LaTeX Info: Redefining \textmicrotypecontext on input line 1339.
\MT@listname@count=\count89
(c:/TeXLive/2018/texmf-dist/tex/latex/microtype/microtype-pdftex.def
File: microtype-pdftex.def 2018/01/14 v2.7a Definitions specific to
pdftex (RS)

LaTeX Info: Redefining \lsstyle on input line 913.
```

```
LaTeX Info: Redefining \lslig on input line 913.
\MT@outer@space=\skip43
)
Package microtype Info: Loading configuration file microtype.cfg.
(c:/TeXLive/2018/texmf-dist/tex/latex/microtype/microtype.cfg
File: microtype.cfg 2018/01/14 v2.7a microtype main configuration file
(RS)
)) (c:/TeXLive/2018/texmf-dist/tex/latex/euler/euler.sty
Package: euler 1995/03/05 v2.5
Package: `euler' v2.5 <1995/03/05> (FJ and FMi)
LaTeX Font Info:    Redeclaring symbol font `letters' on input line 35.
LaTeX Font Info:    Encoding `OML' has changed to `U' for symbol font
(Font)              `letters' in the math version `normal' on input line
35.
LaTeX Font Info:    Overwriting symbol font `letters' in version `normal'
(Font)                  OML/cmm/m/it --> U/eur/m/n on input line 35.
LaTeX Font Info:    Encoding `OML' has changed to `U' for symbol font
(Font)              `letters' in the math version `bold' on input line
35.
LaTeX Font Info:    Overwriting symbol font `letters' in version `bold'
(Font)                  OML/cmm/b/it --> U/eur/m/n on input line 35.
LaTeX Font Info:    Overwriting symbol font `letters' in version `bold'
(Font)                  U/eur/m/n --> U/eur/b/n on input line 36.
LaTeX Font Info:    Redeclaring math symbol \Gamma on input line 47.
LaTeX Font Info:    Redeclaring math symbol \Delta on input line 48.
LaTeX Font Info:    Redeclaring math symbol \Theta on input line 49.
LaTeX Font Info:    Redeclaring math symbol \Lambda on input line 50.
LaTeX Font Info:    Redeclaring math symbol \Xi on input line 51.
LaTeX Font Info:    Redeclaring math symbol \Pi on input line 52.
LaTeX Font Info:    Redeclaring math symbol \Sigma on input line 53.
LaTeX Font Info:    Redeclaring math symbol \Upsilon on input line 54.
LaTeX Font Info:    Redeclaring math symbol \Phi on input line 55.
LaTeX Font Info:    Redeclaring math symbol \Psi on input line 56.
LaTeX Font Info:    Redeclaring math symbol \Omega on input line 57.
\symEulerFraktur=\mathgroup4
LaTeX Font Info:    Overwriting symbol font `EulerFraktur' in version
`bold'
(Font)                  U/euf/m/n --> U/euf/b/n on input line 63.
LaTeX Info: Redefining \oldstylenums on input line 85.
\symEulerScript=\mathgroup5
LaTeX Font Info:    Overwriting symbol font `EulerScript' in version
`bold'
(Font)                  U/eus/m/n --> U/eus/b/n on input line 93.
LaTeX Font Info:    Redeclaring math symbol \aleph on input line 97.
LaTeX Font Info:    Redeclaring math symbol \Re on input line 98.
LaTeX Font Info:    Redeclaring math symbol \Im on input line 99.
LaTeX Font Info:    Redeclaring math delimiter \vert on input line 101.
LaTeX Font Info:    Redeclaring math delimiter \backslash on input line
103.
LaTeX Font Info:    Redeclaring math symbol \neg on input line 106.
LaTeX Font Info:    Redeclaring math symbol \wedge on input line 108.
LaTeX Font Info:    Redeclaring math symbol \vee on input line 110.
LaTeX Font Info:    Redeclaring math symbol \setminus on input line 112.
LaTeX Font Info:    Redeclaring math symbol \sim on input line 113.
```

```
LaTeX Font Info:    Redeclaring math symbol \mid on input line 114.
LaTeX Font Info:    Redeclaring math delimiter \arrowvert on input line
116.
LaTeX Font Info:    Redeclaring math symbol \mathsection on input line
117.
\symEulerExtension=\mathgroup6
LaTeX Font Info:    Redeclaring math symbol \coprod on input line 125.
LaTeX Font Info:    Redeclaring math symbol \prod on input line 125.
LaTeX Font Info:    Redeclaring math symbol \sum on input line 125.
LaTeX Font Info:    Redeclaring math symbol \intop on input line 130.
LaTeX Font Info:    Redeclaring math symbol \ointop on input line 131.
LaTeX Font Info:    Redeclaring math symbol \braceld on input line 132.
LaTeX Font Info:    Redeclaring math symbol \bracerd on input line 133.
LaTeX Font Info:    Redeclaring math symbol \bracelu on input line 134.
LaTeX Font Info:    Redeclaring math symbol \braceru on input line 135.
LaTeX Font Info:    Redeclaring math symbol \infty on input line 136.
LaTeX Font Info:    Redeclaring math symbol \nearrow on input line 153.
LaTeX Font Info:    Redeclaring math symbol \searrow on input line 154.
LaTeX Font Info:    Redeclaring math symbol \nwarrow on input line 155.
LaTeX Font Info:    Redeclaring math symbol \swarrow on input line 156.
LaTeX Font Info:    Redeclaring math symbol \Leftrightarrow on input line
157.
LaTeX Font Info:    Redeclaring math symbol \Leftarrow on input line 158.
LaTeX Font Info:    Redeclaring math symbol \Rightarrow on input line
159.
LaTeX Font Info:    Redeclaring math symbol \leftrightarrow on input line
160.
LaTeX Font Info:    Redeclaring math symbol \leftarrow on input line 161.
LaTeX Font Info:    Redeclaring math symbol \rightarrow on input line
163.
LaTeX Font Info:    Redeclaring math delimiter \uparrow on input line
166.
LaTeX Font Info:    Redeclaring math delimiter \downarrow on input line
168.
LaTeX Font Info:    Redeclaring math delimiter \updownarrow on input line
170.
LaTeX Font Info:    Redeclaring math delimiter \Uparrow on input line
172.
LaTeX Font Info:    Redeclaring math delimiter \Downarrow on input line
174.
LaTeX Font Info:    Redeclaring math delimiter \Updownarrow on input line
176.
LaTeX Font Info:    Redeclaring math symbol \leftharpoonup on input line
177.
LaTeX Font Info:    Redeclaring math symbol \leftharpoondown on input
line 178.

LaTeX Font Info:    Redeclaring math symbol \rightharpoonup on input line
179.
LaTeX Font Info:    Redeclaring math symbol \rightharpoondown on input
line 180
.
LaTeX Font Info:    Redeclaring math delimiter \lbrace on input line 182.
LaTeX Font Info:    Redeclaring math delimiter \rbrace on input line 184.
```

```
\symcmmigroup=\mathgroup7
LaTeX Font Info:    Overwriting symbol font `cmmigroup' in version `bold'
(Font)                  OML/cmm/m/it --> OML/cmm/b/it on input line 200.
LaTeX Font Info:    Redeclaring math accent \vec on input line 201.
LaTeX Font Info:    Redeclaring math symbol \triangleleft on input line
202.
LaTeX Font Info:    Redeclaring math symbol \triangleright on input line
203.
LaTeX Font Info:    Redeclaring math symbol \star on input line 204.
LaTeX Font Info:    Redeclaring math symbol \lhook on input line 205.
LaTeX Font Info:    Redeclaring math symbol \rhook on input line 206.
LaTeX Font Info:    Redeclaring math symbol \flat on input line 207.
LaTeX Font Info:    Redeclaring math symbol \natural on input line 208.
LaTeX Font Info:    Redeclaring math symbol \sharp on input line 209.
LaTeX Font Info:    Redeclaring math symbol \smile on input line 210.
LaTeX Font Info:    Redeclaring math symbol \frown on input line 211.
LaTeX Font Info:    Redeclaring math accent \grave on input line 245.
LaTeX Font Info:    Redeclaring math accent \acute on input line 246.
LaTeX Font Info:    Redeclaring math accent \tilde on input line 247.
LaTeX Font Info:    Redeclaring math accent \ddot on input line 248.
LaTeX Font Info:    Redeclaring math accent \check on input line 249.
LaTeX Font Info:    Redeclaring math accent \breve on input line 250.
LaTeX Font Info:    Redeclaring math accent \bar on input line 251.
LaTeX Font Info:    Redeclaring math accent \dot on input line 252.
LaTeX Font Info:    Redeclaring math accent \hat on input line 254.
) (c:/TeXLive/2018/texmf-dist/tex/latex/merriweather/merriweather.sty
Package: merriweather 2014/01/22 (Bob Tennent) Supports
Merriweather(Sans) font
s for all LaTeX engines.
(c:/TeXLive/2018/texmf-dist/tex/generic/ifxetex/ifxetex.sty
Package: ifxetex 2010/09/12 v0.6 Provides ifxetex conditional
) (c:/TeXLive/2018/texmf-dist/tex/generic/oberdiek/ifluatex.sty
Package: ifluatex 2016/05/16 v1.4 Provides the ifluatex switch (HO)
Package ifluatex Info: LuaTeX not detected.
) (c:/TeXLive/2018/texmf-dist/tex/latex/base/textcomp.sty
Package: textcomp 2017/04/05 v2.0i Standard LaTeX package
Package textcomp Info: Sub-encoding information:
(textcomp)               5 = only ISO-Adobe without \textcurrency
(textcomp)               4 = 5 + \texteuro
(textcomp)               3 = 4 + \textohm
(textcomp)               2 = 3 + \textestimated + \textcurrency
(textcomp)               1 = TS1 - \textcircled - \t
(textcomp)               0 = TS1 (full)
(textcomp)             Font families with sub-encoding setting implement
(textcomp)             only a restricted character set as indicated.
(textcomp)             Family '?' is the default used for unknown fonts.
(textcomp)             See the documentation for details.
Package textcomp Info: Setting ? sub-encoding to TS1/1 on input line 79.
(c:/TeXLive/2018/texmf-dist/tex/latex/base/ts1enc.def
File: ts1enc.def 2001/06/05 v3.0e (jk/car/fm) Standard LaTeX file
Now handling font encoding TS1 ...
... processing UTF-8 mapping file for font encoding TS1
(c:/TeXLive/2018/texmf-dist/tex/latex/base/ts1enc.dfu
File: ts1enc.dfu 2018/04/05 v1.2c UTF-8 support for inputenc
```

```
defining Unicode char U+00A2 (decimal 162)
defining Unicode char U+00A3 (decimal 163)
defining Unicode char U+00A4 (decimal 164)
defining Unicode char U+00A5 (decimal 165)
defining Unicode char U+00A6 (decimal 166)
defining Unicode char U+00A7 (decimal 167)
defining Unicode char U+00A8 (decimal 168)
defining Unicode char U+00A9 (decimal 169)
defining Unicode char U+00AA (decimal 170)
defining Unicode char U+00AC (decimal 172)
defining Unicode char U+00AE (decimal 174)
defining Unicode char U+00AF (decimal 175)
defining Unicode char U+00B0 (decimal 176)
defining Unicode char U+00B1 (decimal 177)
defining Unicode char U+00B2 (decimal 178)
defining Unicode char U+00B3 (decimal 179)
defining Unicode char U+00B4 (decimal 180)
defining Unicode char U+00B5 (decimal 181)
defining Unicode char U+00B6 (decimal 182)
defining Unicode char U+00B7 (decimal 183)
defining Unicode char U+00B9 (decimal 185)
defining Unicode char U+00BA (decimal 186)
defining Unicode char U+00BC (decimal 188)
defining Unicode char U+00BD (decimal 189)
defining Unicode char U+00BE (decimal 190)
defining Unicode char U+00D7 (decimal 215)
defining Unicode char U+00F7 (decimal 247)
defining Unicode char U+0192 (decimal 402)
defining Unicode char U+02C7 (decimal 711)
defining Unicode char U+02D8 (decimal 728)
defining Unicode char U+02DD (decimal 733)
defining Unicode char U+0E3F (decimal 3647)
defining Unicode char U+2016 (decimal 8214)
defining Unicode char U+2020 (decimal 8224)
defining Unicode char U+2021 (decimal 8225)
defining Unicode char U+2022 (decimal 8226)
defining Unicode char U+2030 (decimal 8240)
defining Unicode char U+2031 (decimal 8241)
defining Unicode char U+203B (decimal 8251)
defining Unicode char U+203D (decimal 8253)
defining Unicode char U+2044 (decimal 8260)
defining Unicode char U+204E (decimal 8270)
defining Unicode char U+2052 (decimal 8274)
defining Unicode char U+20A1 (decimal 8353)
defining Unicode char U+20A4 (decimal 8356)
defining Unicode char U+20A6 (decimal 8358)
defining Unicode char U+20A9 (decimal 8361)
defining Unicode char U+20AB (decimal 8363)
defining Unicode char U+20AC (decimal 8364)
defining Unicode char U+20B1 (decimal 8369)
defining Unicode char U+2103 (decimal 8451)
defining Unicode char U+2116 (decimal 8470)
defining Unicode char U+2117 (decimal 8471)
defining Unicode char U+211E (decimal 8478)
```

```
   defining Unicode char U+2120 (decimal 8480)
   defining Unicode char U+2122 (decimal 8482)
   defining Unicode char U+2126 (decimal 8486)
   defining Unicode char U+2127 (decimal 8487)
   defining Unicode char U+212E (decimal 8494)
   defining Unicode char U+2190 (decimal 8592)
   defining Unicode char U+2191 (decimal 8593)
   defining Unicode char U+2192 (decimal 8594)
   defining Unicode char U+2193 (decimal 8595)
   defining Unicode char U+2329 (decimal 9001)
   defining Unicode char U+232A (decimal 9002)
   defining Unicode char U+2422 (decimal 9250)
   defining Unicode char U+25E6 (decimal 9702)
   defining Unicode char U+25EF (decimal 9711)
   defining Unicode char U+266A (decimal 9834)
   defining Unicode char U+FEFF (decimal 65279)
))
LaTeX Info: Redefining \oldstylenums on input line 334.
Package textcomp Info: Setting cmr sub-encoding to TS1/0 on input line
349.
Package textcomp Info: Setting cmss sub-encoding to TS1/0 on input line
350.
Package textcomp Info: Setting cmtt sub-encoding to TS1/0 on input line
351.
Package textcomp Info: Setting cmvtt sub-encoding to TS1/0 on input line
352.
Package textcomp Info: Setting cmbr sub-encoding to TS1/0 on input line
353.
Package textcomp Info: Setting cmtl sub-encoding to TS1/0 on input line
354.
Package textcomp Info: Setting ccr sub-encoding to TS1/0 on input line
355.
Package textcomp Info: Setting ptm sub-encoding to TS1/4 on input line
356.
Package textcomp Info: Setting pcr sub-encoding to TS1/4 on input line
357.
Package textcomp Info: Setting phv sub-encoding to TS1/4 on input line
358.
Package textcomp Info: Setting ppl sub-encoding to TS1/3 on input line
359.
Package textcomp Info: Setting pag sub-encoding to TS1/4 on input line
360.
Package textcomp Info: Setting pbk sub-encoding to TS1/4 on input line
361.
Package textcomp Info: Setting pnc sub-encoding to TS1/4 on input line
362.
Package textcomp Info: Setting pzc sub-encoding to TS1/4 on input line
363.
Package textcomp Info: Setting bch sub-encoding to TS1/4 on input line
364.
Package textcomp Info: Setting put sub-encoding to TS1/5 on input line
365.
Package textcomp Info: Setting uag sub-encoding to TS1/5 on input line
366.
```

Package textcomp Info: Setting ugq sub-encoding to TS1/5 on input line 367.
Package textcomp Info: Setting ul8 sub-encoding to TS1/4 on input line 368.
Package textcomp Info: Setting ul9 sub-encoding to TS1/4 on input line 369.
Package textcomp Info: Setting augie sub-encoding to TS1/5 on input line 370.
Package textcomp Info: Setting dayrom sub-encoding to TS1/3 on input line 371.
Package textcomp Info: Setting dayroms sub-encoding to TS1/3 on input line 372.

Package textcomp Info: Setting pxr sub-encoding to TS1/0 on input line 373.
Package textcomp Info: Setting pxss sub-encoding to TS1/0 on input line 374.
Package textcomp Info: Setting pxtt sub-encoding to TS1/0 on input line 375.
Package textcomp Info: Setting txr sub-encoding to TS1/0 on input line 376.
Package textcomp Info: Setting txss sub-encoding to TS1/0 on input line 377.
Package textcomp Info: Setting txtt sub-encoding to TS1/0 on input line 378.
Package textcomp Info: Setting lmr sub-encoding to TS1/0 on input line 379.
Package textcomp Info: Setting lmdh sub-encoding to TS1/0 on input line 380.
Package textcomp Info: Setting lmss sub-encoding to TS1/0 on input line 381.
Package textcomp Info: Setting lmssq sub-encoding to TS1/0 on input line 382.
Package textcomp Info: Setting lmvtt sub-encoding to TS1/0 on input line 383.
Package textcomp Info: Setting lmtt sub-encoding to TS1/0 on input line 384.
Package textcomp Info: Setting qhv sub-encoding to TS1/0 on input line 385.
Package textcomp Info: Setting qag sub-encoding to TS1/0 on input line 386.
Package textcomp Info: Setting qbk sub-encoding to TS1/0 on input line 387.
Package textcomp Info: Setting qcr sub-encoding to TS1/0 on input line 388.
Package textcomp Info: Setting qcs sub-encoding to TS1/0 on input line 389.
Package textcomp Info: Setting qpl sub-encoding to TS1/0 on input line 390.
Package textcomp Info: Setting qtm sub-encoding to TS1/0 on input line 391.
Package textcomp Info: Setting qzc sub-encoding to TS1/0 on input line 392.

```
Package textcomp Info: Setting qhvc sub-encoding to TS1/0 on input line
393.
Package textcomp Info: Setting futs sub-encoding to TS1/4 on input line
394.
Package textcomp Info: Setting futx sub-encoding to TS1/4 on input line
395.
Package textcomp Info: Setting futj sub-encoding to TS1/4 on input line
396.
Package textcomp Info: Setting hlh sub-encoding to TS1/3 on input line
397.
Package textcomp Info: Setting hls sub-encoding to TS1/3 on input line
398.
Package textcomp Info: Setting hlst sub-encoding to TS1/3 on input line
399.
Package textcomp Info: Setting hlct sub-encoding to TS1/5 on input line
400.
Package textcomp Info: Setting hlx sub-encoding to TS1/5 on input line
401.
Package textcomp Info: Setting hlce sub-encoding to TS1/5 on input line
402.
Package textcomp Info: Setting hlcn sub-encoding to TS1/5 on input line
403.
Package textcomp Info: Setting hlcw sub-encoding to TS1/5 on input line
404.
Package textcomp Info: Setting hlcf sub-encoding to TS1/5 on input line
405.
Package textcomp Info: Setting pplx sub-encoding to TS1/3 on input line
406.
Package textcomp Info: Setting pplj sub-encoding to TS1/3 on input line
407.
Package textcomp Info: Setting ptmx sub-encoding to TS1/4 on input line
408.
Package textcomp Info: Setting ptmj sub-encoding to TS1/4 on input line
409.
) (c:/TeXLive/2018/texmf-dist/tex/latex/xkeyval/xkeyval.sty
Package: xkeyval 2014/12/03 v2.7a package option processing (HA)
(c:/TeXLive/2018/texmf-dist/tex/generic/xkeyval/xkeyval.tex
(c:/TeXLive/2018/te
xmf-dist/tex/generic/xkeyval/xkvutils.tex
\XKV@toks=\toks18
\XKV@tempa@toks=\toks19
)
\XKV@depth=\count90
File: xkeyval.tex 2014/12/03 v2.7a key=value parser (HA)
)) (c:/TeXLive/2018/texmf-dist/tex/latex/base/fontenc.sty
Package: fontenc 2017/04/05 v2.0i Standard LaTeX package
) (c:/TeXLive/2018/texmf-dist/tex/latex/fontaxes/fontaxes.sty
Package: fontaxes 2014/03/23 v1.0d Font selection axes
LaTeX Info: Redefining \upshape on input line 29.
LaTeX Info: Redefining \itshape on input line 31.
LaTeX Info: Redefining \slshape on input line 33.
LaTeX Info: Redefining \scshape on input line 37.
) (c:/TeXLive/2018/texmf-dist/tex/latex/mweights/mweights.sty
```

```
Package: mweights 2017/03/30 (Bob Tennent) Support package for multiple-
weight
font packages.
LaTeX Info: Redefining \bfseries on input line 22.
LaTeX Info: Redefining \mdseries on input line 30.
LaTeX Info: Redefining \rmfamily on input line 38.
LaTeX Info: Redefining \sffamily on input line 66.
LaTeX Info: Redefining \ttfamily on input line 94.
)) (c:/TeXLive/2018/texmf-dist/tex/latex/mathastext/mathastext.sty
Package: mathastext 2016/11/06 v1.3r Use the text font in math mode (JFB)
\mst@exists@muskip=\muskip10
\mst@forall@muskip=\muskip11
\mst@prime@muskip=\muskip12
\mst@do@nonletters=\toks20
\mst@do@easynonletters=\toks21
\mst@do@az=\toks22
\mst@do@AZ=\toks23
\symmtoperatorfont=\mathgroup8
\symmtletterfont=\mathgroup9
LaTeX Font Info:    Overwriting math alphabet `\mathnormalbold' in
version `nor
mal'
(Font)                  T1/Merriweather-OsF/bx/it --> T1/Merriweather-
OsF/bx/it
 on input line 1102.
LaTeX Font Info:    Overwriting math alphabet `\mathnormalbold' in
version `bol
d'
(Font)                  T1/Merriweather-OsF/bx/it --> T1/Merriweather-
OsF/bx/it
 on input line 1102.
LaTeX Font Info:    Overwriting symbol font `mtletterfont' in version
`normal'
(Font)                  T1/Merriweather-OsF/m/it --> T1/Merriweather-
OsF/m/it o
n input line 1102.
LaTeX Font Info:    Overwriting symbol font `mtletterfont' in version
`bold'
(Font)                  T1/Merriweather-OsF/m/it --> T1/Merriweather-
OsF/bx/it
on input line 1102.
LaTeX Font Info:    Overwriting symbol font `mtoperatorfont' in version
`normal
'
(Font)                  T1/Merriweather-OsF/m/n --> T1/Merriweather-
OsF/m/n on
input line 1102.
LaTeX Font Info:    Overwriting symbol font `mtoperatorfont' in version
`bold'
(Font)                  T1/Merriweather-OsF/m/n --> T1/Merriweather-
OsF/bx/n on
 input line 1102.
LaTeX Font Info:    Overwriting math alphabet `\Mathbf' in version
`normal'
```

```
(Font)                  T1/Merriweather-OsF/bx/n --> T1/Merriweather-
OsF/bx/n o
n input line 1102.
LaTeX Font Info:    Overwriting math alphabet `\Mathbf' in version `bold'
(Font)                  T1/Merriweather-OsF/bx/n --> T1/Merriweather-
OsF/bx/n o
n input line 1102.
LaTeX Font Info:    Overwriting math alphabet `\Mathit' in version
`normal'
(Font)                  T1/Merriweather-OsF/m/it --> T1/Merriweather-
OsF/m/it o
n input line 1102.
LaTeX Font Info:    Overwriting math alphabet `\Mathit' in version `bold'
(Font)                  T1/Merriweather-OsF/m/it --> T1/Merriweather-
OsF/bx/it
on input line 1102.
LaTeX Font Info:    Overwriting math alphabet `\Mathsf' in version
`normal'
(Font)                  T1/MerriweatherSans-TLF/m/n -->
T1/MerriweatherSans-TLF
/m/n on input line 1102.
LaTeX Font Info:    Overwriting math alphabet `\Mathsf' in version `bold'
(Font)                  T1/MerriweatherSans-TLF/m/n -->
T1/MerriweatherSans-TLF
/bx/n on input line 1102.
LaTeX Font Info:    Overwriting math alphabet `\Mathtt' in version
`normal'
(Font)                  T1/lmtt/m/n --> T1/lmtt/m/n on input line 1102.
LaTeX Font Info:    Overwriting math alphabet `\Mathtt' in version `bold'
(Font)                  T1/lmtt/m/n --> T1/lmtt/bx/n on input line 1102.
** Latin letters in the normal (resp. bold) math versions are now
** set up to use the fonts T1/Merriweather-OsF/m(bx)/it
** Other characters (digits, ...) and \log-like names will be
** typeset with the n shape.
** ! and ?
** punctuation: , . : ; and \colon
LaTeX Info: Redefining \relbar on input line 1248.
** minus as endash
** \hbar
** + and =
LaTeX Info: Redefining \Relbar on input line 1350.
** adding = ; and + to \nfss@catcodes
** parentheses ( ) [ ] and slash /
** alldelims: < > \backslash \setminus | \vert \mid \{ and \}
LaTeX Font Info:    Redeclaring math delimiter \backslash on input line
1396.
LaTeX Font Info:    Redeclaring math symbol \setminus on input line 1408.
LaTeX Info: Redefining \models on input line 1417.
** \# \mathdollar \% \&
** \i and \j
LaTeX Info: Redefining \i on input line 1988.
LaTeX Info: Redefining \j on input line 1989.
** \HUGE has been (re)-defined.
** mathastext has declared larger sizes for subscripts.
```

```
** To keep LaTeX defaults, use option `defaultmathsizes'.
) (c:/TeXLive/2018/texmf-dist/tex/latex/relsize/relsize.sty
Package: relsize 2013/03/29 ver 4.1
) (c:/TeXLive/2018/texmf-dist/tex/latex/ms/ragged2e.sty
Package: ragged2e 2009/05/21 v2.1 ragged2e Package (MS)
(c:/TeXLive/2018/texmf-dist/tex/latex/ms/everysel.sty
Package: everysel 2011/10/28 v1.2 EverySelectfont Package (MS)
)
\CenteringLeftskip=\skip44
\RaggedLeftLeftskip=\skip45
\RaggedRightLeftskip=\skip46
\CenteringRightskip=\skip47
\RaggedLeftRightskip=\skip48
\RaggedRightRightskip=\skip49
\CenteringParfillskip=\skip50
\RaggedLeftParfillskip=\skip51
\RaggedRightParfillskip=\skip52
\JustifyingParfillskip=\skip53
\CenteringParindent=\skip54
\RaggedLeftParindent=\skip55
\RaggedRightParindent=\skip56
\JustifyingParindent=\skip57
) (c:/TeXLive/2018/texmf-dist/tex/latex/xcolor/xcolor.sty
Package: xcolor 2016/05/11 v2.12 LaTeX color extensions (UK)
(c:/TeXLive/2018/texmf-dist/tex/latex/graphics-cfg/color.cfg
File: color.cfg 2016/01/02 v1.6 sample color configuration
)
Package xcolor Info: Driver file: pdftex.def on input line 225.
(c:/TeXLive/2018/texmf-dist/tex/latex/graphics-def/pdftex.def
File: pdftex.def 2018/01/08 v1.0l Graphics/color driver for pdftex
)
Package xcolor Info: Model `cmy' substituted by `cmy0' on input line
1348.
Package xcolor Info: Model `hsb' substituted by `rgb' on input line 1352.
Package xcolor Info: Model `RGB' extended on input line 1364.
Package xcolor Info: Model `HTML' substituted by `rgb' on input line
1366.
Package xcolor Info: Model `Hsb' substituted by `hsb' on input line 1367.
Package xcolor Info: Model `tHsb' substituted by `hsb' on input line
1368.
Package xcolor Info: Model `HSB' substituted by `hsb' on input line 1369.
Package xcolor Info: Model `Gray' substituted by `gray' on input line
1370.
Package xcolor Info: Model `wave' substituted by `hsb' on input line
1371.
) (c:/TeXLive/2018/texmf-dist/tex/latex/colortbl/colortbl.sty
Package: colortbl 2018/05/02 v1.0c Color table columns (DPC)
(c:/TeXLive/2018/texmf-dist/tex/latex/tools/array.sty
Package: array 2018/04/30 v2.4h Tabular extension package (FMi)
\col@sep=\dimen104
\ar@mcellbox=\box26
\extrarowheight=\dimen105
\NC@list=\toks24
\extratabsurround=\skip58
```

```
\backup@length=\skip59
\ar@cellbox=\box27
)
\everycr=\toks25
\minrowclearance=\skip60
) (c:/TeXLive/2018/texmf-dist/tex/latex/graphics/graphicx.sty
Package: graphicx 2017/06/01 v1.1a Enhanced LaTeX Graphics (DPC,SPQR)
(c:/TeXLive/2018/texmf-dist/tex/latex/graphics/graphics.sty
Package: graphics 2017/06/25 v1.2c Standard LaTeX Graphics (DPC,SPQR)
(c:/TeXLive/2018/texmf-dist/tex/latex/graphics/trig.sty
Package: trig 2016/01/03 v1.10 sin cos tan (DPC)
) (c:/TeXLive/2018/texmf-dist/tex/latex/graphics-cfg/graphics.cfg
File: graphics.cfg 2016/06/04 v1.11 sample graphics configuration
)
Package graphics Info: Driver file: pdftex.def on input line 99.
)
\Gin@req@height=\dimen106
\Gin@req@width=\dimen107
) (c:/TeXLive/2018/texmf-dist/tex/latex/etoolbox/etoolbox.sty
Package: etoolbox 2018/02/11 v2.5e e-TeX tools for LaTeX (JAW)
\etb@tempcnta=\count91
) (c:/TeXLive/2018/texmf-dist/tex/latex/xpatch/xpatch.sty
(c:/TeXLive/2018/texm
f-dist/tex/latex/l3kernel/expl3.sty
Package: expl3 2018-06-14 L3 programming layer (loader)
(c:/TeXLive/2018/texmf-dist/tex/latex/l3kernel/expl3-code.tex
Package: expl3 2018-06-14 L3 programming layer (code)
\c_max_int=\count92
\l_tmpa_int=\count93
\l_tmpb_int=\count94
\g_tmpa_int=\count95
\g_tmpb_int=\count96
\g__kernel_prg_map_int=\count97
\c_log_iow=\count98
\l_iow_line_count_int=\count99
\l__iow_line_target_int=\count100
\l__iow_one_indent_int=\count101
\l__iow_indent_int=\count102
\c_zero_dim=\dimen108
\c_max_dim=\dimen109
\l_tmpa_dim=\dimen110
\l_tmpb_dim=\dimen111
\g_tmpa_dim=\dimen112
\g_tmpb_dim=\dimen113
\c_zero_skip=\skip61
\c_max_skip=\skip62
\l_tmpa_skip=\skip63
\l_tmpb_skip=\skip64
\g_tmpa_skip=\skip65
\g_tmpb_skip=\skip66
\c_zero_muskip=\muskip13
\c_max_muskip=\muskip14
\l_tmpa_muskip=\muskip15
\l_tmpb_muskip=\muskip16
```

```
\g_tmpa_muskip=\muskip17
\g_tmpb_muskip=\muskip18
\l_keys_choice_int=\count103
\l__intarray_loop_int=\count104
\c__intarray_sp_dim=\dimen114
\g__intarray_font_int=\count105
\c__fp_leading_shift_int=\count106
\c__fp_middle_shift_int=\count107
\c__fp_trailing_shift_int=\count108
\c__fp_big_leading_shift_int=\count109
\c__fp_big_middle_shift_int=\count110
\c__fp_big_trailing_shift_int=\count111
\c__fp_Bigg_leading_shift_int=\count112
\c__fp_Bigg_middle_shift_int=\count113
\c__fp_Bigg_trailing_shift_int=\count114
\c__kernel_randint_max_int=\count115
\g__fp_array_int=\count116
\l__fp_array_loop_int=\count117
\l__sort_length_int=\count118
\l__sort_min_int=\count119
\l__sort_top_int=\count120
\l__sort_max_int=\count121
\l__sort_true_max_int=\count122
\l__sort_block_int=\count123
\l__sort_begin_int=\count124
\l__sort_end_int=\count125
\l__sort_A_int=\count126
\l__sort_B_int=\count127
\l__sort_C_int=\count128
\l__tl_analysis_normal_int=\count129
\l__tl_analysis_index_int=\count130
\l__tl_analysis_nesting_int=\count131
\l__tl_analysis_type_int=\count132
\l__regex_internal_a_int=\count133
\l__regex_internal_b_int=\count134
\l__regex_internal_c_int=\count135
\l__regex_balance_int=\count136
\l__regex_group_level_int=\count137
\l__regex_mode_int=\count138
\c__regex_cs_in_class_mode_int=\count139
\c__regex_cs_mode_int=\count140
\l__regex_catcodes_int=\count141
\l__regex_default_catcodes_int=\count142
\c__regex_catcode_D_int=\count143
\c__regex_catcode_S_int=\count144
\c__regex_catcode_L_int=\count145
\c__regex_catcode_O_int=\count146
\c__regex_catcode_A_int=\count147
\c__regex_all_catcodes_int=\count148
\l__regex_show_lines_int=\count149
\l__regex_min_state_int=\count150
\l__regex_max_state_int=\count151
\l__regex_left_state_int=\count152
\l__regex_right_state_int=\count153
```

```
\l__regex_capturing_group_int=\count154
\l__regex_min_pos_int=\count155
\l__regex_max_pos_int=\count156
\l__regex_curr_pos_int=\count157
\l__regex_start_pos_int=\count158
\l__regex_success_pos_int=\count159
\l__regex_curr_char_int=\count160
\l__regex_curr_catcode_int=\count161
\l__regex_last_char_int=\count162
\l__regex_case_changed_char_int=\count163
\l__regex_curr_state_int=\count164
\l__regex_step_int=\count165
\l__regex_min_active_int=\count166
\l__regex_max_active_int=\count167
\l__regex_replacement_csnames_int=\count168
\l__regex_match_count_int=\count169
\l__regex_min_submatch_int=\count170
\l__regex_submatch_int=\count171
\l__regex_zeroth_submatch_int=\count172
\g__regex_trace_regex_int=\count173
\c_empty_box=\box28
\l_tmpa_box=\box29
\l_tmpb_box=\box30
\g_tmpa_box=\box31
\g_tmpb_box=\box32
\l__box_top_dim=\dimen115
\l__box_bottom_dim=\dimen116
\l__box_left_dim=\dimen117
\l__box_right_dim=\dimen118
\l__box_top_new_dim=\dimen119
\l__box_bottom_new_dim=\dimen120
\l__box_left_new_dim=\dimen121
\l__box_right_new_dim=\dimen122
\l__box_internal_box=\box33
\l__coffin_internal_box=\box34
\l__coffin_internal_dim=\dimen123
\l__coffin_offset_x_dim=\dimen124
\l__coffin_offset_y_dim=\dimen125
\l__coffin_x_dim=\dimen126
\l__coffin_y_dim=\dimen127
\l__coffin_x_prime_dim=\dimen128
\l__coffin_y_prime_dim=\dimen129
\c_empty_coffin=\box35
\l__coffin_aligned_coffin=\box36
\l__coffin_aligned_internal_coffin=\box37
\l_tmpa_coffin=\box38
\l_tmpb_coffin=\box39
\l__coffin_display_coffin=\box40
\l__coffin_display_coord_coffin=\box41
\l__coffin_display_pole_coffin=\box42
\l__coffin_display_offset_dim=\dimen130
\l__coffin_display_x_dim=\dimen131
\l__coffin_display_y_dim=\dimen132
\g__file_internal_ior=\read1
```

```
\l__coffin_bounding_shift_dim=\dimen133
\l__coffin_left_corner_dim=\dimen134
\l__coffin_right_corner_dim=\dimen135
\l__coffin_bottom_corner_dim=\dimen136
\l__coffin_top_corner_dim=\dimen137
\l__coffin_scaled_total_height_dim=\dimen138
\l__coffin_scaled_width_dim=\dimen139
\l__seq_internal_a_int=\count174
\l__seq_internal_b_int=\count175
) (c:/TeXLive/2018/texmf-dist/tex/latex/l3kernel/l3pdfmode.def
File: l3pdfmode.def 2018-06-14 v L3 Experimental driver: PDF mode
\l__driver_color_stack_int=\count176
))
Package: xpatch 2012/10/02 v0.3 Extending etoolbox patching commands
(c:/TeXLive/2018/texmf-dist/tex/latex/l3packages/xparse/xparse.sty
Package: xparse 2018-05-12 L3 Experimental document command parser
\l__xparse_current_arg_int=\count177
\g__xparse_grabber_int=\count178
\l__xparse_m_args_int=\count179
\l__xparse_mandatory_args_int=\count180
\l__xparse_v_nesting_int=\count181
)
.................................................
. LaTeX info: "xparse/define-command"
.
. Defining command \xpatchcmd with sig. '' on line 125.
.................................................
.................................................
. LaTeX info: "xparse/define-command"
.
. Defining command \xpretocmd with sig. '' on line 126.
.................................................
.................................................
. LaTeX info: "xparse/define-command"
.
. Defining command \xapptocmd with sig. '' on line 127.
.................................................
.................................................
. LaTeX info: "xparse/define-command"
.
. Defining command \xshowcmd with sig. '' on line 128.
.................................................
.................................................
. LaTeX info: "xparse/define-command"
.
. Defining command \xpatchbibmacro with sig. 'm' on line 131.
.................................................
.................................................
. LaTeX info: "xparse/define-command"
.
. Defining command \xpretobibmacro with sig. 'm' on line 133.
.................................................
.................................................
. LaTeX info: "xparse/define-command"
```

```
.
. Defining command \xapptobibmacro with sig. 'm' on line 135.
.................................................
.................................................
. LaTeX info: "xparse/define-command"
.
. Defining command \xshowbibmacro with sig. 'm' on line 137.
.................................................
.................................................
. LaTeX info: "xparse/define-command"
.
. Defining command \xpatchfieldformat with sig. 'O{*}m' on line 139.
.................................................
.................................................
. LaTeX info: "xparse/define-command"
.
. Defining command \xpretofieldformat with sig. 'O{*}m' on line 141.
.................................................
.................................................
. LaTeX info: "xparse/define-command"
.
. Defining command \xapptofieldformat with sig. 'O{*}m' on line 143.
.................................................
.................................................
. LaTeX info: "xparse/define-command"
.
. Defining command \xshowfieldformat with sig. 'O{*}m' on line 145.
.................................................
.................................................
. LaTeX info: "xparse/define-command"
.
. Defining command \xpatchnameformat with sig. 'O{*}m' on line 147.
.................................................
.................................................
. LaTeX info: "xparse/define-command"
.
. Defining command \xpretonameformat with sig. 'O{*}m' on line 149.
.................................................
.................................................
. LaTeX info: "xparse/define-command"
.
. Defining command \xapptonameformat with sig. 'O{*}m' on line 151.
.................................................
.................................................
. LaTeX info: "xparse/define-command"
.
. Defining command \xshownameformat with sig. 'O{*}m' on line 153.
.................................................
.................................................
. LaTeX info: "xparse/define-command"
.
. Defining command \xpatchlistformat with sig. 'O{*}m' on line 155.
.................................................
.................................................
```

```
. LaTeX info: "xparse/define-command"
.
. Defining command \xpretolistformat with sig. 'O{*}m' on line 157.
.................................................
.................................................
. LaTeX info: "xparse/define-command"
.
. Defining command \xapptolistformat with sig. 'O{*}m' on line 159.
.................................................
.................................................
. LaTeX info: "xparse/define-command"
.
. Defining command \xshowlistformat with sig. 'O{*}m' on line 161.
.................................................
.................................................
. LaTeX info: "xparse/define-command"
.
. Defining command \xpatchindexfieldformat with sig. 'O{*}m' on line 163.
.................................................
.................................................
. LaTeX info: "xparse/define-command"
.
. Defining command \xpretoindexfieldformat with sig. 'O{*}m' on line 165.
.................................................
.................................................
. LaTeX info: "xparse/define-command"
.
. Defining command \xapptoindexfieldformat with sig. 'O{*}m' on line 167.
.................................................
.................................................
. LaTeX info: "xparse/define-command"
.
. Defining command \xshowindexfieldformat with sig. 'O{*}m' on line 169.
.................................................
.................................................
. LaTeX info: "xparse/define-command"
.
. Defining command \xpatchindexnameformat with sig. 'O{*}m' on line 171.
.................................................
.................................................
. LaTeX info: "xparse/define-command"
.
. Defining command \xpretoindexnameformat with sig. 'O{*}m' on line 173.
.................................................
.................................................
. LaTeX info: "xparse/define-command"
.
. Defining command \xapptoindexnameformat with sig. 'O{*}m' on line 175.
.................................................
.................................................
. LaTeX info: "xparse/define-command"
.
. Defining command \xshowindexnameformat with sig. 'O{*}m' on line 177.
.................................................
```

```
.................................................
. LaTeX info: "xparse/define-command"
.
. Defining command \xpatchindexlistformat with sig. 'O{*}m' on line 179.
.................................................
.................................................
. LaTeX info: "xparse/define-command"
.
. Defining command \xpretoindexlistformat with sig. 'O{*}m' on line 181.
.................................................
.................................................
. LaTeX info: "xparse/define-command"
.
. Defining command \xappindextolistformat with sig. 'O{*}m' on line 183.
.................................................
.................................................
. LaTeX info: "xparse/define-command"
.
. Defining command \xshowindexlistformat with sig. 'O{*}m' on line 185.
.................................................
.................................................
. LaTeX info: "xparse/define-command"
.
. Defining command \xpatchbibdriver with sig. 'm' on line 187.
.................................................
.................................................
. LaTeX info: "xparse/define-command"
.
. Defining command \xpretobibdriver with sig. 'm' on line 189.
.................................................
.................................................
. LaTeX info: "xparse/define-command"
.
. Defining command \xapptobibdriver with sig. 'm' on line 191.
.................................................
.................................................
. LaTeX info: "xparse/define-command"
.
. Defining command \xshowbibdriver with sig. 'm' on line 193.
.................................................
) (c:/TeXLive/2018/texmf-dist/tex/latex/environ/environ.sty
Package: environ 2014/05/04 v0.3 A new way to define environments
(c:/TeXLive/2018/texmf-dist/tex/latex/trimspaces/trimspaces.sty
Package: trimspaces 2009/09/17 v1.1 Trim spaces around a token list
)
\@envbody=\toks26
) (c:/TeXLive/2018/texmf-dist/tex/latex/lastpage/lastpage.sty
Package: lastpage 2015/03/29 v1.2m Refers to last page's name (HMM; JPG)
) (c:/TeXLive/2018/texmf-dist/tex/latex/graphics/rotating.sty
Package: rotating 2016/08/11 v2.16d rotated objects in LaTeX
(c:/TeXLive/2018/texmf-dist/tex/latex/base/ifthen.sty
Package: ifthen 2014/09/29 v1.1c Standard LaTeX ifthen package (DPC)
)
\c@r@tfl@t=\count182
```

```
\rotFPtop=\skip67
\rotFPbot=\skip68
\rot@float@box=\box43
\rot@mess@toks=\toks27
) (c:/TeXLive/2018/texmf-dist/tex/latex/graphics/lscape.sty
Package: lscape 2000/10/22 v3.01 Landscape Pages (DPC)
) (c:/TeXLive/2018/texmf-dist/tex/latex/tools/afterpage.sty
Package: afterpage 2014/10/28 v1.08 After-Page Package (DPC)
\AP@output=\toks28
\AP@partial=\box44
\AP@footins=\box45
) (c:/TeXLive/2018/texmf-dist/tex/latex/textpos/textpos.sty
Package: textpos 2016/06/07 v1.8
Package: textpos 2016/06/07 1.8, absolute positioning of text on the page
(c:/TeXLive/2018/texmf-dist/tex/latex/ms/everyshi.sty
Package: everyshi 2001/05/15 v3.00 EveryShipout Package (MS)
)
\TP@textbox=\box46
\TP@holdbox=\box47
\TPHorizModule=\dimen140
\TPVertModule=\dimen141
\TP@margin=\dimen142
\TP@absmargin=\dimen143
Grid set 16 x 16 = 37.34424pt x 52.81541pt
\TPboxrulesize=\dimen144
\TP@ox=\dimen145
\TP@oy=\dimen146
\TP@tbargs=\toks29
\TP@prevdepth=\dimen147
TextBlockOrigin set to 0pt x 0pt
) (c:/TeXLive/2018/texmf-dist/tex/latex/url/url.sty
\Urlmuskip=\muskip19
Package: url 2013/09/16  ver 3.4  Verb mode for urls, etc.
) (c:/TeXLive/2018/texmf-dist/tex/latex/caption/newfloat.sty
Package: newfloat 2018/03/04 v1.1a Defining new floating environments
(AR)
) (c:/TeXLive/2018/texmf-dist/tex/latex/mdframed/mdframed.sty
Package: mdframed 2013/07/01 1.9b: mdframed
(c:/TeXLive/2018/texmf-dist/tex/latex/oberdiek/kvoptions.sty
Package: kvoptions 2016/05/16 v3.12 Key value format for package options
(HO)
(c:/TeXLive/2018/texmf-dist/tex/generic/oberdiek/ltxcmds.sty
Package: ltxcmds 2016/05/16 v1.23 LaTeX kernel commands for general use
(HO)
) (c:/TeXLive/2018/texmf-dist/tex/generic/oberdiek/kvsetkeys.sty
Package: kvsetkeys 2016/05/16 v1.17 Key value parser (HO)
(c:/TeXLive/2018/texmf-dist/tex/generic/oberdiek/infwarerr.sty
Package: infwarerr 2016/05/16 v1.4 Providing info/warning/error messages
(HO)
) (c:/TeXLive/2018/texmf-dist/tex/generic/oberdiek/etexcmds.sty
Package: etexcmds 2016/05/16 v1.6 Avoid name clashes with e-TeX commands
(HO)
Package etexcmds Info: Could not find \expanded.
```

```
(etexcmds)             That can mean that you are not using pdfTeX 1.50
or
(etexcmds)             that some package has redefined \expanded.
(etexcmds)             In the latter case, load this package earlier.
))) (c:/TeXLive/2018/texmf-dist/tex/latex/oberdiek/zref-abspage.sty
Package: zref-abspage 2016/05/21 v2.26 Module abspage for zref (HO)
(c:/TeXLive/2018/texmf-dist/tex/latex/oberdiek/zref-base.sty
Package: zref-base 2016/05/21 v2.26 Module base for zref (HO)
(c:/TeXLive/2018/texmf-dist/tex/generic/oberdiek/kvdefinekeys.sty
Package: kvdefinekeys 2016/05/16 v1.4 Define keys (HO)
) (c:/TeXLive/2018/texmf-dist/tex/generic/oberdiek/pdftexcmds.sty
Package: pdftexcmds 2018/01/30 v0.27 Utility functions of pdfTeX for
LuaTeX (HO
)
Package pdftexcmds Info: LuaTeX not detected.
Package pdftexcmds Info: \pdf@primitive is available.
Package pdftexcmds Info: \pdf@ifprimitive is available.
Package pdftexcmds Info: \pdfdraftmode found.
) (c:/TeXLive/2018/texmf-dist/tex/latex/oberdiek/auxhook.sty
Package: auxhook 2016/05/16 v1.4 Hooks for auxiliary files (HO)
)
Package zref Info: New property list: main on input line 759.
Package zref Info: New property: default on input line 760.
Package zref Info: New property: page on input line 761.
) (c:/TeXLive/2018/texmf-dist/tex/generic/oberdiek/atbegshi.sty
Package: atbegshi 2016/06/09 v1.18 At begin shipout hook (HO)
)
\c@abspage=\count183
Package zref Info: New property: abspage on input line 62.
) (c:/TeXLive/2018/texmf-dist/tex/latex/needspace/needspace.sty
Package: needspace 2010/09/12 v1.3d reserve vertical space
)
\mdf@templength=\skip69
\c@mdf@globalstyle@cnt=\count184
\mdf@skipabove@length=\skip70
\mdf@skipbelow@length=\skip71
\mdf@leftmargin@length=\skip72
\mdf@rightmargin@length=\skip73
\mdf@innerleftmargin@length=\skip74
\mdf@innerrightmargin@length=\skip75
\mdf@innertopmargin@length=\skip76
\mdf@innerbottommargin@length=\skip77
\mdf@splittopskip@length=\skip78
\mdf@splitbottomskip@length=\skip79
\mdf@outermargin@length=\skip80
\mdf@innermargin@length=\skip81
\mdf@linewidth@length=\skip82
\mdf@innerlinewidth@length=\skip83
\mdf@middlelinewidth@length=\skip84
\mdf@outerlinewidth@length=\skip85
\mdf@roundcorner@length=\skip86
\mdf@footenotedistance@length=\skip87
\mdf@userdefinedwidth@length=\skip88
\mdf@needspace@length=\skip89
```

```
\mdf@frametitleaboveskip@length=\skip90
\mdf@frametitlebelowskip@length=\skip91
\mdf@frametitlerulewidth@length=\skip92
\mdf@frametitleleftmargin@length=\skip93
\mdf@frametitlerightmargin@length=\skip94
\mdf@shadowsize@length=\skip95
\mdf@extratopheight@length=\skip96
\mdf@subtitleabovelinewidth@length=\skip97
\mdf@subtitlebelowlinewidth@length=\skip98
\mdf@subtitleaboveskip@length=\skip99
\mdf@subtitlebelowskip@length=\skip100
\mdf@subtitleinneraboveskip@length=\skip101
\mdf@subtitleinnerbelowskip@length=\skip102
\mdf@subsubtitleabovelinewidth@length=\skip103
\mdf@subsubtitlebelowlinewidth@length=\skip104
\mdf@subsubtitleaboveskip@length=\skip105
\mdf@subsubtitlebelowskip@length=\skip106
\mdf@subsubtitleinneraboveskip@length=\skip107
\mdf@subsubtitleinnerbelowskip@length=\skip108
(c:/TeXLive/2018/texmf-dist/tex/latex/mdframed/md-frame-0.mdf
File: md-frame-0.mdf 2013/07/01\ 1.9b: md-frame-0
)
\mdf@frametitlebox=\box48
\mdf@footnotebox=\box49
\mdf@splitbox@one=\box50
\mdf@splitbox@two=\box51
\mdf@splitbox@save=\box52
\mdfsplitboxwidth=\skip109
\mdfsplitboxtotalwidth=\skip110
\mdfsplitboxheight=\skip111
\mdfsplitboxdepth=\skip112
\mdfsplitboxtotalheight=\skip113
\mdfframetitleboxwidth=\skip114
\mdfframetitleboxtotalwidth=\skip115
\mdfframetitleboxheight=\skip116
\mdfframetitleboxdepth=\skip117
\mdfframetitleboxtotalheight=\skip118
\mdffootnoteboxwidth=\skip119
\mdffootnoteboxtotalwidth=\skip120
\mdffootnoteboxheight=\skip121
\mdffootnoteboxdepth=\skip122
\mdffootnoteboxtotalheight=\skip123
\mdftotallinewidth=\skip124
\mdfboundingboxwidth=\skip125
\mdfboundingboxtotalwidth=\skip126
\mdfboundingboxheight=\skip127
\mdfboundingboxdepth=\skip128
\mdfboundingboxtotalheight=\skip129
\mdf@freevspace@length=\skip130
\mdf@horizontalwidthofbox@length=\skip131
\mdf@verticalmarginwhole@length=\skip132
\mdf@horizontalspaceofbox=\skip133
\mdfsubtitleheight=\skip134
\mdfsubsubtitleheight=\skip135
```

```
\c@mdfcountframes=\count185

****** mdframed patching \endmdf@trivlist

****** -- success******

.................................................
. LaTeX info: "xparse/define-command"
.
. Defining command \newmdtheoremenv with sig. 'O{} m o m o ' on line 601.
.................................................
.................................................
. LaTeX info: "xparse/define-command"
.
. Defining command \mdtheorem with sig. ' O{} m o m o ' on line 701.
.................................................
\mdf@envdepth=\count186
\c@mdf@env@i=\count187
\c@mdf@env@ii=\count188
\c@mdf@zref@counter=\count189
Package zref Info: New property: mdf@pagevalue on input line 895.
) (c:/TeXLive/2018/texmf-dist/tex/latex/titlesec/titlesec.sty
Package: titlesec 2016/03/21 v2.10.2 Sectioning titles
\ttl@box=\box53
\beforetitleunit=\skip136
\aftertitleunit=\skip137
\ttl@plus=\dimen148
\ttl@minus=\dimen149
\ttl@toksa=\toks30
\titlewidth=\dimen150
\titlewidthlast=\dimen151
\titlewidthfirst=\dimen152
) (c:/TeXLive/2018/texmf-dist/tex/latex/koma-script/scrextend.sty
Package: scrextend 2018/03/30 v3.25 KOMA-Script package (extend other
classes w
ith features of KOMA-Script classes)
(c:/TeXLive/2018/texmf-dist/tex/latex/koma-script/scrkbase.sty
Package: scrkbase 2018/03/30 v3.25 KOMA-Script package (KOMA-Script-
dependent b
asics and keyval usage)
(c:/TeXLive/2018/texmf-dist/tex/latex/koma-script/scrbase.sty
Package: scrbase 2018/03/30 v3.25 KOMA-Script package (KOMA-Script-
independent
basics and keyval usage)
(c:/TeXLive/2018/texmf-dist/tex/latex/koma-script/scrlfile.sty
Package: scrlfile 2018/03/30 v3.25 KOMA-Script package (loading files)
)))
Package scrextend Info: unexpected definition of `\@makefnmark'.
(scrextend)             Trying to patch it on input line 1544.
Package scrextend Info: patch seems to be successfull on input line 1544.
)

LaTeX Font Warning: Font shape `T1/cmr/m/n' in size <7.5> not available
(Font)              size <7> substituted on input line 65.
```

```
(c:/TeXLive/2018/texmf-dist/tex/latex/tools/calc.sty
Package: calc 2017/05/25 v4.3 Infix arithmetic (KKT,FJ)
\calc@Acount=\count190
\calc@Bcount=\count191
\calc@Adimen=\dimen153
\calc@Bdimen=\dimen154
\calc@Askip=\skip138
\calc@Bskip=\skip139
LaTeX Info: Redefining \setlength on input line 80.
LaTeX Info: Redefining \addtolength on input line 81.
\calc@Ccount=\count192
\calc@Cskip=\skip140
) (c:/TeXLive/2018/texmf-dist/tex/latex/geometry/geometry.sty
Package: geometry 2018/04/16 v5.8 Page Geometry
(c:/TeXLive/2018/texmf-dist/tex/generic/oberdiek/ifvtex.sty
Package: ifvtex 2016/05/16 v1.6 Detect VTeX and its facilities (HO)
Package ifvtex Info: VTeX not detected.
)
\Gm@cnth=\count193
\Gm@cntv=\count194
\c@Gm@tempcnt=\count195
\Gm@bindingoffset=\dimen155
\Gm@wd@mp=\dimen156
\Gm@odd@mp=\dimen157
\Gm@even@mp=\dimen158
\Gm@layoutwidth=\dimen159
\Gm@layoutheight=\dimen160
\Gm@layouthoffset=\dimen161
\Gm@layoutvoffset=\dimen162
\Gm@dimlist=\toks31
) (c:/TeXLive/2018/texmf-dist/tex/latex/hyperref/hyperref.sty
Package: hyperref 2018/02/06 v6.86b Hypertext links for LaTeX
(c:/TeXLive/2018/texmf-dist/tex/generic/oberdiek/hobsub-hyperref.sty
Package: hobsub-hyperref 2016/05/16 v1.14 Bundle oberdiek, subset
hyperref (HO)

(c:/TeXLive/2018/texmf-dist/tex/generic/oberdiek/hobsub-generic.sty
Package: hobsub-generic 2016/05/16 v1.14 Bundle oberdiek, subset generic
(HO)
Package: hobsub 2016/05/16 v1.14 Construct package bundles (HO)
Package hobsub Info: Skipping package `infwarerr' (already loaded).
Package hobsub Info: Skipping package `ltxcmds' (already loaded).
Package hobsub Info: Skipping package `ifluatex' (already loaded).
Package hobsub Info: Skipping package `ifvtex' (already loaded).
Package: intcalc 2016/05/16 v1.2 Expandable calculations with integers
(HO)
Package hobsub Info: Skipping package `ifpdf' (already loaded).
Package hobsub Info: Skipping package `etexcmds' (already loaded).
Package hobsub Info: Skipping package `kvsetkeys' (already loaded).
Package hobsub Info: Skipping package `kvdefinekeys' (already loaded).
Package hobsub Info: Skipping package `pdftexcmds' (already loaded).
Package: pdfescape 2016/05/16 v1.14 Implements pdfTeX's escape features
(HO)
```

```
Package: bigintcalc 2016/05/16 v1.4 Expandable calculations on big
integers (HO
)
Package: bitset 2016/05/16 v1.2 Handle bit-vector datatype (HO)
Package: uniquecounter 2016/05/16 v1.3 Provide unlimited unique counter
(HO)
)
Package hobsub Info: Skipping package `hobsub' (already loaded).
Package: letltxmacro 2016/05/16 v1.5 Let assignment for LaTeX macros (HO)
Package: hopatch 2016/05/16 v1.3 Wrapper for package hooks (HO)
Package: xcolor-patch 2016/05/16 xcolor patch
Package: atveryend 2016/05/16 v1.9 Hooks at the very end of document (HO)
Package hobsub Info: Skipping package `atbegshi' (already loaded).
Package: refcount 2016/05/16 v3.5 Data extraction from label references
(HO)
Package: hycolor 2016/05/16 v1.8 Color options for hyperref/bookmark (HO)
)
\@linkdim=\dimen163
\Hy@linkcounter=\count196
\Hy@pagecounter=\count197
(c:/TeXLive/2018/texmf-dist/tex/latex/hyperref/pd1enc.def
File: pd1enc.def 2018/02/06 v6.86b Hyperref: PDFDocEncoding definition
(HO)
Now handling font encoding PD1 ...
... no UTF-8 mapping file for font encoding PD1
)
\Hy@SavedSpaceFactor=\count198
(c:/TeXLive/2018/texmf-dist/tex/latex/latexconfig/hyperref.cfg
File: hyperref.cfg 2002/06/06 v1.2 hyperref configuration of TeXLive
)
Package hyperref Info: Option `colorlinks' set `true' on input line 4383.
Package hyperref Info: Hyper figures OFF on input line 4509.
Package hyperref Info: Link nesting OFF on input line 4514.
Package hyperref Info: Hyper index ON on input line 4517.
Package hyperref Info: Plain pages OFF on input line 4524.
Package hyperref Info: Backreferencing OFF on input line 4529.
Package hyperref Info: Implicit mode ON; LaTeX internals redefined.
Package hyperref Info: Bookmarks ON on input line 4762.
\c@Hy@tempcnt=\count199
LaTeX Info: Redefining \url on input line 5115.
\XeTeXLinkMargin=\dimen164
\Fld@menulength=\count266
\Field@Width=\dimen165
\Fld@charsize=\dimen166
Package hyperref Info: Hyper figures OFF on input line 6369.
Package hyperref Info: Link nesting OFF on input line 6374.
Package hyperref Info: Hyper index ON on input line 6377.
Package hyperref Info: backreferencing OFF on input line 6384.
Package hyperref Info: Link coloring ON on input line 6387.
Package hyperref Info: Link coloring with OCG OFF on input line 6394.
Package hyperref Info: PDF/A mode OFF on input line 6399.
LaTeX Info: Redefining \ref on input line 6439.
LaTeX Info: Redefining \pageref on input line 6443.
\Hy@abspage=\count267
```

```
\c@Item=\count268
\c@Hfootnote=\count269
)
Package hyperref Info: Driver (autodetected): hpdftex.
(c:/TeXLive/2018/texmf-dist/tex/latex/hyperref/hpdftex.def
File: hpdftex.def 2018/02/06 v6.86b Hyperref driver for pdfTeX
\HyAnn@Count=\count270
\Fld@listcount=\count271
\c@bookmark@seq@number=\count272
(c:/TeXLive/2018/texmf-dist/tex/latex/oberdiek/rerunfilecheck.sty
Package: rerunfilecheck 2016/05/16 v1.8 Rerun checks for auxiliary files
(HO)
Package uniquecounter Info: New unique counter `rerunfilecheck' on input
line 2
82.
)
\Hy@SectionHShift=\skip141
) (c:/TeXLive/2018/texmf-dist/tex/latex/preprint/authblk.sty
Package: authblk 2001/02/27 1.3 (PWD)
\affilsep=\skip142
\@affilsep=\skip143
\c@Maxaffil=\count273
\c@authors=\count274
\c@affil=\count275
) (c:/TeXLive/2018/texmf-dist/tex/latex/footmisc/footmisc.sty
Package: footmisc 2011/06/06 v5.5b a miscellany of footnote facilities
\FN@temptoken=\toks32
\footnotemargin=\dimen167
\c@pp@next@reset=\count276
Package footmisc Info: Declaring symbol style bringhurst on input line
855.
Package footmisc Info: Declaring symbol style chicago on input line 863.
Package footmisc Info: Declaring symbol style wiley on input line 872.
Package footmisc Info: Declaring symbol style lamport-robust on input
line 883.

Package footmisc Info: Declaring symbol style lamport* on input line 903.
Package footmisc Info: Declaring symbol style lamport*-robust on input
line 924
.
) (c:/TeXLive/2018/texmf-dist/tex/latex/fancyhdr/fancyhdr.sty
Package: fancyhdr 2017/06/30 v3.9a Extensive control of page headers and
footer
s
\f@nch@headwidth=\skip144
\f@nch@O@elh=\skip145
\f@nch@O@erh=\skip146
\f@nch@O@olh=\skip147
\f@nch@O@orh=\skip148
\f@nch@O@elf=\skip149
\f@nch@O@erf=\skip150
\f@nch@O@olf=\skip151
\f@nch@O@orf=\skip152
) (c:/TeXLive/2018/texmf-dist/tex/generic/oberdiek/alphalph.sty
```

```
Package: alphalph 2016/05/16 v2.5 Convert numbers to letters (HO)
)
\c@authorfn=\count277
(c:/TeXLive/2018/texmf-dist/tex/latex/abstract/abstract.sty
Package: abstract 2009/06/08 v1.2a configurable abstracts
\abstitleskip=\skip153
\absleftindent=\skip154
\absrightindent=\skip155
\absparindent=\skip156
\absparsep=\skip157
)
Package newfloat Info: New float `keypoints' with options
`placement=t!,name=kp
t' on input line 286.
\c@keypoints=\count278
\newfloat@ftype=\count279
Package newfloat Info: float type `keypoints'=8 on input line 286.
(c:/TeXLive/2018/texmf-dist/tex/latex/enumitem/enumitem.sty
Package: enumitem 2011/09/28 v3.5.2 Customized lists
\labelindent=\skip158
\enit@outerparindent=\dimen168
\enit@toks=\toks33
\enit@inbox=\box54
\enitdp@description=\count280
) (c:/TeXLive/2018/texmf-dist/tex/latex/quoting/quoting.sty
Package: quoting 2014/01/28 v0.1c Consolidated environment for displayed
text
\quo@toppartop=\skip159
) (c:/TeXLive/2018/texmf-dist/tex/latex/sttools/stfloats.sty
Package: stfloats 2017/03/27 v3.3 Improve float mechanism and
baselineskip sett
ings
\@dblbotnum=\count281
\c@dblbotnumber=\count282
) (c:/TeXLive/2018/texmf-dist/tex/latex/booktabs/booktabs.sty
Package: booktabs 2016/04/27 v1.618033 publication quality tables
\heavyrulewidth=\dimen169
\lightrulewidth=\dimen170
\cmidrulewidth=\dimen171
\belowrulesep=\dimen172
\belowbottomsep=\dimen173
\aboverulesep=\dimen174
\abovetopsep=\dimen175
\cmidrulesep=\dimen176
\cmidrulekern=\dimen177
\defaultaddspace=\dimen178
\@cmidla=\count283
\@cmidlb=\count284
\@aboverulesep=\dimen179
\@belowrulesep=\dimen180
\@thisruleclass=\count285
\@lastruleclass=\count286
\@thisrulewidth=\dimen181
) (c:/TeXLive/2018/texmf-dist/tex/latex/tools/tabularx.sty
```

```
Package: tabularx 2016/02/03 v2.11b `tabularx' package (DPC)
\TX@col@width=\dimen182
\TX@old@table=\dimen183
\TX@old@col=\dimen184
\TX@target=\dimen185
\TX@delta=\dimen186
\TX@cols=\count287
\TX@ftn=\toks34
)
\enitdp@tablenotes=\count288
(c:/TeXLive/2018/texmf-dist/tex/latex/caption/caption.sty
Package: caption 2018/05/01 v3.3-147 Customizing captions (AR)
(c:/TeXLive/2018/texmf-dist/tex/latex/caption/caption3.sty
Package: caption3 2018/05/27 v1.8a caption3 kernel (AR)
Package caption3 Info: TeX engine: e-TeX on input line 64.
\captionmargin=\dimen187
\captionmargin@=\dimen188
\captionwidth=\dimen189
\caption@tempdima=\dimen190
\caption@indent=\dimen191
\caption@parindent=\dimen192
\caption@hangindent=\dimen193
)
\c@ContinuedFloat=\count289
Package caption Info: hyperref package is loaded.
Package caption Info: rotating package is loaded.
) (c:/TeXLive/2018/texmf-dist/tex/latex/natbib/natbib.sty
Package: natbib 2010/09/13 8.31b (PWD, AO)
\bibhang=\skip160
\bibsep=\skip161
LaTeX Info: Redefining \cite on input line 694.
\c@NAT@ctr=\count290
)) (c:/TeXLive/2018/texmf-dist/tex/latex/siunitx/siunitx.sty
Package: siunitx 2018/05/17 v2.7s A comprehensive (SI) units package
(c:/TeXLive/2018/texmf-dist/tex/latex/amsmath/amstext.sty
Package: amstext 2000/06/29 v2.01 AMS text
(c:/TeXLive/2018/texmf-dist/tex/latex/amsmath/amsgen.sty
File: amsgen.sty 1999/11/30 v2.0 generic functions
\@emptytoks=\toks35
\ex@=\dimen194
)) (c:/TeXLive/2018/texmf-dist/tex/latex/l3packages/l3keys2e/l3keys2e.sty
Package: l3keys2e 2018-05-12 LaTeX2e option processing using LaTeX3 keys
)
\l__siunitx_tmp_box=\box55
\l__siunitx_tmp_dim=\dimen195
\l__siunitx_tmp_int=\count291
\l__siunitx_number_mantissa_length_int=\count292
\l__siunitx_number_uncert_length_int=\count293
\l__siunitx_round_int=\count294
\l__siunitx_process_decimal_int=\count295
\l__siunitx_process_uncertainty_int=\count296
\l__siunitx_process_fixed_int=\count297
\l__siunitx_process_integer_min_int=\count298
\l__siunitx_process_precision_int=\count299
```

```
\l__siunitx_group_min_int=\count300
\l__siunitx_angle_marker_box=\box56
\l__siunitx_angle_unit_box=\box57
\l__siunitx_angle_marker_dim=\dimen196
\l__siunitx_angle_unit_dim=\dimen197
\l__siunitx_unit_int=\count301
\l__siunitx_unit_denominator_int=\count302
\l__siunitx_unit_numerator_int=\count303
\l__siunitx_unit_prefix_int=\count304
\l__siunitx_unit_prefix_base_int=\count305
\l__siunitx_unit_prefix_gram_int=\count306
\l__siunitx_number_product_int=\count307
\c__siunitx_one_fill_skip=\skip162
\l__siunitx_table_unit_align_skip=\skip163
\l__siunitx_table_exponent_dim=\dimen198
\l__siunitx_table_integer_dim=\dimen199
\l__siunitx_table_mantissa_dim=\dimen256
\l__siunitx_table_marker_dim=\dimen257
\l__siunitx_table_result_dim=\dimen258
\l__siunitx_table_uncert_dim=\dimen259
\l__siunitx_table_fill_pre_dim=\dimen260
\l__siunitx_table_fill_post_dim=\dimen261
\l__siunitx_table_fill_mid_dim=\dimen262
\l__siunitx_table_pre_box=\box58
\l__siunitx_table_post_box=\box59
\l__siunitx_table_mantissa_box=\box60
\l__siunitx_table_result_box=\box61
\l__siunitx_table_number_align_skip=\skip164
\l__siunitx_table_text_align_skip=\skip165
.................................................
. LaTeX info: "xparse/define-command"
.
. Defining command \DeclareBinaryPrefix with sig. 'mmm' on line 7237.
.................................................
.................................................
. LaTeX info: "xparse/define-command"
.
. Defining command \DeclareSIPostPower with sig. 'mm' on line 7240.
.................................................
.................................................
. LaTeX info: "xparse/define-command"
.
. Defining command \DeclareSIPrefix with sig. 'mmm' on line 7243.
.................................................
.................................................
. LaTeX info: "xparse/define-command"
.
. Defining command \DeclareSIPrePower with sig. 'mm' on line 7246.
.................................................
.................................................
. LaTeX info: "xparse/define-command"
.
. Defining command \DeclareSIQualifier with sig. 'mm' on line 7249.
.................................................
```

```
.................................................
. LaTeX info: "xparse/define-command"
.
. Defining command \DeclareSIUnit with sig. 'O{}mm' on line 7252.
.................................................
.................................................
. LaTeX info: "xparse/define-command"
.
. Defining command \DeclareSIUnitWithOptions with sig. 'mmm' on line
7255.
.................................................
.................................................
. LaTeX info: "xparse/define-command"
.
. Defining command \ang with sig. 'o>{\SplitArgument {2}{;}}m' on line
7270.
.................................................
.................................................
. LaTeX info: "xparse/define-command"
.
. Defining command \num with sig. 'om' on line 7279.
.................................................
.................................................
. LaTeX info: "xparse/define-command"
.
. Defining command \numlist with sig. 'o>{\SplitList {;}}m' on line 7288.
.................................................
.................................................
. LaTeX info: "xparse/define-command"
.
. Defining command \numrange with sig. 'omm' on line 7297.
.................................................
.................................................
. LaTeX info: "xparse/define-command"
.
. Defining command \SIlist with sig. 'o>{\SplitList {;}}mm' on line 7309.
.................................................
.................................................
. LaTeX info: "xparse/define-command"
.
. Defining command \SIrange with sig. 'ommm' on line 7321.
.................................................
.................................................
. LaTeX info: "xparse/define-command"
.
. Defining command \SI with sig. 'omom' on line 7333.
.................................................
.................................................
. LaTeX info: "xparse/define-command"
.
. Defining command \sisetup with sig. 'm' on line 7336.
.................................................
.................................................
. LaTeX info: "xparse/define-command"
```

```
.
. Defining command \tablenum with sig. 'om' on line 7351.
.................................................
.................................................
. LaTeX info: "xparse/define-command"
.
. Defining command \si with sig. 'om' on line 7363.
.................................................
.................................................
. LaTeX info: "xparse/define-command"
.
. Defining command \numInBookmark with sig. 'om' on line 7410.
.................................................
.................................................
. LaTeX info: "xparse/define-command"
.
. Defining command \numrangeInBookmark with sig. 'omm' on line 7412.
.................................................
.................................................
. LaTeX info: "xparse/define-command"
.
. Defining command \SIInBookmark with sig. 'omom' on line 7414.
.................................................
.................................................
. LaTeX info: "xparse/define-command"
.
. Defining command \SIlistInBookmark with sig. 'omm' on line 7416.
.................................................
.................................................
. LaTeX info: "xparse/define-command"
.
. Defining command \SIrangeInBookmark with sig. 'ommm' on line 7418.
.................................................
.................................................
. LaTeX info: "xparse/define-command"
.
. Defining command \siInBookmark with sig. 'om' on line 7419.
.................................................
(c:/TeXLive/2018/texmf-dist/tex/latex/translator/translator.sty
Package: translator 2018/01/04 v1.12 Easy translation of strings in LaTeX
)) (c:/TeXLive/2018/texmf-dist/tex/latex/listings/listings.sty
\lst@mode=\count308
\lst@gtempboxa=\box62
\lst@token=\toks36
\lst@length=\count309
\lst@currlwidth=\dimen263
\lst@column=\count310
\lst@pos=\count311
\lst@lostspace=\dimen264
\lst@width=\dimen265
\lst@newlines=\count312
\lst@lineno=\count313
\lst@maxwidth=\dimen266
(c:/TeXLive/2018/texmf-dist/tex/latex/listings/lstmisc.sty
```

```
File: lstmisc.sty 2015/06/04 1.6 (Carsten Heinz)
\c@lstnumber=\count314
\lst@skipnumbers=\count315
\lst@framebox=\box63
) (c:/TeXLive/2018/texmf-dist/tex/latex/listings/listings.cfg
File: listings.cfg 2015/06/04 1.6 listings configuration
))
Package: listings 2015/06/04 1.6 (Carsten Heinz)
(c:/TeXLive/2018/texmf-dist/tex/generic/ulem/ulem.sty
\UL@box=\box64
\UL@hyphenbox=\box65
\UL@skip=\skip166
\UL@hook=\toks37
\UL@height=\dimen267
\UL@pe=\count316
\UL@pixel=\dimen268
\ULC@box=\box66
Package: ulem 2012/05/18
\ULdepth=\dimen269
) (./main.aux)
\openout1 = `main.aux'.

LaTeX Font Info:    Checking defaults for OML/cmm/m/it on input line 95.
LaTeX Font Info:    ... okay on input line 95.
LaTeX Font Info:    Checking defaults for T1/cmr/m/n on input line 95.
LaTeX Font Info:    ... okay on input line 95.
LaTeX Font Info:    Checking defaults for OT1/cmr/m/n on input line 95.
LaTeX Font Info:    ... okay on input line 95.
LaTeX Font Info:    Checking defaults for OMS/cmsy/m/n on input line 95.
LaTeX Font Info:    ... okay on input line 95.
LaTeX Font Info:    Checking defaults for OMX/cmex/m/n on input line 95.
LaTeX Font Info:    ... okay on input line 95.
LaTeX Font Info:    Checking defaults for U/cmr/m/n on input line 95.
LaTeX Font Info:    ... okay on input line 95.
LaTeX Font Info:    Checking defaults for TS1/cmr/m/n on input line 95.
LaTeX Font Info:    Try loading font information for TS1+cmr on input
line 95.
(c:/TeXLive/2018/texmf-dist/tex/latex/base/ts1cmr.fd
File: ts1cmr.fd 2014/09/29 v2.5h Standard LaTeX font definitions
)
LaTeX Font Info:    ... okay on input line 95.
LaTeX Font Info:    Checking defaults for PD1/pdf/m/n on input line 95.
LaTeX Font Info:    ... okay on input line 95.
LaTeX Font Info:    Try loading font information for T1+Merriweather-OsF
on inp
ut line 95.
(c:/TeXLive/2018/texmf-dist/tex/latex/merriweather/T1Merriweather-OsF.fd
File: T1Merriweather-OsF.fd 2014/01/22 (autoinst) Font definitions for
T1/Merri
weather-OsF.
)
LaTeX Font Info:    Font shape `T1/Merriweather-OsF/m/n' will be
(Font)              scaled to size 7.5pt on input line 95.
LaTeX Info: Redefining \microtypecontext on input line 95.
```

```
Package microtype Info: Generating PDF output.
Package microtype Info: Character protrusion enabled (level 2).
Package microtype Info: Using default protrusion set `alltext'.
Package microtype Info: Automatic font expansion enabled (level 2),
(microtype)             stretch: 20, shrink: 20, step: 1, non-selected.
Package microtype Info: Using default expansion set `basictext'.
Package microtype Info: No adjustment of tracking.
Package microtype Info: No adjustment of interword spacing.
Package microtype Info: No adjustment of character kerning.
Package microtype Info: Loading generic protrusion settings for font
family
(microtype)             `Merriweather-OsF' (encoding: T1).
(microtype)             For optimal results, create family-specific
settings.
(microtype)             See the microtype manual for details.
LaTeX Font Info:    Redeclaring symbol font `operators' on input line 95.
LaTeX Font Info:    Encoding `OT1' has changed to `T1' for symbol font
(Font)              `operators' in the math version `normal' on input
line 95.
LaTeX Font Info:    Overwriting symbol font `operators' in version
`normal'
(Font)                  OT1/cmr/m/n --> T1/Merriweather-OsF/m/n on input
line 9
5.
LaTeX Font Info:    Encoding `OT1' has changed to `T1' for symbol font
(Font)              `operators' in the math version `bold' on input line
95.
LaTeX Font Info:    Overwriting symbol font `operators' in version `bold'
(Font)                  OT1/cmr/bx/n --> T1/Merriweather-OsF/m/n on input
line
95.
LaTeX Font Info:    Overwriting symbol font `operators' in version `bold'
(Font)                  T1/Merriweather-OsF/m/n --> T1/Merriweather-
OsF/bx/n on
 input line 95.
LaTeX Font Info:    Redeclaring math alphabet \mathbf on input line 95.
LaTeX Font Info:    Overwriting math alphabet `\mathbf' in version
`normal'
(Font)                  OT1/cmr/bx/n --> T1/Merriweather-OsF/bx/n on
input line
 95.
LaTeX Font Info:    Overwriting math alphabet `\mathbf' in version `bold'
(Font)                  OT1/cmr/bx/n --> T1/Merriweather-OsF/bx/n on
input line
 95.
LaTeX Font Info:    Redeclaring math alphabet \mathsf on input line 95.
LaTeX Font Info:    Overwriting math alphabet `\mathsf' in version
`normal'
(Font)                  OT1/cmss/m/n --> T1/MerriweatherSans-TLF/m/n on
input l
ine 95.
LaTeX Font Info:    Overwriting math alphabet `\mathsf' in version `bold'
(Font)                  OT1/cmss/bx/n --> T1/MerriweatherSans-TLF/m/n on
input
```

```
line 95.
LaTeX Font Info:    Redeclaring math alphabet \mathit on input line 95.
LaTeX Font Info:    Overwriting math alphabet `\mathit' in version
`normal'
(Font)                  OT1/cmr/m/it --> T1/Merriweather-OsF/m/it on
input line
 95.
LaTeX Font Info:    Overwriting math alphabet `\mathit' in version `bold'
(Font)                  OT1/cmr/bx/it --> T1/Merriweather-OsF/m/it on
input lin
e 95.
LaTeX Font Info:    Redeclaring math alphabet \mathtt on input line 95.
LaTeX Font Info:    Overwriting math alphabet `\mathtt' in version
`normal'
(Font)                  OT1/cmtt/m/n --> T1/lmtt/m/n on input line 95.
LaTeX Font Info:    Overwriting math alphabet `\mathtt' in version `bold'
(Font)                  OT1/cmtt/m/n --> T1/lmtt/m/n on input line 95.
LaTeX Font Info:    Overwriting math alphabet `\mathsf' in version `bold'
(Font)                  T1/MerriweatherSans-TLF/m/n -->
T1/MerriweatherSans-TLF
/bx/n on input line 95.
LaTeX Font Info:    Overwriting math alphabet `\mathit' in version `bold'
(Font)                  T1/Merriweather-OsF/m/it --> T1/Merriweather-
OsF/bx/it
on input line 95.
\c@mv@tabular=\count317
\c@mv@boldtabular=\count318
ABD: EverySelectfont initializing macros
LaTeX Info: Redefining \selectfont on input line 95.
(c:/TeXLive/2018/texmf-dist/tex/context/base/mkii/supp-pdf.mkii
[Loading MPS to PDF converter (version 2006.09.02).]
\scratchcounter=\count319
\scratchdimen=\dimen270
\scratchbox=\box67
\nofMPsegments=\count320
\nofMParguments=\count321
\everyMPshowfont=\toks38
\MPscratchCnt=\count322
\MPscratchDim=\dimen271
\MPnumerator=\count323
\makeMPintoPDFobject=\count324
\everyMPtoPDFconversion=\toks39
) (c:/TeXLive/2018/texmf-dist/tex/latex/oberdiek/epstopdf-base.sty
Package: epstopdf-base 2016/05/15 v2.6 Base part for package epstopdf
(c:/TeXLive/2018/texmf-dist/tex/latex/oberdiek/grfext.sty
Package: grfext 2016/05/16 v1.2 Manage graphics extensions (HO)
)
Package epstopdf-base Info: Redefining graphics rule for `.eps' on input
line 4
38.
Package grfext Info: Graphics extension search list:
(grfext)
[.pdf,.png,.jpg,.mps,.jpeg,.jbig2,.jb2,.PDF,.PNG,.JPG,.JPE
G,.JBIG2,.JB2,.eps]
```

```
(grfext)             \AppendGraphicsExtensions on input line 456.
(c:/TeXLive/2018/texmf-dist/tex/latex/latexconfig/epstopdf-sys.cfg
File: epstopdf-sys.cfg 2010/07/13 v1.3 Configuration of (r)epstopdf for
TeX Liv
e
))
Package lastpage Info: Please have a look at the pageslts package at
(lastpage)             https://www.ctan.org/pkg/pageslts
(lastpage)             ! on input line 95.
ABD: EveryShipout initializing macros
\AtBeginShipoutBox=\box68
*geometry* driver: auto-detecting
*geometry* detected driver: pdftex
*geometry* verbose mode - [ preamble ] result:
* driver: pdftex
* paper: a4paper
* layout: <same size as paper>
* layoutoffset:(h,v)=(0.0pt,0.0pt)
* modes: includefoot twoside
* h-part:(L,W,R)=(54.64pt, 488.22787pt, 54.64pt)
* v-part:(T,H,B)=(66.0pt, 745.04684pt, 34.0pt)
* \paperwidth=597.50787pt
* \paperheight=845.04684pt
* \textwidth=488.22787pt
* \textheight=715.04684pt
* \oddsidemargin=-17.62999pt
* \evensidemargin=-17.62999pt
* \topmargin=-47.76999pt
* \headheight=17.5pt
* \headsep=24.0pt
* \topskip=10.0pt
* \footskip=30.0pt
* \marginparwidth=48.0pt
* \marginparsep=10.0pt
* \columnsep=18.0pt
* \skip\footins=22.0pt plus 2.0pt
* \hoffset=0.0pt
* \voffset=0.0pt
* \mag=1000
* \@twocolumntrue
* \@twosidetrue
* \@mparswitchtrue
* \@reversemarginfalse
* (1in=72.27pt=25.4mm, 1cm=28.453pt)

Package hyperref Info: Link coloring ON on input line 95.
(c:/TeXLive/2018/texmf-dist/tex/latex/hyperref/nameref.sty
Package: nameref 2016/05/21 v2.44 Cross-referencing by name of section
(c:/TeXLive/2018/texmf-dist/tex/generic/oberdiek/gettitlestring.sty
Package: gettitlestring 2016/05/16 v1.5 Cleanup title references (HO)
)
\c@section@level=\count325
)
LaTeX Info: Redefining \ref on input line 95.
```

```
LaTeX Info: Redefining \pageref on input line 95.
LaTeX Info: Redefining \nameref on input line 95.
(./main.out) (./main.out)
\@outlinefile=\write3
\openout3 = `main.out'.

\@gscitedetails=\box69
\@gscitedetailsheight=\skip167
\@gsheadbox=\box70
\@gsheadboxheight=\skip168
LaTeX Font Info:    Font shape `T1/Merriweather-OsF/b/n' will be
(Font)              scaled to size 6.5pt on input line 95.
LaTeX Font Info:    Calculating math sizes for size <7.5> on input line
95.
LaTeX Font Info:    Font shape `T1/Merriweather-OsF/m/n' will be
(Font)              scaled to size 6.24973pt on input line 95.
LaTeX Font Info:    Font shape `T1/Merriweather-OsF/m/n' will be
(Font)              scaled to size 5.24997pt on input line 95.
LaTeX Font Info:    Try loading font information for U+eur on input line
95.
(c:/TeXLive/2018/texmf-dist/tex/latex/amsfonts/ueur.fd
File: ueur.fd 2013/01/14 v3.01 Euler Roman
) (c:/TeXLive/2018/texmf-dist/tex/latex/microtype/mt-eur.cfg
File: mt-eur.cfg 2006/07/31 v1.1 microtype config. file: AMS Euler Roman
(RS)
)

LaTeX Font Warning: Font shape `OMS/cmsy/m/n' in size <7.5> not available
(Font)              size <7> substituted on input line 95.

LaTeX Font Info:    External font `cmex10' loaded for size
(Font)              <7.5> on input line 95.
LaTeX Font Info:    External font `cmex10' loaded for size
(Font)              <6.24973> on input line 95.
LaTeX Font Info:    External font `cmex10' loaded for size
(Font)              <5.24997> on input line 95.
LaTeX Font Info:    Try loading font information for U+euf on input line
95.
(c:/TeXLive/2018/texmf-dist/tex/latex/amsfonts/ueuf.fd
File: ueuf.fd 2013/01/14 v3.01 Euler Fraktur
) (c:/TeXLive/2018/texmf-dist/tex/latex/microtype/mt-euf.cfg
File: mt-euf.cfg 2006/07/03 v1.1 microtype config. file: AMS Euler
Fraktur (RS)

)
LaTeX Font Info:    Try loading font information for U+eus on input line
95.
(c:/TeXLive/2018/texmf-dist/tex/latex/amsfonts/ueus.fd
File: ueus.fd 2013/01/14 v3.01 Euler Script
) (c:/TeXLive/2018/texmf-dist/tex/latex/microtype/mt-eus.cfg
File: mt-eus.cfg 2006/07/28 v1.2 microtype config. file: AMS Euler Script
(RS)
)
```

```
LaTeX Font Info:    Try loading font information for U+euex on input line
95.
(c:/TeXLive/2018/texmf-dist/tex/latex/amsfonts/ueuex.fd
File: ueuex.fd 2013/01/14 v3.01 Euler extra symbols
)

LaTeX Font Warning: Font shape `OML/cmm/m/it' in size <7.5> not available
(Font)              size <7> substituted on input line 95.

LaTeX Font Info:    Font shape `T1/Merriweather-OsF/m/it' will be
(Font)              scaled to size 7.5pt on input line 95.
LaTeX Font Info:    Font shape `T1/Merriweather-OsF/m/it' will be
(Font)              scaled to size 6.24973pt on input line 95.
LaTeX Font Info:    Font shape `T1/Merriweather-OsF/m/it' will be
(Font)              scaled to size 5.24997pt on input line 95.
LaTeX Font Info:    Font shape `T1/Merriweather-OsF/m/n' will be
(Font)              scaled to size 8.0pt on input line 95.
LaTeX Font Info:    Font shape `T1/Merriweather-OsF/m/it' will be
(Font)              scaled to size 8.0pt on input line 95.
LaTeX Font Info:    Font shape `T1/Merriweather-OsF/b/it' will be
(Font)              scaled to size 8.0pt on input line 95.
Package caption Info: Begin \AtBeginDocument code.
Package caption Info: listings package is loaded.
Package caption Info: End \AtBeginDocument code.

(c:/TeXLive/2018/texmf-dist/tex/latex/translator/translator-basic-
dictionary-En
glish.dict
Dictionary: translator-basic-dictionary, Language: English
) (c:/TeXLive/2018/texmf-dist/tex/latex/siunitx/siunitx-abbreviations.cfg
File: siunitx-abbreviations.cfg 2017/11/26 v2.7k siunitx: Abbreviated
units
)
\c@lstlisting=\count326
LaTeX Font Info:    Try loading font information for T1+MerriweatherSans-
TLF on
 input line 95.
(c:/TeXLive/2018/texmf-dist/tex/latex/merriweather/T1MerriweatherSans-
TLF.fd
File: T1MerriweatherSans-TLF.fd 2014/01/22 (autoinst) Font definitions
for T1/M
erriweatherSans-TLF.
)
LaTeX Font Info:    Font shape `T1/MerriweatherSans-TLF/m/n' will be
(Font)              scaled to size 7.5pt on input line 95.
Package microtype Info: Loading generic protrusion settings for font
family
(microtype)             `MerriweatherSans-TLF' (encoding: T1).
(microtype)             For optimal results, create family-specific
settings.
(microtype)             See the microtype manual for details.
LaTeX Font Info:    Font shape `T1/MerriweatherSans-TLF/m/n' will be
(Font)              scaled to size 6.24973pt on input line 95.
LaTeX Font Info:    Font shape `T1/MerriweatherSans-TLF/m/n' will be
```

```
(Font)              scaled to size 5.24997pt on input line 95.
LaTeX Font Info:    Try loading font information for T1+lmtt on input
line 95.
(c:/TeXLive/2018/texmf-dist/tex/latex/lm/t1lmtt.fd
File: t1lmtt.fd 2009/10/30 v1.6 Font defs for Latin Modern
)
Package microtype Info: Loading generic protrusion settings for font
family
(microtype)                `lmtt' (encoding: T1).
(microtype)                For optimal results, create family-specific
settings.
(microtype)                See the microtype manual for details.
TextBlockOrigin set to 4pc+6.64pt x 4pc+6pt
<oup.pdf, id=109, 49.18375pt x 48.18pt>
File: oup.pdf Graphic file (type pdf)
<use oup.pdf>
Package pdftex.def Info: oup.pdf  used on input line 114.
(pdftex.def)             Requested size: 59.38191pt x 58.17038pt.
<gigascience-logo.pdf, id=110, 99.37125pt x 33.12375pt>
File: gigascience-logo.pdf Graphic file (type pdf)
<use gigascience-logo.pdf>
Package pdftex.def Info: gigascience-logo.pdf  used on input line 114.
(pdftex.def)             Requested size: 126.00902pt x 42.0pt.

Overfull \hbox (54.64pt too wide) in paragraph at lines 114--114
[][]
 []

LaTeX Font Info:    Font shape `T1/Merriweather-OsF/m/n' will be
(Font)              scaled to size 14.0pt on input line 114.
LaTeX Font Info:    Font shape `T1/Merriweather-OsF/m/n' will be
(Font)              scaled to size 8.99997pt on input line 114.
LaTeX Font Info:    Calculating math sizes for size <14> on input line
114.
LaTeX Font Info:    Font shape `T1/Merriweather-OsF/m/n' will be
(Font)              scaled to size 11.66617pt on input line 114.
LaTeX Font Info:    Font shape `T1/Merriweather-OsF/m/n' will be
(Font)              scaled to size 9.79996pt on input line 114.
LaTeX Font Info:    External font `cmex10' loaded for size
(Font)              <14> on input line 114.
LaTeX Font Info:    External font `cmex10' loaded for size
(Font)              <11.66617> on input line 114.
LaTeX Font Info:    External font `cmex10' loaded for size
(Font)              <9.79996> on input line 114.
LaTeX Font Info:    Font shape `T1/Merriweather-OsF/m/it' will be
(Font)              scaled to size 14.0pt on input line 114.
LaTeX Font Info:    Font shape `T1/Merriweather-OsF/m/it' will be
(Font)              scaled to size 11.66617pt on input line 114.
LaTeX Font Info:    Font shape `T1/Merriweather-OsF/m/it' will be
(Font)              scaled to size 9.79996pt on input line 114.
LaTeX Font Info:    Font shape `T1/MerriweatherSans-TLF/m/n' will be
(Font)              scaled to size 14.0pt on input line 114.
LaTeX Font Info:    Font shape `T1/MerriweatherSans-TLF/m/n' will be
(Font)              scaled to size 11.66617pt on input line 114.
```

```
LaTeX Font Info:    Font shape `T1/MerriweatherSans-TLF/m/n' will be
(Font)              scaled to size 9.79996pt on input line 114.
LaTeX Font Info:    Font shape `T1/Merriweather-OsF/b/n' will be
(Font)              scaled to size 18.0pt on input line 114.
LaTeX Font Info:    Font shape `T1/Merriweather-OsF/m/n' will be
(Font)              scaled to size 13.0pt on input line 114.
LaTeX Font Info:    Calculating math sizes for size <13> on input line
114.
LaTeX Font Info:    Font shape `T1/Merriweather-OsF/m/n' will be
(Font)              scaled to size 10.83287pt on input line 114.
LaTeX Font Info:    Font shape `T1/Merriweather-OsF/m/n' will be
(Font)              scaled to size 9.09996pt on input line 114.

LaTeX Font Warning: Font shape `OMS/cmsy/m/n' in size <13> not available
(Font)              size <12> substituted on input line 114.

LaTeX Font Info:    External font `cmex10' loaded for size
(Font)              <13> on input line 114.
LaTeX Font Info:    External font `cmex10' loaded for size
(Font)              <10.83287> on input line 114.
LaTeX Font Info:    External font `cmex10' loaded for size
(Font)              <9.09996> on input line 114.

LaTeX Font Warning: Font shape `OML/cmm/m/it' in size <13> not available
(Font)              size <12> substituted on input line 114.

LaTeX Font Info:    Font shape `T1/Merriweather-OsF/m/it' will be
(Font)              scaled to size 13.0pt on input line 114.
LaTeX Font Info:    Font shape `T1/Merriweather-OsF/m/it' will be
(Font)              scaled to size 10.83287pt on input line 114.
LaTeX Font Info:    Font shape `T1/Merriweather-OsF/m/it' will be
(Font)              scaled to size 9.09996pt on input line 114.
LaTeX Font Info:    Font shape `T1/MerriweatherSans-TLF/m/n' will be
(Font)              scaled to size 13.0pt on input line 114.
LaTeX Font Info:    Font shape `T1/MerriweatherSans-TLF/m/n' will be
(Font)              scaled to size 10.83287pt on input line 114.
LaTeX Font Info:    Font shape `T1/MerriweatherSans-TLF/m/n' will be
(Font)              scaled to size 9.09996pt on input line 114.
LaTeX Font Info:    Try loading font information for TS1+Merriweather-OsF
on in
put line 114.
(c:/TeXLive/2018/texmf-dist/tex/latex/merriweather/TS1Merriweather-OsF.fd
File: TS1Merriweather-OsF.fd 2014/01/22 (autoinst) Font definitions for
TS1/Mer
riweather-OsF.
)
LaTeX Font Info:    Font shape `TS1/Merriweather-OsF/m/n' will be
(Font)              scaled to size 10.83287pt on input line 114.
Package microtype Info: Loading generic protrusion settings for font
family
(microtype)                `Merriweather-OsF' (encoding: TS1).
(microtype)                For optimal results, create family-specific
settings.
(microtype)                See the microtype manual for details.
```

```
LaTeX Font Info:    Font shape `T1/Merriweather-OsF/m/n' will be
(Font)              scaled to size 9.0pt on input line 114.
LaTeX Font Info:    Font shape `T1/Merriweather-OsF/m/n' will be
(Font)              scaled to size 7.0pt on input line 114.
LaTeX Font Info:    Font shape `T1/Merriweather-OsF/m/n' will be
(Font)              scaled to size 5.0pt on input line 114.
LaTeX Font Info:    External font `cmex10' loaded for size
(Font)              <9> on input line 114.
LaTeX Font Info:    External font `cmex10' loaded for size
(Font)              <7> on input line 114.
LaTeX Font Info:    External font `cmex10' loaded for size
(Font)              <5> on input line 114.
LaTeX Font Info:    Font shape `T1/Merriweather-OsF/m/it' will be
(Font)              scaled to size 9.0pt on input line 114.
LaTeX Font Info:    Font shape `T1/Merriweather-OsF/m/it' will be
(Font)              scaled to size 7.0pt on input line 114.
LaTeX Font Info:    Font shape `T1/Merriweather-OsF/m/it' will be
(Font)              scaled to size 5.0pt on input line 114.
LaTeX Font Info:    Font shape `T1/MerriweatherSans-TLF/m/n' will be
(Font)              scaled to size 9.0pt on input line 114.
LaTeX Font Info:    Font shape `T1/MerriweatherSans-TLF/m/n' will be
(Font)              scaled to size 7.0pt on input line 114.
LaTeX Font Info:    Font shape `T1/MerriweatherSans-TLF/m/n' will be
(Font)              scaled to size 5.0pt on input line 114.
LaTeX Font Info:    Font shape `T1/Merriweather-OsF/m/n' will be
(Font)              scaled to size 6.5pt on input line 114.
LaTeX Font Info:    Calculating math sizes for size <6.5> on input line
114.
LaTeX Font Info:    Font shape `T1/Merriweather-OsF/m/n' will be
(Font)              scaled to size 5.41643pt on input line 114.
LaTeX Font Info:    Font shape `T1/Merriweather-OsF/m/n' will be
(Font)              scaled to size 4.54997pt on input line 114.

LaTeX Font Warning: Font shape `OMS/cmsy/m/n' in size <6.5> not available
(Font)              size <6> substituted on input line 114.


LaTeX Font Warning: Font shape `OMS/cmsy/m/n' in size <5.41643> not
available
(Font)              size <5> substituted on input line 114.


LaTeX Font Warning: Font shape `OMS/cmsy/m/n' in size <4.54997> not
available
(Font)              size <5> substituted on input line 114.

LaTeX Font Info:    External font `cmex10' loaded for size
(Font)              <6.5> on input line 114.
LaTeX Font Info:    External font `cmex10' loaded for size
(Font)              <5.41643> on input line 114.
LaTeX Font Info:    External font `cmex10' loaded for size
(Font)              <4.54997> on input line 114.

LaTeX Font Warning: Font shape `OML/cmm/m/it' in size <6.5> not available
```

```
(Font)              size <6> substituted on input line 114.


LaTeX Font Warning: Font shape `OML/cmm/m/it' in size <5.41643> not
available
(Font)              size <5> substituted on input line 114.


LaTeX Font Warning: Font shape `OML/cmm/m/it' in size <4.54997> not
available
(Font)              size <5> substituted on input line 114.

LaTeX Font Info:    Font shape `T1/Merriweather-OsF/m/it' will be
(Font)              scaled to size 6.5pt on input line 114.
LaTeX Font Info:    Font shape `T1/Merriweather-OsF/m/it' will be
(Font)              scaled to size 5.41643pt on input line 114.
LaTeX Font Info:    Font shape `T1/Merriweather-OsF/m/it' will be
(Font)              scaled to size 4.54997pt on input line 114.
LaTeX Font Info:    Font shape `T1/MerriweatherSans-TLF/m/n' will be
(Font)              scaled to size 6.5pt on input line 114.
LaTeX Font Info:    Font shape `T1/MerriweatherSans-TLF/m/n' will be
(Font)              scaled to size 5.41643pt on input line 114.
LaTeX Font Info:    Font shape `T1/MerriweatherSans-TLF/m/n' will be
(Font)              scaled to size 4.54997pt on input line 114.
LaTeX Font Info:    Font shape `TS1/Merriweather-OsF/m/n' will be
(Font)              scaled to size 5.41643pt on input line 114.

Overfull \hbox (54.64pt too wide) in paragraph at lines 114--114
[][][]
 []

LaTeX Font Info:    Font shape `T1/Merriweather-OsF/b/n' will be
(Font)              scaled to size 10.0pt on input line 114.
LaTeX Font Info:    Font shape `T1/Merriweather-OsF/b/n' will be
(Font)              scaled to size 8.0pt on input line 114.

Overfull \hbox (54.64pt too wide) in paragraph at lines 114--114
[][][]
 []

LaTeX Font Info:    Font shape `T1/Merriweather-OsF/b/n' will be
(Font)              scaled to size 8.5pt on input line 126.
LaTeX Font Info:    Font shape `T1/Merriweather-OsF/b/n' will be
(Font)              scaled to size 7.5pt on input line 128.

Package natbib Warning: Citation `stephens2015big' on page 1 undefined on
input
 line 128.


Package natbib Warning: Citation `foster2017intersection' on page 1
undefined o
n input line 128.
```

```
Package natbib Warning: Citation `peters2018phenomenal' on page 1
undefined on
input line 128.


Package natbib Warning: Citation `peng2008bioimage' on page 1 undefined
on inpu
t line 128.


Package natbib Warning: Citation `brown2018big' on page 1 undefined on
input li
ne 128.


Package natbib Warning: Citation `cook2018european' on page 1 undefined
on inpu
t line 128.


Package natbib Warning: Citation `tan2012delay' on page 1 undefined on
input li
ne 134.


Package natbib Warning: Citation `still2015gearing' on page 1 undefined
on inpu
t line 134.


Package natbib Warning: Citation `convolbo2018geodis' on page 1 undefined
on in
put line 134.


Package natbib Warning: Citation `fox2009above' on page 1 undefined on
input li
ne 134.


Package natbib Warning: Citation `mansouri2018data' on page 1 undefined
on inpu
t line 134.


Package natbib Warning: Citation `williams2016growing' on page 1
undefined on i
nput line 136.


Underfull \vbox (badness 1859) has occurred while \output is active []
```

```
Underfull \vbox (badness 10000) has occurred while \output is active []

LaTeX Font Info:    Font shape `T1/Merriweather-OsF/m/n' will be
(Font)              scaled to size 7.8pt on input line 137.
LaTeX Font Info:    Font shape `T1/Merriweather-OsF/b/n' will be
(Font)              scaled to size 7.8pt on input line 137.
[1{c:/TeXLive/2018/texmf-var/fonts/map/pdftex/updmap/pdftex.map}


 <./oup.pdf> <./gigascience-logo.pdf>]

Package natbib Warning: Citation `leipzig2017review' on page 2 undefined
on inp
ut line 140.


Package natbib Warning: Citation `lampa2016towards' on page 2 undefined
on inpu
t line 142.


Package natbib Warning: Citation `di2017nextflow' on page 2 undefined on
input
line 142.


Package natbib Warning: Citation `moreno2018galaxy' on page 2 undefined
on inpu
t line 142.


Package natbib Warning: Citation `novella2018container' on page 2
undefined on
input line 142.


Package natbib Warning: Citation `oci2016' on page 2 undefined on input
line 14
2.


Package natbib Warning: Citation `dean2008mapreduce' on page 2 undefined
on inp
ut line 148.


Package natbib Warning: Citation `bhandarkar2010mapreduce' on page 2
undefined
on input line 148.


Package natbib Warning: Citation `gunarathne2010mapreduce' on page 2
undefined
on input line 148.
```

```
Package natbib Warning: Citation `mohammed2014applications' on page 2 
undefined
 on input line 148.


Package natbib Warning: Citation `guo2018bioinformatics' on page 2 
undefined on
 input line 148.


Package natbib Warning: Citation `schonherr2012cloudgene' on page 2 
undefined o
n input line 148.


Package natbib Warning: Citation `zaharia2016apache' on page 2 undefined 
on inp
ut line 151.


Package natbib Warning: Citation `ding2011more' on page 2 undefined on 
input li
ne 153.


Package natbib Warning: Citation `di2017nextflow' on page 2 undefined on 
input
line 153.


Package natbib Warning: Citation `shimel2016docker' on page 2 undefined 
on inpu
t line 156.

LaTeX Font Info:    Font shape `TS1/Merriweather-OsF/m/n' will be
(Font)              scaled to size 7.5pt on input line 161.
LaTeX Font Info:    Font shape `T1/Merriweather-OsF/b/sl' in size <7.5> 
not ava
ilable
(Font)              Font shape `T1/Merriweather-OsF/b/it' tried instead 
on inpu
t line 167.
LaTeX Font Info:    Font shape `T1/Merriweather-OsF/b/it' will be
(Font)              scaled to size 7.5pt on input line 167.

Package natbib Warning: Citation `kudla2006high' on page 2 undefined on 
input l
ine 168.


Package natbib Warning: Citation `ubuntuDocker' on page 2 undefined on 
input li
```

```
ne 168.

Package hyperref Info: bookmark level for unknown lstlisting defaults to
0 on i
nput line 170.
LaTeX Font Info:    Font shape `T1/lmtt/bx/n' in size <7.5> not available
(Font)              Font shape `T1/lmtt/b/n' tried instead on input line
171.

Package natbib Warning: Citation `odersky2004overview' on page 2
undefined on i
nput line 188.


Package natbib Warning: Citation `kluyver2016jupyter' on page 2 undefined
on in
put line 188.


Package natbib Warning: Citation `cheng2018building' on page 2 undefined
on inp
ut line 188.


Package natbib Warning: Citation `zaharia2012resilient' on page 2
undefined on
input line 188.

LaTeX Font Info:    Font shape `T1/Merriweather-OsF/m/it' will be
(Font)              scaled to size 7.8pt on input line 189.
[2]

Package natbib Warning: Citation `zaharia2012resilient' on page 3
undefined on
input line 199.


Package natbib Warning: Citation `hashpartitioner' on page 3 undefined on
input
 line 208.


Package natbib Warning: Citation `peek1998unix' on page 3 undefined on
input li
ne 211.


Package natbib Warning: Citation `tevanian1987unix' on page 3 undefined
on inpu
t line 211.


Package natbib Warning: Citation `snyder1990tmpfs' on page 3 undefined on
input
```

line 211.

Package natbib Warning: Citation `snyder1990tmpfs' on page 3 undefined on input
line 211.

[3]

Package natbib Warning: Citation `cpouta' on page 4 undefined on input line 216
.

Package natbib Warning: Citation `cheng2018building' on page 4 undefined on inp
ut line 216.

Package natbib Warning: Citation `benchmarks' on page 4 undefined on input line
216.

Package natbib Warning: Citation `tf_spark' on page 4 undefined on input line 2
16.

Package natbib Warning: Citation `cheng2012structure' on page 4 undefined on in
put line 226.

Package natbib Warning: Citation `mcgann2011fred' on page 4 undefined on input
line 228.

Package natbib Warning: Citation `sdsorter' on page 4 undefined on input line 2
28.

Package natbib Warning: Citation `dalby1992description' on page 4 undefined on
input line 255.

Package natbib Warning: Citation `benchmarks' on page 4 undefined on input line
255.

```
Package natbib Warning: Citation `backbro1997unexpected' on page 4
undefined on
 input line 255.


Underfull \hbox (badness 1137) in paragraph at lines 257--258
[]\T1/Merriweather-OsF/m/n/7.5 The map phase pro-duces a pose for each
molecule
 in
 []


Package natbib Warning: Citation `papadatos2015surechembl' on page 4
undefined
on input line 259.


Package natbib Warning: Citation `irwin2012zinc' on page 4 undefined on
input l
ine 259.


Package natbib Warning: Citation `mathur2007new' on page 4 undefined on
input l
ine 261.

[4]

Package natbib Warning: Citation `karki2015defining' on page 5 undefined
on inp
ut line 267.


Package natbib Warning: Citation `10002015global' on page 5 undefined on
input
line 269.


Package natbib Warning: Citation `Collins1999' on page 5 undefined on
input lin
e 269.


Package natbib Warning: Citation `Kruglyak1999' on page 5 undefined on
input li
ne 269.


Package natbib Warning: Citation `stephens2015big' on page 5 undefined on
input
 line 271.
```

```
Package natbib Warning: Citation `li2009fast' on page 5 undefined on
input line
 271.


Package natbib Warning: Citation `mckenna2010genome' on page 5 undefined
on inp
ut line 271.


Package natbib Warning: Citation `Cock2010' on page 5 undefined on input
line 3
22.


Package natbib Warning: Citation `Li2009' on page 5 undefined on input
line 322
.


Package natbib Warning: Citation `Li2009' on page 5 undefined on input
line 322
.


Package natbib Warning: Citation `Li2009' on page 5 undefined on input
line 322
.


Package natbib Warning: Citation `danecek2011variant' on page 5 undefined
on in
put line 330.


Package natbib Warning: Citation `gatkdoc' on page 5 undefined on input
line 33
0.


Underfull \vbox (badness 1521) has occurred while \output is active []

[5]

Package natbib Warning: Citation `danecek2011variant' on page 6 undefined
on in
put line 334.


Package natbib Warning: Citation `10002015global' on page 6 undefined on
input
line 338.
```

```
Package natbib Warning: Citation `benchmarks' on page 6 undefined on
input line
 346.


Package natbib Warning: Citation `khanam2015map' on page 6 undefined on
input l
ine 353.


Package natbib Warning: Citation `chaimov2016scaling' on page 6 undefined
on in
put line 357.


Package natbib Warning: Citation `nothaft2015rethinking' on page 6
undefined on
 input line 369.


Package natbib Warning: Citation `adam_pipe' on page 6 undefined on input
line
369.

[6]

Package natbib Warning: Citation `duck2016survey' on page 7 undefined on
input
line 371.


Package natbib Warning: Citation `dahlo2018tracking' on page 7 undefined
on inp
ut line 371.


Package natbib Warning: Citation `mare' on page 7 undefined on input line
375.


Package natbib Warning: Citation `benchmarks' on page 7 undefined on
input line
 375.


Package natbib Warning: Citation `zaharia2010spark' on page 7 undefined
on inpu
t line 380.


Package natbib Warning: Citation `zaharia2016apache' on page 7 undefined
on inp
ut line 380.
```

```
Package natbib Warning: Citation `kluyver2016jupyter' on page 7 undefined
on in
put line 383.


Package natbib Warning: Citation `cheng2018building' on page 7 undefined
on inp
ut line 383.


Package natbib Warning: Citation `kubernetes' on page 7 undefined on
input line
 383.


Package natbib Warning: Citation `hindman2011mesos' on page 7 undefined
on inpu
t line 383.


Package natbib Warning: Citation `yarn' on page 7 undefined on input line
383.


Package natbib Warning: Citation `zaharia2012resilient' on page 7
undefined on
input line 386.


Package natbib Warning: Citation `odersky2004overview' on page 7
undefined on i
nput line 386.


Package natbib Warning: Citation `python' on page 7 undefined on input
line 386
.


Package natbib Warning: Citation `java' on page 7 undefined on input line
386.


Package natbib Warning: Citation `ihaka1996r' on page 7 undefined on
input line
 386.


Package natbib Warning: Citation `hashpartitioner' on page 7 undefined on
input
 line 388.
```

```
Package natbib Warning: Citation `partitioner' on page 7 undefined on
input lin
e 388.


Package natbib Warning: Citation `shimel2016docker' on page 7 undefined
on inpu
t line 394.


Package natbib Warning: Citation `oci2016' on page 7 undefined on input
line 39
4.


Package natbib Warning: Citation `lxc' on page 7 undefined on input line
397.


Package natbib Warning: Citation `kamp2000jails' on page 7 undefined on
input l
ine 397.

[7]

Package natbib Warning: Citation `irwin2012zinc' on page 8 undefined on
input l
ine 420.


Package natbib Warning: Citation `10002015global' on page 8 undefined on
input
line 422.


Underfull \hbox (badness 1515) in paragraph at lines 422--423
[]\T1/Merriweather-OsF/m/n/7.5 The 1KPG [\T1/Merriweather-OsF/b/n/7.5 ?
\T1/Mer
riweather-OsF/m/n/7.5 ] data set sup-port-ing the SNP eval-u-
 []


Package natbib Warning: Citation `gigadb' on page 8 undefined on input
line 424
.


Package natbib Warning: Citation `10002015global' on page 8 undefined on
input
line 432.


Underfull \hbox (badness 1082) in paragraph at lines 438--440
```

```
\T1/Merriweather-OsF/m/n/7.5 Hori-zon 2020 pro-gramme un-der grant agree-
ment n
um-ber
 []


Package natbib Warning: Citation `toor2017snic' on page 8 undefined on
input li
ne 445.

No file main.bbl.
[8]

LaTeX Warning: File `figure_1.pdf' not found on input line 454.


! Package pdftex.def Error: File `figure_1.pdf' not found: using draft
setting.


See the pdftex.def package documentation for explanation.
Type  H <return>  for immediate help.
 ...

l.454 ...aphics[width=0.5\textwidth]{figure_1.pdf}

Try typing  <return>  to proceed.
If that doesn't work, type  X <return>  to quit.

LaTeX Font Info:    Font shape `T1/Merriweather-OsF/m/n' will be
(Font)              scaled to size 6.0pt on input line 455.
LaTeX Font Info:    Font shape `T1/Merriweather-OsF/b/n' will be
(Font)              scaled to size 6.0pt on input line 455.
LaTeX Font Info:    Font shape `T1/Merriweather-OsF/m/it' will be
(Font)              scaled to size 6.0pt on input line 455.

LaTeX Warning: File `figure_2.pdf' not found on input line 461.


! Package pdftex.def Error: File `figure_2.pdf' not found: using draft
setting.


See the pdftex.def package documentation for explanation.
Type  H <return>  for immediate help.
 ...

l.461 ...egraphics[width=\textwidth]{figure_2.pdf}

Try typing  <return>  to proceed.
If that doesn't work, type  X <return>  to quit.


LaTeX Warning: File `figure_3.pdf' not found on input line 468.
```

```
! Package pdftex.def Error: File `figure_3.pdf' not found: using draft
setting.


See the pdftex.def package documentation for explanation.
Type  H <return>  for immediate help.
 ...

l.468 ...aphics[width=0.7\textwidth]{figure_3.pdf}

Try typing  <return>  to proceed.
If that doesn't work, type  X <return>  to quit.


LaTeX Warning: File `figure_4.pdf' not found on input line 475.


! Package pdftex.def Error: File `figure_4.pdf' not found: using draft
setting.


See the pdftex.def package documentation for explanation.
Type  H <return>  for immediate help.
 ...

l.475 ...aphics[width=0.7\textwidth]{figure_4.pdf}

Try typing  <return>  to proceed.
If that doesn't work, type  X <return>  to quit.


LaTeX Warning: File `figure_5.pdf' not found on input line 482.


! Package pdftex.def Error: File `figure_5.pdf' not found: using draft
setting.


See the pdftex.def package documentation for explanation.
Type  H <return>  for immediate help.
 ...

l.482 ...aphics[width=0.7\textwidth]{figure_5.pdf}

Try typing  <return>  to proceed.
If that doesn't work, type  X <return>  to quit.


AED: lastpage setting LastPage
[9] [10] [11] [12] [13]

Package natbib Warning: There were undefined citations.
```

```
Package atveryend Info: Empty hook `BeforeClearDocument' on input line
487.
Package atveryend Info: Empty hook `AfterLastShipout' on input line 487.
(./main.aux)
Package atveryend Info: Executing hook `AtVeryEndDocument' on input line
487.
Package atveryend Info: Executing hook `AtEndAfterFileList' on input line
487.
Package rerunfilecheck Info: File `main.out' has not changed.
(rerunfilecheck)             Checksum:
7C6ED8765A454B96D126E827831881A1;1811.

LaTeX Font Warning: Size substitutions with differences
(Font)              up to 1.0pt have occurred.

 )
Here is how much of TeX's memory you used:
 28291 strings out of 492646
 511105 string characters out of 6133325
 666438 words of memory out of 5000000
 31593 multiletter control sequences out of 15000+600000
 601320 words of font info for 228 fonts, out of 8000000 for 9000
 1141 hyphenation exceptions out of 8191
 64i,12n,102p,2086b,1616s stack positions out of
5000i,500n,10000p,200000b,80000s
{c:/TeXLive/2018/texmf-dist/fonts/enc/dvips/lm/lm-
ec.enc}{c:/TeXLive/2018/tex
mf-
dist/fonts/enc/dvips/merriweather/mwth_jnnjab.enc}{c:/TeXLive/2018/texmf-
dis
t/fonts/enc/dvips/merriweather/mwth_ywgpba.enc}<c:/TeXLive/2018/texmf-
dist/font
s/type1/sorkin/merriweather/Merriweather-Bold.pfb><c:/TeXLive/2018/texmf-
dist/f
onts/type1/sorkin/merriweather/Merriweather-
BoldIt.pfb><c:/TeXLive/2018/texmf-d
ist/fonts/type1/sorkin/merriweather/Merriweather-
Italic.pfb><c:/TeXLive/2018/te
xmf-dist/fonts/type1/sorkin/merriweather/Merriweather-
Regular.pfb><c:/TeXLive/2
018/texmf-
dist/fonts/type1/public/amsfonts/cm/cmsy7.pfb><c:/TeXLive/2018/texmf-
dist/fonts/type1/public/amsfonts/euler/eusm7.pfb><c:/TeXLive/2018/texmf-
dist/fo
nts/type1/public/lm/lmtk10.pfb><c:/TeXLive/2018/texmf-
dist/fonts/type1/public/l
m/lmtt8.pfb>
Output written on main.pdf (13 pages, 244037 bytes).
PDF statistics:
 366 PDF objects out of 1000 (max. 8388607)
 335 compressed objects within 4 object streams
 130 named destinations out of 1000 (max. 500000)
 54491 words of extra memory for PDF output out of 61914 (max. 10000000)
```

Click here to access/download;Manuscript;MaRe___gigascience.pdf

OXFORD (GIGA)^n SCIENCE

GigaScience, 2019, 1–12

doi: xx.xxxx/xxxx
Manuscript in Preparation
Technical Note

TECHNICAL NOTE

MaRe: Processing Big Data with Application Containers on Apache Spark

Marco Capuccini[1,2,*], Martin Dahlö[2,3,4], Salman Toor[1] and Ola Spjuth[2]

[1]Department of Information Technology, Uppsala University, Sweden and [2]Department of Pharmaceutical Biosciences, Uppsala University, Sweden and [3]Science for Life Laboratory, Uppsala University, Sweden and [4]Uppsala Multidisciplinary Center for Advanced Computational Science, Uppsala University, Sweden

[*] m.capuccini@gmail.com

Abstract

Background. Life science is increasingly driven by Big Data analytics, and the MapReduce programming model has been proven successful for data-intensive analyses. However, current MapReduce frameworks offer poor support for reusing existing processing tools in bioinformatics pipelines. Further, these frameworks do not have native support for application containers, which are becoming popular in scientific data processing.
Results. Here we present MaRe, an open-source programming library which introduces support for Docker containers in Apache Spark. Apache Spark and Docker are the MapReduce framework and container engine that have collected the largest open source community, thus MaRe provides interoperability with the cutting-edge software ecosystem. We demonstrate MaRe on two data-intensive applications in life science, showing ease of use and scalability.
Conclusions. MaRe enables scalable data-intensive processing in life science with Apache Spark and application containers. When compared with current best practices, that involve the use of workflow systems, MaRe has the advantage of providing data locality, ingestion from heterogeneous storage systems and interactive processing. MaRe is generally-applicable and available as open source software.

Key words: MapReduce; application containers; Big Data; Apache Spark; workflows.

Findings

Background and purpose

Life science is increasingly driven by Big Data analytics. From genomics, proteomics and metabolomics to bioimaging and drug discovery, scientists need to analyze larger and larger amounts of data [1, 2, 3, 4, 5]. This means that datasets can no longer be stored and processed in a researcher's workstation, but they instead need to be handled on distributed systems, at organization level. For instance, the European Bioinformatics Institute, in Hinxton (United Kingdom), offers a total storage capacity of over 160 petabytes for biologically-significant data [6]. Such amounts of data pose major challenges for scientific analyses. First, there is a need to efficiently scale existing processing tools over massive datasets. In fact, bioinformatics software that was originally developed with the simplistic view of small-scale data, will not scale on distributed computing platforms out of the box. The process of adapting such tools may introduce disruptive changes to the existing codebase, and it is generally unsustainable for most organizations. Secondly, the complexity in programming distributed systems may be hard to cope with for most researchers, who instead need to focus on the biological problem at hand. In addition, as life science is exploratory, scientists increasingly demand being able to run interactive analyses rather than submitting jobs to batch systems. Thirdly, when handling Big Data in distributed systems, data locality is a major concern. Indeed, if once data could be shuffled with little regard, with massive datasets it is not only inefficient [7], but also prohibitively expensive in terms of power consumption – estimated to be in the order of several hundred thousand dollars per year for a single

Compiled on: April 1, 2020.
Draft manuscript prepared by the author.

next-generation High-Performance Computing (HPC) cluster [8]. For geographically dispersed datasets, locality-awareness becomes even more challenging, as computing resources need to be dynamically acquired close to the data [9]. Cloud computing solves this problem by enabling the allocation of virtual infrastructure on demand [10]. However, heterogeneity in storage systems for cloud providers [11], makes it hard to abstract data ingestion from many different sources. Finally, as bioinformatics software is characterized by complex software dependencies, deploying and managing a vast collection of tools in a large distributed system also represents a major challenge [12].

Current bionformatics best practices make use of workflow systems, to orchestrate analyses over distributed computing platforms [13]. Workflow systems provide high-level Application Programming Interfaces (APIs) that allow for defining an execution graph of existing processing tools. At run time, the execution graph is used to pipeline the analysis on distributed cloud or HPC resources. Hence, the parallelization of the analysis is transparently carried out, by executing non-dependent tasks at the same time. Cutting-edge workflow systems, such as Luigi [14], NextFlow [15], Galaxy [16] and Pachyderm [17] allow for running processing tools as application containers. This light-weight packaging technology allows for encapsulating complete software environments, so that distributed systems can run the processing tools with no need of additional dependencies, in an isolated manner [18]. Hence, container-enabled workflow systems provide a fairly easy way to define distributed analyses comprising existing bioinformatics tools, and eliminating the need for managing complex software delivery process and dependency management. Nevertheless, workflow-oriented processing falls short when it comes to Big Data analyses. To the best of the authors knowledge, all of these systems utilize a decoupled shared storage system, for synchronization and intermediate results storage. When dealing with large datasets, this translates to a massive and unnecessary communication in the underlying infrastructure. In addition, workflow systems usually support a limited amount of storage backends, not seldom only POSIX file systems, making it hard to ingest data from heterogeneous cloud resources. Finally, due to their batch-oriented nature, it is also intrinsically hard to enable interactive, exploratory analyses using workflow-oriented frameworks.

Google's MapReduce programming model and its associated implementation pioneered uncomplicated Big Data analytics on distributed computing platforms [19]. When using MapReduce, the analysis is defined in a high-level programming language that hides challenging parallel programming details including fault tolerance, data distribution and locality-aware scheduling. Open-source implementations of MapReduce are well established in industrial and scientific applications [20, 21], and numerous success stories in life science have been reported [22, 23, 24].

Apache Spark has emerged as the project that collected the largest community, in the open-source MapReduce ecosystems [25]. In addition to the MapReduce implementation, Apache Spark also provides increasingly important features, such as in-memory, interactive and stream processing. Furthermore, due to broad collaborations in the open-source community, Apache Spark supports all of the major storage systems, enabling data ingestion from heterogeneous cloud resources. These characteristics are particularly appealing for the case of Big Data in life science. Nevertheless, Apache Spark, and other similar frameworks, offer poor support for composing analyses out of existing processing tools. This is usually limited to calling external programs, which can only access data sequentially, without support for application containers [26]. In fact, the main way of implementing analytics in MapReduce-oriented environments is to code each transformation using one of the available APIs. This way of implementing analyses contrasts with current best practices in bioinformatics, that promote the usage of existing tools as application containers with the goal of improving delivery, interoperability and reproducibility of scientific pipelines [15].

Here we introduce MaRe: an open-source programming library that extends Apache Spark, introducing comprehensive support for external tools and application containers in MapReduce. Similarly to container-enabled workflow systems, MaRe allows to define analyses in a high-level language, in which data transformations are performed by application containers. In addition, MaRe provides seamless management of data locality as well as full interoperability, with the Apache Spark ecosystem. This last point allows MaRe analyses to ingest data from heterogeneous cloud storage systems, and also provides support interactive processing. Finally, by supporting Docker, the de facto standard container engine [27], MaRe is compatible with numerous existing container images.

In summary, the key contributions of the presented work are:

- We introduce MaRe: an open-source MapReduce-oriented programming library for container-based data processing on top of Apache Spark.
- We benchmark MaRe on two data-intensive applications in life science, showing ease of use and scalability.

MaRe

Programming Model

We introduce the MaRe programming model using a simple, yet interesting, example in genomics. A DNA sequence can be represented as a text file written in a language of 4 characters: A,T,G,C. The GC content in a DNA sequence has interesting biological implications; for instance there is evidence that GC-rich genes are expressed more efficiently than GC-poor genes [28]. Hence, within a large DNA sequence it can be interesting to count G and C occurrences. Given an Ubuntu Docker image [29], the task can easily be implemented in MaRe using POSIX tools. Listing 1 shows such implementation.

Listing 1. GC count in MaRe

```
val gcCount = new MaRe(genomeRDD).map(
  inputMountPoint = TextFile("/dna"),
  outputMountPoint = TextFile("/count"),
  imageName = "ubuntu",
  command = """
    grep -o '[GC]' /dna | wc -l > /count
  """
).reduce(
  inputMountPoint = TextFile("/counts"),
  outputMountPoint = TextFile("/sum"),
  imageName = "ubuntu",
  command = """
    awk '{s+=$1} END {print s}' /counts > /sum
  """
)
```

Being based on Apache Spark, MaRe has a similar programming model. The control flow of the analysis is coded in Scala [30], by the program in listing 1. Such program is called *driver* in the Apache Spark terminology. The driver program can be packaged and submitted to a cluster (in batch mode), or executed interactively using a notebook environment such as Jupyter [31] or Apache Zeppelin [32]. Listing 1 starts by instantiating a `MaRe` object, which takes a Resilient Distributed Dataset (RDD) [33], containing the input genome file in text

format. Such RDD can be easily loaded using the Apache Spark API from any of the supported storage backends. The `map` primitive (line 1 to 8) applies a command from the Docker image to each partition of the RDD. In our example we specify the Ubuntu image on line 4, and we use a command that combines `grep` and `wc` to filter and count GC occurrences (on line 6). The partition are mounted in the Docker containers in the configured input mount point (`"/dna"` at line 2), and the command results are loaded back to MaRe from the configured output mount point (`"/count"` on line 3). In the example we use *TextFile* mount points as the input data is in text format. By default, MaRe considers each line in a text file as a separate record, but custom record separators can also be configured using the *TextFile* constructor.

At this point it is important to mention that MaRe can also handle binary files. For such data formats, the driver program should specify mount points of type `BinaryFiles`. In this case, each RDD record is considered as a distinct binary file, thus the specified mount point results in a directory containing multiple files (as opposed to `TextFile` that mounts the records in a single file). We provide an example of the `BinaryFiles` mount point in the evaluation section.

Coming back to listing 1, after applying the `map` primitive, each RDD partition is transformed into a distinct GC count. The `reduce` primitive (line 8 to 15), aggregates the counts in each partition to a cumulative sum. Again, we use mount points of type `TextFile`, to mount the intermediate counts in the containers (`"/counts"` on line 9) and to read back the cumulative sum (`"/sum"` on line 10). The sum is computed using the `awk` command from the Ubuntu image (lines 11 to 14). Finally, the result is returned to the `gcCount` variable at line 1.

From the GC example, the reader may have noticed that our programming model is strongly inspired by MapReduce. In addition, Apache Spark users may have noticed that the GC count problem can easily be solved in pure Spark code. Indeed, the aim of the example is just to provide an easy introduction to MaRe, and two real-world applications are available in the evaluation section.

Apart from `map` and `reduce`, MaRe provides an additional primitive. For real-world applications, we noticed that it is often needed to group dataset records according to a specific logic before applying `map` or `reduce`. For this reason, MaRe also provides a `repartitonBy` primitive, which repartitions the RDD records according to a configurable grouping rule. More specifically, the `repartitonBy` primitive takes into account a user-provided `keyBy` function, which is used to compute a key for each record in the dataset. Then, the repartitioning is performed accordingly so that records with same key end up in the same partition. An example of `repartitonBy` is available in the evaluation section.

Implementation

MaRe comes as a thin layer on top of the RDD API [33], and it relies on Apache Spark to provide important features such as data locality, data ingestion, interactive processing, and fault tolerance. The implementation effort consists of: (i) leveraging the RDD API to implement the MaRe primitives and (ii) handling data between containers and RDD structures.

Primitives. Each instance of a `MaRe` object retains an underlying RDD, which represents an abstraction of a dataset that is partitioned across Apache Spark workers. The `map`, `reduce` and `repartitionBy` primitives utilize the underlying RDD API to operate such dataset.

Figure 1 shows the execution diagram for the `map` primitive. For simplicity, in figure 1 we show a single partition per worker, but in reality workers may retain multiple partitions. This primitive takes an input RDD that is partitioned over N nodes, and it transforms each partition using a Docker container command - thus returning a new RDD'. This logic is implemented using *mapPartitions* from the RDD API. When calling *mapPartitions*, MaRe specifies a lambda expression that: (i) makes the data available in the input mount point, (ii) runs the Docker container and (iii) retrieves the results from the output mount point. When using *mapPartitions*, Apache Spark generates a single stage, thus no data shuffle is performed.

Figure 2 shows the execution diagram for the `reduce` primitive. This primitive takes an input RDD, partitioned over N nodes, and it iteratively aggregates records, reducing the number of partition until an RDD', containing a single result partition, is returned. Again, the input RDD may retain multiple partitions per node. However, as opposed to the `map` primitive, RDD' always contains a single partition when it is returned. Given a user-configured depth K, the records in the RDD are aggregated using a tree-like algorithm. In each of the K levels in the tree, the records within each partitions are first aggregated using a Docker container command. Like the `map` primitive, this first transformation is implemented using *mapPartitions*, from the RDD API. Then, the number of partitions is decreased using *repartition* from the RDD API. This process is repeated K times until one single partition is left. At this point the records within the remaining partition are aggregated again using *mapPartitions* (from the RDD API), and RDD' is returned. A new stage is generated each time *repartition* is used. Hence, `reduce` leads to K data shuffles. For this reason, when aggregating records, the user-provided command should always reduce the size of the partition. In addition, for results consistency, the command should perform an associative and commutative operation. By default MaRe sets K to 2, however the user may chose a higher tree depth when it is not possible to sufficiently reduce the dataset size in one go.

Finally, the `repartitionBy` primitive is implemented by using *keyBy*, and then *repartition* from the RDD API. MaRe uses the user-provided grouping rule with *keyBy*, to compute a key for each RDD record, and then it applies `repartition` in counjuction with *HashPartitioner* [34], which makes sure that records with same key end up in the same partition.

Data Handling. One of the advantages of Apache Spark over other MapReduce-like systems is the ability of retaining data in memory. To achieve this when passing the data to the application containers, there are a few options available: (i) Unix pipes [35], (ii) memory-mapped files [36] and (iii) *tmpfs* [37]. Solution (i) and (ii) are the most memory-efficient as they do not need to materialize the data when passing it to the containers. However, (i) allows to see records only once in a stream-like manner, while (ii) requires the container-wrapped tools to be able to read from a memory-mapped file. Apache Spark loads data in memory sequentially and partiton-wise. Partition size is configurable and often equals to the block size in the underlying storage system. For the Hadoop Distributed File System (HDFS) this value defaults to 128MB, meaning that on a 8-core machine materializing again partitions on an in-memory file system would require 2GB of memory in total – which is usually not a problem on modern data centers. Therefore, to support any wrapped-tool, we decided to start by implementing solution (iii). This means that MaRe uses an in-memory *tmpfs* file system as temporary file space for the input and output mount points. The solution allows to provide a standard POSIX mount point to the containers, while still retaining reasonable performance [37]. However, MaRe also provides users with the option of selecting any other disk-based file system for the temporary mount points. Even if this could in principle edge performance, this can be useful when a dockerized tool does not allow for splitting large partitions in smaller chunks of records – we show an example of this in the evaluation sec-

tion.

Evaluation

We evaluate MaRe on two data-intensive applications in life science. The first application can be decomposed to somewhat independent jobs, where the data assigned to each job can be relatively small. This is where MapReduce-oriented programming libraries such as MaRe excel. Conversely, the second application requires to compute larger chunks of data all at once, thus allowing us to show the performance penalty that is introduced in such case. More in detail we evaluate: (i) how the analyses can be implemented in MaRe and (ii) how the analyses scale over multiple nodes. To the best of our knowledge, no stable Spark-native implementation of the tools presented in the analyses is publicly available, making a fair performance comparison with a system that does not delegate data processing to an external application container unfeasible. To this extent, we would like to add that if such implementation were available there would be no advantage in rewriting the analyses using our programming library.

The scalability experiments were carried out on cPouta: an OpenStack-based cloud service operated by the Information Technology Center for Science (CSC) in Finland [38]. The driver programs were run interactively using an Apache Zeppelin environment [32], and the notebooks were made available to sustain reproducibility [39]. In addition, we also made available a deployment automation that enables to replicate our setup on cPouta, as well as any other OpenStack-based cloud provider [40].

Virtual Screening

Virtual Screening (VS) is a computer-based method to identify potential drug candidates, by evaluating the binding affinity of virtual compounds against a biological target protein [41]. Given a 3D target structure, a molecular docking software is run against a large library of known molecular representations. For each compound in the virtual molecular library the docking software produces a pose, representing the orientation of the molecule in the target structure, and a binding affinity score. The poses with the highest affinity scores can be considered as potential drug leads for the target protein.

VS is data-intensive as molecular libraries usually contain millions of compounds. A simple, yet effective, approach to scale VS consists of: (i) distributing the molecular library over several nodes, (ii) running the docking software in parallel and (iii) aggregating the top-scoring poses. Listing 2 shows how this logic can be implemented in MaRe, using FRED [42] as molecular docking software, and sdsorter [43] to filter the top-scoring poses.

Listing 2. Virtual Screening in MaRe

```
1   val topPosesRDD = new MaRe(libraryRDD).map(
2     inputMountPoint = TextFile("/in.sdf", "\n$$$$\n"),
3     outputMountPoint = TextFile("/out.sdf", "\n$$$$\n"),
4     imageName = "mcapuccini/oe:latest",
5     command = """
6       fred -receptor /var/openeye/hiv1_protease.oeb \
7         -hitlist_size 0 \
8         -conftest none \
9         -dbase /in.sdf \
10        -docked_molecule_file /out.sdf
11    """
12  ).reduce(
13    inputMountPoint = TextFile("/in.sdf", "\n$$$$\n"),
14    outputMountPoint = TextFile("/out.sdf", "\n$$$$\n"),
15    imageName = "mcapuccini/sdsorter:latest",
16    command = """
17      sdsorter -reversesort="FRED Chemgauss4 score" \
18        -keep-tag="FRED Chemgauss4 score" \
19        -nbest=30 \
20        /in.sdf /out.sdf
21    """
22  )
```

In listing 2, we initialize MaRe by passing it a molecular library that was previously loaded as an RDD (`libraryRDD` on line 1). We implement the parallel molecular docking using the `map` primitive. On line 2 and 3, we set input and output mount points as text files, and assuming the library to be in Structure-Data File (SDF) format [44] we use the custom record separator: `"\n$$$$\n"`. On line 4, we specify a Docker image containing FRED. The image is not publicly available as it also contains our FRED license, but the license can be obtained free of charge for research purposes and we provide a *Dockerfile* [39] to build the image. On line 5, we specify the FRED command. We use a HIV-1 protease receptor [45] as target (which is wrapped in the Docker image), and we set: (i) `-hitlist_size 0` to not filter the poses in this stage, (ii) `-conftest none` to consider the input molecules as single conformations, (iii) `-dbase /in.sdf` to read the input molecules from the input mount point and (iv) `-docked_molecule_file /out.sdf` to write the poses to the output mount point.

The map phase produces a pose for each molecule in `libraryRDD`. On line 12, we use the `reduce` primitive to filter the top 30 poses. On line 13 and 14, we set the input and output mount points as we do for the `map` primitive. On line 15, we specify a publicly available Docker image containing sdsorter. On line 16, we specify the sdsorter command, and we set: (i) `-reversesort="FRED Chemgauss4 score"` to sort the poses from highest to lowest FRED score, (ii) `-keep-tag="FRED Chemgauss4 score"` to keep the score in the results, (iii) `-nbest=30` to output the top 30 poses and (iv) `/in.sdf /out.sdf` to read and write from the input mount point and to the output mount point respectively. Please notice that this command performs an associative and commutative operation, thus ensuring correctness in the reduce phase. Finally, the results are returned to `topPosesRDD`, on line 1.

We benchmarked the analysis coded in listing 2 against the SureChEMBL library [46] retrieved from the ZINC database [47], containing ~2.2M molecules. The benchmark ran on top of a stand-alone Apache Spark cluster composed of 1 master and 12 worker nodes. Each node provided 10 cores and 43GB of memory, thus resulting in a total of 120 cores and 516GB of memory. The data was made available to the workers using a co-located HDFS storage. Under these settings, we evaluated the scalability in terms of Weak Scaling Efficiency (WSE). This performance metric shows how the system scale when increasing data and parallelism. To compute the WSEs we first ran the benchmark on 1/12 of the dataset using the dockerized tools on a worker node using their built-in, single-node parallelization. Then, we ran again the pipeline using MaRe on 2/12, 4/12, 6/12, ...and 12/12 of the datasets, using 2, 4, 6, ...and 12 worker nodes respectively. The WSE is then computed as the time for processing 1/12 of the data using the built-in, single-node parallelization, divided by the time for processing N/12 of the data using N nodes (for N=2,4,6,...,12). The ideal case, when doubling the number of nodes, is to be able to process twice as much data in the same amount of time. Hence, a higher WSE indicates better performance.

Figure 3 shows the WSE for the full analysis, when using *tmpfs* and a disk-based, *ext4* file system [48] as temporary mount points. From the experiments it emerges that there is little difference between the two methods in terms of scaling

efficiency – *tmpfs* improved the WSE by 0.02 at most. Indeed, the results in figure 3 indicate very good scalability with a WSE close to ideal for both *tmpfs* and *ext4*. For 120 cores, the full benchmark ran in 2 hours and 21 minutes while 1/12 of the input data was processed by the the built-in, single-node parallelization in 2 hours and 14 minutes – resulting in 0.94 WSE. This means that the overhead introduced by MaRe accounts for only 7 minutes in total.

Finally, to ensure the correctness of the parallelization, we ran sdsorter and FRED on a single core against 1K molecules that we randomly sampled from SureChEMBL, and we compared the results with those produced by the code in listing 2.

Single Nucleotide Polymorphism Calling

A Single Nucleotide Polymorphism (SNP) is a position in a DNA sequence where a single nucleotide (or base pair) is different when compared to another DNA sequence [49]. When considering multiple samples, DNA sequences are usually compared individually to a reference genome: an agreed-upon sequence that is considered to represent an organisms genome. Once each DNA sequence has had its SNPs detected, or *called*, the differences between the samples can be compared.

SNPs are frequently occurring. In fact, in humans roughly every 850th base pair is a SNP [50]. Calling SNPs has several use cases. For instance, SNPs can be used as high-resolution markers when comparing genomic regions between samples [51], as well as indicators of diseases in an individual [52]. Modern high-throughput sequencing methods for reading DNA often make use of a technique called *massively parallel sequencing*, to read sequences longer than ~200 base pairs, with a sufficiently small error rate. This is done by cleaving multiple copies of the source DNA into random fragments (called *reads*) that are small enough to be accurately read, and then by aligning them to a reference genome. The overlapping fragments together form the sequence of the source DNA.

In order to accurately sequence 3 billion bases from a single human individual, 30-fold more reads data needs to be sequenced [1]. This makes SNP calling data-intensive, thus requiring parallelization. A simple MapReduce-oriented approach consists of: (i) distributing the reads across several nodes, (ii) aligning the reads to a reference genome in parallel and (iii) calling the SNPs with respect to the reference genome. The last step requires all the reads from a chromosome to be included in the SNP calling, thus the maximum allowed parallelism is equal to the total number of chromosomes. Listing 3 shows how the described parallelization can be implemented in MaRe, using BWA for the alignment [53] and GATK [54] for the SNP calling. As opposite the VS example, BWA and GATK provide a multithreaded implementation of the algorithms. Therefore, in listing 3, we leverage such implementation for single-node parallelization.

Listing 3. SNP Calling in MaRe

```
1   val snpRDD = new MaRe(readsRDD).map(
2     inputMountPoint = TextFile("/in.fastq"),
3     outputMountPoint = TextFile("/out.sam"),
4     imageName = "mcapuccini/alignment:latest",
5     command = """
6       bwa mem -t 8 \
7         -p /ref/human_g1k_v37.fasta \
8         /in.fastq \
9         | samtools view > /out.sam
10    """
11  ).repartitionBy(
12      keyBy = (sam: String) => parseChromosomeId(sam),
13      numPartitions = numberOfNodes
14  ).map(
15    inputMountPoint = TextFile("/in.sam"),
16    outputMountPoint = BinaryFiles("/out"),
17      imageName = "mcapuccini/alignment:latest",
18      command = """
19        cat /ref/human_g1k_v37.dict /in.sam \
20          > /in.hdr.sam
21        gatk AddOrReplaceReadGroups \
22          --INPUT=/in.hdr.sam \
23          --OUTPUT=/in.hdr.sort.rg.bam \
24          --SORT_ORDER=coordinate \
25          [ ... header options ... ]
26        gatk BuildBamIndex \
27          --INPUT=/in.hdr.sort.rg.bam
28        gatk HaplotypeCallerSpark \
29          -R /ref/human_g1k_v37.fasta \
30          -I /in.hdr.sort.rg.bam \
31          -O /out/${RANDOM}.g.vcf
32        gzip /out/*
33    """
34  ).reduce(
35      inputMountPoint = BinaryFiles("/in"),
36      outputMountPoint = BinaryFiles("/out"),
37      imageName = "opengenomics/vcftools-tools:latest",
38      command = """
39        vcf-concat /in/*.vcf.gz \
40          | gzip -c > /out/merged.${RANDOM}.g.vcf.gz
41      """
42  )
```

In listing 3, MaRe is initialized by passing an RDD containing the reads for a human individual in interleaved FASTQ format[55] (`readsRDD` on line 1). We implement the parallel reads alignment using the `map` primitive. From line 2 to 4, we set the mount points as text files, and we specify a publicly available Docker image containing the necessary software tools. On line 5 we specify the BWA command and we set: (i) `-t 8` to utilize 8 threads, (ii) `-p /ref/human_g1k_v37.fasta` to specify the reference genome location (in the container) and (iii) the input mount point `/in.fastq`. In addition, on line 9 we pipe the results to another software, called `samtools` [56], to convert them from the binary BAM format [56] to the text SAM format [56]. Converting the results to text format makes it easier to parse the chromosome location in the next step.

When calling SNPs, GATK needs to read all of the aligned reads for a certain DNA region. Using chromosomes to define the regions makes sure that no reads will span a region break point – a problem that would need to be handled if chromosomes were to be split in smaller regions. To achieve this we need to: (i) perform a chromosome-wise repartition of the dataset and (ii) allow MaRe to write temporary mount point data to disk. Point (ii) is enabled by setting the `TMPDIR` environment variable to a disk mount, in the Apache Zeppelin configuration. Even if this could potentially edge performance, this is necessary as the full partition size exceeds the *tmpfs* capacity in our worker nodes. Point (i) is implemented by using the `repartitionBy` primitive, on line 11. In particular, we specify a `keyBy` function that parses and returns a the chromosome identifier (on line 12), and a number of partitions that is equal to the number of worker nodes (on line 13).

The `map` primitive (on line 14) uses the chromosome-wise partitioning to perform the SNP calling, with GATK. Since the data is in SAM format, we set the input mount point as text file (line 15). However, since we are going to zip the results before aggregating the SNPs (line 32), we set the output mount point as a binary files directory (`"/out"`, on line 16). On line 17, we set the same Docker image that we used for the initial mapping step and, on line 18, we specify a command that: (i)

prepends the necessary SAM header to the input data (which is available inside the container under `/ref/human_g1k_v37.dic`, on line 19), (ii) coverts the SAM input in BAM format (line 23), (iii) builds an index for the BAM format (line 26) and (iv) runs the multithreaded SNP calling using GATK, producing a Variant Call Format (VCF) file [57] (line 28). A detailed description of the options, used for each command, can be found in the GATK documentation [58].

Finally, to aggregate the SNPs to a single zipped file, we use the `reduce` primitive. In this case we use binary file mount points (lines 35 and 36) and a publicly available image containing the VCFtools software [57] (line 37). On line 39, the specified command uses `vcf-concat` to merge all of the VCF files in the input mount point, and then it zips and writes them to the output mount point (line 40). Since MaRe applies the reduce command iteratively, intermediate partitions will contain multiple files. Therefore, to avoid file-name clashes, we include a random identifier in the command output (`$RANDOM` at line 40).

We benchmarked the analysis in listing 3 against the full individual reads dataset `HG02666` (~30GB compressed FASTQ files), from the 1000 Genomes Project (1KGP) [50]. The benchmark ran on top of a stand-alone Apache Spark cluster composed of 1 master and 14 worker nodes. Each node provided 8 cores and 40GB of memory, thus resulting in a total of 112 cores and 480GB of memory. In addition, since after the chromosome-wise repartitioning, the partition size exceeded the *tmpfs* space in our workers, we used cloud favors with a local Solid State Drive (SSD). This allowed to write and read the temporary mount point data faster when compared to the previous benchmark. The data was made available to the workers using a co-located HDFS storage. Under these settings, we evaluated the scalability in terms of Strong Scaling Efficiency (SSE). This performance metric shows how the system scale when increasing the parallelism while keeping the input size static. We evaluated this benchmark using SSE instead of WSE as there is no trivial way for downsampling the reference genome while keeping the behaviour of the tools unaltered; the algorithms end up taking longer as they perform an exhaustive search when the reference genome is downsampled. To compute the SSEs we first ran the benchmark using the dockerized tools on a worker node with their built-in, single-node parallelization. Then, we ran again the pipeline using MaRe on 6, 8, 10, 12 and 14 worker nodes. Then, let T_1 be the time for running the benchmark using the built-in, single-node parallelization and T_N be the time for running the benchmark using N nodes (for N=6,8,10,12), we computed the SSE as $T_1/(N \times T_N)$ – we did not run on 2 and 4 nodes as the dataset size exceeded the total memory available to the Spark workers in these settings. The ideal case, when doubling the number of nodes, is to be able to run the benchmark twice as fast. Hence, a higher SSE indicates better performance.

Figure 4 shows the SSE for the full analysis. The SSE starts at 0.76 for 48 cores and decreases to 0.59 when running on 112 cores. Even if this does not show optimal performance, as in the VS use case, it still indicates good scalability. Indeed, the full benchmark ran in 3 hours and 24 minutes using MaRe on 112 cores, while it took 28 hours and 14 minutes using the built-in, single-node parallelization – leading to a speedup of 8.3.

The alignment portion of the benchmark uses BWA which allows to input the reads using pipes. It is interesting to compare how the SSE differs when using this input method as opposed to materializing the data on a temporary *ext4* file space. Even though the standard RDD API provides a *pipe* method to do so, as we mentioned previously, this built-in implementation runs the external tool for each RDD record – which would result in a considerable overhead. Instead, we compare the SSE achieved by MaRe with a *pipePartion* method, available in our benchmark repository [39], which pipes entire RDD partitions though a single dockerized tool instance. Figure 5 shows the results of this comparison. Using pipes improved the SSE by ~0.15 when running on 48 and 64 cores, by ~0.08 when running on 80 and 96 cores and by ~0.12 when running on 112 cores. However, this improvement accounted for saving 6 minutes when running on 112 cores, which is negligible as the full analysis (including variant calling) took more than 3 hours to complete in such setting.

Discussion and conclusions

Big Data applications are getting increasing momentum in life science. Data is nowadays stored and processed in distributed systems, often in a geographically dispersed manner. This introduces a layer of complexity that MapReduce frameworks, such as Apache Spark, excel at handling [59]. Container engines, and in particular Docker, are also becoming an essential part of bioinformatics pipelines as they improve delivery, interoperability and reproducibility of scientific analyses. By enabling application containers in MapReduce, MaRe constitutes an important advancement in the scientific data-processing software ecosystem. When compared to current best practices in bioinformatics, relying solely on using workflow systems to orchestrate data pipelines, MaRe has the advantage of providing locality-aware scheduling, transparent ingestion from heterogeneous storage systems and interactivity. As data becomes larger and more globally distributed, we envision scientists to instantiate MaRe close to the data, and perform interactive analyses via cloud-oriented resources. In addition to the interactive mode, MaRe also support batch-oriented processing. This is important as it enables integration with existing bioinformatics pipelines. In practical terms, a packaged MaRe application can be launched by a workflow engine to enable data-intensive phases in a pipeline, and submitted to any of the resource managers supported by the Apache Spark community (including HPC systems [60]).

In the evaluation section we have shown how researchers can easily implement two widely-used applications in life science, using MaRe. Both analyses can be coded in less than 50 lines of code, and they are seamlessly parallelized. The results show near optimal scalability for the VS application with *tmpfs* improving performance over *ext4* only by a negligible factor. The reason why there is no relevant performance improvement in using the former is that the containers running time dominate over the time for materializing data on the temporary file space. Even though this may vary in other applications, in our experience this will often be the case for bioinformatics analyses, not justifying the additional effort in setting up a *tmpfs* space.

Scalability in the SNP calling analysis is reasonably good but far from optimal. The reason for this is that before running the haplotype caller, a reasonable amount of data needs to be shuffled across the nodes as GATK needs to see all of the data for a single chromosome at once in order to function properly, thus causing a large amount of data to be materialized on disk. Such overhead can be partly mitigated by enabling data streams via standard input and output between MaRe and containers – as the results in figure 5 show. This constitutes an area for future improvement, however since GATK is unable to read data from the standard input such improvement would not be directly applicable to the presented use case.

ADAM [61], a genomics data-processing framework built on top of Apache Spark, shows ideal scalability for a few, commonly-used preprocessing steps in genomics pipelines – such as the SNP pipeline that we show in this paper. Nevertheless, in real-world scenarios external software would still

need to be employed to compose end-to-end workflows. Indeed, ADAM itself provides a utility to integrate external tools into its pipelines [62]. Since such utility is based on pipes and it does not support application containers natively, it provides less flexibility if compared to MaRe. Indeed, as MaRe is fully interoperable with Apache Spark, our recommendation for running genomics pipelines would be to use ADAM for the supported preprocessing steps and then MaRe to integrate external tools in the workflow.

The benchmarks that we show in this paper are representative of two classes of problems where the application of MaRe could lead to different results in terms of performance. Materializing data is a necessary to support any containerized tool, but our results show that this edges performance when records in large partitions need to be processed all together. In this case, reimplementing the analyses natively in Spark using the language of choice could lead to better performance – ADAM is a good example of this approach. It is however important to point out that the the effort of reimplementing existing bioinformatics tools is seldom sustainable by research organization. To give the reader an idea of this, ADAM is the product of a large collaboration maintaining thousands of lines of code. Due to the current proliferation and heterogeneity of bioinformatics tools [63, 64], it is hard to imagine that such effort would generally be sustainable for many other applications. To this extent, MaRe stands out as it enables bioinformaticians to develop interoperable, distributed pipelines that scale reasonably well without the need to rewrite the existing codebase.

In conclusion, MaRe provides a MapReduce-oriented model to enable container-based bioinformatics analyses at scale. The project is available on GitHub [65] under an open source license, along with all of the code to reproduce the analyses in the evaluation section [39].

Methods

Apache Spark

Apache Spark is an open source cluster-computing framework, for the analysis of large-scale dataset [66]. The project originally started with the aim of overcoming lack of in-memory processing in traditional MapReduce frameworks. Today, Apache Spark has evolved in a unified analytics engine, encompassing high-level APIs for machine learning, streaming, graph processing and SQL, and it has become the largest open source project in Big Data analytics with over 1000 contributors and over 1000 adopting organizations [25].

Clustering model

The Apache Spark clustering model includes: a driver program, one or more worker nodes and a cluster manager. The driver program is written by the user and controls the flow of the programmed analysis. For interactive analysis the driver program can run in notebooks environments such as Jupyter [31] and Apache Zeppelin [32]. Worker nodes communicate with the driver program, thus executing the distributed analysis as defined by the user. Finally, a cluster manager handles resources in the cluster, allowing for the executing processes to acquire them in the worker nodes. Apache Spark is cluster-manager agnostic and it can run in stand alone settings, as well as on some popular platforms (e.g., Kubernetes [67], Mesos [68] and Hadoop YARN [69]).

Resilient Distributed Datasets

Resilient Distributed Datasets (RDDs) [33] are central in the Apache Spark programming model. RDDs are an abstraction of a dataset that is partitioned across the worker nodes. Hence, partitions can be operated in parallel in a scalable and fault-tolerant manner, and possibly cached in memory for recurrent access. As a unified processing engine, Apache Spark offers support for ingesting RDDs from numerous big-data-oriented storage systems. RDDs can be operated through: Scala [30], Python [70], Java [71] and R [72] APIs. Such APIs expose RDDs as object collections, and they offer high-level methods to transform the datasets.

The *mapPartition* and the *repartition* methods, from the RDD API, are useful to understand the MaRe implementation. The *mapPartition* method is inspired by functional programming languages. It takes as an argument a lambda expression that codes a data transformation, and it applies it to each partition, returning a new RDD. The *repartition* method, as the name suggests, changes the way the dataset records are partitioned across the worker nodes. It can be used to increase and decrease the number of partitions, thus affecting the level of parallelism, and it can also sort records in partitions, according to custom logics. In this case, an additional RDD method, namely *keyBy*, needs to be used to compute a key for each RDD record. Similarly to *mapPartition*, *keyBy* applies a user-provided lambda expression to compute the record keys. Such keys are then used by *repartition* in conjunction with an extension of the *Partitioner* class [34] to assign records to partitions. For instance, when using *HashPartitioner* [73] records with same key always end up in the same RDD partition.

Stages and data locality

RDD methods are lazily applied to the underlying dataset. This means that until something needs to be written to a storage system, or returned to the driver program, nothing is computed. In this way, Apache Spark can build a direct acyclic graph and thus optimize the physical execution plan. A physical execution plan is composed of processing tasks that are organized in stages. Typically, inside each stage the physical execution plan preserves data locality, while between stages a data shuffle occurs. In particular, a sequence of mapPartition methods generate a single stage, giving place to almost no communication in the physical execution plan. In contrast, each time *repartition* is applied to an RDD, a new stage is generated (and data shuffling occurs).

Docker

Docker has emerged as the de-facto standard application container engine [27]. Like Virtual Machines (VMs), application containers enable the encapsulation of software components so that any compliant computer system can execute them with no additional dependencies [18]. The advantage of Docker and similar container engines over virtualization consists of eliminating the need of running an Operating System (OS) for each isolated environment. As opposite to hypervisors, container engines leverage on kernel namespaces to isolate software environments, and thus run containers straight on the host OS. This makes application containers considerably lighter than VMs, enabling a more granular compartmentalization of software components.

Software Delivery

By enabling the encapsulation of entire software stacks, container engines have the potential of considerably simplify application delivery. Engines such as LXC [74] and Jails [75] have been available for almost two decades. Nevertheless, when compared to Docker these systems are poor in terms of software delivery functionalities. This is the reason why software containers popularity exploded only when Docker emerged.

Docker containers can be defined using a text specification

language. Using such language, users compose a *Dockerfile* which is parsed by Docker, and then complied into a Docker image. Docker images can then be released to public or private registries, becoming immediately available over the Internet. Therefore, by running the Docker engine, the end users can conveniently start the released containers locally.

Volumes

When using Docker containers for data processing, volumes play an important role. Indeed, there is a need for a mechanism to pass the input data to the containers, and to retrieve the processed output from the isolated environment. Docker volumes allow for defining shared file spaces between containers and the host OS. Such volumes can be easily created when starting containers, by specifying a mapping between host OS file, or directories, and container mount points. Inside the containers these shared objects simply appear as regular files, or directories, under the specified mount point.

Availability of supporting source code and requirements

Project name: MaRe
Project home page: https://github.com/mcapuccini/MaRe
Operating system(s): Platform independent
Programming language: Scala
Other requirements: Apache Spark and Docker
License: Apache License 2.0
Research Resource Identification Initiative ID: SCR_018069

Availability of supporting data

The data set supporting the VS evaluation in this article is available in the ZINC database [47]. The specific subset that we used is available at: http://zinc12.docking.org/catalogs/surechembl.

The 1KPG [50] data set supporting the SNP evaluation is available on Amazon S3 (s3://1000genomes/phase3/data/HG02666). The relative BioProject accession number is PRJNA28889.

Images, results in tabular format and an archival copy of the code are also available via GigaDB [76].

Declarations

List of abbreviations

1KGP: one thousand genome project; API: application programming interface; CSC: information technology center for science; HDFS: hadoop distributed file system; HPC: high-performance computing; OS: operative system; RDD: resilient distributed dataset; SDF: structure-data file; SNP: single nucleotide polymorphism; VCF: variant call format; VM: virtual machine; VS: virtual screening; WSE: weak scaling efficiency.

Ethics approval and consent to participate

All of the 1KGP data is consented for analysis, publication and distribution. Ethics and consents are extensively explained in the 1KGP publications [50].

Competing interests

The authors declare that they have no competing interests.

Funding

This research was supported by The European Commission's Horizon 2020 programme under grant agreement number 654241 (PhenoMeNal).

Author's contributions

MC and OS conceived the project. MC designed and implemented MaRe. MC and MD carried out the evaluation experiments. MD provided expertise in genomics. ST provided expertise in cloud computing. All authors read and approved the final manuscript.

Acknowledgment

We kindly acknowledge contributions to cloud resources by CSC (https://www.csc.fi), the Nordic e-Infrastructure Collaboration (https://neic.no) and the SNIC Science Cloud [77]. Academic license for docking software was provided by OpenEye Scientific.

References

1. Stephens ZD, Lee SY, Faghri F, Campbell RH, Zhai C, Efron MJ, et al. Big data: astronomical or genomical? PLoS biology 2015;13(7):e1002195.
2. Foster LJ, DeMarco ML, At the Intersection of Proteomics and Big Data Science. Clinical Chemistry; 2017.
3. Peters K, Bradbury J, Bergmann S, Capuccini M, Cascante M, de Atauri P, et al. PhenoMeNal: Processing and analysis of Metabolomics data in the Cloud. GigaScience 2018;8(2):giy149.
4. Peng H. Bioimage informatics: a new area of engineering biology. Bioinformatics 2008;24(17):1827–1836.
5. Brown N, Cambruzzi J, Cox PJ, Davies M, Dunbar J, Plumbley D, et al. Big Data in Drug Discovery. In: Progress in medicinal chemistry, vol. 57 Elsevier; 2018.p. 277–356.
6. Cook CE, Lopez R, Stroe O, Cochrane G, Brooksbank C, Birney E, et al. The European Bioinformatics Institute in 2018: tools, infrastructure and training. Nucleic acids research 2018;47(D1):D15–D22.
7. Tan J, Meng X, Zhang L. Delay tails in MapReduce scheduling. ACM SIGMETRICS Performance Evaluation Review 2012;40(1):5–16.
8. Gearing Up for the Next Challenge in High-Performance Computing;. Accessed: 2019-04-25. https://str.llnl.gov/march-2015/still.
9. Convolbo MW, Chou J, Hsu CH, Chung YC. GEODIS: towards the optimization of data locality-aware job scheduling in geo-distributed data centers. Computing 2018;100(1):21–46.
10. Fox A, Griffith R, Joseph A, Katz R, Konwinski A, Lee G, et al. Above the clouds: A berkeley view of cloud computing. Dept Electrical Eng and Comput Sciences, University of California, Berkeley, Rep UCB/EECS 2009;28(13):2009.
11. Mansouri Y, Toosi AN, Buyya R. Data storage management in cloud environments: Taxonomy, survey, and future directions. ACM Computing Surveys (CSUR) 2018;50(6):91.
12. Williams CL, Sica JC, Killen RT, Balis UG. The growing need for microservices in bioinformatics. Journal of Pathology Informatics 2016;7.
13. Leipzig J. A review of bioinformatic pipeline frameworks. Briefings in bioinformatics 2017;18(3):530–536.
14. Lampa S, Alvarsson J, Spjuth O. Towards agile large-scale predictive modelling in drug discovery with flow-based

programming design principles. Journal of cheminformatics 2016;8(1):67.
15. Di Tommaso P, Chatzou M, Floden EW, Barja PP, Palumbo E, Notredame C. Nextflow enables reproducible computational workflows. Nature biotechnology 2017;35(4):316.
16. Moreno P, Pireddu L, Roger P, Goonasekera N, Afgan E, Van Den Beek M, et al. Galaxy-Kubernetes integration: scaling bioinformatics workflows in the cloud. BioRxiv 2018;p. 488643.
17. Novella JA, Emami Khoonsari P, Herman S, Whitenack D, Capuccini M, Burman J, et al. Container-based bioinformatics with Pachyderm. Bioinformatics 2018;35(5):839–846.
18. Open Container Initiative, The 5 principles of Standard Containers; 2016. Accessed: 2019-04-25. https://github.com/opencontainers/runtime-spec/blob/master/principles.md.
19. Dean J, Ghemawat S. MapReduce: simplified data processing on large clusters. Communications of the ACM 2008;51(1):107–113.
20. Bhandarkar M. MapReduce programming with apache Hadoop. In: 2010 IEEE International Symposium on Parallel & Distributed Processing (IPDPS) IEEE; 2010. p. 1–1.
21. Gunarathne T, Wu TL, Qiu J, Fox G. MapReduce in the Clouds for Science. In: 2010 IEEE second international conference on cloud computing technology and science IEEE; 2010. p. 565–572.
22. Mohammed EA, Far BH, Naugler C. Applications of the MapReduce programming framework to clinical big data analysis: current landscape and future trends. BioData mining 2014;7(1):22.
23. Guo R, Zhao Y, Zou Q, Fang X, Peng S. Bioinformatics applications on apache spark. GigaScience 2018;7(8):giy098.
24. Schönherr S, Forer L, Weißensteiner H, Kronenberg F, Specht G, Kloss-Brandstätter A. Cloudgene: A graphical execution platform for MapReduce programs on private and public clouds. BMC bioinformatics 2012;13(1):200.
25. Zaharia M, Xin RS, Wendell P, Das T, Armbrust M, Dave A, et al. Apache spark: a unified engine for big data processing. Communications of the ACM 2016;59(11):56–65.
26. Ding M, Zheng L, Lu Y, Li L, Guo S, Guo M. More convenient more overhead: the performance evaluation of hadoop streaming. In: Proceedings of the 2011 ACM Symposium on Research in Applied Computation ACM; 2011. p. 307–313.
27. Shimel A, Docker becomes de facto Linux standard; 2016. Accessed: 2019-04-25. http://www.networkworld.com/article/2226751/opensource-subnet/docker-becomes-de-facto-linux-standard.html.
28. Kudla G, Lipinski L, Caffin F, Helwak A, Zylicz M. High guanine and cytosine content increases mRNA levels in mammalian cells. PLoS biology 2006;4(6):e180.
29. Ubuntu Docker Image;. Accessed: 2019-04-25. https://hub.docker.com/_/ubuntu.
30. Odersky M, Altherr P, Cremet V, Emir B, Maneth S, Micheloud S, et al. An overview of the Scala programming language; 2004.
31. Kluyver T, Ragan-Kelley B, Pérez F, Granger BE, Bussonnier M, Frederic J, et al. Jupyter Notebooks-a publishing format for reproducible computational workflows. In: ELPUB; 2016. p. 87–90.
32. Cheng Y, Liu FC, Jing S, Xu W, Chau DH. Building big data processing and visualization pipeline through apache zeppelin. In: Proceedings of the Practice and Experience on Advanced Research Computing ACM; 2018. p. 57.
33. Zaharia M, Chowdhury M, Das T, Dave A, Ma J, McCauley M, et al. Resilient distributed datasets: A fault-tolerant abstraction for in-memory cluster computing. In: Proceedings of the 9th USENIX conference on Networked Systems Design and Implementation USENIX Association; 2012. p. 2–2.
34. Laskowski J, HashPartitioner;. Accessed: 2019-04-25. https://jaceklaskowski.gitbooks.io/mastering-apache-spark/spark-rdd-HashPartitioner.html.
35. Peek J, O'Reilly T, Loukides M. UNIX power tools 1998;.
36. Tevanian A, Rashid RF, Young M, Golub DB, Thompson MR, Bolosky WJ, et al. A UNIX Interface for Shared Memory and Memory Mapped Files Under Mach. In: USENIX Summer Citeseer; 1987. p. 53–68.
37. Snyder P. tmpfs: A virtual memory file system. In: Proceedings of the autumn 1990 EUUG Conference; 1990. p. 241–248.
38. cPouta IaaS Cloud;. Accessed: 2019-04-25. https://research.csc.fi/cpouta.
39. MaRe Benchmarks;. Accessed: 2019-04-25. https://github.com/mcapuccini/mare-benchmarks.
40. OpenStack Apache Spark Terraform Module;. Accessed: 2019-04-25. https://github.com/mcapuccini/terraform-openstack-spark.
41. Cheng T, Li Q, Zhou Z, Wang Y, Bryant SH. Structure-based virtual screening for drug discovery: a problem-centric review. The AAPS journal 2012;14(1):133–141.
42. McGann M. FRED pose prediction and virtual screening accuracy. Journal of chemical information and modeling 2011;51(3):578–596.
43. sdsorter;. Accessed: 2019-04-25. https://sourceforge.net/projects/sdsorter.
44. Dalby A, Nourse JG, Hounshell WD, Gushurst AK, Grier DL, Leland BA, et al. Description of several chemical structure file formats used by computer programs developed at Molecular Design Limited. Journal of chemical information and computer sciences 1992;32(3):244–255.
45. Bäckbro K, Löwgren S, Österlund K, Atepo J, Unge T, Hultén J, et al. Unexpected binding mode of a cyclic sulfamide HIV-1 protease inhibitor. Journal of medicinal chemistry 1997;40(6):898–902.
46. Papadatos G, Davies M, Dedman N, Chambers J, Gaulton A, Siddle J, et al. SureChEMBL: a large-scale, chemically annotated patent document database. Nucleic acids research 2015;44(D1):D1220–D1228.
47. Irwin JJ, Sterling T, Mysinger MM, Bolstad ES, Coleman RG. ZINC: a free tool to discover chemistry for biology. Journal of chemical information and modeling 2012;52(7):1757–1768.
48. Mathur A, Cao M, Bhattacharya S, Dilger A, Tomas A, Vivier L. The new ext4 filesystem: current status and future plans. In: Proceedings of the Linux symposium, vol. 2 Citeseer; 2007. p. 21–33.
49. Karki R, Pandya D, Elston RC, Ferlini C. Defining "mutation" and "polymorphism" in the era of personal genomics. BMC medical genomics 2015;8(1):37.
50. Consortium GP, et al. A global reference for human genetic variation. Nature 2015;526(7571):68.
51. Collins FS. Medical and Societal Consequences of the Human Genome Project. New England Journal of Medicine 1999;341(1):28–37. https://doi.org/10.1056/NEJM199907013410106, pMID: 10387940.
52. Kruglyak L. Prospects for whole-genome linkage disequilibrium mapping of common disease genes. Nature Genetics 1999;22(2):139–144. https://doi.org/10.1038/9642.
53. Li H, Durbin R. Fast and accurate short read alignment with Burrows–Wheeler transform. bioinformatics 2009;25(14):1754–1760.
54. McKenna A, Hanna M, Banks E, Sivachenko A, Cibulskis K, Kernytsky A, et al. The Genome Analysis Toolkit: a MapReduce framework for analyzing next-generation DNA se-

quencing data. Genome research 2010;20(9):1297–1303.
55. Cock PJA, Fields CJ, Goto N, Heuer ML, Rice PM. The Sanger FASTQ file format for sequences with quality scores, and the Solexa/Illumina FASTQ variants. Nucleic Acids Res 2010 Apr;38(6):1767–1771. https://www.ncbi.nlm.nih.gov/pubmed/20015970, 20015970[pmid].
56. Li H, Handsaker B, Wysoker A, Fennell T, Ruan J, Homer N, et al. The Sequence Alignment/Map format and SAMtools. Bioinformatics 2009 06;25(16):2078–2079. https://doi.org/10.1093/bioinformatics/btp352.
57. Danecek P, Auton A, Abecasis G, Albers CA, Banks E, DePristo MA, et al. The variant call format and VCFtools. Bioinformatics 2011;27(15):2156–2158.
58. GATK Documentation;. Accessed: 2019-04-25. https://software.broadinstitute.org/gatk/documentation/tooldocs/current.
59. Khanam Z, Agarwal S. Map-reduce implementations: survey and performance comparison. Int J Comput Sci Inf Technol(IJCSIT) 2015;7(4).
60. Chaimov N, Malony A, Canon S, Iancu C, Ibrahim KZ, Srinivasan J. Scaling spark on hpc systems. In: Proceedings of the 25th ACM International Symposium on High-Performance Parallel and Distributed Computing ACM; 2016. p. 97–110.
61. Nothaft FA, Massie M, Danford T, Zhang Z, Laserson U, Yeksigian C, et al. Rethinking data-intensive science using scalable analytics systems. In: Proceedings of the 2015 ACM SIGMOD International Conference on Management of Data; 2015. p. 631–646.
62. ADAM Pipe API;. Accessed: 2019-04-25. https://adam.readthedocs.io/en/latest/api/pipes/.
63. Duck G, Nenadic G, Filannino M, Brass A, Robertson DL, Stevens R. A survey of bioinformatics database and software usage through mining the literature. PloS one 2016;11(6):e0157989.
64. Dahlö M, Scofield DG, Schaal W, Spjuth O. Tracking the NGS revolution: managing life science research on shared high-performance computing clusters. GigaScience 2018 04;7(5). https://doi.org/10.1093/gigascience/giy028.
65. MaRe;. Accessed: 2019-04-25. https://github.com/mcapuccini/MaRe.
66. Zaharia M, Chowdhury M, Franklin MJ, Shenker S, Stoica I. Spark: Cluster computing with working sets. HotCloud 2010;10(10-10):95.
67. Kubernetes;. Accessed: 2019-04-25. https://kubernetes.io.
68. Hindman B, Konwinski A, Zaharia M, Ghodsi A, Joseph AD, Katz RH, et al. Mesos: A platform for fine-grained resource sharing in the data center. In: NSDI, vol. 11; 2011. p. 22–22.
69. Hadoop YARN;. Accessed: 2019-04-25. https://hadoop.apache.org/docs/current/hadoop-yarn/hadoop-yarn-site/YARN.html.
70. The Python Programming Language;. Accessed: 2019-04-25. http://www.python.org.
71. The Java Programming Language;. Accessed: 2019-04-25. https://docs.oracle.com/javase/8/docs/technotes/guides/language/index.html.
72. Ihaka R, Gentleman R. R: a language for data analysis and graphics. Journal of computational and graphical statistics 1996;5(3):299–314.
73. Laskowski J, Partitioner;. Accessed: 2019-04-25. https://jaceklaskowski.gitbooks.io/mastering-apache-spark/spark-rdd-partitions.html.
74. LXC;. Accessed: 2019-04-25. https://linuxcontainers.org/.
75. Kamp PH, Watson RN. Jails: Confining the omnipotent root. In: Proceedings of the 2nd International SANE Conference, vol. 43; 2000. p. 116.
76. Capuccini M, Dahlö M, Toor S, Spjuth O. Supporting data for "MaRe: Processing Big Data with Application Containers on Apache Spark" GigaScience Database 2020;http://dx.doi.org/10.5524/10073.
77. Toor S, Lindberg M, Falman I, Vallin A, Mohill O, Freyhult P, et al. SNIC science cloud (SSC): A national-scale cloud infrastructure for swedish academia. In: 2017 IEEE 13th International Conference on e-Science (e-Science) IEEE; 2017. p. 219–227.

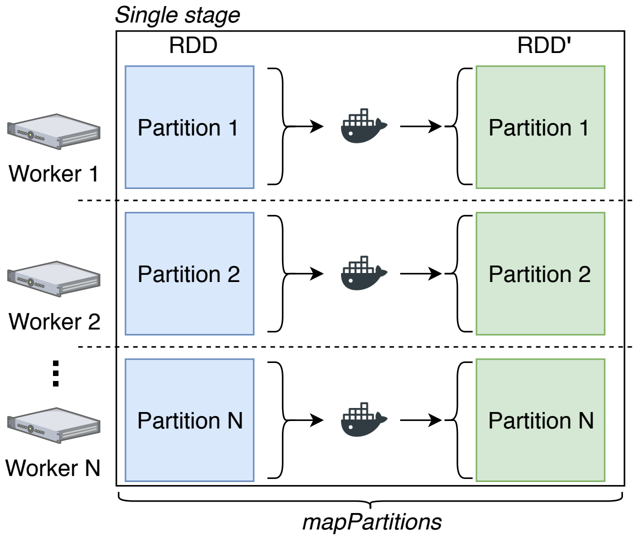

Figure 1. Execution diagram for the `map` primitive. The primitive takes an RDD that is partitioned over N nodes, it transforms each partition using a Docker container and it returns a new RDD'. The logic is implemented using *mapPartitions* from the RDD API. Since *mapPartitions* generates a single stage, data is not shuffled between nodes.

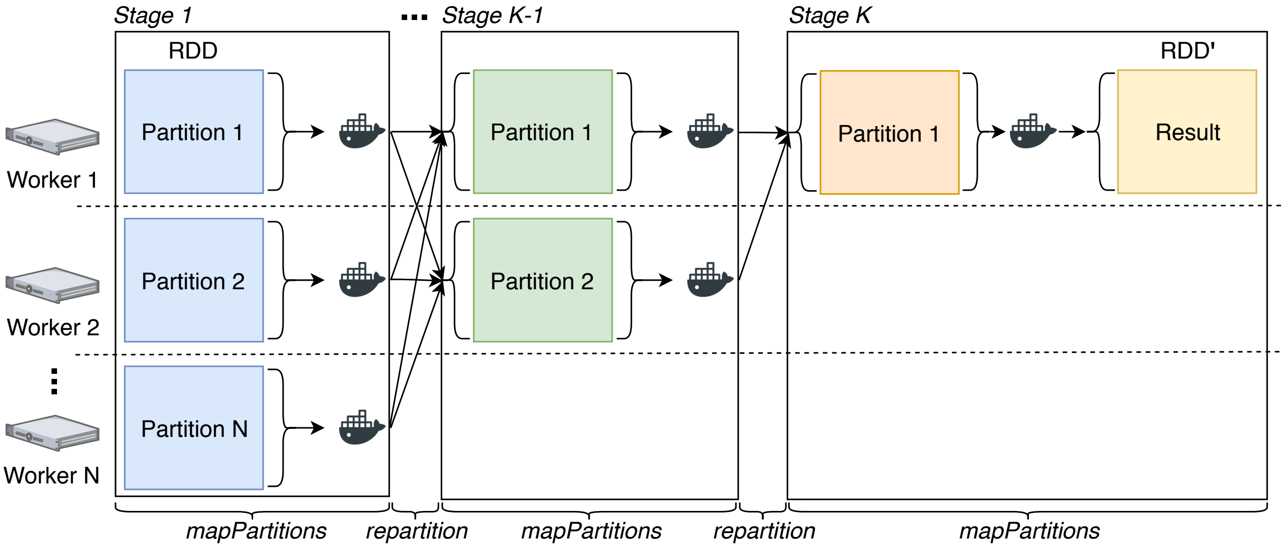

Figure 2. Execution diagram for the `reduce` primitive. The primitive takes an input RDD, partitioned over N nodes, and it iteratively aggregates records using a Docker container, reducing the number of partition until an RDD', containing a single result partition, is returned. The logic is implemented using *mapPartitions* and *repartition* from the RDD API, to aggregate records in partitions and to decrease the number of partitions respectively. Since `repartition` is called in each of the K iterations, K stages are generated, giving place to K data shuffles.

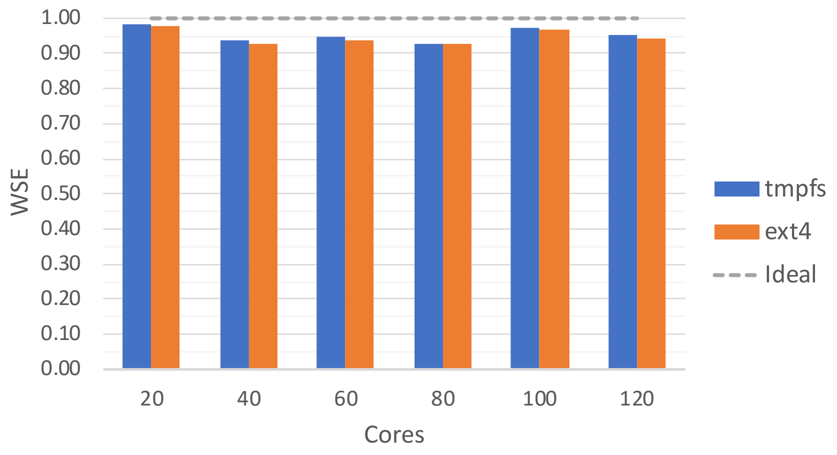

Figure 3. WSE for the VS application implemented in MaRe (listing 2). The results are produced by using SureChEMBL as input and we show the WSE when using *tmpfs* and *ext4* as temporary mount point for passing the data to the containers.

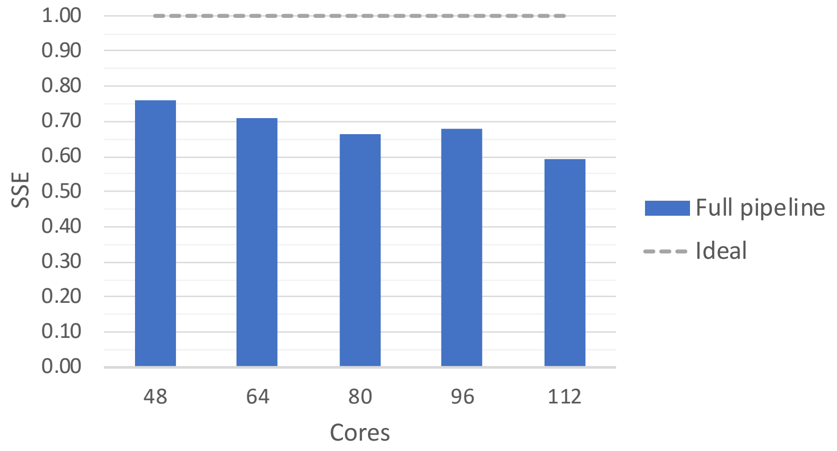

Figure 4. SSE for the SNP calling implemented in MaRe (listing 3). The results are produced by using a full individual dataset from the 1KGP as input.

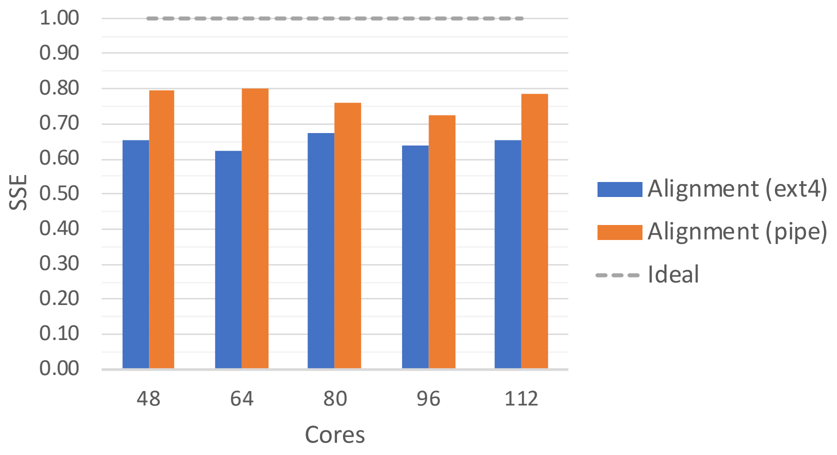

Figure 5. SSE for the SNP calling alignment stage implemented in MaRe (listing 3, lines 1 to 13). The results are produced by using a full individual dataset from the 1KGP as input and we show the SSE when using an SSD-based, *ext4* temporary mount point as well as Unix pipes for passing the data to the containers.

Click here to access/download;Figure;figure_1.tiff

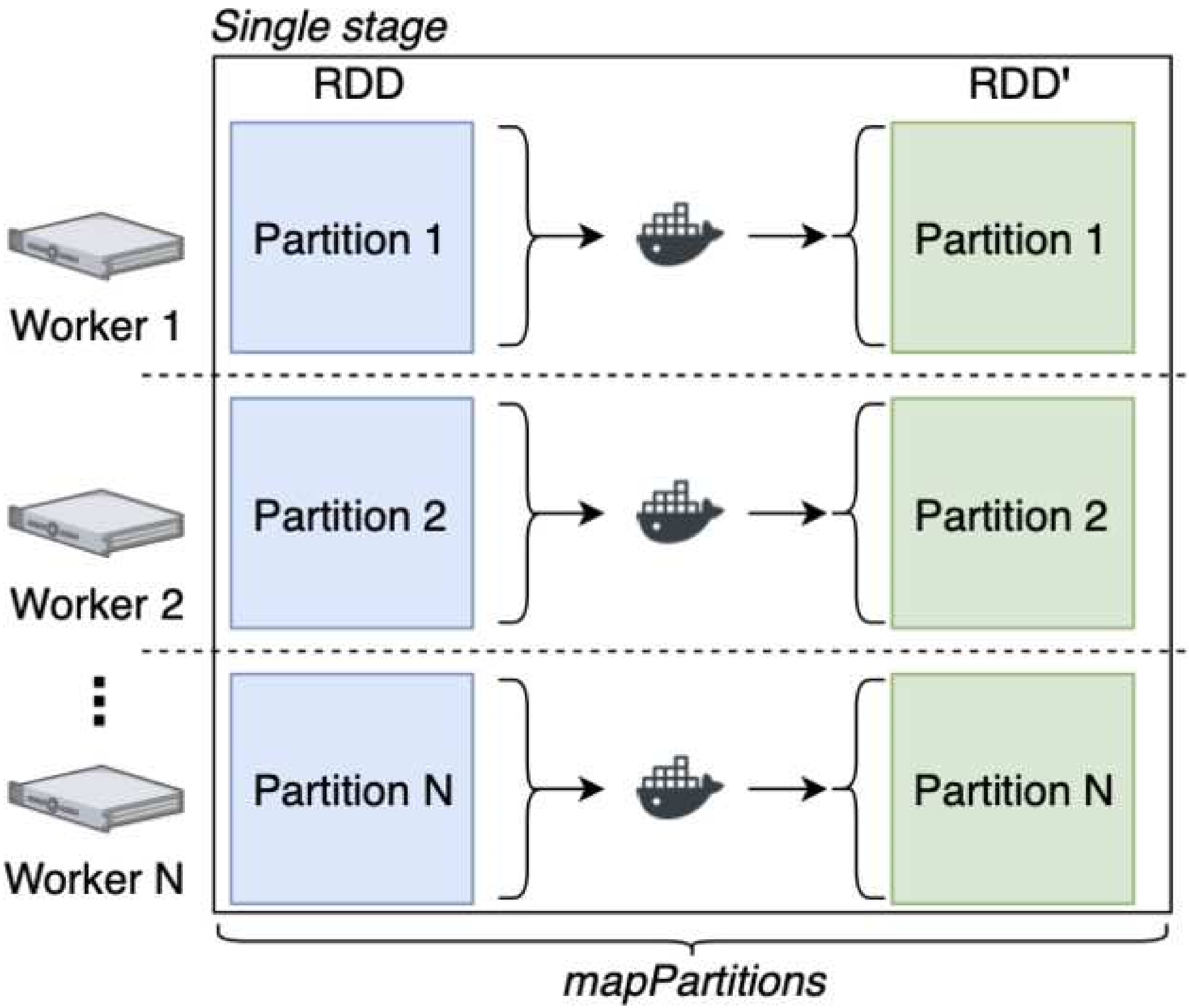

Click here to access/download;Figure;figure_2.tiff

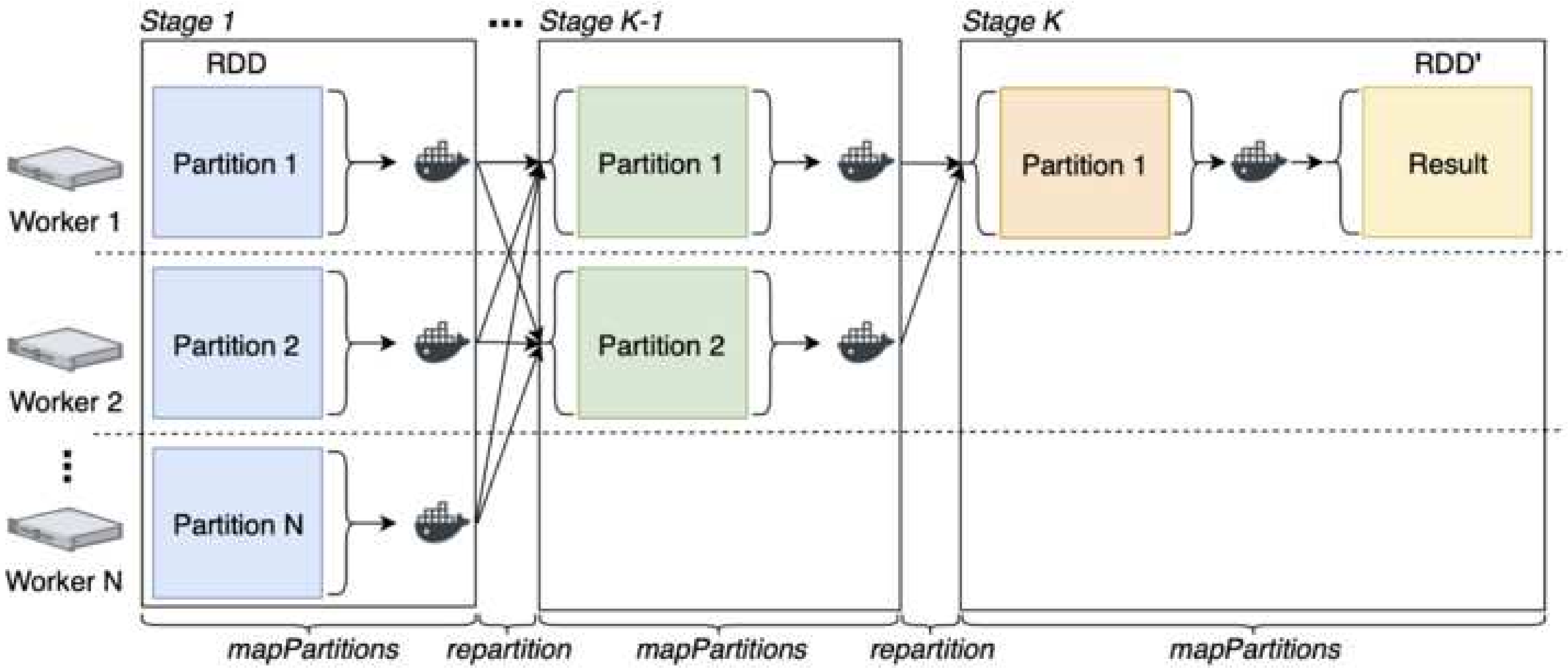

Click here to access/download;Figure;figure_3.tiff

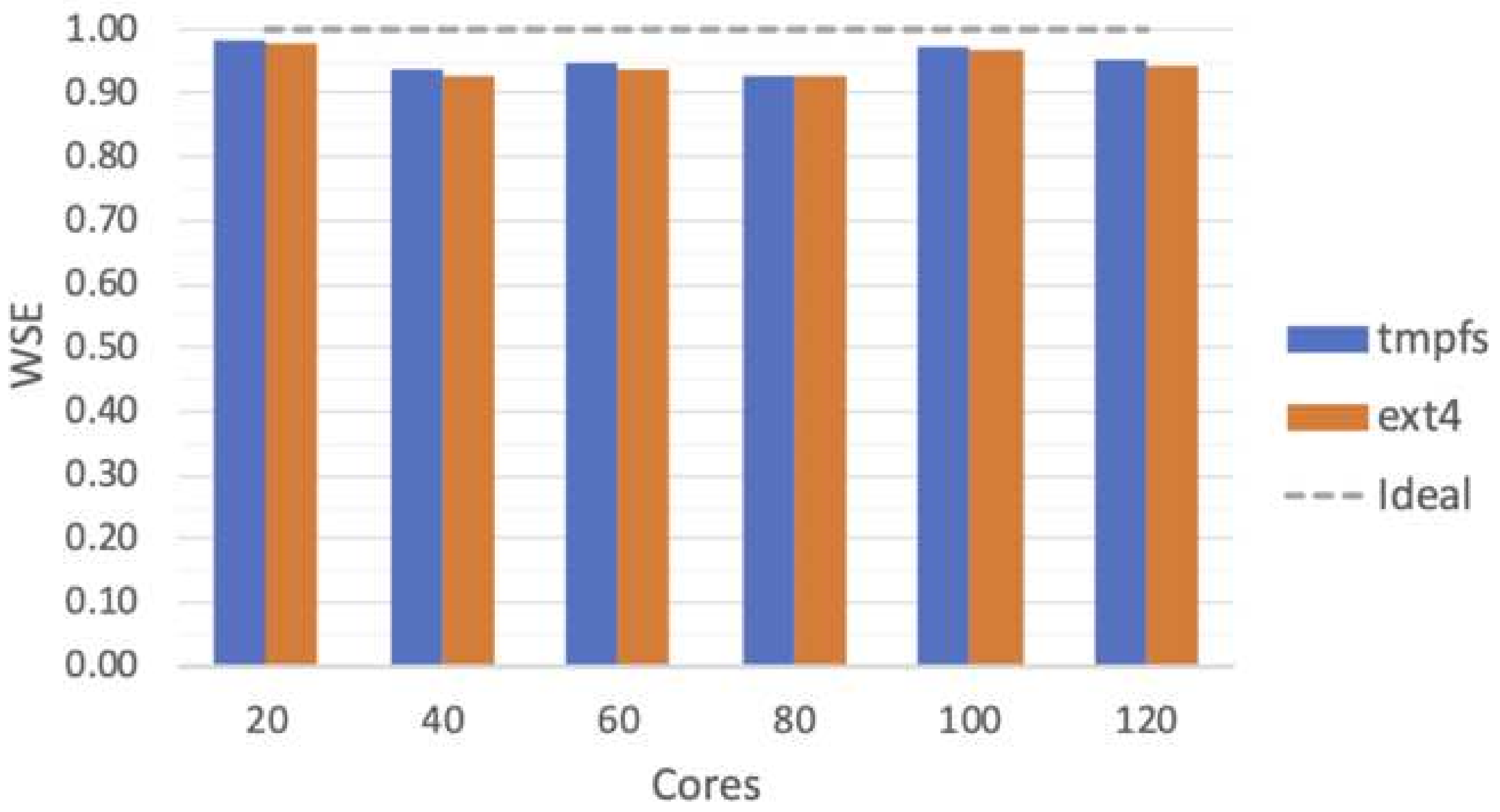

Click here to access/download;Figure;figure_4.tiff

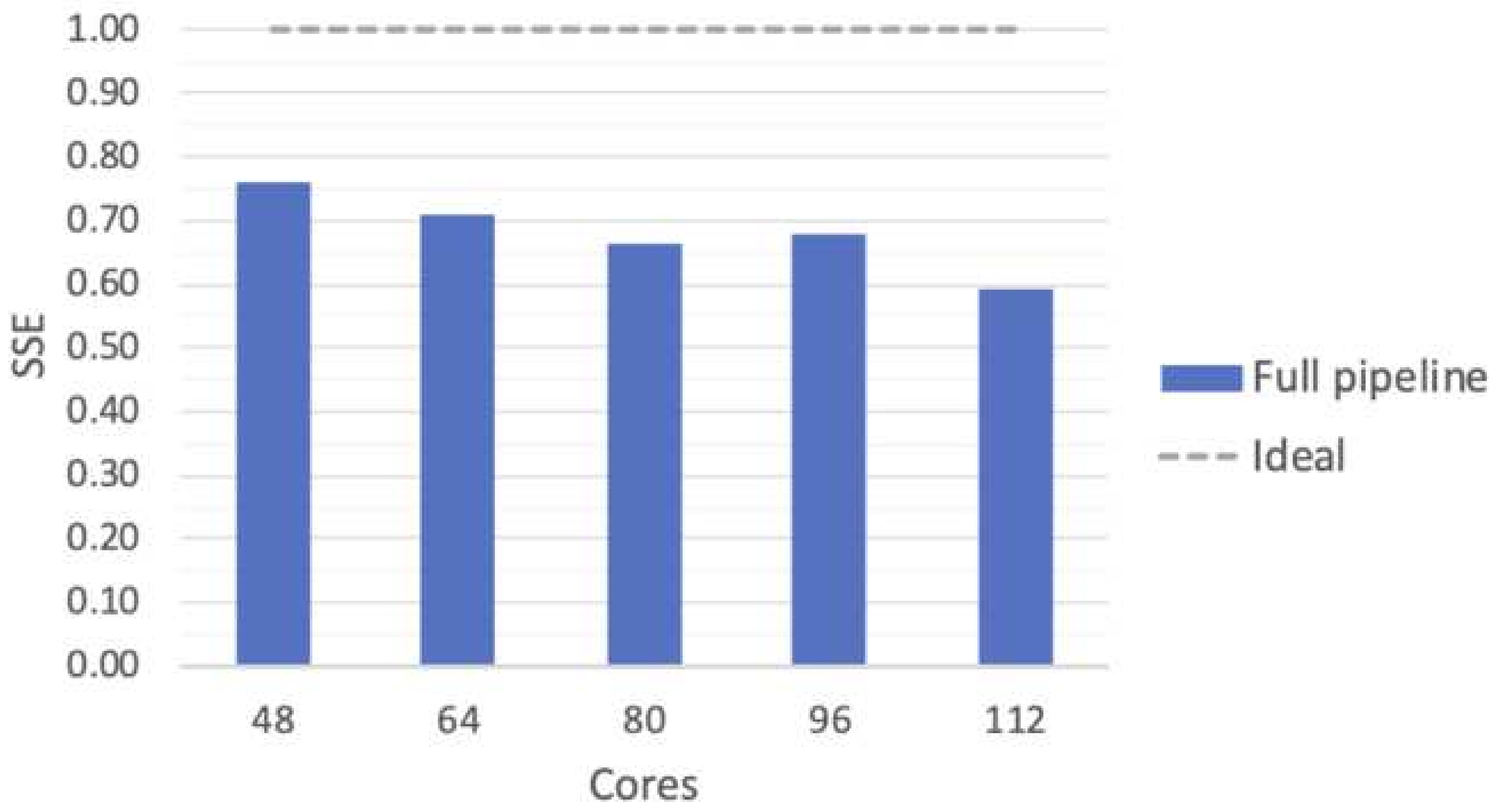

Click here to access/download;Figure;figure_5.tiff

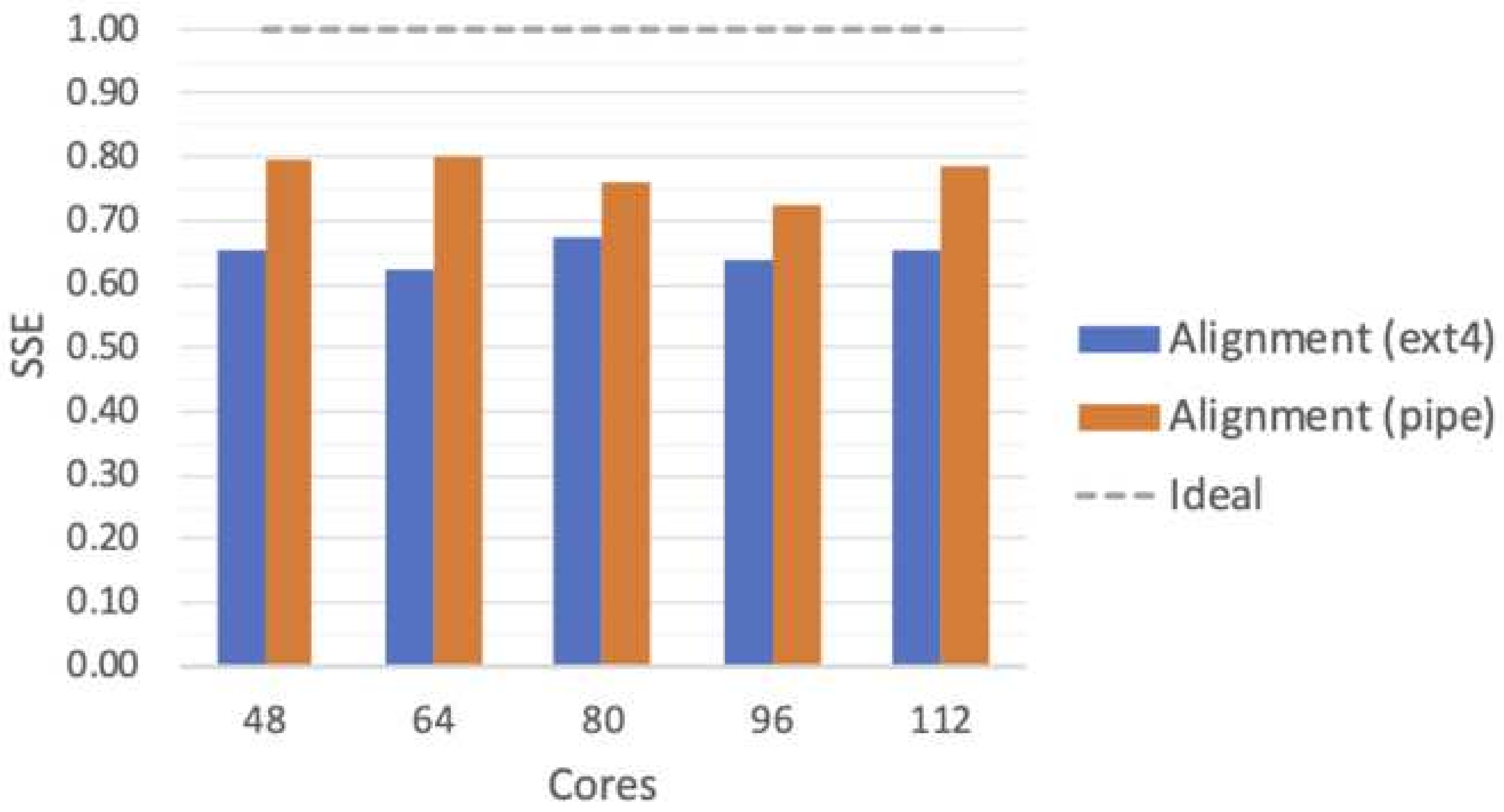

Click here to access/download;Personal Cover;MaRe Rebuttal 2.pdf

Dear Editor,

Please find attached our revised manuscript “MaRe: Processing Big Data with Application Containers on Apache Spark” (GIGA-D-19-00170) submitted for publication in GigaScience.

We are pleased with the reviewer's positive outlook.

Below we provide responses to the remaining issues pointed out by the editor and describe the updates we have made in the revised version of our manuscript. We hope that you now find it suitable for publication.

Sincerely,

Marco Capuccini and co-authors

> Prior to publication, please include a citation to your upcoming GigaDB dataset (including the DOI link) to your reference list, and cite this by number in the data availability section

We added the citation to the GigaDB dataset in the data availability section.

> In the data availability section, please also include the relevant citation (and BioProject accession number(s)) for the 1000 Genomes data

We added the citation to the 1KGP publication and its BioProject accession number in the data availability section.

> please also consider to mention details on exactly which data from ZINC was used, to help reproducibility

We added the URL to the specific ZINC subset that we used in the data availability section.
