# Supplementary material for: MaRe: Processing Big Data with application containers on Apache Spark
Source: Gigascience. 2020 May 5;9(5):giaa042. doi: 10.1093/gigascience/giaa042 (PMC7199472; doi:10.1093/gigascience/giaa042)
Supplement: giaa042_Response_to_Reviewer_Comments_Original_Submission [file giaa042_response_to_reviewer_comments_original_submission.pdf]

Author's Response To Reviewer Comments

Close

Dear Editor,

Please find attached our revised manuscript "MaRe: Processing Big Data with Application Containers on Apache Spark" (GIGA-D-19-00170) submitted for publication in GigaScience.

We thank the reviewers for constructive criticism and in particular for suggesting to improve the evaluation section of our manuscript. We have now run the requested comparative experimentation where possible and included the results in the revised version. We believe that the scope and usefulness of the framework are now properly identified and discussed. In addition, we improved the sections that the reviewers found unclear.

We also have registered MaRe on SciCrunch.org and added its resource ID to the manuscript. Further, we have now specified the full URL to the 1000 genome project subset that we have utilized in the analysis.

Below we provide responses and comments to the reviewer's remarks and describe the updates we have made in the revised version of our manuscript. We hope that you now find it suitable for publication.

Sincerely,

Marco Capuccini and co-authors

Additional changes triggered by both reviewers comments
Even if not asked by the reviewers we did some minor changes to the evaluation section, which were triggered by the comparative experimentation.

First, the scaling efficiency measures in the first version of the manuscript were relative, meaning that we were computing the measures using the MaRe parallelization on a single node. When doing a comparative experimentation we instead need an absolute baseline to compute the measures, hence the scaling efficiency for both of the two use cases is now computed using the dockerized tools, built-in parallelization on a single node. We updated the evaluation section accordingly.

Second, when running the comparative experiments we were initially getting inconsistent results as the worker node flavors that we were previously using allowed overcommitted CPUs. This means that when increasing the number of nodes we did not always get the real parallelism that we were expecting. Changing the worker node flavor to one that does not allow overcommitment solved the problem. We updated the evaluation section describing the specifications of such flavor. Please note that we use slightly different node flavors in the two use cases now. In particular for the genomics use case we use a flavor with local SSD drive, which allows for materializing larger partitions faster on disk. As this kind of machines are scarce in our cloud provider, we scaled the analysis only up to 112 cores in the new version of the manuscript.

Finally, we switched the scaling efficiency metric for the genomics use case to Strong Scaling Efficiency (SSE). In the previous version of the manuscript the Weak Scaling Efficiency (WSE) was calculated without downsampling the input reference genome, thus giving a poor estimate of the metric. As there is no straightforward way of downsampling the reference genome without altering the behaviour of the tools, we kept the input data size fixed when increasing the parallelism in the new version of the study; thus computing SSE instead of WSE. We updated the evaluation section of the paper accordingly.

Reviewer #1
> I found the thesis of the paper to be interesting but a bit confusing. The title of the paper says that MaRe is a MapReduce oriented framework for processing Big Data. Then, it is said during the abstract that MaRe is a (new?) programming model. However, further discussion reveals that the programming

model proposed by MaRe is essentially (a subset of) the same of Spark, with the only major difference being the ability to interact with external programs in a more seamless way than using the primitives coming with Spark.

> To this end, I think the authors should better clarify their contribution and, probably, put it in the right perspective. Namely, MaRe would be better presented as a software library acting as a wrapper for a Spark RDD and aiming to simplify the integration with external programs.

Thanks for pointing this out. Our initial reasoning was to see MaRe as a new programming model on top of Spark, but we agree that this can be confusing. To make our contribution more clear we updated the title of our manuscript, the abstract and the summary points in the last paragraph of the introduction.

> I also think the authors missed one important point while developing their work. Ok, I am aware of the best practices encouraging the reuse of existing tools, but I would like to know if, using MaRe, I have to suffer from, let's say, a 10x slowdown with respect to a native implementation. Or, I would like to know what is the speedup achievable with respect to the usage of the standard facilities coming with Spark for running external processes. Instead, there is no evaluation of these cases. I think there are at least two solutions alternative to MaRe that should be considered in a comparative experimentation:

> - the transformation to apply to a certain dataset is not delegated to an external tool, but natively implemented in Spark using the language of choice

We understand your point. However, if some native Spark implementations of the tools used in the presented benchmarks were available there would be no need to reimplement them using MaRe. We could consider such implementations as existing tools and definitely encourage using them instead of our programming library. To the best of our knowledge, the only available Spark-based implementation of virtual screening (use case 1) was presented in our previous papers [1,2] and the only Spark-based implementation of genomics pipelines (use case 2) that has reached production readiness is ADAM [3]. Made an exception for a few preprocessing steps in ADAM, both of these existing implementations delegate data processing to external tools using pipes; the data is not solely processed using the language of choice.

Reusing existing tools is often the case in bioinformatics data processing as the effort of reimplementing single-node tools is seldom sustainable. Convincing arguments are the bioinformatics data pipelines available in repositories such as nf-core [4]. Also, another interesting supporting fact is that the development of the Spark-native tools in the GATK suite started in 2016 (this can be checked on GitHub https://github.com/broadinstitute/gatk) and even if backed by the Broad Institute still failed to produce a stable release; besides we, despite quite a lot of effort, couldn’t get the current beta to run on our cluster without errors. This clearly shows how much effort needs to be put in reimplementing such tools natively in Spark.

We expanded the first paragraph of the evaluation section to make our argument clear in the manuscript. Also, we now state clearly in the second last paragraph of the “discussion and conclusions” section that ADAM still relies on external tools to run real-world use cases.

> - the transformation is run through an external program by using the 'pipe' facility available with Spark.

Thanks for suggesting this comparison. Testing directly against RDD pipe would not make a fair comparison because such a method starts an instance of the external tool for each RDD record, thus introducing a considerable overhead. Please notice that MaRe feeds entire RDD partitions to the containers, hence generating way less tool startup overhead. However, similarly to what it was done in ADAM [3] and in our previous virtual screening implementation [1,2], for the revised manuscript we implemented a pipePartition method that pipes entire RDD partitions to the external tools and ran the comparison against it, where allowed by the external tools. In the added benchmarks, the only tool that allows for inputting the data via standard input is BWA, so this comparison was possible only for the alignment portion of the second benchmark. Please refer to figure 5, and its referencing paragraph in the updated evaluation section to see the results of such comparison.

> As an alternative, if the target application does not support the possibility of taking its input from the stdin, the input data is preliminarly saved in a file (e.g., using /tmpfs as MaRe does) and, then, it is used to run the external program.

As the reviewer acknowledges, copying/saving data on a preliminary file is exactly what MaRe does, so there would be no difference in performance when doing it manually in Spark. However, this would take many lines of codes, especially when implementing the reduce method, while MaRe makes it seamless. We believe this to be already clear in the implementation section of the manuscript.

> Along this line, another point that would have required a better investigation is the choice of the solution to be used for storing temporary data to be processed by an external program. To this end, the solution chosen by the authors is to temporarily store data in memory using /tmpfs. I may be wrong, but this should mean that, at some point during the execution of an external process, the overall amount of available memory is decreased because input and/or output data is represented twice. This may have important consequences in processes where there is a high degree of parallelism and the amount of memory for executor is limited.

Thanks for pointing this out. It is true that by materializing the data on tmpfs we need twice as much memory for representing the partitions. However, please notice that Spark does not load partitions all at once, but it does it sequentially as resources become available. Since the partition size is configurable in Spark, one can tune it so that the total required memory will not exceed the available resources. Also notice that the partition size in Spark is equal to the block size in HDFS (128MB). This means that for a 8-cores machine a user would need 2GB of memory for representing the partitions twice. This is in most cases acceptable in modern data centers.

Representing data twice becomes a problem only when the user needs to aggregate large amounts of data on a single partition. This is necessary in our second benchmark, as GATK requires to see entire chromosomes at once. In such case a disk mount can be used instead of tmpfs; in our updated benchmark we used a local SSD drive.

We expanded the “data handling” section of the paper to make these points more clear.

> Conversely, the choice of storing this data on a persistent storage rather than in memory would be able to overcome this problem but would severely affect the performance of a process. These issues are briefly mentioned in the 'Discussion and conclusions section', while they would have required a much deeper investigation.

Thanks for pointing this out. An experimental comparison between tmpfs and persistent storage is possible for the virtual screening use case; please recall that for the other use case the intermediate partitions are too large to fit tmpfs. Please refer to figure 1 and its referencing text for the results of such comparison.

Surprisingly, there is very little overhead introduced by writing temporary data on the persistent disk; we used a regular block storage served over the network instead of SSD to evaluate this in the most penalizing settings. The reason why little overhead is introduced is that the partitions can be copied to the persistent disk relatively fast before the docker containers are started (recall that MaRe runs the tools for entire partitions and not record-wise). Then, since the container running time dominates over the data copying time, the total cost in terms of total running time is roughly the same. We update the second paragraph of the “discussion and conclusion” section accordingly.

> There are also some typos spread across the paper, such as:
> - Section 'Findings'.'Background and Purposes', page 2 : 'Finally by supporting Docker,' should be 'Finally, by supporting Docker'

Fixed.

> - Section 'Mare'.'Implementation', page 3: 'within each partitions' should be 'within each partition'

Fixed.

> - Section 'Evaluation', page 4 : 'Amanzon' should be 'Amazon'

Fixed.

> Finally, I think that the authors should put less emphasis on the possibility to ingest data from

heterogeneous cloud resources as it is essentially inherited for free from Spark.

This is a good point. We removed this from the last paragraph of the introduction and we left out the benchmarks against multiple cloud storages.

Reviewer #2
> This first half of this work describes MaRe, a useful addition to the toolbox for scaling genomics analysis: a relatively simple approach to distributing container-based data-intensive analysis, based on MapReduce. The authors implement the framework in a sensible fashion, taking advantage of the various benefits of Apache Spark. The framework seems reasonable and useful.

> I am not convinced of the second part of the paper, which looks to evaluate MaRe using two real world applications. Admittedly it is not trivial to implement a distributive framework for generic applications that scales well, but that is sort of the point of the paper. Some specific concerns are around showing the the approach works for what is essentially a trivial distribution problem - where the data per job is small and jobs are relatively transactional and independent - but the major point of a general framework is that it is useful for more complex tasks, which the second variant calling example is.

Thanks for raising this point. Implementing a distributed framework for scaling any kind of application is out of the scope of this paper. Here, we aim at providing an alternative to workflow systems, which are broadly used in bioinformatics, that builds on top of the Apache Spark ecosystem. While scaling independent tasks is admittedly trivial, integrating application containers in Spark, such that containerized bioinformatics pipelines can easily be expressed in a few lines of code and yet scale reasonably good is not a simple problem. This is the main achievement of the presented work.

We believe this to be already clear in the current status of the paper.

> I have the nagging feeling that the specifics of the evaluation task here were set to the advantage of the framework, and still the outcome was just OK. The problem, as always, is that the individual tasks are dependent on I/O, and as the authors identify, data distribution is the factor in this example that dominates the scalability.

Thanks for pointing this out. Our intention with the evaluation of our work was to show two use cases that are somewhat representative of two classes problems that one may encounter when distributing bioinformatics pipelines. The first use case matches perfectly the MapReduce approach implemented by MaRe, thus we are able to show a scaling efficiency that is close to ideal; not “just OK”. In the second use case we deliberately expose where MaRe falls short by setting up a scenario in which the MapReduce model is disadvantaged. In our perspective the fact that even for this kind of problem the analysis still scales “just OK” is a strength of our framework rather than a weakness.

We updated the first paragraph of the evaluation section to make this more clear.

> This excerpt from the discussions and conclusions is telling:
> "Scalability in the SNP calling analysis is reasonably good but far from optimal. The reason for this is that before running the haplotype caller, a reasonable amount of data needs to be shuffled across the nodes as GATK needs to see all of
the data for a single chromosome at once in order to function properly, thus causing a large amount of data to be materialized on disk. Such overhead can be partly mitigated by enabling data streams via standard input and output between MaRe and containers, which constitutes an area for future improvement."

> In summary I think this paper needs additional work on the evaluation to identify the scope of the usefulness of the framework.; and the evaluation section itself needs to be clearer. The authors state :"It is however important to point out that while ADAM is application specific, MaRe applies to a variety of use cases in bioinformatics and it stands out by enabling distributed SNP calling in less than 50 lines of code."
If that's the case, I think the paper needs to identify and discuss the performance that can be expected across different types of use cases, and why.

Please refer to the previous point. Our intention with the two use cases is to show two classes of problems for which one can expect ideal or suboptimal performance. We updated the first paragraph of the evaluation section to make this more clear.

> As a suggestion, a comparison to ADAM leading to a discussion of what the fundamental challenges of scaling I/O intensive tasks are and how that might map to different common tasks in bioinformatics, would be useful.

Thanks for suggesting a comparison with ADAM. We realized that we did not state explicitly that ADAM implements only a few preprocessing steps of the SNP pipeline [3]. Near-ideal scalability is shown in [3] only for these preprocessing steps, however in real-world settings some external tools would be needed to run a complete analysis. Indeed, ADAM provides a modified version of RDD pipes for running external tools [5], but no study has yet quantified what kind of performance one can expect when running external tools in ADAM. One major problem with pipes is that not every external tool is capable of accepting data via standard input. GATK, which provides a state of the art variant caller, is an example of such a tool. For this reason we were not able to reproduce the same pipeline that we ran for our genomics benchmark using ADAM. However, the first part of the pipeline uses a tool that can read data via standard input (BWA). Hence, we could compare the scaling efficiency that we obtained using MaRe, for this first portion of the pipeline, with a similar implementation of the modified RDD pipe routine included in ADAM. We prefered to simply reimplement this routine as the ADAM project requires many dependencies that would be hard to bring in our environment. Figure 5, and its referencing text, present the results of this new comparison. We also added the discussed details about ADAM in the fourth paragraph of the “discussion and conclusion” section.

> Or at least a discussion of the characteristics of problems that MaRe would suit.

We expanded the second last paragraph of the “discussion and conclusion” section to point out where MaRe falls short. In summary, when records in large partitions need to be processed all together it is not reasonable to expect ideal scalability, however given the effort that sometimes need to be put in reimplementing bioinformatics tools in Spark, scaling the analyses in MaRe could prove to be more sustainable.

> There are also a handful of expression and grammatical errors:

>> "Such amounts of data poses major challenges for scientific analyses"

> such amounts of data _pose_ major challenges…

Fixed.

> "but also prohibitively expensive in terms of power consumption, estimated to be in the order of several hundred thousand dollars per year"

>> is this for all life science data transfer over the entire planet? The sentence needs some qualification.

Referring to the cited work, this is for a single next-generation HPC cluster. We added this detail to the sentence to make it more clear.

> "In summary, the key contribution of the presented work are:"

> In summary, the key contributions of the presented work are:

Fixed.

> "We demonstrate data ingestion from three large-scale storage systems: Hadoop Distributed File System (HDFS) [41], Swift [42] and Amanzon S3 [43]. In our settings HDFS was co-located with the Apache Spark"

>> Amazon S3

Fixed.

> "One of the advantages of Apache Spark over other MapReduce-like systems is the ability of retaining data in memory. Hence, for better performance it is preferable to keep RDD records in memory when mounting them in the containers."

> the word 'Hence' doesn't make logical sense in this sentence.

We removed the sentence starting with “Hence”.

> "The full benchmark runs in ~1.8 hours when using 128 vCPUs, including data ingestion from S3."

>> It's impossible to know what this means as it is given in isolation - no context and no comparison to performance after. I think this is an example of a significant flaw in the paper, in that the theoretical and implementation parts seem fine, but the evaluation is not really convincing.

This is a good point. We added some details about the running time on a single node to add some context and comparison of the statement. Please refer to the second last paragraph of the “Virtual Screening” section and to the second last paragraph of the “Single Nucleotide Polymorphism” section.

References

1. Capuccini M, Ahmed L, Schaal W, Laure E, Spjuth O. Large-scale virtual screening on public cloud resources with Apache Spark. J Cheminform. 2017;9: 15.
2. Ahmed L, Georgiev V, Capuccini M, Toor S, Schaal W, Laure E, et al. Efficient iterative virtual screening with Apache Spark and conformal prediction. J Cheminform. 2018;10: 8.
3. Nothaft FA, Linderman M, Franklin MJ, Joseph AD, Patterson DA, Massie M, et al. Rethinking Data-Intensive Science Using Scalable Analytics Systems. Proceedings of the 2015 ACM SIGMOD International Conference on Management of Data - SIGMOD ’15. 2015. doi:10.1145/2723372.2742787
4. Ewels P. nf-core. [cited 28 Jan 2020]. Available: https://nf-co.re/pipelines
5. Using ADAM’s Pipe API — bdgenomics.adam 0.23.0-SNAPSHOT documentation. [cited 29 Jan 2020]. Available: https://adam.readthedocs.io/en/latest/api/pipes/

Close
