# Supplementary material for: MaRe: Processing Big Data with application containers on Apache Spark
Source: Gigascience. 2020 May 5;9(5):giaa042. doi: 10.1093/gigascience/giaa042 (PMC7199472; doi:10.1093/gigascience/giaa042)
Supplement: giaa042_Response_to_Reviewer_Comments_Revision_1 [file giaa042_response_to_reviewer_comments_revision_1.pdf]

# Author's Response To Reviewer Comments

Close

Dear Editor,

Please find attached our revised manuscript "MaRe: Processing Big Data with Application Containers on Apache Spark" (GIGA-D-19-00170) submitted for publication in GigaScience.

We are pleased with the reviewer's positive outlook.

Below we provide responses to the remaining issues pointed out by the editor and describe the updates we have made in the revised version of our manuscript. We hope that you now find it suitable for publication.

Sincerely,

Marco Capuccini and co-authors

> Prior to publication, please include a citation to your upcoming GigaDB dataset (including the DOI link) to your reference list, and cite this by number in the data availability section

We added the citation to the GigaDB dataset in the data availability section.

> In the data availability section, please also include the relevant citation (and BioProject accession number(s) ) for the 1000 Genomes data

We added the citation to the 1KGP publication and its BioProject accession number in the data availability section.

> please also consider to mention details on exactly which data from ZINC was used, to help reproducibility

We added the URL to the specific ZINC subset that we used in the data availability section.

Close
