# Supplementary material for: MaRe: Processing Big Data with application containers on Apache Spark
Source: Gigascience. 2020 May 5;9(5):giaa042. doi: 10.1093/gigascience/giaa042 (PMC7199472; doi:10.1093/gigascience/giaa042)
Supplement: giaa042_Response_to_Reviewer_Comments_Revision_2 [file giaa042_response_to_reviewer_comments_revision_2.pdf]

Author's Response To Reviewer Comments

Close

Dear Hans,

Thanks for pointing out the typo. I have now update the bibtex file and the PDF to fix it.

Regards
Marco Capuccini

Close
