# Supplementary material for: MaRe: Processing Big Data with application containers on Apache Spark
Source: Gigascience. 2020 May 5;9(5):giaa042. doi: 10.1093/gigascience/giaa042 (PMC7199472; doi:10.1093/gigascience/giaa042)
Supplement: giaa042_Reviewer_1_Report_Original_Submission — Umberto Ferraro Petrillo -- 6/14/2019 Reviewed [file giaa042_reviewer_1_report_original_submission.pdf]

Reviewer Report

Title: MaRe: Processing Big Data with Application Containers on Apache Spark

Version: Original Submission **Date:** 6/14/2019

Reviewer name: Umberto Ferraro Petrillo

Reviewer Comments to Author:

I found the thesis of the paper to be interesting but a bit confusing. The title of the paper says that MaRe is a MapReduce oriented framework for processing Big Data. Then, it is said during the abstract that MaRe is a (new?) programming model. However, further discussion reveals that the programming model proposed by MaRe is essentially (a subset of) the same of Spark, with the only major difference being the ability to interact with external programs in a more seamless way than using the primitives coming with Spark.
To this end, I think the authors should better clarify their contribution and, probably, put it in the right perspective. Namely, MaRe would be better presented as a software library acting as a wrapper for a Spark RDD and aiming to simplify the integration with external programs.
I also think the authors missed one important point while developing their work. Ok, I am aware of the best practices encouraging the reuse of existing tools, but I would like to know if, using MaRe, I have to suffer from, let's say, a 10x slowdown with respect to a native implementation. Or, I would like to know what is the speedup achievable with respect to the usage of the standard facilities coming with Spark for running external processes. Instead, there is no evaluation of these cases. I think there are at least two solutions alternative to MaRe that should be considered in a comparative experimentation:
- the transformation to apply to a certain dataset is not delegated to an external tool, but natively implemented in Spark using the language of choice
- the transformation is run through an external program by using the 'pipe' facility available with Spark. As an alternative, if the target application does not support the possibility of taking its input from the stdin, the input data is preliminarly saved in a file (e.g., using /tmpfs as MaRe does) and, then, it is used to run the external program

Along this line, another point that would have required a better investigation is the choice of the solution to be used for storing temporary data to be processed by an external program. To this end, the solution chosen by the authors is to temporarily store data in memory using /tmpfs. I may be wrong, but this should mean that, at some point during the execution of an external process, the overall amount of available memory is decreased because input and/or output data is represented twice. This may have important consequences in processes where there is a high degree of parallelism and the amount of memory for executor is limited. Conversely, the choice of storing this data on a persistent storage rather than in memory would be able to overcome this problem but would severely affect the performance of a process. These issues are briefly mentioned in the 'Discussion and conclusions section', while they would have required a much deeper investigation.
There are also some typos spread across the paper, such as:
- Section 'Findings'.'Background and Purposes', page 2 : 'Finally by supporting Docker,' should be 'Finally,

by supporting Docker'
- Section 'Mare'.'Implementation', page 3: 'within each partitions' should be 'within each partition'
- Section 'Evaluation', page 4 : 'Amanzon' should be 'Amazon'

Finally, I think that the authors should put less emphasis on the possibility to ingest data from heterogeneous cloud resources as it is essentially inherited for free from Spark.

Level of Interest

Please indicate how interesting you found the manuscript: Choose an item.

Quality of Written English

Please indicate the quality of language in the manuscript: Choose an item.

Declaration of Competing Interests

Please complete a declaration of competing interests, considering the following questions:

- Have you in the past five years received reimbursements, fees, funding, or salary from an organisation that may in any way gain or lose financially from the publication of this manuscript, either now or in the future?
- Do you hold any stocks or shares in an organisation that may in any way gain or lose financially from the publication of this manuscript, either now or in the future?
- Do you hold or are you currently applying for any patents relating to the content of the manuscript?
- Have you received reimbursements, fees, funding, or salary from an organization that holds or has applied for patents relating to the content of the manuscript?
- Do you have any other financial competing interests?
- Do you have any non-financial competing interests in relation to this paper?

If you can answer no to all of the above, write 'I declare that I have no competing interests' below. If your reply is yes to any, please give details below.

I declare that I have no competing interests

I agree to the open peer review policy of the journal. I understand that my name will be included on my report to the authors and, if the manuscript is accepted for publication, my named report including any attachments I upload will be posted on the website along with the authors' responses. I agree for my report to be made available under an Open Access Creative Commons CC-BY license (http://creativecommons.org/licenses/by/4.0/). I understand that any comments which I do not wish to be included in my named report can be included as confidential comments to the editors, which will not be published.

Choose an item.

To further support our reviewers, we have joined with Publons, where you can gain additional credit to further highlight your hard work (see: https://publons.com/journal/530/gigascience). On publication of this paper, your review will be automatically added to Publons, you can then choose whether or not to claim your Publons credit. I understand this statement.

Yes Choose an item.
