# Supplementary material for: MaRe: Processing Big Data with application containers on Apache Spark
Source: Gigascience. 2020 May 5;9(5):giaa042. doi: 10.1093/gigascience/giaa042 (PMC7199472; doi:10.1093/gigascience/giaa042)
Supplement: giaa042_Reviewer_2_Report_Original_Submission — Andrew Lonie -- 6/24/2019 Reviewed [file giaa042_reviewer_2_report_original_submission.pdf]

Reviewer Report

Title: MaRe: Processing Big Data with Application Containers on Apache Spark

Version: Original Submission **Date:** 6/24/2019

Reviewer name: Andrew Lonie

Reviewer Comments to Author:

This first half of this work describes MaRe, a useful addition to the toolbox for scaling genomics analysis: a relatively simple approach to distributing container-based data-intensive analysis, based on MapReduce. The authors implement the framework in a sensible fashion, taking advantage of the various benefits of Apache Spark. The framework seems reasonable and useful.

I am not convinced of the second part of the paper, which looks to evaluate MaRe using two real world applications. Admittedly it is not trivial to implement a distributive framework for generic applications that scales well, but that is sort of the point of the paper. Some specific concerns are around showing the the approach works for what is essentially a trivial distribution problem - where the data per job is small and jobs are relatively transactional and independent - but the major point of a general framework is that it is useful for more complex tasks, which the second variant calling example is. I have the nagging feeling that the specifics of the evaluation task here were set to the advantage of the framework, and still the outcome was just OK. The problem, as always, is that the individual tasks are dependent on I/O, and as the authors identify, data distribution is the factor in this example that dominates the scalability.

This excerpt from the discussions and conclusions is telling:

"Scalability in the SNP calling analysis is reasonably good but far from optimal. The reason for this is that before running the haplotype caller, a reasonable amount of data needs to be shuffled across the nodes as GATK needs to see all of

the data for a single chromosome at once in order to function properly, thus causing a large amount of data to be materialized on disk. Such overhead can be partly mitigated by enabling data streams via standard input and output between MaRe and containers, which constitutes an area for future improvement."

In summary I think this paper needs additional work on the evaluation to identify the scope of the usefulness of the framework.; and the evaluation section itself needs to be clearer. The authors state :"It is however important to point out that while ADAM is application specific, MaRe applies to a variety of use cases in bioinformatics and it stands out by enabling distributed SNP calling in less than 50 lines of code."

If that's the case, I think the paper needs to identify and discuss the performance that can be expected across different types of use cases, and why. As a suggestion, a comparison to ADAM leading to a discussion of what the fundamental challenges of scaling I/O intensive tasks are and how that might map to different common tasks in bioinformatics, would be useful. Or at least a discussion of the characteristics of problems that MaRe would suit.

There are also a handful of expression and grammatical errors:

"Such amounts of data poses major challenges for scientific analyses"

> such amounts of data _pose_ major challenges...
"but also prohibitively expensive in terms of power consumption, estimated to be in the order of several hundred thousand dollars per year"
> is this for all life science data transfer over the entire planet? The sentence needs some qualification.
"In summary, the key contribution of the presented work are:"
> In summary, the key contributions of the presented work are:
"We demonstrate data ingestion from three large-scale storage systems: Hadoop Distributed File System (HDFS) [41], Swift [42] and Amanzon S3 [43]. In our settings HDFS was co-located with the Apache Spark"
> Amazon S3
"One of the advantages of Apache Spark over other MapReduce-like systems is the ability of retaining data in memory. Hence, for better performance it is preferable to keep RDD records in memory when mounting them in the containers."
> the word 'Hence' doesn't make logical sense in this sentence.
"The full benchmark runs in ~1.8 hours when using 128 vCPUs, including data ingestion from S3."
> It's impossible to know what this means as it is given in isolation - no context and no comparison to performance after. I think this is an example of a significant flaw in the paper, in that the theoretical and implementation parts seem fine, but the evaluation is not really convincing.

Level of Interest

Please indicate how interesting you found the manuscript: Choose an item.

Quality of Written English

Please indicate the quality of language in the manuscript: Choose an item.

Declaration of Competing Interests

Please complete a declaration of competing interests, considering the following questions:

- Have you in the past five years received reimbursements, fees, funding, or salary from an organisation that may in any way gain or lose financially from the publication of this manuscript, either now or in the future?
- Do you hold any stocks or shares in an organisation that may in any way gain or lose financially from the publication of this manuscript, either now or in the future?
- Do you hold or are you currently applying for any patents relating to the content of the manuscript?
- Have you received reimbursements, fees, funding, or salary from an organization that holds or has applied for patents relating to the content of the manuscript?
- Do you have any other financial competing interests?

- Do you have any non-financial competing interests in relation to this paper?

If you can answer no to all of the above, write 'I declare that I have no competing interests' below. If your reply is yes to any, please give details below.

I declare that I have no competing interests

I agree to the open peer review policy of the journal. I understand that my name will be included on my report to the authors and, if the manuscript is accepted for publication, my named report including any attachments I upload will be posted on the website along with the authors' responses. I agree for my report to be made available under an Open Access Creative Commons CC-BY license (http://creativecommons.org/licenses/by/4.0/). I understand that any comments which I do not wish to be included in my named report can be included as confidential comments to the editors, which will not be published.

Choose an item.

To further support our reviewers, we have joined with Publons, where you can gain additional credit to further highlight your hard work (see: https://publons.com/journal/530/gigascience). On publication of this paper, your review will be automatically added to Publons, you can then choose whether or not to claim your Publons credit. I understand this statement.

Yes Choose an item.
